# Supplementary material for: Bipartite network models to design combination therapies in acute myeloid leukaemia
Source: Nat Commun. 2022 Apr 19;13:2128. doi: 10.1038/s41467-022-29793-5 (PMC9018865; doi:10.1038/s41467-022-29793-5)
Supplement: Supplementary file 1 — Supplementary Information [file 41467_2022_29793_MOESM1_ESM.pdf]

Supplementary Figures

Supplementary Figure 1: Patient and drug nodes in the Beat AML dataset's projected networks. Both bar plots feature an x axis that corresponds to the cumulative weights (strength of nodes). On the y axis, the nodes are ranked according to their strength in the patient and drug similarity networks, respectively.

Supplementary Figure 2: The complete landscapes of interactions for all 135 drug combinations. It consists of a total of 25 positive and 20 negative combinations that multiply to three cell lines. The title of the plot and the axes names correspond to the cell lines and drug combinations examined. Additionally, Axes contains information on the dosage for each drug. The color bar legend reflects the percentage of cell viability that is inhibited by drug treatment. Additionally, a trimmed version of the landscape is provided to emphasize the synergistic effect of two drugs rather than the toxic effect of a single agent at a high dosage.

Supplementary Figure 3: The correlation coefficients of single drug responses of the top five drugs in two clusters (ten drugs) which were used for in vitro experiments. This heatmap depicts the pairwise correlation coefficients between ex vivo models in the Beat AML dataset and three cancer cell lines that we experimentally tested. The color legend is displayed along with the histogram of all pairwise correlation coefficients.

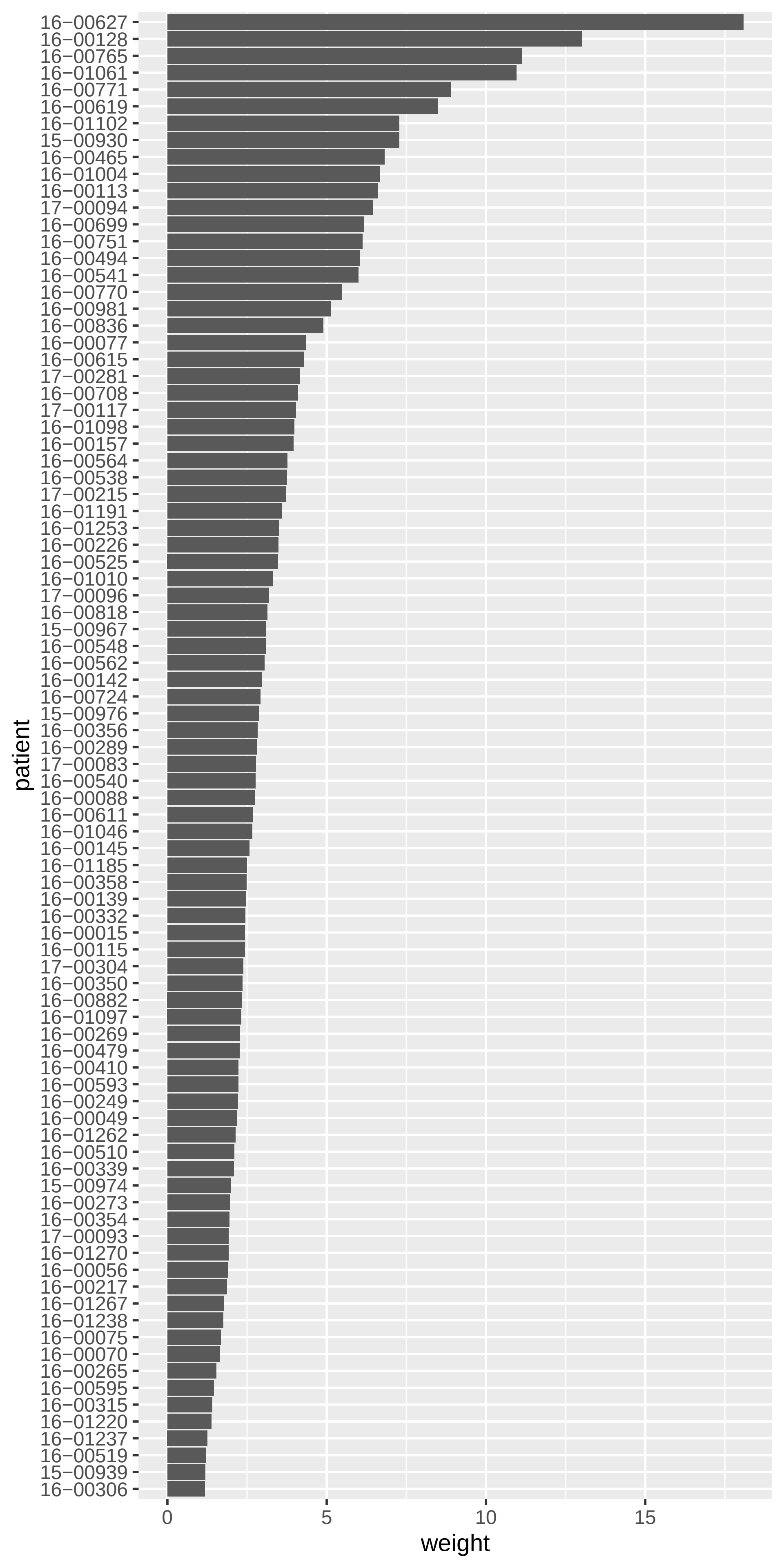
16−00627
16−00128
16−00765
16−01061
16−00771
16−00619
16−01102
15−00930
16−00465
16−01004
16−00113
17−00094
16−00699
16−00751
16−00494
16−00541
16−00770
16−00981
16−00836
16−00077
16−00615
17−00281
16−00708
17−00117
16−01098
16−00157
16−00564
16−00538
17−00215
16−01191
16−01253
16−00226
16−00525
16−01010
17−00096
16−00818
15−00967
16−00548
16−00562
16−00142
16−00724
15−00976
16−00356
16−00289
17−00083
16−00540
16−00088
16−00611
16−01046
16−00145
16−01185
16−00358
16−00139
16−00332
16−00015
16−00115
17−00304
16−00350
16−00882
16−01097
16−00269
16−00479
16−00410
16−00593
16−00249
16−00049
16−01262
16−00510
16−00339
15−00974
16−00273
16−00354
17−00093
16−01270
16−00056
16−00217
16−01267
16−01238
16−00075
16−00070
16−00265
16−00595
16−00315
16−01220
16−01237
16−00519
15−00939
16−00306
patient
0
5
10
15
weight

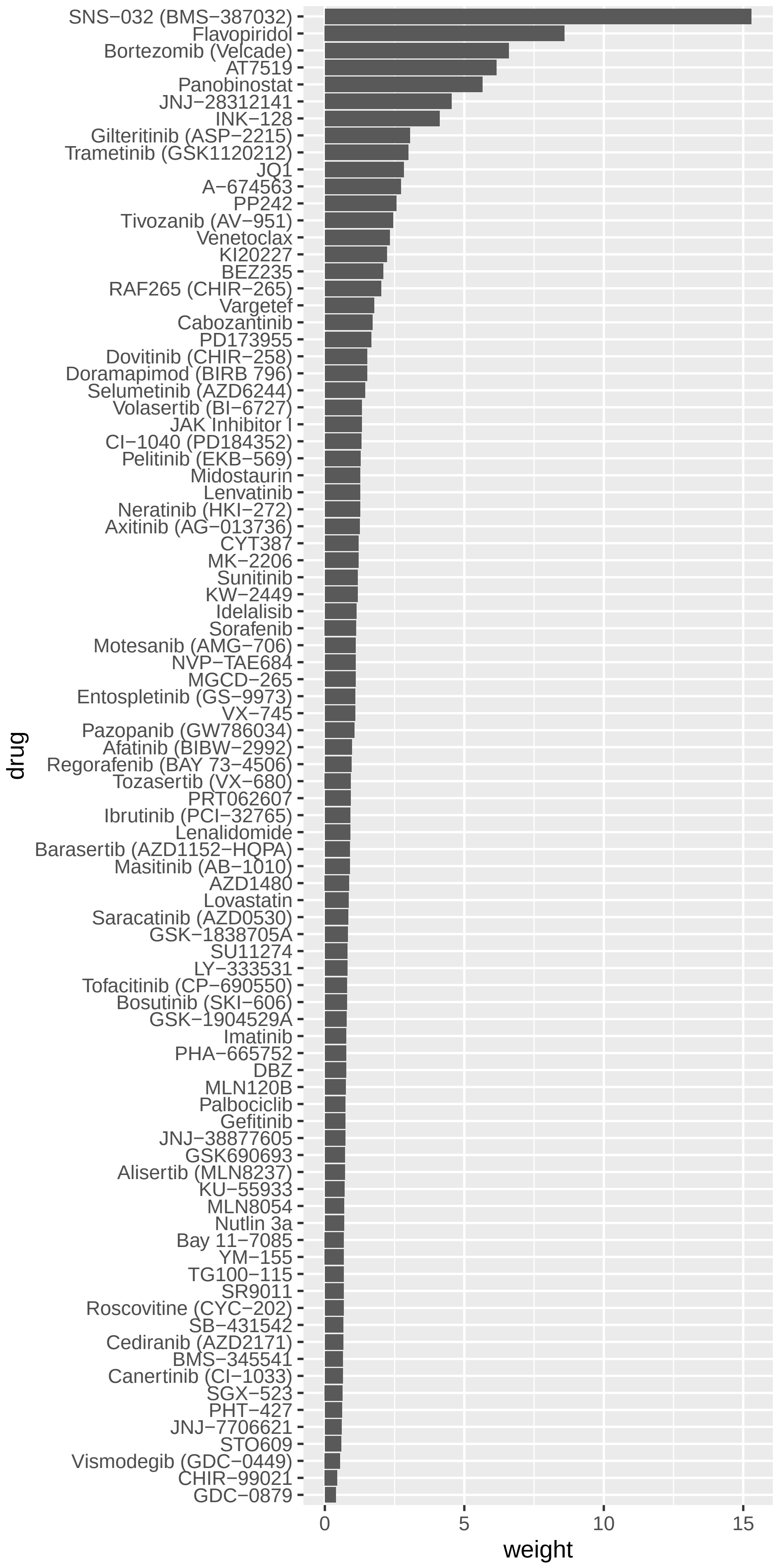

SNS−032 (BMS−387032)
Flavopiridol
Bortezomib (Velcade)
AT7519
Panobinostat
JNJ−28312141
INK−128
Gilteritinib (ASP−2215)
Trametinib (GSK1120212)
JQ1
A−674563
PP242
Tivozanib (AV−951)
Venetoclax
KI20227
BEZ235
RAF265 (CHIR−265)
Vargetef
Cabozantinib
PD173955
Dovitinib (CHIR−258)
Doramapimod (BIRB 796)
Selumetinib (AZD6244)
Volasertib (BI−6727)
JAK Inhibitor I
CI−1040 (PD184352)
Pelitinib (EKB−569)
Midostaurin
Lenvatinib
Neratinib (HKI−272)
Axitinib (AG−013736)
CYT387
MK−2206
Sunitinib
KW−2449
Idelalisib
Sorafenib
Motesanib (AMG−706)
NVP−TAE684
MGCD−265
Entospletinib (GS−9973)
VX−745
Pazopanib (GW786034)
Afatinib (BIBW−2992)
Regorafenib (BAY 73−4506)
Tozasertib (VX−680)
PRT062607
Ibrutinib (PCI−32765)
Lenalidomide
Barasertib (AZD1152−HQPA)
Masitinib (AB−1010)
AZD1480
Lovastatin
Saracatinib (AZD0530)
GSK−1838705A
SU11274
LY−333531
Tofacitinib (CP−690550)
Bosutinib (SKI−606)
GSK−1904529A
Imatinib
PHA−665752
DBZ
MLN120B
Palbociclib
Gefitinib
JNJ−38877605
GSK690693
Alisertib (MLN8237)
KU−55933
MLN8054
Nutlin 3a
Bay 11−7085
YM−155
TG100−115
SR9011
Roscovitine (CYC−202)
SB−431542
Cediranib (AZD2171)
BMS−345541
Canertinib (CI−1033)
SGX−523
PHT−427
JNJ−7706621
STO609
Vismodegib (GDC−0449)
CHIR−99021
GDC−0879
drug
0
5
10
15
weight

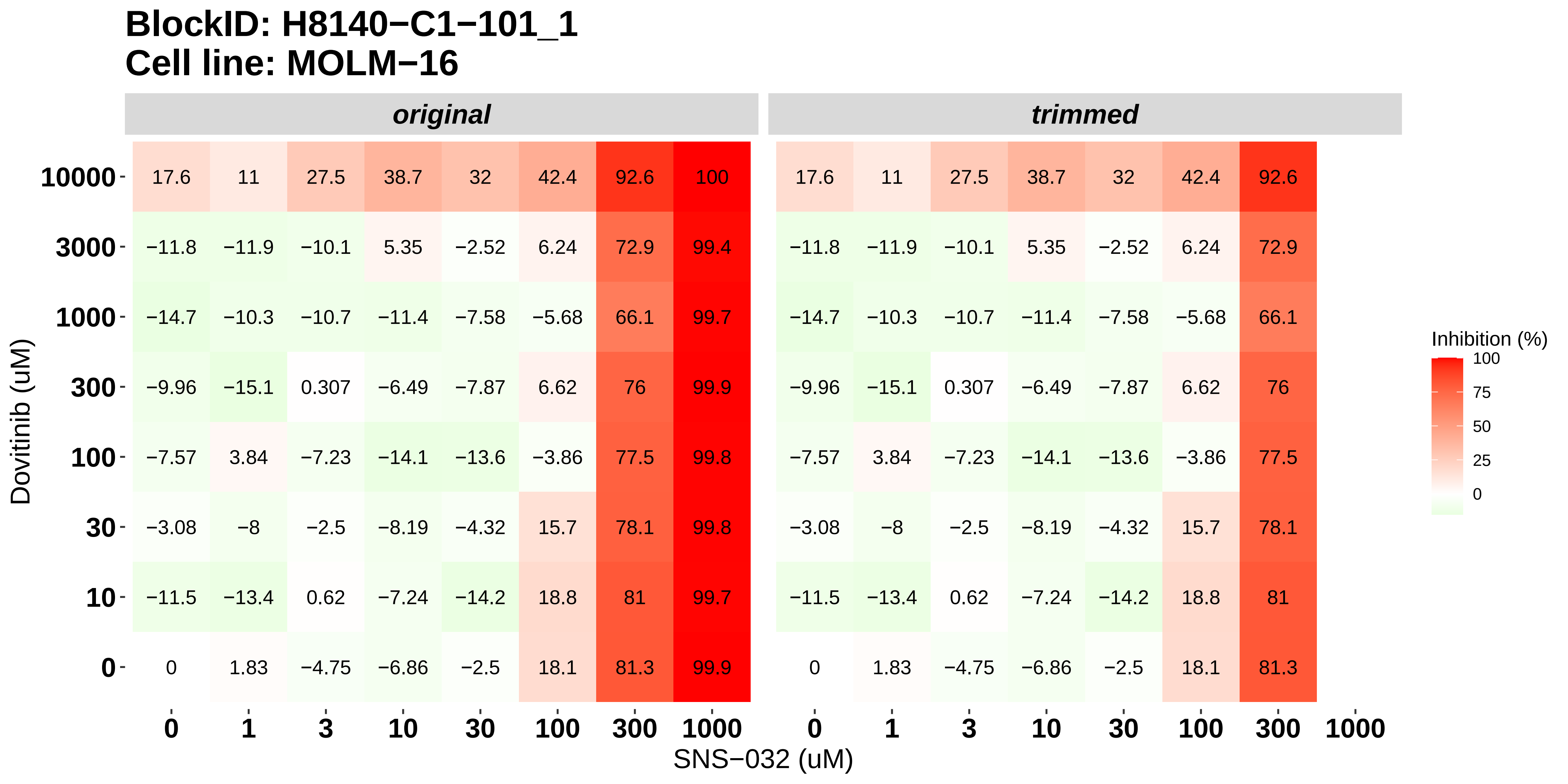

BlockID: H8140−C1−101_1
Cell line: MOLM−16
original
trimmed
10000 17.6 11 27.5 38.7 32 42.4 92.6 100
3000 −11.8 −11.9 −10.1 5.35 −2.52 6.24 72.9 99.4
1000 −14.7 −10.3 −10.7 −11.4 −7.58 −5.68 66.1 99.7
300 −9.96 −15.1 0.307 −6.49 −7.87 6.62 76 99.9
100 −7.57 3.84 −7.23 −14.1 −13.6 −3.86 77.5 99.8
30 −3.08 −8 −2.5 −8.19 −4.32 15.7 78.1 99.8
10 −11.5 −13.4 0.62 −7.24 −14.2 18.8 81 99.7
0 0 1.83 −4.75 −6.86 −2.5 18.1 81.3 99.9
17.6 11 27.5 38.7 32 42.4 92.6
−11.8 −11.9 −10.1 5.35 −2.52 6.24 72.9
−14.7 −10.3 −10.7 −11.4 −7.58 −5.68 66.1
−9.96 −15.1 0.307 −6.49 −7.87 6.62 76
−7.57 3.84 −7.23 −14.1 −13.6 −3.86 77.5
−3.08 −8 −2.5 −8.19 −4.32 15.7 78.1
−11.5 −13.4 0.62 −7.24 −14.2 18.8 81
0 1.83 −4.75 −6.86 −2.5 18.1 81.3
0 1 3 10 30 100 300 1000
0 1 3 10 30 100 300 1000
Dovitinib (uM)
SNS−032 (uM)
Inhibition (%)
100
75
50
25
0

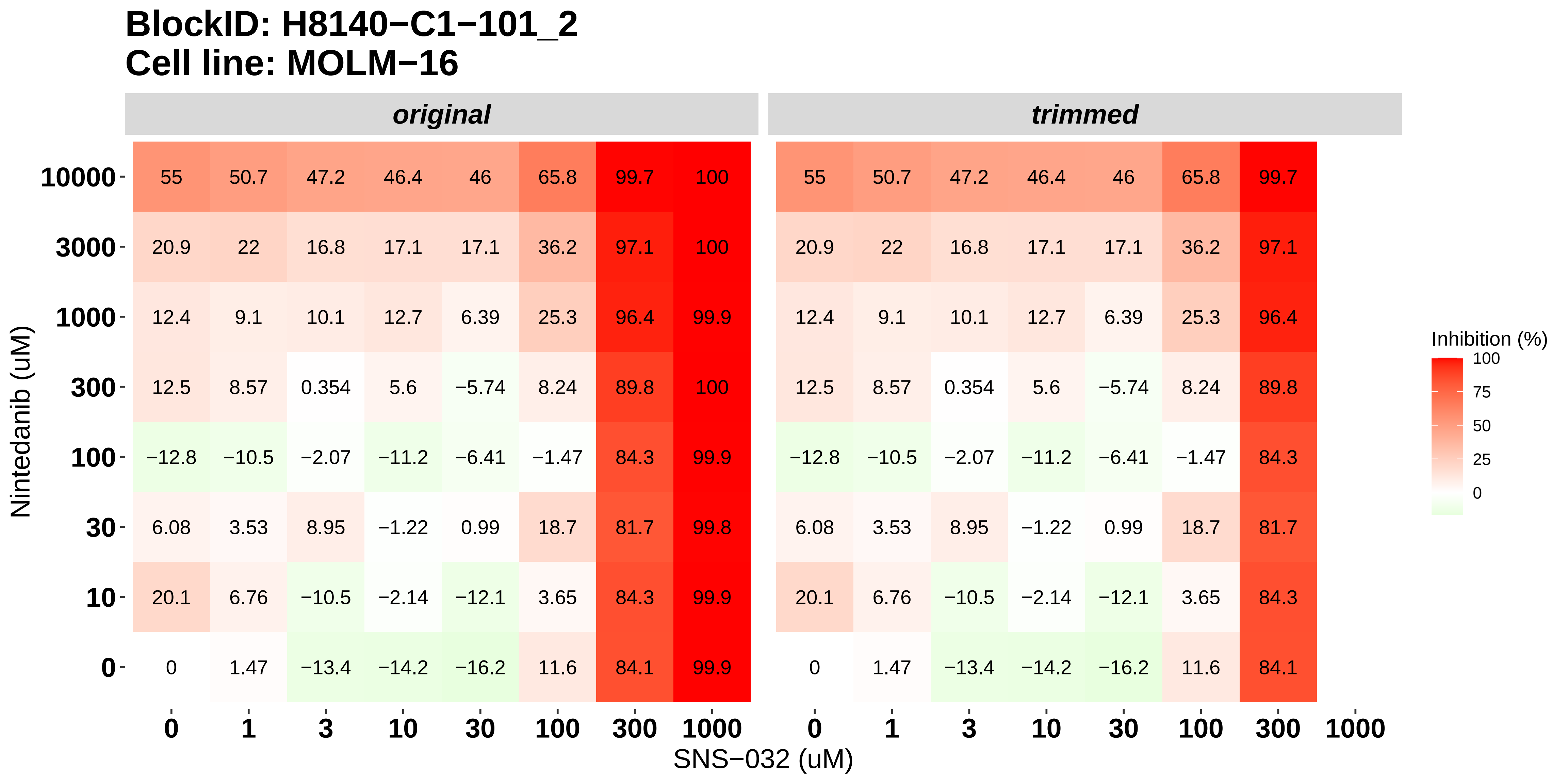
BlockID: H8140−C1−101_2
Cell line: MOLM−16
original
trimmed
Nintedanib (uM)
SNS−032 (uM)
Inhibition (%)
100
75
50
25
0
10000 3000 1000 300 100 30 10 0
0 1 3 10 30 100 300 1000
55 50.7 47.2 46.4 46 65.8 99.7 100
20.9 22 16.8 17.1 17.1 36.2 97.1 100
12.4 9.1 10.1 12.7 6.39 25.3 96.4 99.9
12.5 8.57 0.354 5.6 −5.74 8.24 89.8 100
−12.8 −10.5 −2.07 −11.2 −6.41 −1.47 84.3 99.9
6.08 3.53 8.95 −1.22 0.99 18.7 81.7 99.8
20.1 6.76 −10.5 −2.14 −12.1 3.65 84.3 99.9
0 1.47 −13.4 −14.2 −16.2 11.6 84.1 99.9
55 50.7 47.2 46.4 46 65.8 99.7
20.9 22 16.8 17.1 17.1 36.2 97.1
12.4 9.1 10.1 12.7 6.39 25.3 96.4
12.5 8.57 0.354 5.6 −5.74 8.24 89.8
−12.8 −10.5 −2.07 −11.2 −6.41 −1.47 84.3
6.08 3.53 8.95 −1.22 0.99 18.7 81.7
20.1 6.76 −10.5 −2.14 −12.1 3.65 84.3
0 1.47 −13.4 −14.2 −16.2 11.6 84.1

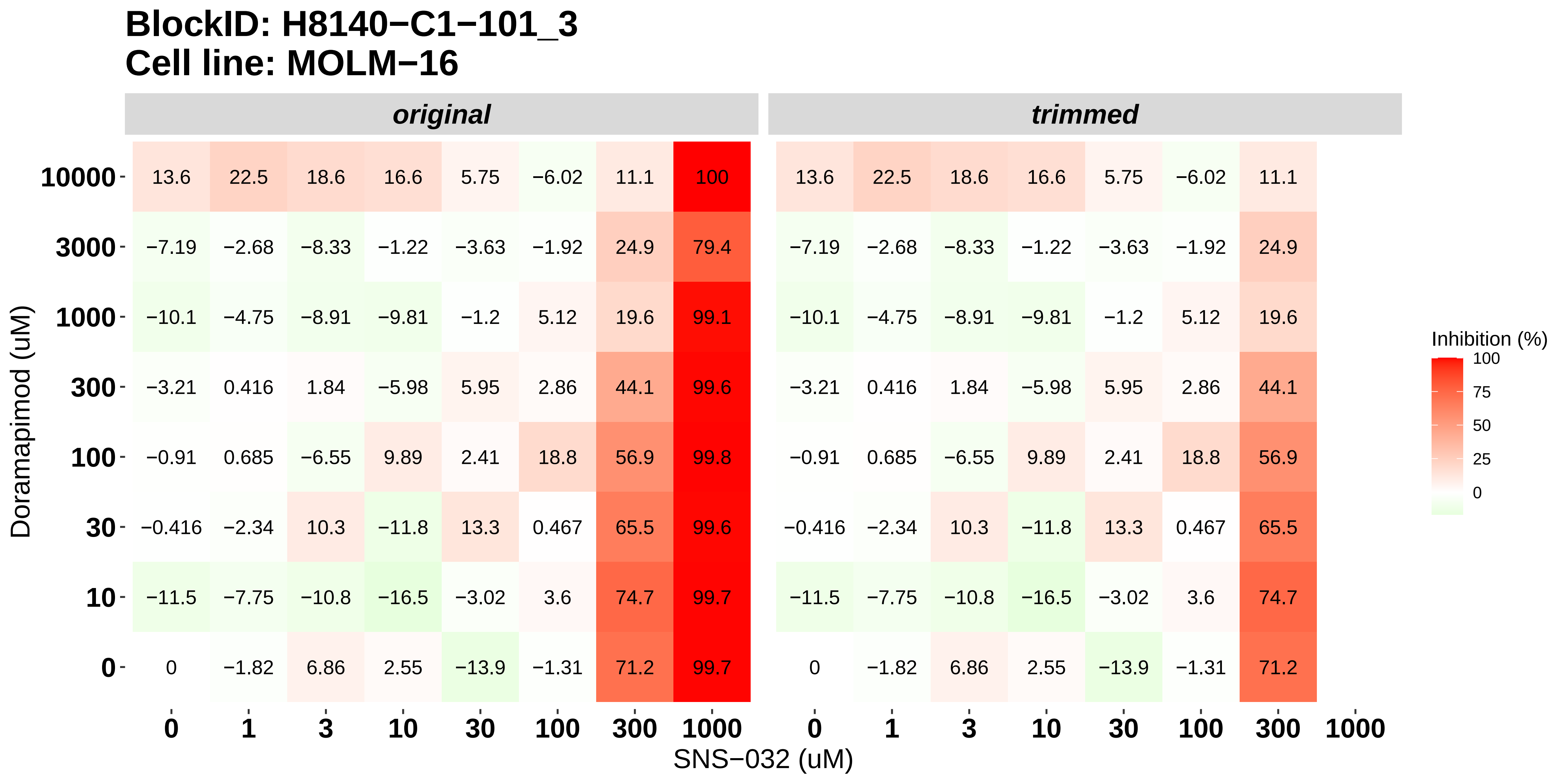
BlockID: H8140−C1−101_3
Cell line: MOLM−16
original
trimmed
Doramapimod (uM)
SNS−032 (uM)
Inhibition (%)
100
75
50
25
0
10000 13.6 22.5 18.6 16.6 5.75 −6.02 11.1 100
3000 −7.19 −2.68 −8.33 −1.22 −3.63 −1.92 24.9 79.4
1000 −10.1 −4.75 −8.91 −9.81 −1.2 5.12 19.6 99.1
300 −3.21 0.416 1.84 −5.98 5.95 2.86 44.1 99.6
100 −0.91 0.685 −6.55 9.89 2.41 18.8 56.9 99.8
30 −0.416 −2.34 10.3 −11.8 13.3 0.467 65.5 99.6
10 −11.5 −7.75 −10.8 −16.5 −3.02 3.6 74.7 99.7
0 0 −1.82 6.86 2.55 −13.9 −1.31 71.2 99.7
0 1 3 10 30 100 300 1000
13.6 22.5 18.6 16.6 5.75 −6.02 11.1
−7.19 −2.68 −8.33 −1.22 −3.63 −1.92 24.9
−10.1 −4.75 −8.91 −9.81 −1.2 5.12 19.6
−3.21 0.416 1.84 −5.98 5.95 2.86 44.1
−0.91 0.685 −6.55 9.89 2.41 18.8 56.9
−0.416 −2.34 10.3 −11.8 13.3 0.467 65.5
−11.5 −7.75 −10.8 −16.5 −3.02 3.6 74.7
0 −1.82 6.86 2.55 −13.9 −1.31 71.2
0 1 3 10 30 100 300 1000

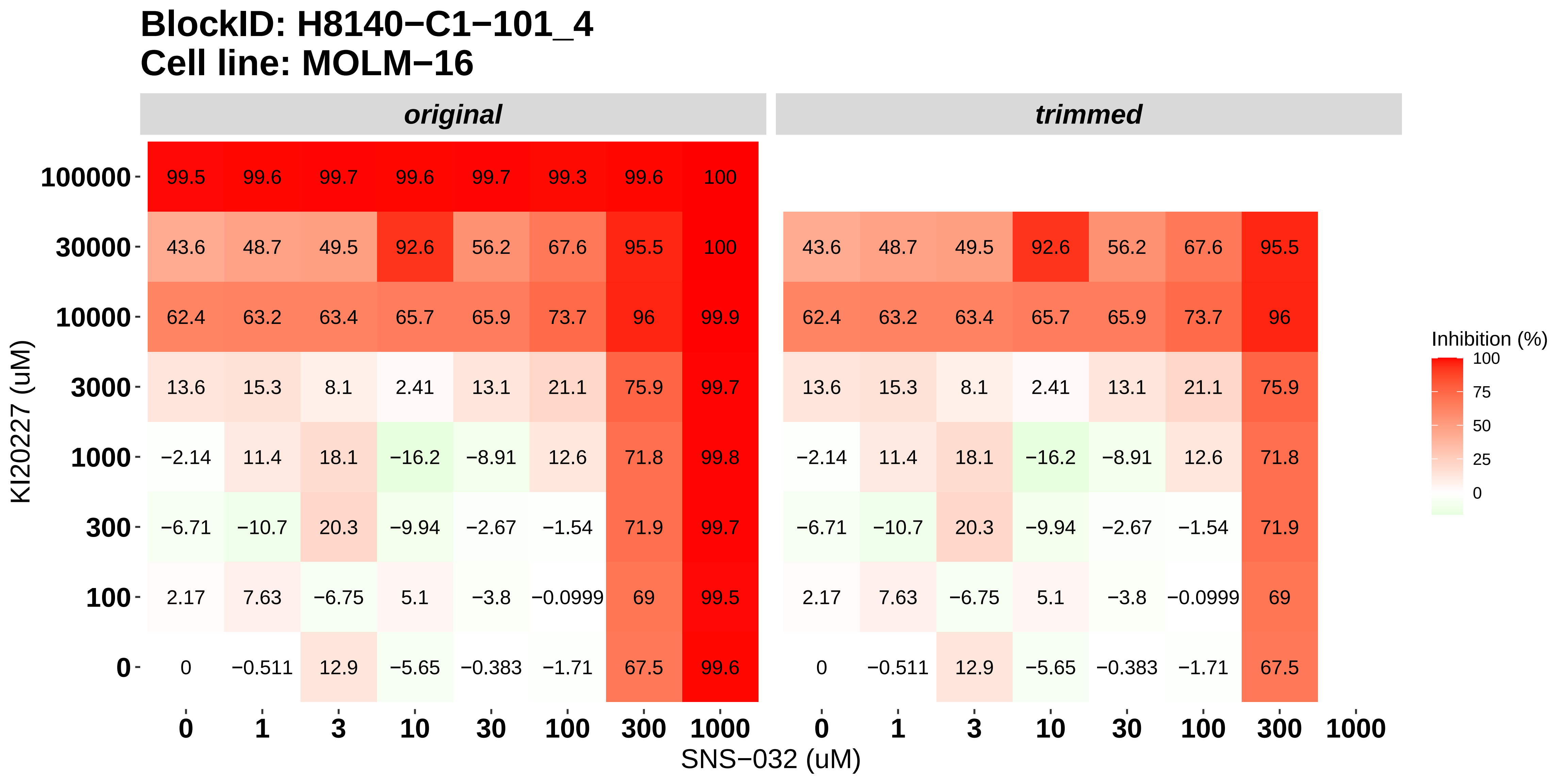

BlockID: H8140−C1−101_4
Cell line: MOLM−16
original
trimmed
100000
30000
10000
3000
1000
300
100
0
KI20227 (uM)
99.5 99.6 99.7 99.6 99.7 99.3 99.6 100
43.6 48.7 49.5 92.6 56.2 67.6 95.5 100
62.4 63.2 63.4 65.7 65.9 73.7 96 99.9
13.6 15.3 8.1 2.41 13.1 21.1 75.9 99.7
−2.14 11.4 18.1 −16.2 −8.91 12.6 71.8 99.8
−6.71 −10.7 20.3 −9.94 −2.67 −1.54 71.9 99.7
2.17 7.63 −6.75 5.1 −3.8 −0.0999 69 99.5
0 −0.511 12.9 −5.65 −0.383 −1.71 67.5 99.6
43.6 48.7 49.5 92.6 56.2 67.6 95.5
62.4 63.2 63.4 65.7 65.9 73.7 96
13.6 15.3 8.1 2.41 13.1 21.1 75.9
−2.14 11.4 18.1 −16.2 −8.91 12.6 71.8
−6.71 −10.7 20.3 −9.94 −2.67 −1.54 71.9
2.17 7.63 −6.75 5.1 −3.8 −0.0999 69
0 −0.511 12.9 −5.65 −0.383 −1.71 67.5
0 1 3 10 30 100 300 1000
0 1 3 10 30 100 300 1000
SNS−032 (uM)
Inhibition (%)
100
75
50
25
0

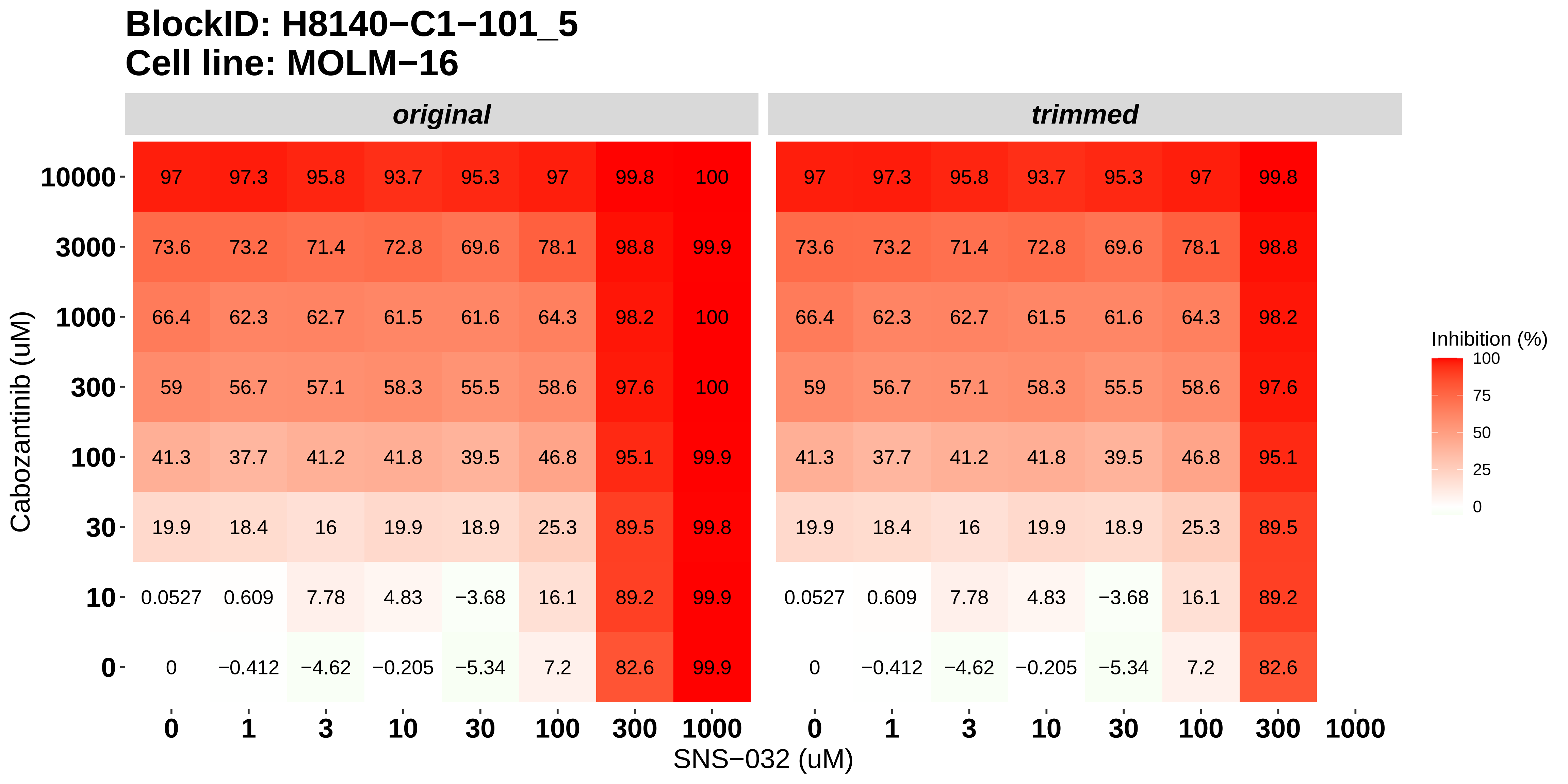
BlockID: H8140−C1−101_5
Cell line: MOLM−16
original
trimmed
Cabozantinib (uM)
SNS−032 (uM)
Inhibition (%)
100
75
50
25
0
10000 97 97.3 95.8 93.7 95.3 97 99.8 100
3000 73.6 73.2 71.4 72.8 69.6 78.1 98.8 99.9
1000 66.4 62.3 62.7 61.5 61.6 64.3 98.2 100
300 59 56.7 57.1 58.3 55.5 58.6 97.6 100
100 41.3 37.7 41.2 41.8 39.5 46.8 95.1 99.9
30 19.9 18.4 16 19.9 18.9 25.3 89.5 99.8
10 0.0527 0.609 7.78 4.83 −3.68 16.1 89.2 99.9
0 0 −0.412 −4.62 −0.205 −5.34 7.2 82.6 99.9
0 1 3 10 30 100 300 1000
10000 97 97.3 95.8 93.7 95.3 97 99.8
3000 73.6 73.2 71.4 72.8 69.6 78.1 98.8
1000 66.4 62.3 62.7 61.5 61.6 64.3 98.2
300 59 56.7 57.1 58.3 55.5 58.6 97.6
100 41.3 37.7 41.2 41.8 39.5 46.8 95.1
30 19.9 18.4 16 19.9 18.9 25.3 89.5
10 0.0527 0.609 7.78 4.83 −3.68 16.1 89.2
0 0 −0.412 −4.62 −0.205 −5.34 7.2 82.6
0 1 3 10 30 100 300 1000

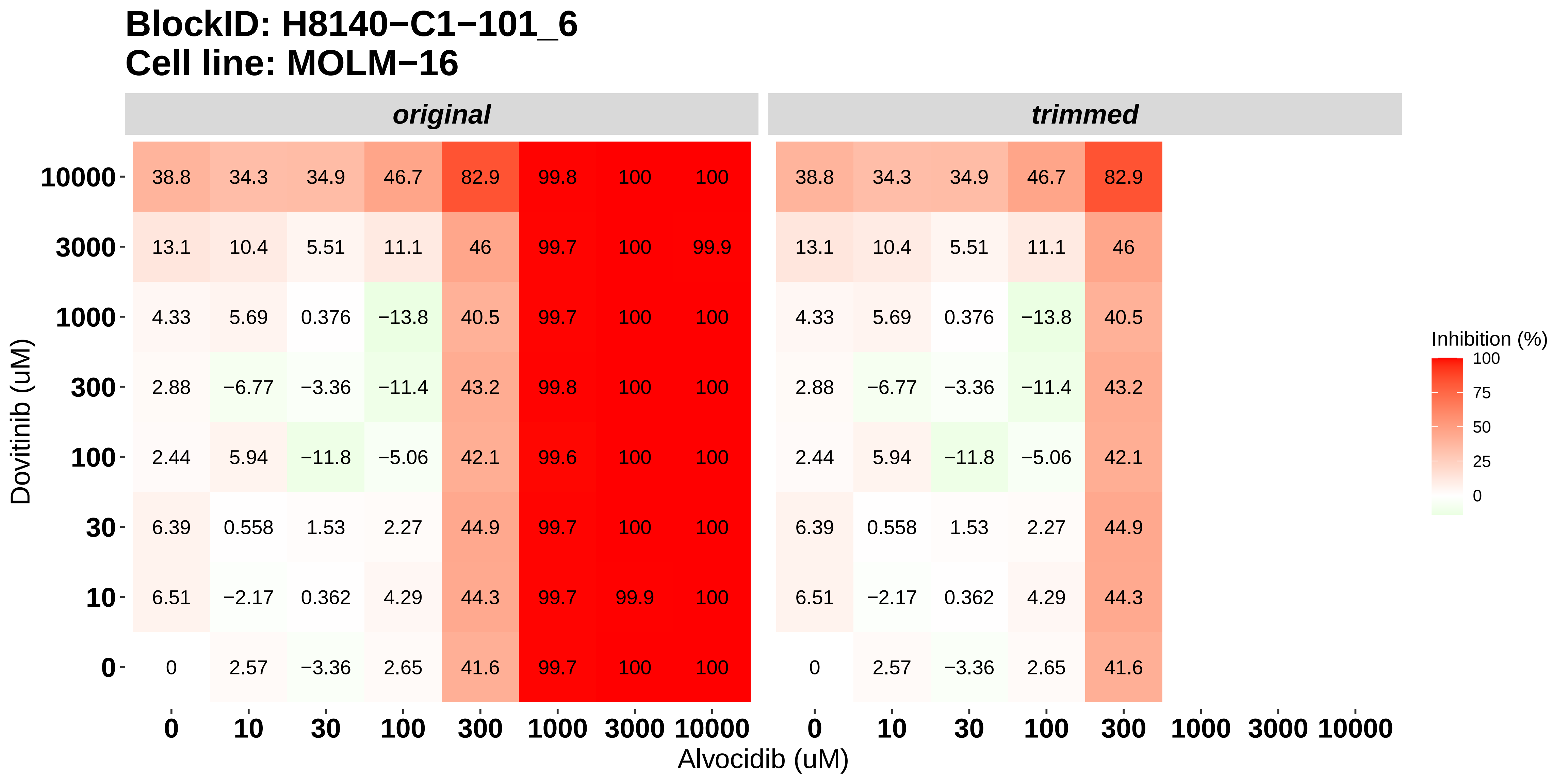

BlockID: H8140−C1−101_6
Cell line: MOLM−16
original
trimmed
Dovitinib (uM)
Alvocidib (uM)
Inhibition (%)
100
75
50
25
0
10000
3000
1000
300
100
30
10
0
38.8 34.3 34.9 46.7 82.9 99.8 100 100
13.1 10.4 5.51 11.1 46 99.7 100 99.9
4.33 5.69 0.376 −13.8 40.5 99.7 100 100
2.88 −6.77 −3.36 −11.4 43.2 99.8 100 100
2.44 5.94 −11.8 −5.06 42.1 99.6 100 100
6.39 0.558 1.53 2.27 44.9 99.7 100 100
6.51 −2.17 0.362 4.29 44.3 99.7 99.9 100
0 2.57 −3.36 2.65 41.6 99.7 100 100
38.8 34.3 34.9 46.7 82.9
13.1 10.4 5.51 11.1 46
4.33 5.69 0.376 −13.8 40.5
2.88 −6.77 −3.36 −11.4 43.2
2.44 5.94 −11.8 −5.06 42.1
6.39 0.558 1.53 2.27 44.9
6.51 −2.17 0.362 4.29 44.3
0 2.57 −3.36 2.65 41.6
0 10 30 100 300 1000 3000 10000
0 10 30 100 300 1000 3000 10000

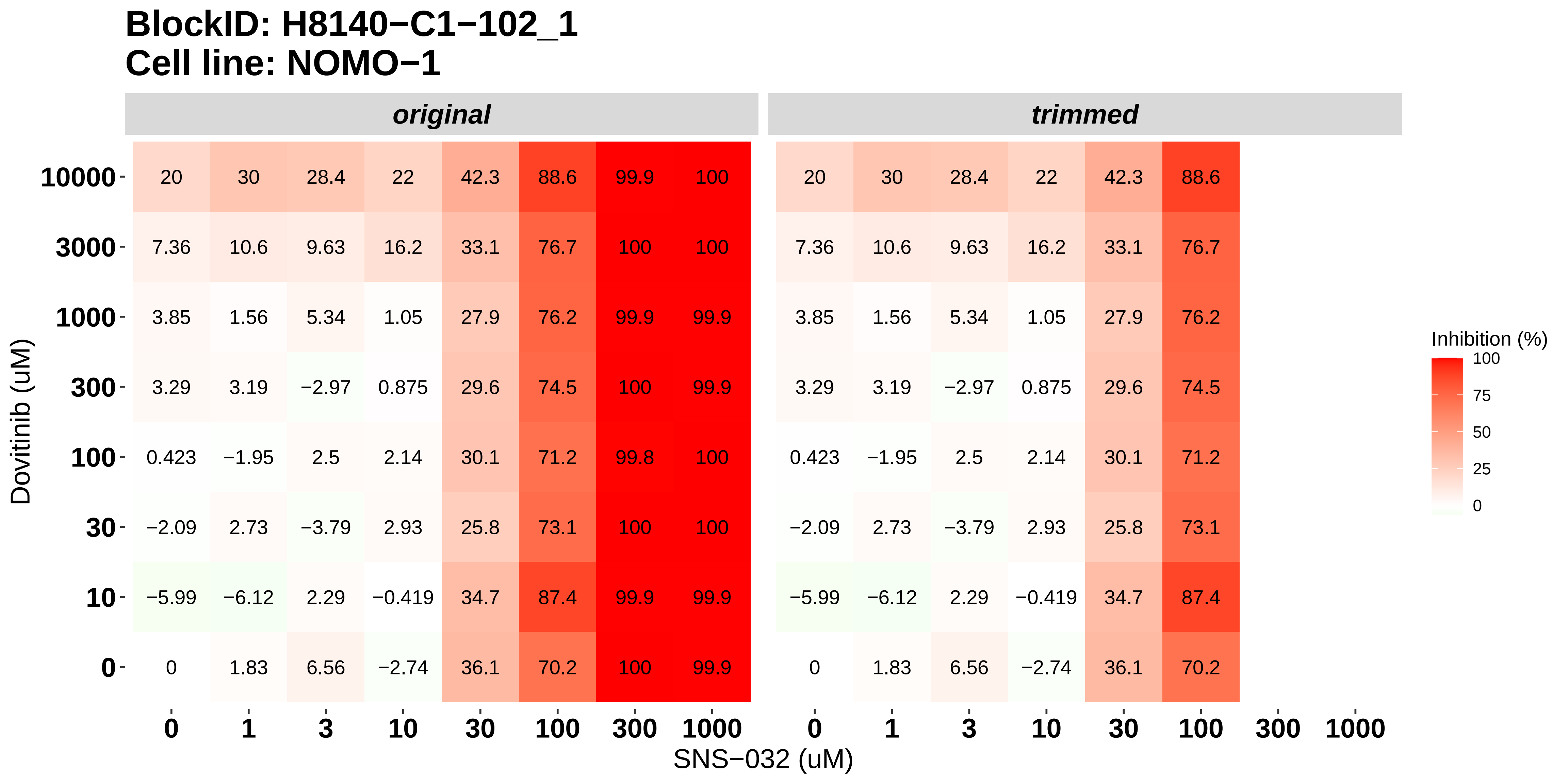
BlockID: H8140−C1−102_1
Cell line: NOMO−1
original
trimmed
Dovitinib (uM)
10000
3000
1000
300
100
30
10
0
SNS−032 (uM)
0 1 3 10 30 100 300 1000
0 1 3 10 30 100 300 1000
Inhibition (%)
100
75
50
25
0
20 30 28.4 22 42.3 88.6 99.9 100
7.36 10.6 9.63 16.2 33.1 76.7 100 100
3.85 1.56 5.34 1.05 27.9 76.2 99.9 99.9
3.29 3.19 −2.97 0.875 29.6 74.5 100 99.9
0.423 −1.95 2.5 2.14 30.1 71.2 99.8 100
−2.09 2.73 −3.79 2.93 25.8 73.1 100 100
−5.99 −6.12 2.29 −0.419 34.7 87.4 99.9 99.9
0 1.83 6.56 −2.74 36.1 70.2 100 99.9
20 30 28.4 22 42.3 88.6
7.36 10.6 9.63 16.2 33.1 76.7
3.85 1.56 5.34 1.05 27.9 76.2
3.29 3.19 −2.97 0.875 29.6 74.5
0.423 −1.95 2.5 2.14 30.1 71.2
−2.09 2.73 −3.79 2.93 25.8 73.1
−5.99 −6.12 2.29 −0.419 34.7 87.4
0 1.83 6.56 −2.74 36.1 70.2

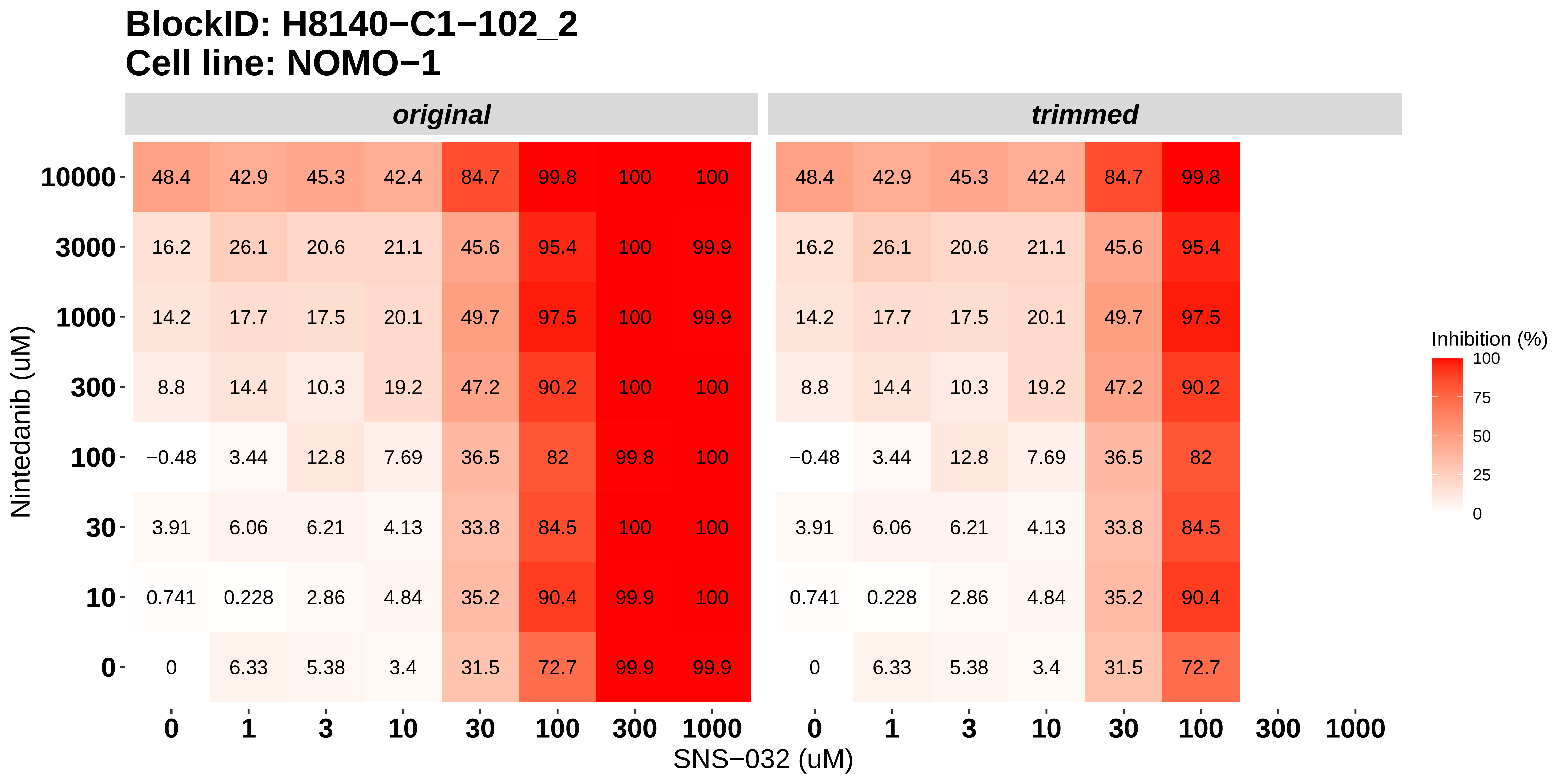
BlockID: H8140−C1−102_2
Cell line: NOMO−1
original
trimmed
Nintedanib (uM)
SNS−032 (uM)
Inhibition (%)
100
75
50
25
0
10000
3000
1000
300
100
30
10
0
0
1
3
10
30
100
300
1000
48.4 42.9 45.3 42.4 84.7 99.8 100 100
16.2 26.1 20.6 21.1 45.6 95.4 100 99.9
14.2 17.7 17.5 20.1 49.7 97.5 100 99.9
8.8 14.4 10.3 19.2 47.2 90.2 100 100
−0.48 3.44 12.8 7.69 36.5 82 99.8 100
3.91 6.06 6.21 4.13 33.8 84.5 100 100
0.741 0.228 2.86 4.84 35.2 90.4 99.9 100
0 6.33 5.38 3.4 31.5 72.7 99.9 99.9
48.4 42.9 45.3 42.4 84.7 99.8
16.2 26.1 20.6 21.1 45.6 95.4
14.2 17.7 17.5 20.1 49.7 97.5
8.8 14.4 10.3 19.2 47.2 90.2
−0.48 3.44 12.8 7.69 36.5 82
3.91 6.06 6.21 4.13 33.8 84.5
0.741 0.228 2.86 4.84 35.2 90.4
0 6.33 5.38 3.4 31.5 72.7

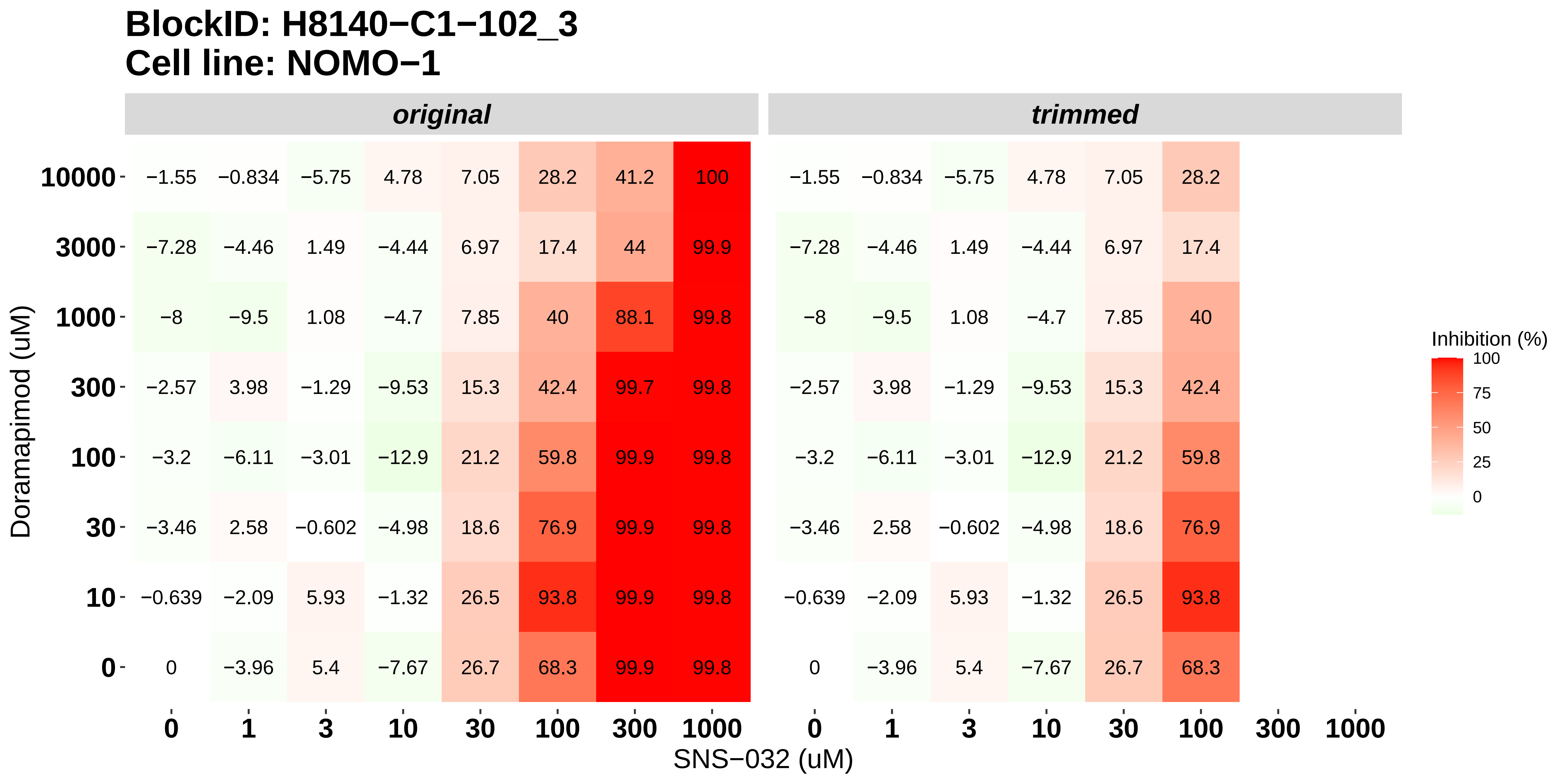

BlockID: H8140−C1−102_3
Cell line: NOMO−1
original
trimmed
Doramapimod (uM)
SNS−032 (uM)
Inhibition (%)

BlockID: H8140−C1−102_4
Cell line: NOMO−1

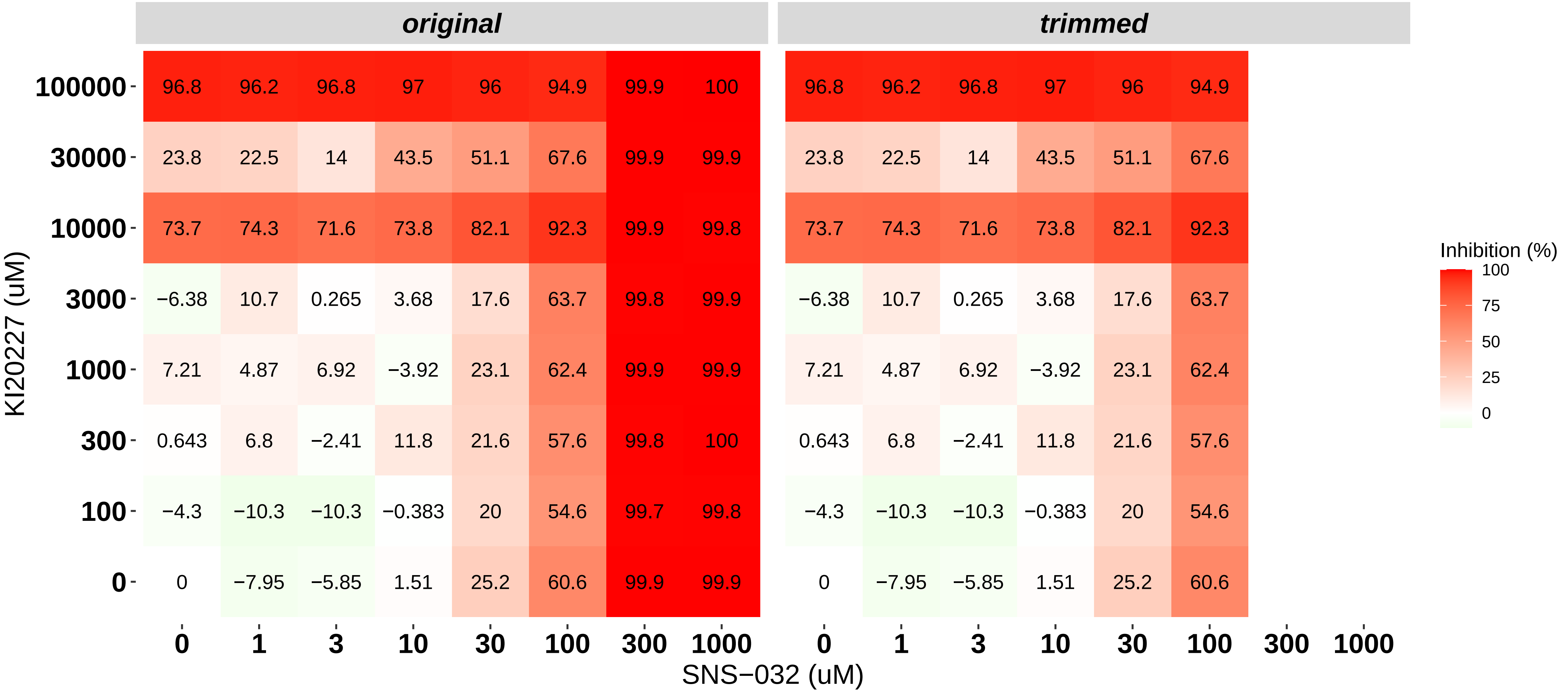

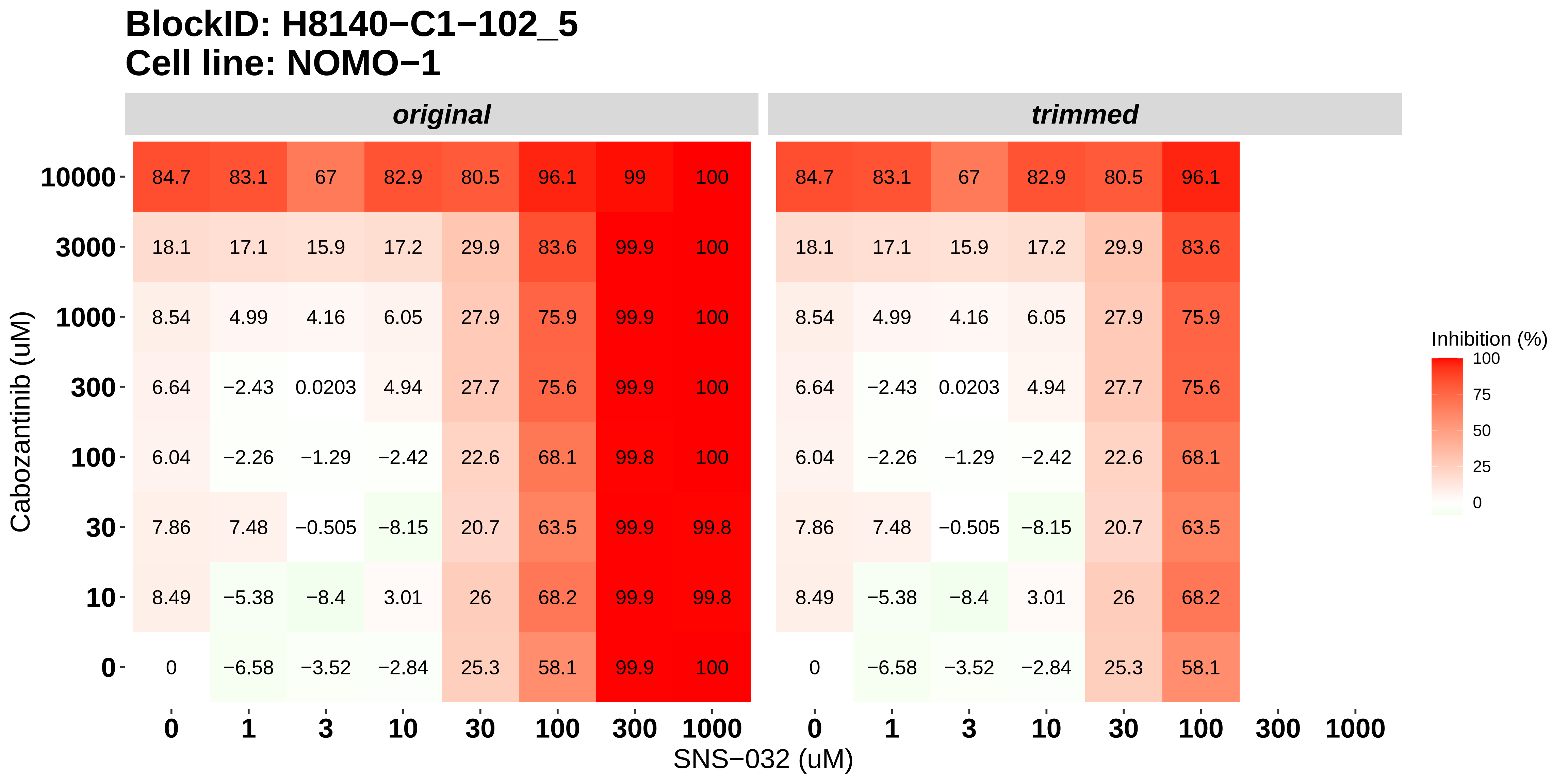
BlockID: H8140−C1−102_5
Cell line: NOMO−1
original
trimmed
Cabozantinib (uM)
10000
3000
1000
300
100
30
10
0
84.7 83.1 67 82.9 80.5 96.1 99 100
18.1 17.1 15.9 17.2 29.9 83.6 99.9 100
8.54 4.99 4.16 6.05 27.9 75.9 99.9 100
6.64 −2.43 0.0203 4.94 27.7 75.6 99.9 100
6.04 −2.26 −1.29 −2.42 22.6 68.1 99.8 100
7.86 7.48 −0.505 −8.15 20.7 63.5 99.9 99.8
8.49 −5.38 −8.4 3.01 26 68.2 99.9 99.8
0 −6.58 −3.52 −2.84 25.3 58.1 99.9 100
84.7 83.1 67 82.9 80.5 96.1
18.1 17.1 15.9 17.2 29.9 83.6
8.54 4.99 4.16 6.05 27.9 75.9
6.64 −2.43 0.0203 4.94 27.7 75.6
6.04 −2.26 −1.29 −2.42 22.6 68.1
7.86 7.48 −0.505 −8.15 20.7 63.5
8.49 −5.38 −8.4 3.01 26 68.2
0 −6.58 −3.52 −2.84 25.3 58.1
0 1 3 10 30 100 300 1000
0 1 3 10 30 100 300 1000
SNS−032 (uM)
Inhibition (%)
100
75
50
25
0

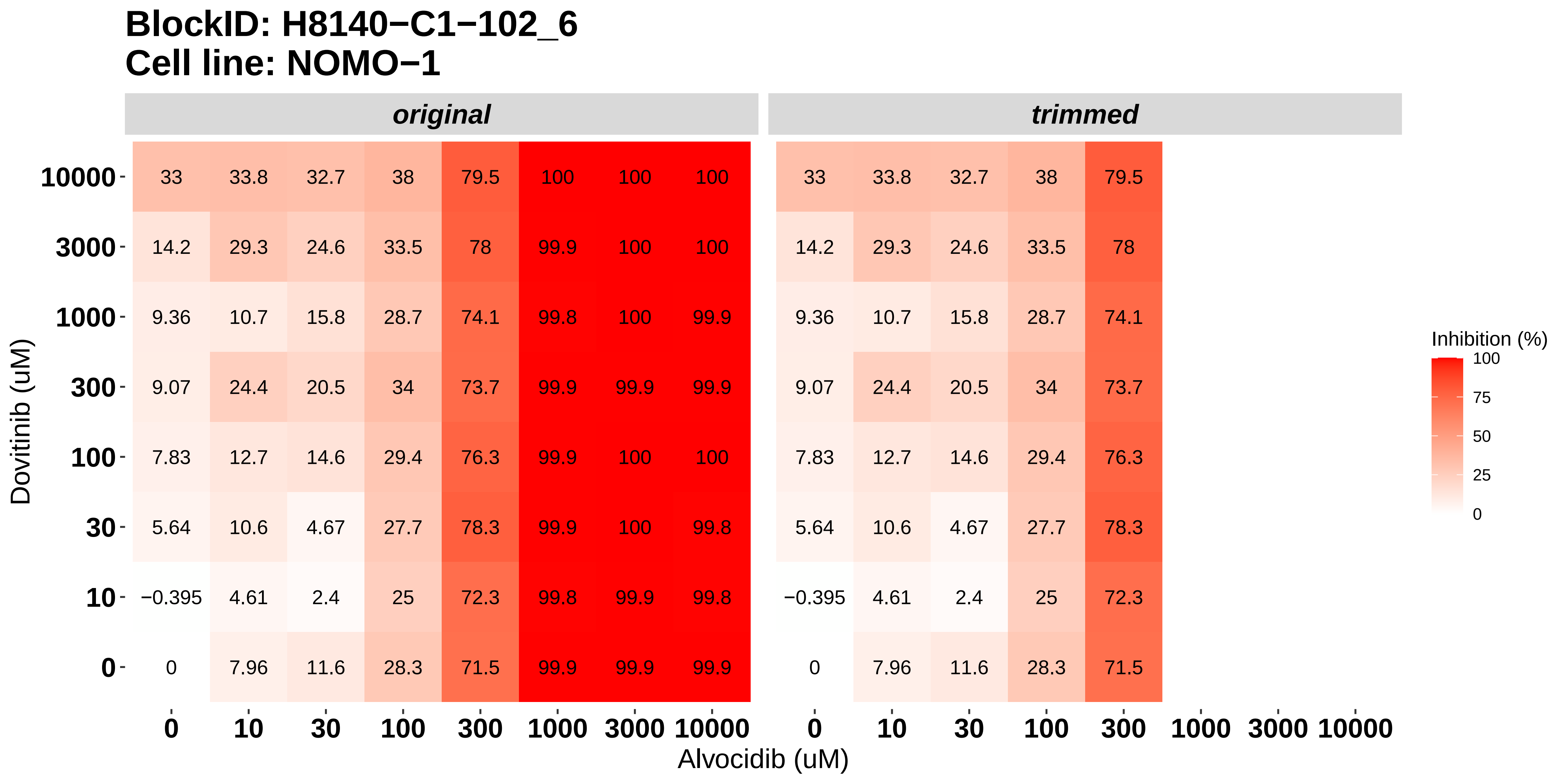
BlockID: H8140−C1−102_6
Cell line: NOMO−1
original
trimmed
Dovitinib (uM)
Alvocidib (uM)
Inhibition (%)
100
75
50
25
0
10000
3000
1000
300
100
30
10
0
33 33.8 32.7 38 79.5 100 100 100
14.2 29.3 24.6 33.5 78 99.9 100 100
9.36 10.7 15.8 28.7 74.1 99.8 100 99.9
9.07 24.4 20.5 34 73.7 99.9 99.9 99.9
7.83 12.7 14.6 29.4 76.3 99.9 100 100
5.64 10.6 4.67 27.7 78.3 99.9 100 99.8
−0.395 4.61 2.4 25 72.3 99.8 99.9 99.8
0 7.96 11.6 28.3 71.5 99.9 99.9 99.9
33 33.8 32.7 38 79.5
14.2 29.3 24.6 33.5 78
9.36 10.7 15.8 28.7 74.1
9.07 24.4 20.5 34 73.7
7.83 12.7 14.6 29.4 76.3
5.64 10.6 4.67 27.7 78.3
−0.395 4.61 2.4 25 72.3
0 7.96 11.6 28.3 71.5
0 10 30 100 300 1000 3000 10000
0 10 30 100 300 1000 3000 10000

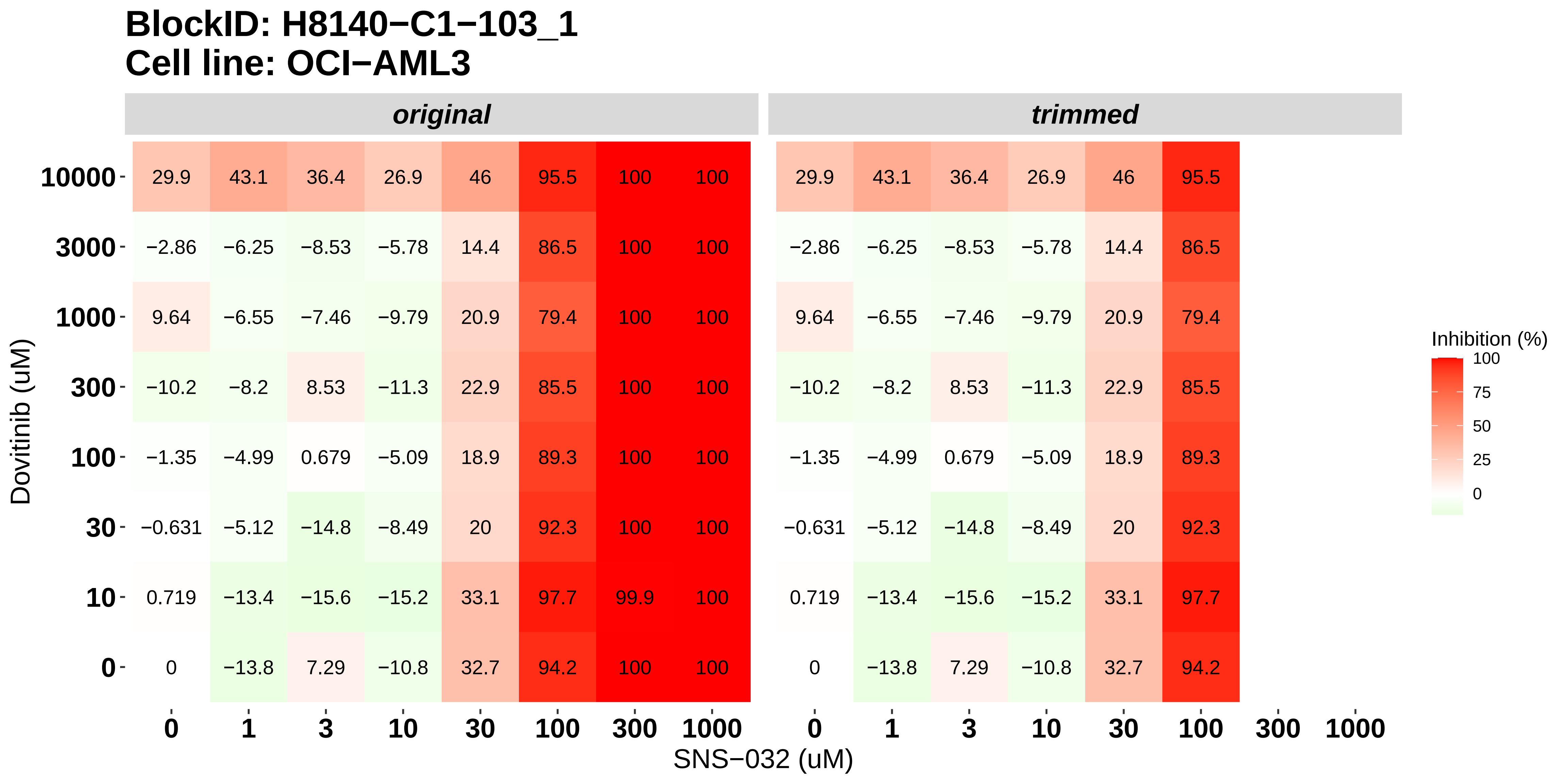
BlockID: H8140−C1−103_1
Cell line: OCI−AML3
original
trimmed
Dovitinib (uM)
10000
3000
1000
300
100
30
10
0
29.9 43.1 36.4 26.9 46 95.5 100 100
−2.86 −6.25 −8.53 −5.78 14.4 86.5 100 100
9.64 −6.55 −7.46 −9.79 20.9 79.4 100 100
−10.2 −8.2 8.53 −11.3 22.9 85.5 100 100
−1.35 −4.99 0.679 −5.09 18.9 89.3 100 100
−0.631 −5.12 −14.8 −8.49 20 92.3 100 100
0.719 −13.4 −15.6 −15.2 33.1 97.7 99.9 100
0 −13.8 7.29 −10.8 32.7 94.2 100 100
29.9 43.1 36.4 26.9 46 95.5
−2.86 −6.25 −8.53 −5.78 14.4 86.5
9.64 −6.55 −7.46 −9.79 20.9 79.4
−10.2 −8.2 8.53 −11.3 22.9 85.5
−1.35 −4.99 0.679 −5.09 18.9 89.3
−0.631 −5.12 −14.8 −8.49 20 92.3
0.719 −13.4 −15.6 −15.2 33.1 97.7
0 −13.8 7.29 −10.8 32.7 94.2
0 1 3 10 30 100 300 1000
0 1 3 10 30 100 300 1000
SNS−032 (uM)
Inhibition (%)
100
75
50
25
0

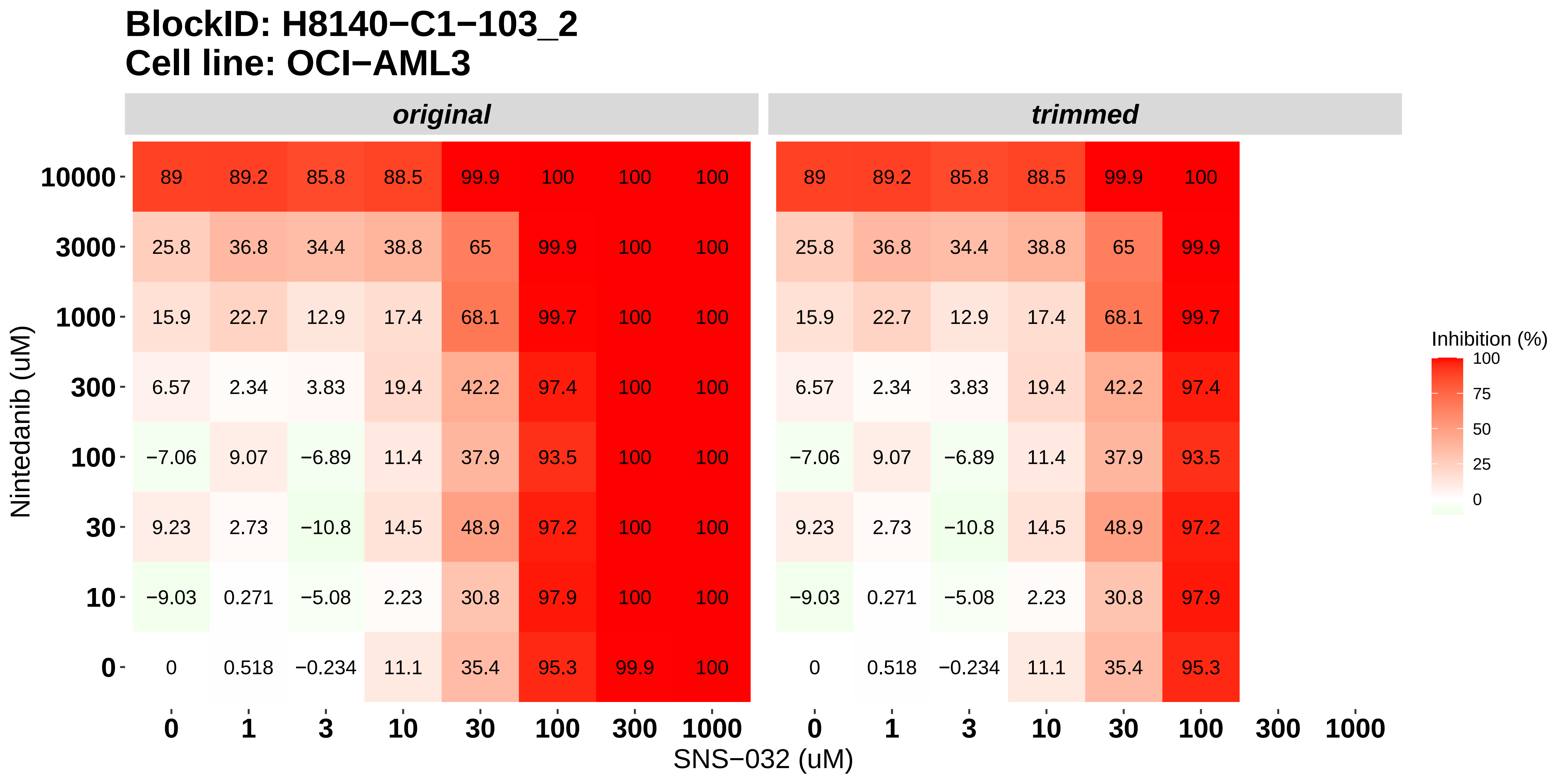
BlockID: H8140−C1−103_2
Cell line: OCI−AML3
original
trimmed
Nintedanib (uM)
SNS−032 (uM)
Inhibition (%)

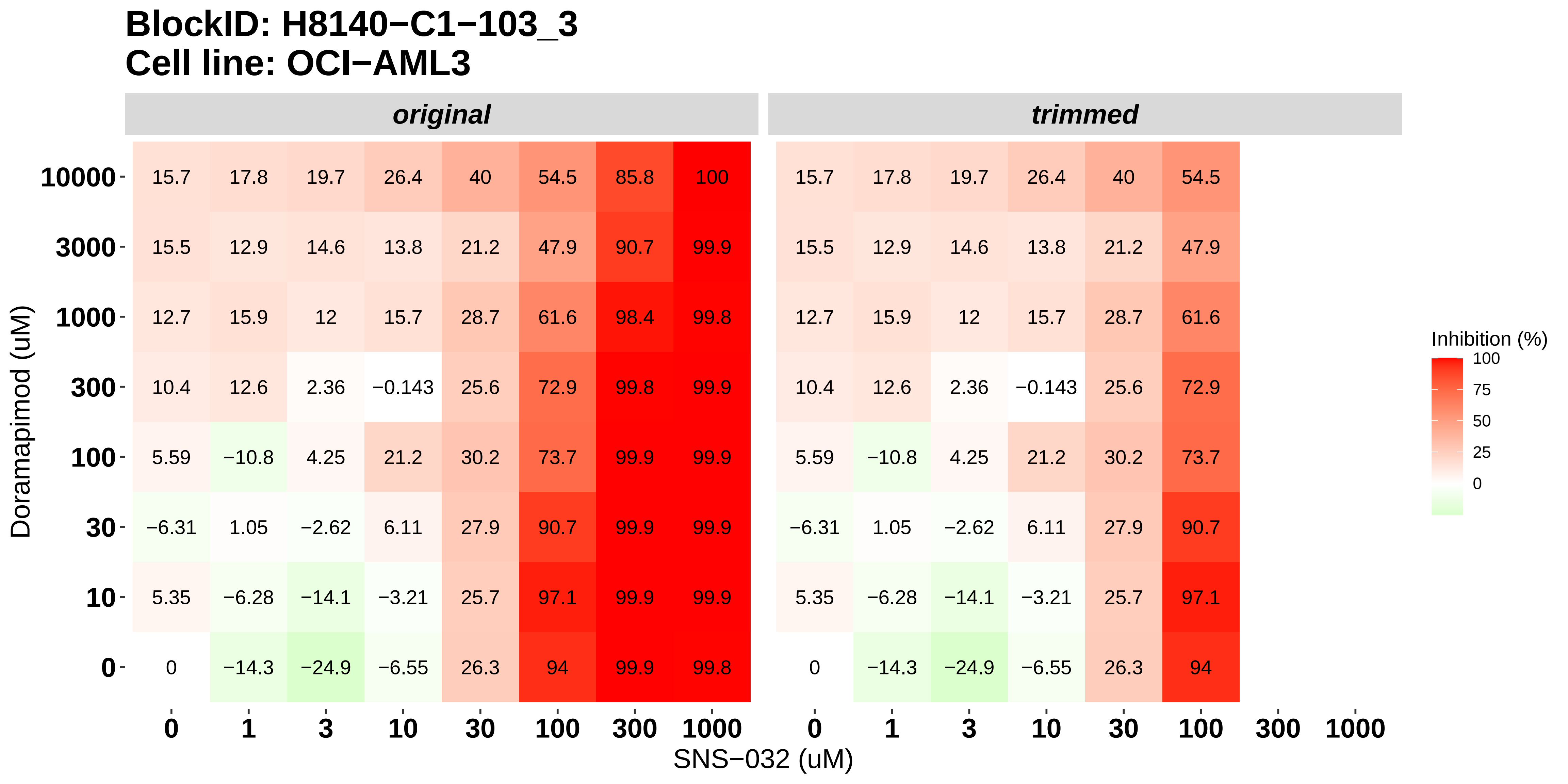
BlockID: H8140−C1−103_3
Cell line: OCI−AML3
original
trimmed
Doramapimod (uM)
SNS−032 (uM)
Inhibition (%)
100
75
50
25
0
10000 15.7 17.8 19.7 26.4 40 54.5 85.8 100
3000 15.5 12.9 14.6 13.8 21.2 47.9 90.7 99.9
1000 12.7 15.9 12 15.7 28.7 61.6 98.4 99.8
300 10.4 12.6 2.36 −0.143 25.6 72.9 99.8 99.9
100 5.59 −10.8 4.25 21.2 30.2 73.7 99.9 99.9
30 −6.31 1.05 −2.62 6.11 27.9 90.7 99.9 99.9
10 5.35 −6.28 −14.1 −3.21 25.7 97.1 99.9 99.9
0 0 −14.3 −24.9 −6.55 26.3 94 99.9 99.8
0 1 3 10 30 100 300 1000
10000 15.7 17.8 19.7 26.4 40 54.5
3000 15.5 12.9 14.6 13.8 21.2 47.9
1000 12.7 15.9 12 15.7 28.7 61.6
300 10.4 12.6 2.36 −0.143 25.6 72.9
100 5.59 −10.8 4.25 21.2 30.2 73.7
30 −6.31 1.05 −2.62 6.11 27.9 90.7
10 5.35 −6.28 −14.1 −3.21 25.7 97.1
0 0 −14.3 −24.9 −6.55 26.3 94
0 1 3 10 30 100 300 1000

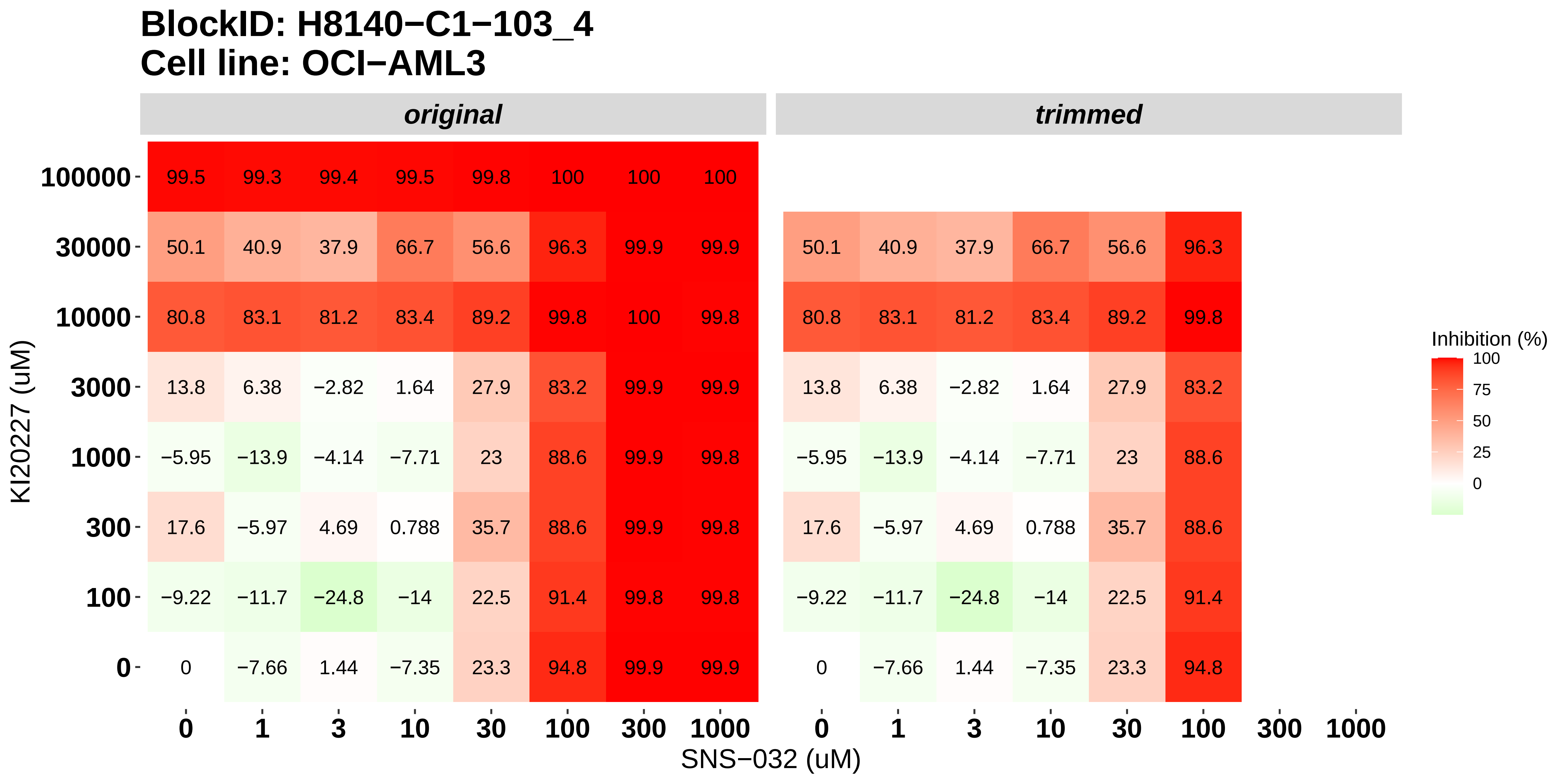

BlockID: H8140−C1−103_4
Cell line: OCI−AML3
original
trimmed
KI20227 (uM)
100000
30000
10000
3000
1000
300
100
0
99.5 99.3 99.4 99.5 99.8 100 100 100
50.1 40.9 37.9 66.7 56.6 96.3 99.9 99.9
80.8 83.1 81.2 83.4 89.2 99.8 100 99.8
13.8 6.38 −2.82 1.64 27.9 83.2 99.9 99.9
−5.95 −13.9 −4.14 −7.71 23 88.6 99.9 99.8
17.6 −5.97 4.69 0.788 35.7 88.6 99.9 99.8
−9.22 −11.7 −24.8 −14 22.5 91.4 99.8 99.8
0 −7.66 1.44 −7.35 23.3 94.8 99.9 99.9
50.1 40.9 37.9 66.7 56.6 96.3
80.8 83.1 81.2 83.4 89.2 99.8
13.8 6.38 −2.82 1.64 27.9 83.2
−5.95 −13.9 −4.14 −7.71 23 88.6
17.6 −5.97 4.69 0.788 35.7 88.6
−9.22 −11.7 −24.8 −14 22.5 91.4
0 −7.66 1.44 −7.35 23.3 94.8
0 1 3 10 30 100 300 1000
0 1 3 10 30 100 300 1000
SNS−032 (uM)
Inhibition (%)
100
75
50
25
0

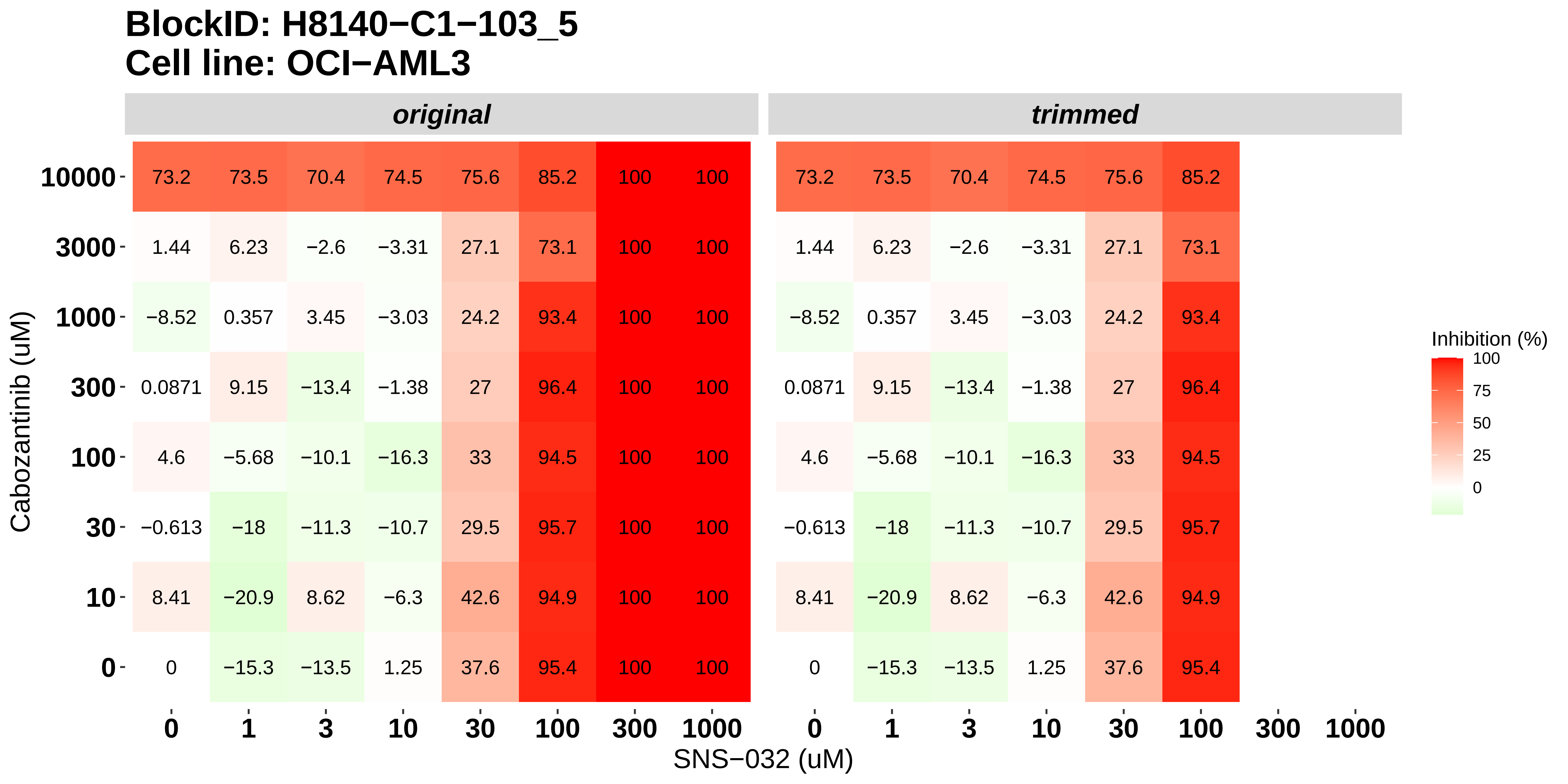
BlockID: H8140−C1−103_5
Cell line: OCI−AML3
original
trimmed
10000
3000
1000
300
100
30
10
0
Cabozantinib (uM)
73.2 73.5 70.4 74.5 75.6 85.2 100 100
1.44 6.23 −2.6 −3.31 27.1 73.1 100 100
−8.52 0.357 3.45 −3.03 24.2 93.4 100 100
0.0871 9.15 −13.4 −1.38 27 96.4 100 100
4.6 −5.68 −10.1 −16.3 33 94.5 100 100
−0.613 −18 −11.3 −10.7 29.5 95.7 100 100
8.41 −20.9 8.62 −6.3 42.6 94.9 100 100
0 −15.3 −13.5 1.25 37.6 95.4 100 100
73.2 73.5 70.4 74.5 75.6 85.2
1.44 6.23 −2.6 −3.31 27.1 73.1
−8.52 0.357 3.45 −3.03 24.2 93.4
0.0871 9.15 −13.4 −1.38 27 96.4
4.6 −5.68 −10.1 −16.3 33 94.5
−0.613 −18 −11.3 −10.7 29.5 95.7
8.41 −20.9 8.62 −6.3 42.6 94.9
0 −15.3 −13.5 1.25 37.6 95.4
0 1 3 10 30 100 300 1000
0 1 3 10 30 100 300 1000
SNS−032 (uM)
Inhibition (%)
100
75
50
25
0

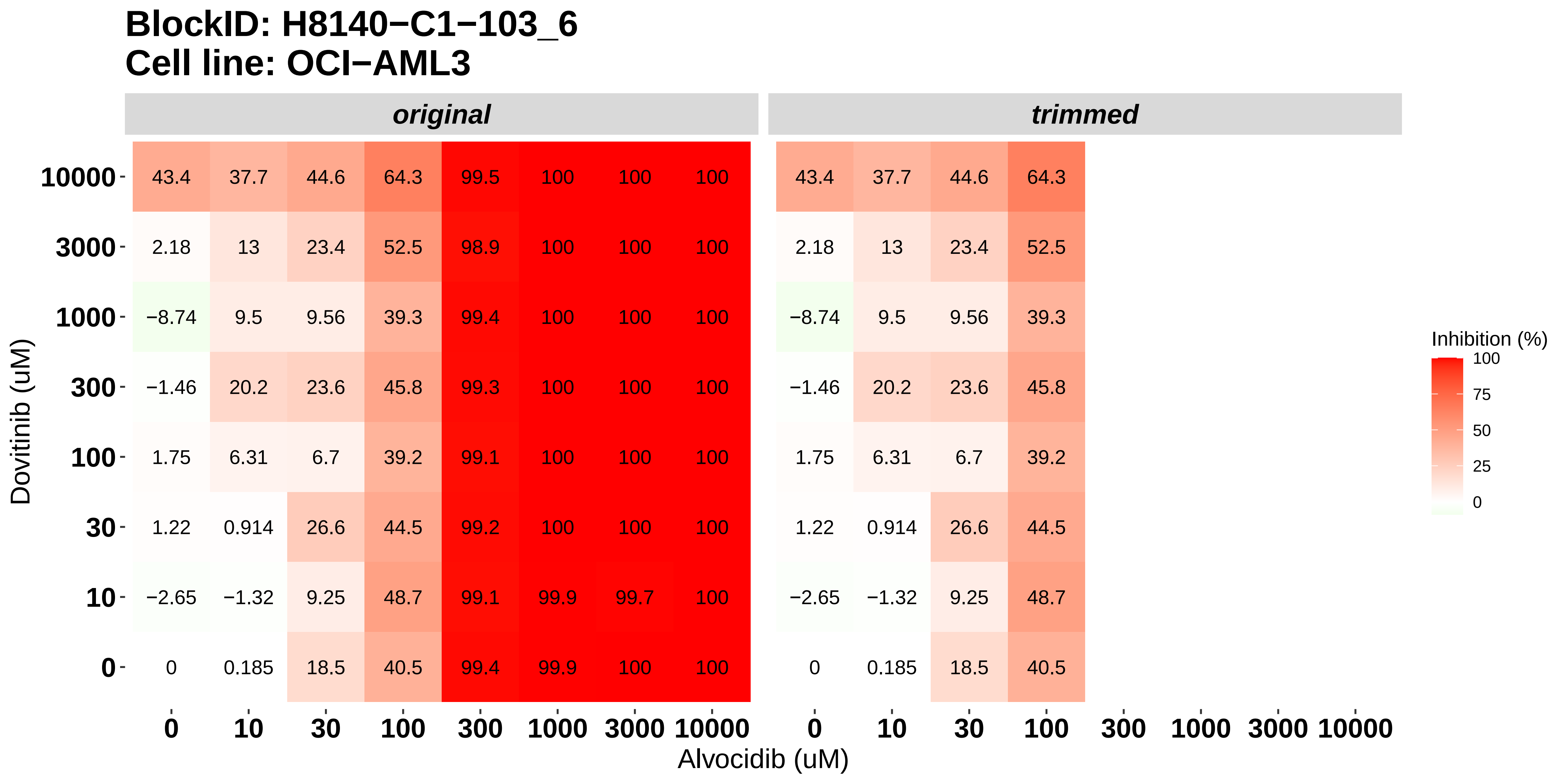
BlockID: H8140−C1−103_6
Cell line: OCI−AML3
original
trimmed
Dovitinib (uM)
Alvocidib (uM)
Inhibition (%)
100
75
50
25
0
10000
3000
1000
300
100
30
10
0
43.4 37.7 44.6 64.3 99.5 100 100 100
2.18 13 23.4 52.5 98.9 100 100 100
−8.74 9.5 9.56 39.3 99.4 100 100 100
−1.46 20.2 23.6 45.8 99.3 100 100 100
1.75 6.31 6.7 39.2 99.1 100 100 100
1.22 0.914 26.6 44.5 99.2 100 100 100
−2.65 −1.32 9.25 48.7 99.1 99.9 99.7 100
0 0.185 18.5 40.5 99.4 99.9 100 100
43.4 37.7 44.6 64.3
2.18 13 23.4 52.5
−8.74 9.5 9.56 39.3
−1.46 20.2 23.6 45.8
1.75 6.31 6.7 39.2
1.22 0.914 26.6 44.5
−2.65 −1.32 9.25 48.7
0 0.185 18.5 40.5
0 10 30 100 300 1000 3000 10000
0 10 30 100 300 1000 3000 10000

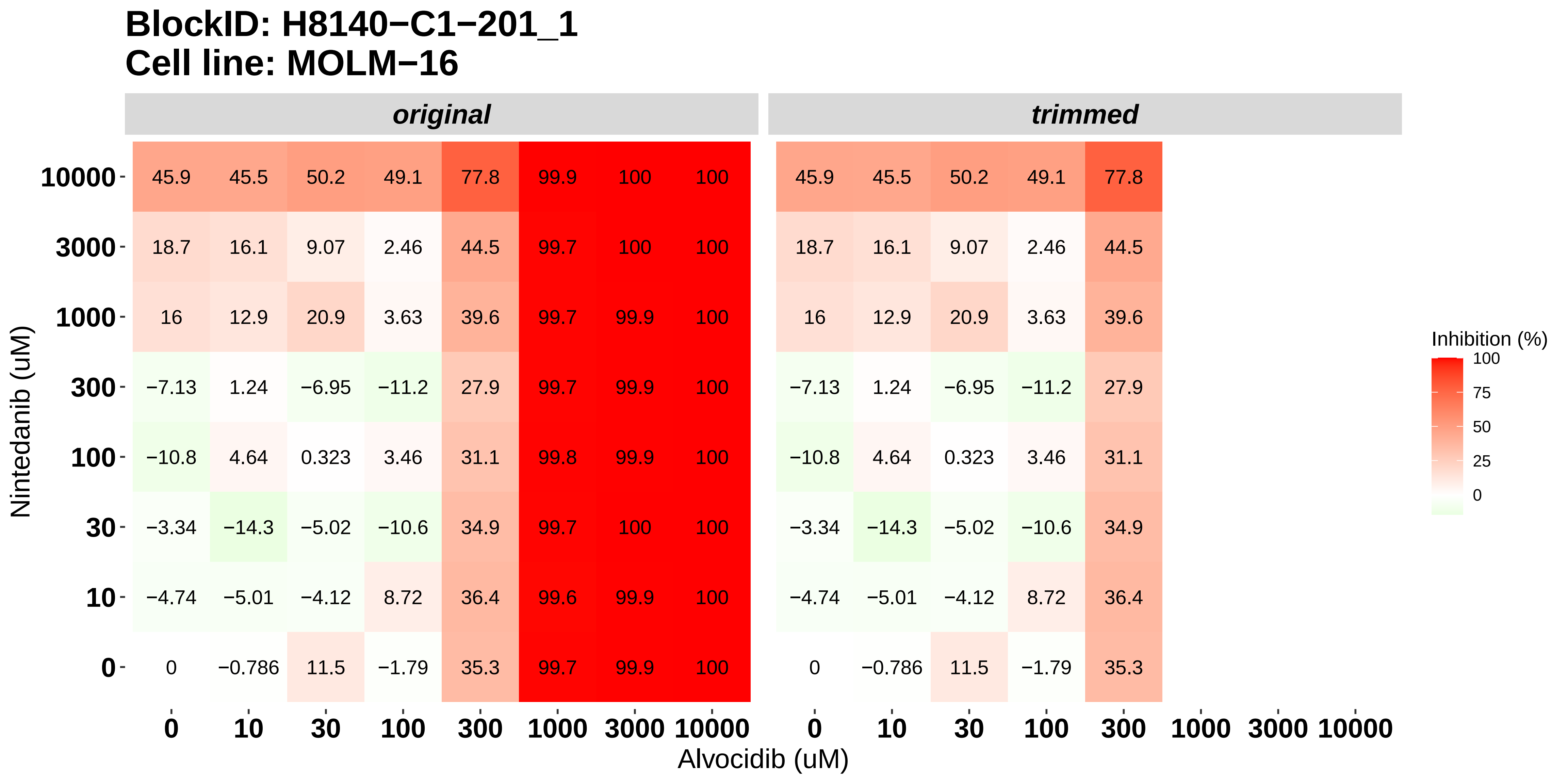
BlockID: H8140−C1−201_1
Cell line: MOLM−16
original
trimmed
Nintedanib (uM)
10000
3000
1000
300
100
30
10
0
Alvocidib (uM)
0
10
30
100
300
1000
3000
10000
Inhibition (%)
100
75
50
25
0
45.9 45.5 50.2 49.1 77.8 99.9 100 100
18.7 16.1 9.07 2.46 44.5 99.7 100 100
16 12.9 20.9 3.63 39.6 99.7 99.9 100
−7.13 1.24 −6.95 −11.2 27.9 99.7 99.9 100
−10.8 4.64 0.323 3.46 31.1 99.8 99.9 100
−3.34 −14.3 −5.02 −10.6 34.9 99.7 100 100
−4.74 −5.01 −4.12 8.72 36.4 99.6 99.9 100
0 −0.786 11.5 −1.79 35.3 99.7 99.9 100
45.9 45.5 50.2 49.1 77.8
18.7 16.1 9.07 2.46 44.5
16 12.9 20.9 3.63 39.6
−7.13 1.24 −6.95 −11.2 27.9
−10.8 4.64 0.323 3.46 31.1
−3.34 −14.3 −5.02 −10.6 34.9
−4.74 −5.01 −4.12 8.72 36.4
0 −0.786 11.5 −1.79 35.3

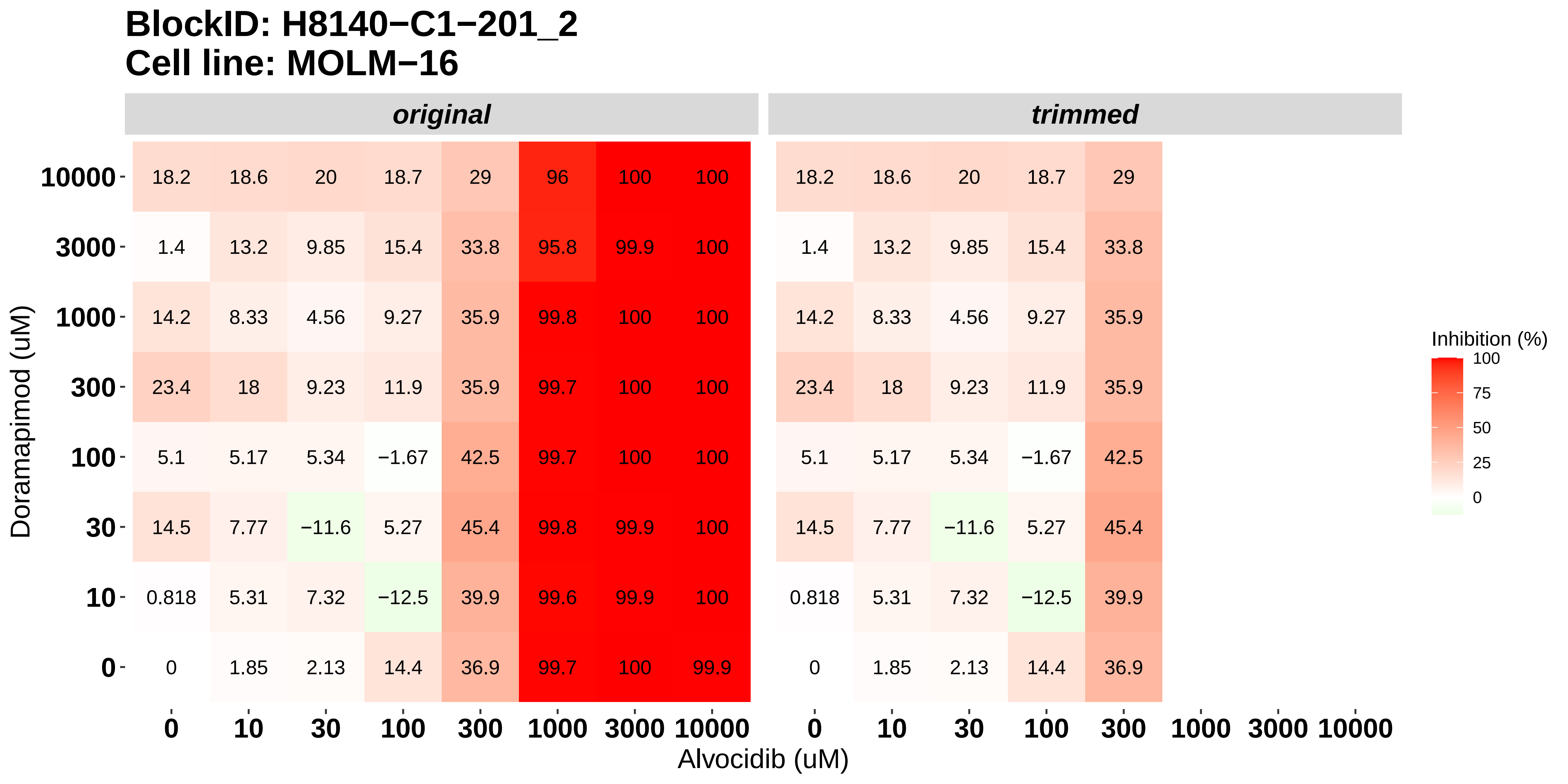
BlockID: H8140−C1−201_2
Cell line: MOLM−16
original
trimmed
Doramapimod (uM)
Alvocidib (uM)
10000
3000
1000
300
100
30
10
0
0
10
30
100
300
1000
3000
10000
18.2 18.6 20 18.7 29 96 100 100
1.4 13.2 9.85 15.4 33.8 95.8 99.9 100
14.2 8.33 4.56 9.27 35.9 99.8 100 100
23.4 18 9.23 11.9 35.9 99.7 100 100
5.1 5.17 5.34 −1.67 42.5 99.7 100 100
14.5 7.77 −11.6 5.27 45.4 99.8 99.9 100
0.818 5.31 7.32 −12.5 39.9 99.6 99.9 100
0 1.85 2.13 14.4 36.9 99.7 100 99.9
18.2 18.6 20 18.7 29
1.4 13.2 9.85 15.4 33.8
14.2 8.33 4.56 9.27 35.9
23.4 18 9.23 11.9 35.9
5.1 5.17 5.34 −1.67 42.5
14.5 7.77 −11.6 5.27 45.4
0.818 5.31 7.32 −12.5 39.9
0 1.85 2.13 14.4 36.9
Inhibition (%)
100
75
50
25
0

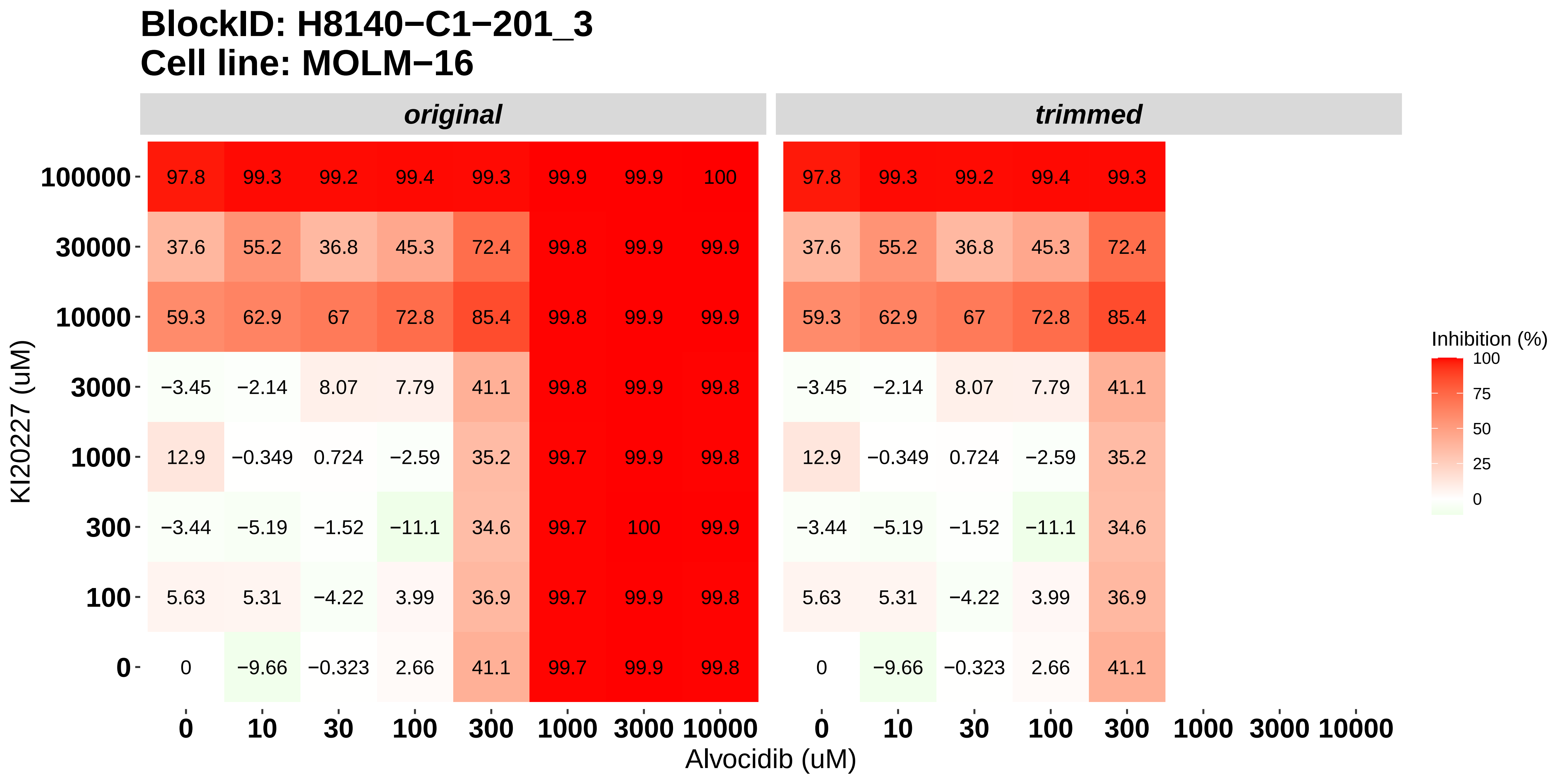
BlockID: H8140−C1−201_3
Cell line: MOLM−16
original
trimmed
100000
97.8 99.3 99.2 99.4 99.3 99.9 99.9 100
30000
37.6 55.2 36.8 45.3 72.4 99.8 99.9 99.9
10000
59.3 62.9 67 72.8 85.4 99.8 99.9 99.9
3000
−3.45 −2.14 8.07 7.79 41.1 99.8 99.9 99.8
1000
12.9 −0.349 0.724 −2.59 35.2 99.7 99.9 99.8
300
−3.44 −5.19 −1.52 −11.1 34.6 99.7 100 99.9
100
5.63 5.31 −4.22 3.99 36.9 99.7 99.9 99.8
0
0 −9.66 −0.323 2.66 41.1 99.7 99.9 99.8
97.8 99.3 99.2 99.4 99.3
37.6 55.2 36.8 45.3 72.4
59.3 62.9 67 72.8 85.4
−3.45 −2.14 8.07 7.79 41.1
12.9 −0.349 0.724 −2.59 35.2
−3.44 −5.19 −1.52 −11.1 34.6
5.63 5.31 −4.22 3.99 36.9
0 −9.66 −0.323 2.66 41.1
0 10 30 100 300 1000 3000 10000
0 10 30 100 300 1000 3000 10000
KI20227 (uM)
Alvocidib (uM)
Inhibition (%)
100
75
50
25
0

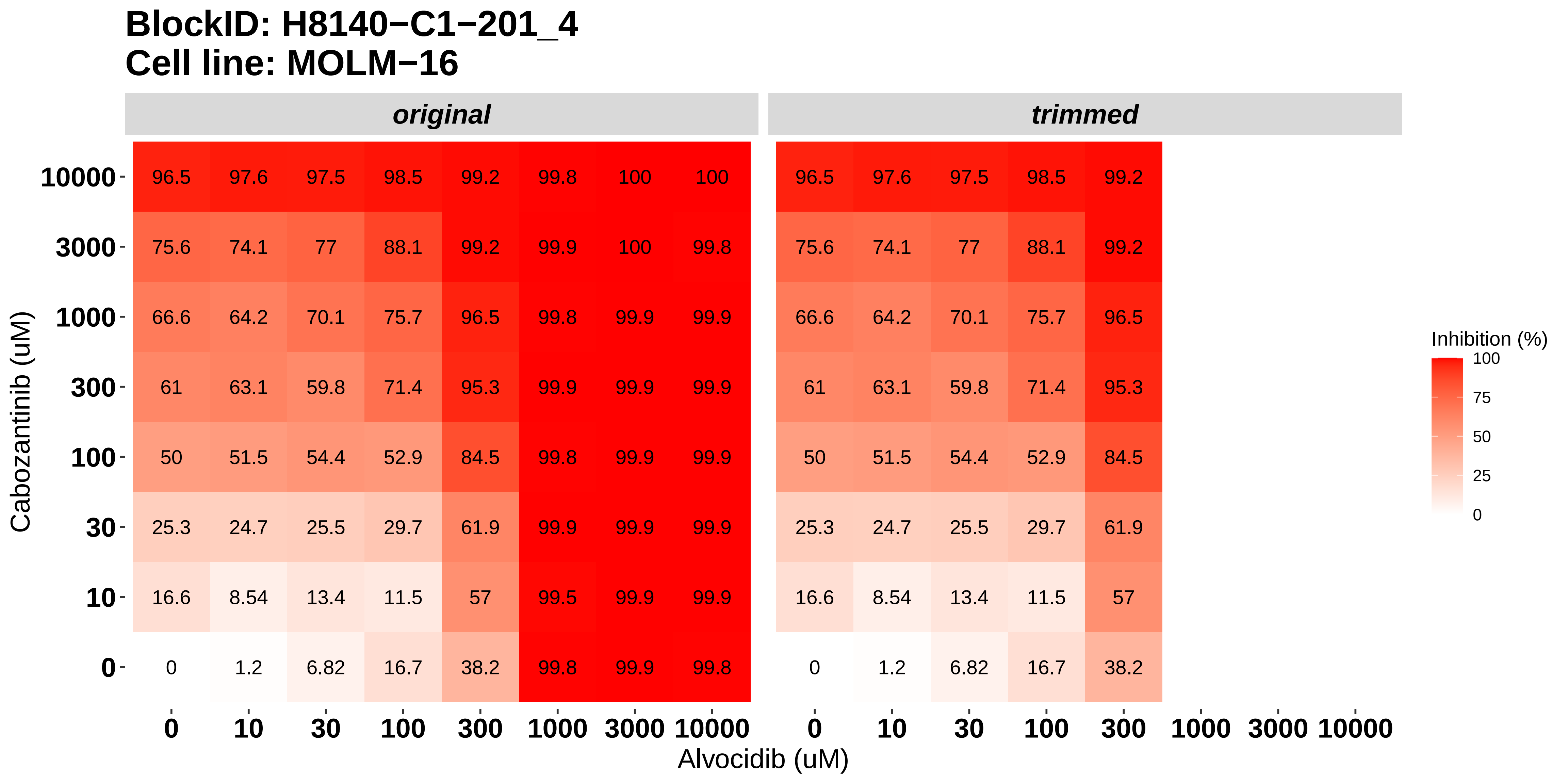

BlockID: H8140−C1−201_4
Cell line: MOLM−16
original
trimmed
Cabozantinib (uM)
Alvocidib (uM)
Inhibition (%)
100
75
50
25
0
10000
3000
1000
300
100
30
10
0
96.5 97.6 97.5 98.5 99.2 99.8 100 100
75.6 74.1 77 88.1 99.2 99.9 100 99.8
66.6 64.2 70.1 75.7 96.5 99.8 99.9 99.9
61 63.1 59.8 71.4 95.3 99.9 99.9 99.9
50 51.5 54.4 52.9 84.5 99.8 99.9 99.9
25.3 24.7 25.5 29.7 61.9 99.9 99.9 99.9
16.6 8.54 13.4 11.5 57 99.5 99.9 99.9
0 1.2 6.82 16.7 38.2 99.8 99.9 99.8
96.5 97.6 97.5 98.5 99.2
75.6 74.1 77 88.1 99.2
66.6 64.2 70.1 75.7 96.5
61 63.1 59.8 71.4 95.3
50 51.5 54.4 52.9 84.5
25.3 24.7 25.5 29.7 61.9
16.6 8.54 13.4 11.5 57
0 1.2 6.82 16.7 38.2
0 10 30 100 300 1000 3000 10000
0 10 30 100 300 1000 3000 10000

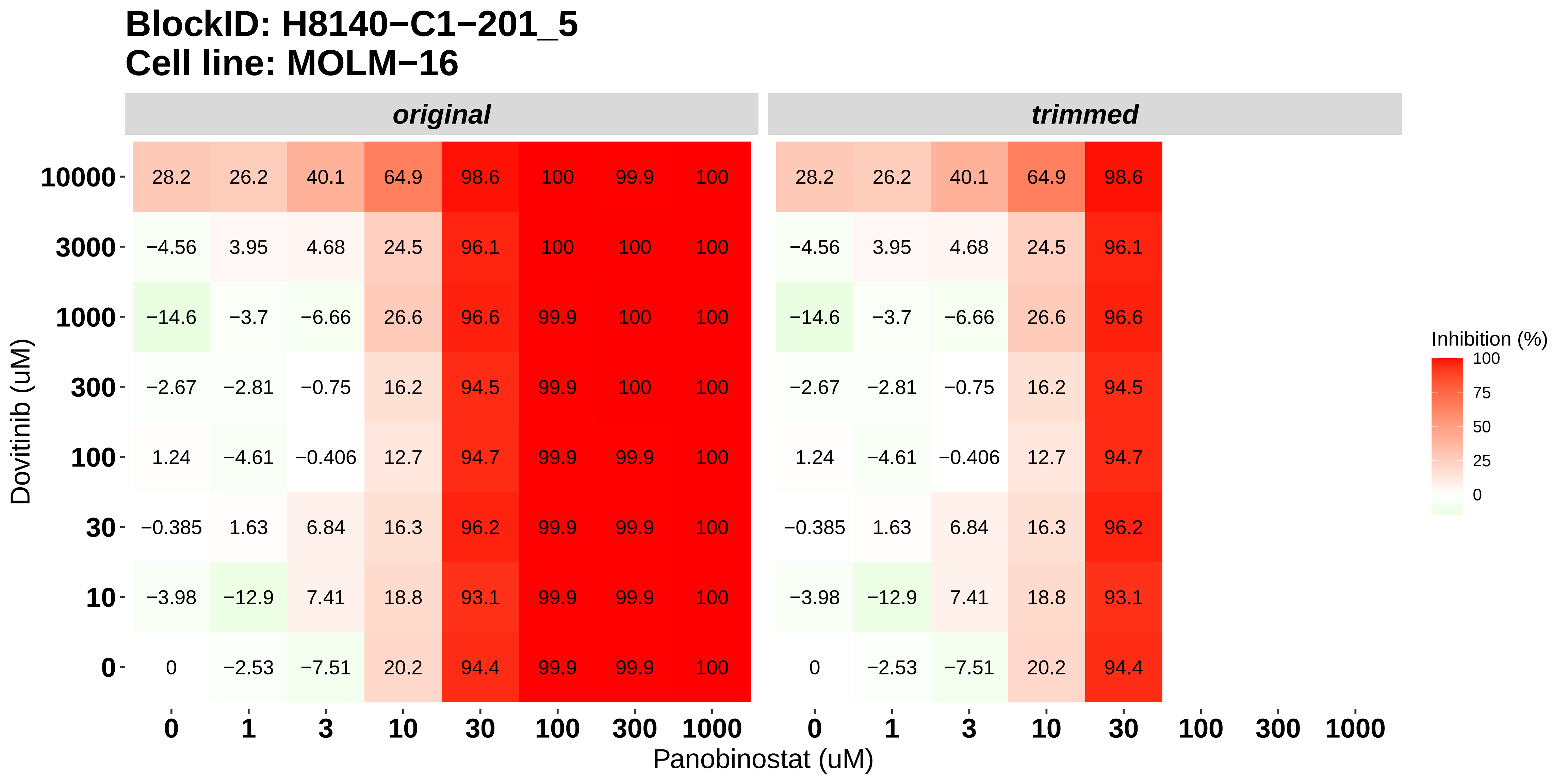
BlockID: H8140−C1−201_5
Cell line: MOLM−16
original
trimmed
Dovitinib (uM)
Panobinostat (uM)
Inhibition (%)
100
75
50
25
0
10000
3000
1000
300
100
30
10
0
0
1
3
10
30
100
300
1000
28.2 26.2 40.1 64.9 98.6 100 99.9 100
−4.56 3.95 4.68 24.5 96.1 100 100 100
−14.6 −3.7 −6.66 26.6 96.6 99.9 100 100
−2.67 −2.81 −0.75 16.2 94.5 99.9 100 100
1.24 −4.61 −0.406 12.7 94.7 99.9 99.9 100
−0.385 1.63 6.84 16.3 96.2 99.9 99.9 100
−3.98 −12.9 7.41 18.8 93.1 99.9 99.9 100
0 −2.53 −7.51 20.2 94.4 99.9 99.9 100
28.2 26.2 40.1 64.9 98.6
−4.56 3.95 4.68 24.5 96.1
−14.6 −3.7 −6.66 26.6 96.6
−2.67 −2.81 −0.75 16.2 94.5
1.24 −4.61 −0.406 12.7 94.7
−0.385 1.63 6.84 16.3 96.2
−3.98 −12.9 7.41 18.8 93.1
0 −2.53 −7.51 20.2 94.4

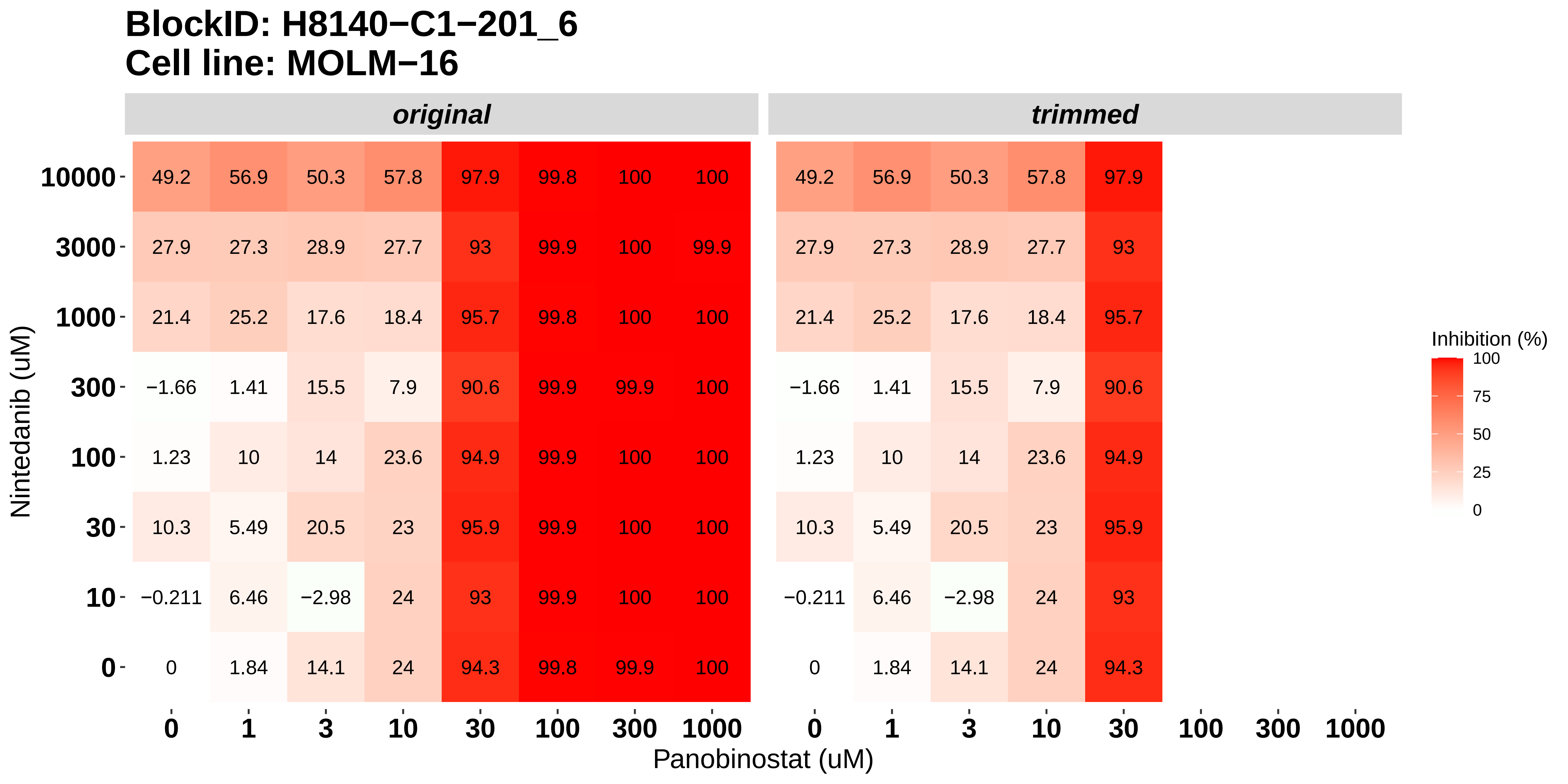
BlockID: H8140−C1−201_6
Cell line: MOLM−16
original
trimmed
Nintedanib (uM)
10000
3000
1000
300
100
30
10
0
Panobinostat (uM)
0
1
3
10
30
100
300
1000
Inhibition (%)
100
75
50
25
0
49.2 56.9 50.3 57.8 97.9 99.8 100 100
27.9 27.3 28.9 27.7 93 99.9 100 99.9
21.4 25.2 17.6 18.4 95.7 99.8 100 100
−1.66 1.41 15.5 7.9 90.6 99.9 99.9 100
1.23 10 14 23.6 94.9 99.9 100 100
10.3 5.49 20.5 23 95.9 99.9 100 100
−0.211 6.46 −2.98 24 93 99.9 100 100
0 1.84 14.1 24 94.3 99.8 99.9 100
49.2 56.9 50.3 57.8 97.9
27.9 27.3 28.9 27.7 93
21.4 25.2 17.6 18.4 95.7
−1.66 1.41 15.5 7.9 90.6
1.23 10 14 23.6 94.9
10.3 5.49 20.5 23 95.9
−0.211 6.46 −2.98 24 93
0 1.84 14.1 24 94.3

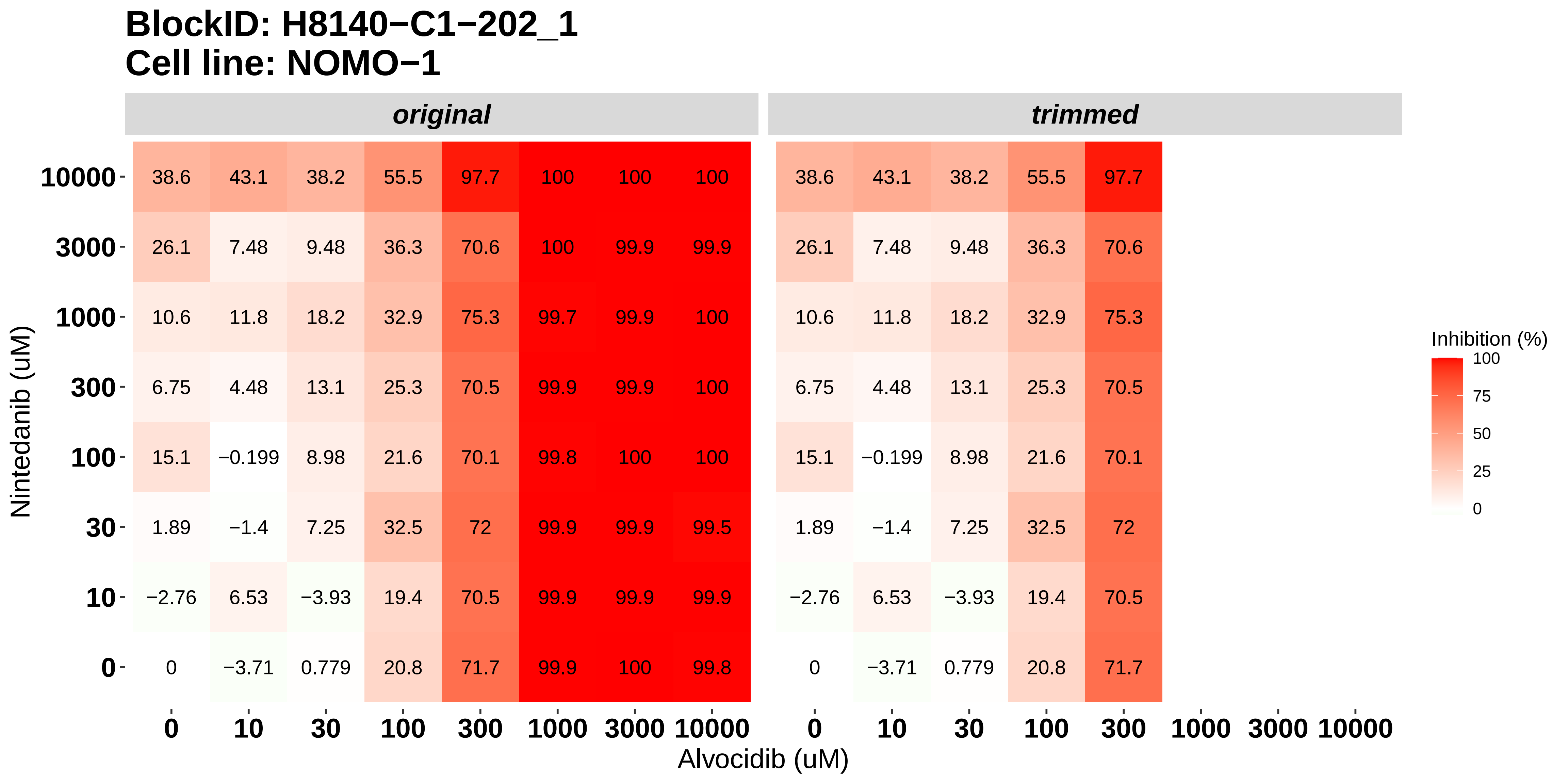
BlockID: H8140−C1−202_1
Cell line: NOMO−1
original
trimmed
Nintedanib (uM)
Alvocidib (uM)
Inhibition (%)
10000
3000
1000
300
100
30
10
0
38.6 43.1 38.2 55.5 97.7 100 100 100
26.1 7.48 9.48 36.3 70.6 100 99.9 99.9
10.6 11.8 18.2 32.9 75.3 99.7 99.9 100
6.75 4.48 13.1 25.3 70.5 99.9 99.9 100
15.1 −0.199 8.98 21.6 70.1 99.8 100 100
1.89 −1.4 7.25 32.5 72 99.9 99.9 99.5
−2.76 6.53 −3.93 19.4 70.5 99.9 99.9 99.9
0 −3.71 0.779 20.8 71.7 99.9 100 99.8
38.6 43.1 38.2 55.5 97.7
26.1 7.48 9.48 36.3 70.6
10.6 11.8 18.2 32.9 75.3
6.75 4.48 13.1 25.3 70.5
15.1 −0.199 8.98 21.6 70.1
1.89 −1.4 7.25 32.5 72
−2.76 6.53 −3.93 19.4 70.5
0 −3.71 0.779 20.8 71.7
0 10 30 100 300 1000 3000 10000
100 75 50 25 0

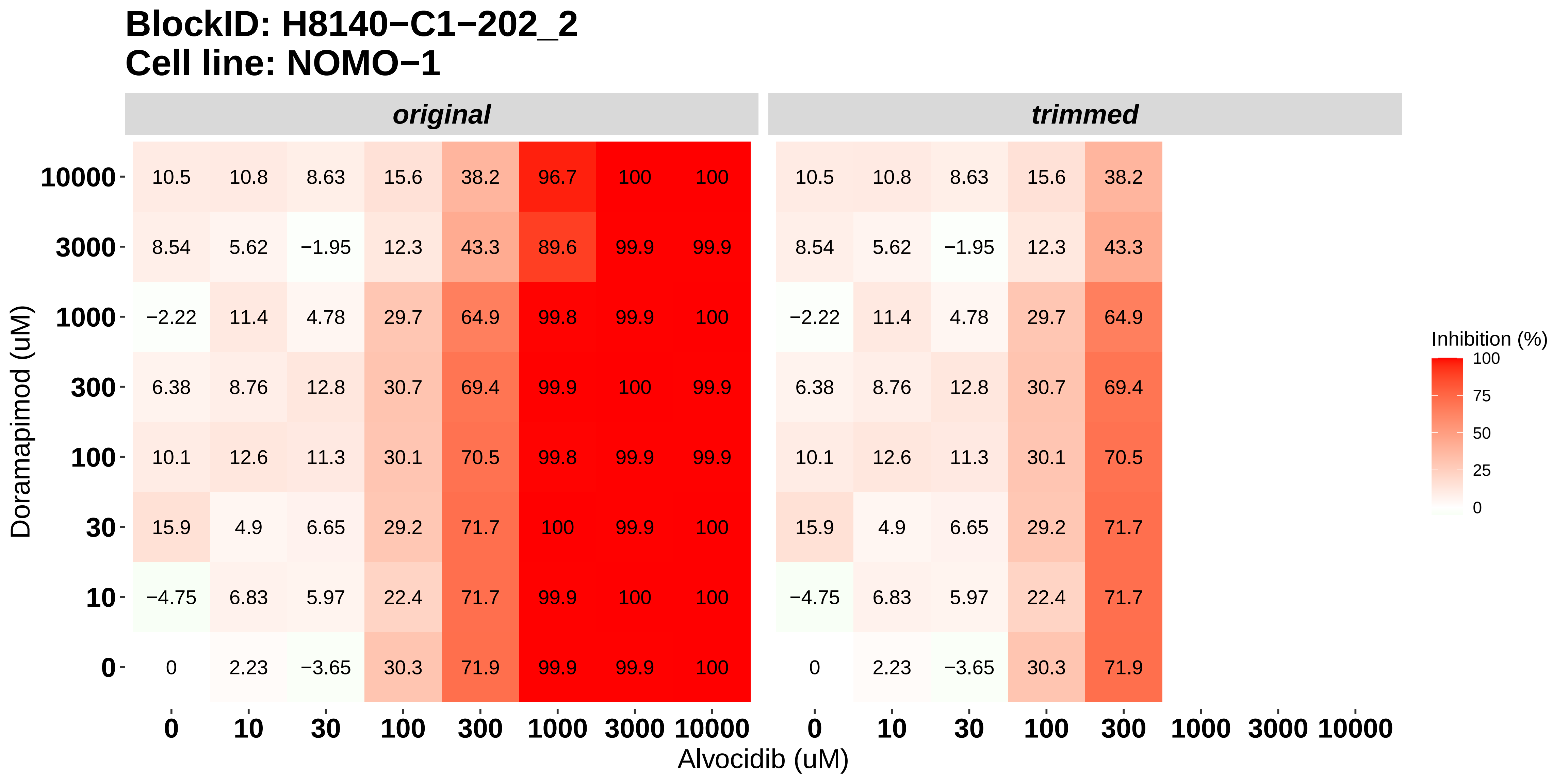

BlockID: H8140−C1−202_2
Cell line: NOMO−1
original
trimmed
Doramapimod (uM)
Alvocidib (uM)
Inhibition (%)
100
75
50
25
0
10000
3000
1000
300
100
30
10
0
10.5 10.8 8.63 15.6 38.2 96.7 100 100
8.54 5.62 −1.95 12.3 43.3 89.6 99.9 99.9
−2.22 11.4 4.78 29.7 64.9 99.8 99.9 100
6.38 8.76 12.8 30.7 69.4 99.9 100 99.9
10.1 12.6 11.3 30.1 70.5 99.8 99.9 99.9
15.9 4.9 6.65 29.2 71.7 100 99.9 100
−4.75 6.83 5.97 22.4 71.7 99.9 100 100
0 2.23 −3.65 30.3 71.9 99.9 99.9 100
0 10 30 100 300 1000 3000 10000
10.5 10.8 8.63 15.6 38.2
8.54 5.62 −1.95 12.3 43.3
−2.22 11.4 4.78 29.7 64.9
6.38 8.76 12.8 30.7 69.4
10.1 12.6 11.3 30.1 70.5
15.9 4.9 6.65 29.2 71.7
−4.75 6.83 5.97 22.4 71.7
0 2.23 −3.65 30.3 71.9
0 10 30 100 300 1000 3000 10000

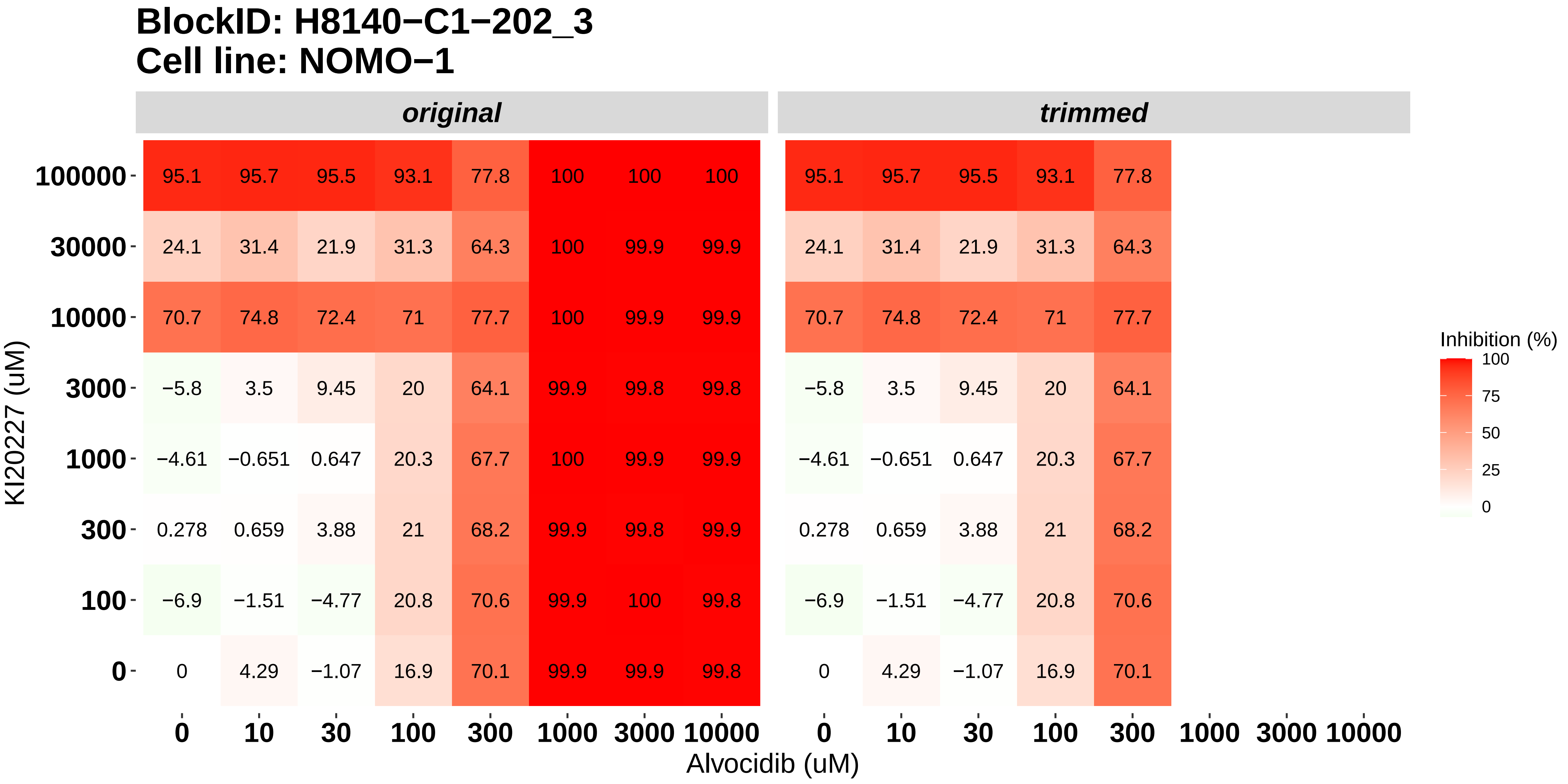
BlockID: H8140−C1−202_3
Cell line: NOMO−1
original
trimmed
100000
30000
10000
3000
1000
300
100
0
KI20227 (uM)
95.1 95.7 95.5 93.1 77.8 100 100 100
24.1 31.4 21.9 31.3 64.3 100 99.9 99.9
70.7 74.8 72.4 71 77.7 100 99.9 99.9
−5.8 3.5 9.45 20 64.1 99.9 99.8 99.8
−4.61 −0.651 0.647 20.3 67.7 100 99.9 99.9
0.278 0.659 3.88 21 68.2 99.9 99.8 99.9
−6.9 −1.51 −4.77 20.8 70.6 99.9 100 99.8
0 4.29 −1.07 16.9 70.1 99.9 99.9 99.8
95.1 95.7 95.5 93.1 77.8
24.1 31.4 21.9 31.3 64.3
70.7 74.8 72.4 71 77.7
−5.8 3.5 9.45 20 64.1
−4.61 −0.651 0.647 20.3 67.7
0.278 0.659 3.88 21 68.2
−6.9 −1.51 −4.77 20.8 70.6
0 4.29 −1.07 16.9 70.1
0 10 30 100 300 1000 3000 10000
0 10 30 100 300 1000 3000 10000
Alvocidib (uM)
Inhibition (%)
100
75
50
25
0

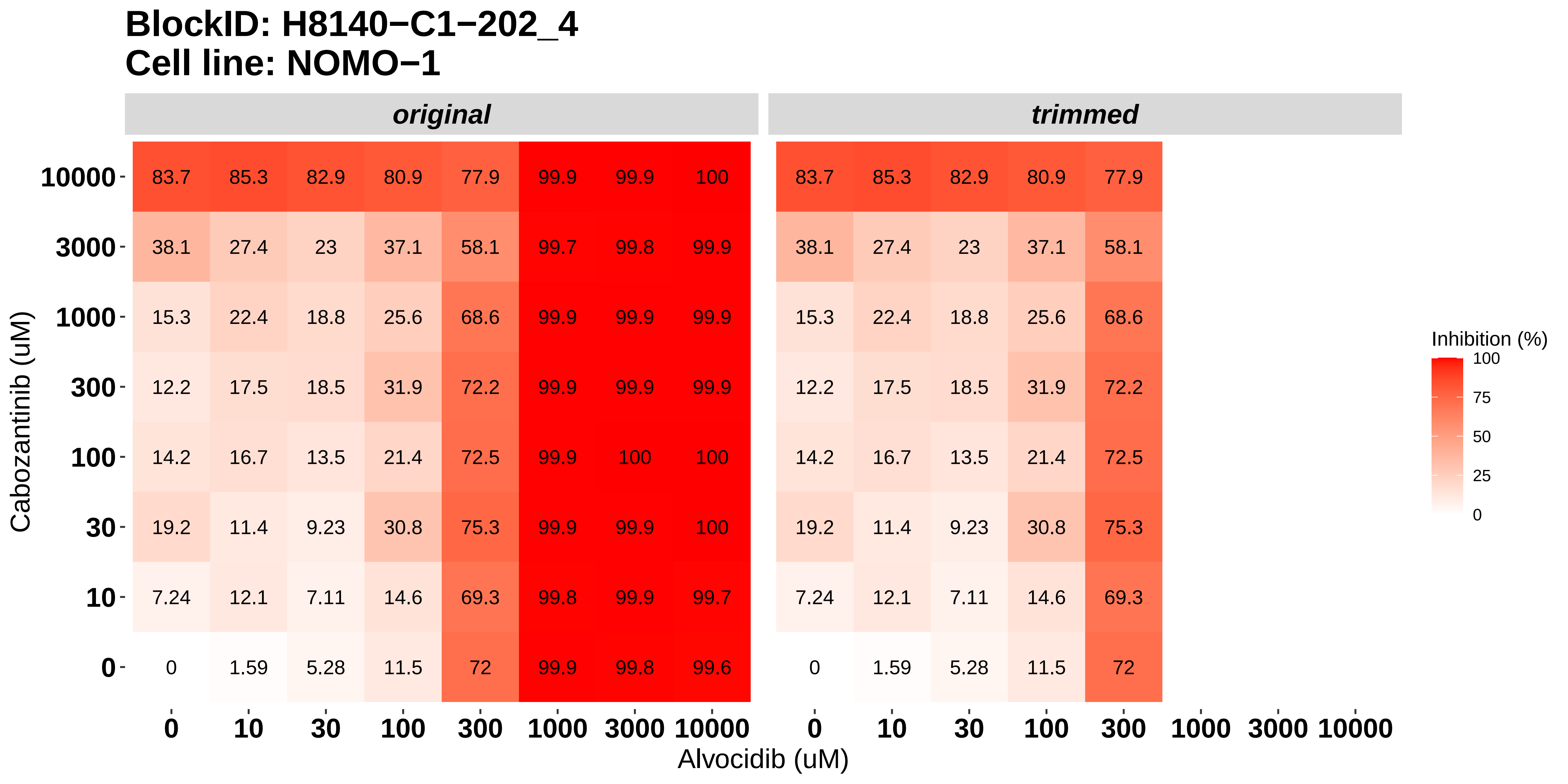
BlockID: H8140−C1−202_4
Cell line: NOMO−1
original
trimmed
Cabozantinib (uM)
Alvocidib (uM)
Inhibition (%)
100
75
50
25
0
10000
3000
1000
300
100
30
10
0
83.7 85.3 82.9 80.9 77.9 99.9 99.9 100
38.1 27.4 23 37.1 58.1 99.7 99.8 99.9
15.3 22.4 18.8 25.6 68.6 99.9 99.9 99.9
12.2 17.5 18.5 31.9 72.2 99.9 99.9 99.9
14.2 16.7 13.5 21.4 72.5 99.9 100 100
19.2 11.4 9.23 30.8 75.3 99.9 99.9 100
7.24 12.1 7.11 14.6 69.3 99.8 99.9 99.7
0 1.59 5.28 11.5 72 99.9 99.8 99.6
83.7 85.3 82.9 80.9 77.9
38.1 27.4 23 37.1 58.1
15.3 22.4 18.8 25.6 68.6
12.2 17.5 18.5 31.9 72.2
14.2 16.7 13.5 21.4 72.5
19.2 11.4 9.23 30.8 75.3
7.24 12.1 7.11 14.6 69.3
0 1.59 5.28 11.5 72
0 10 30 100 300 1000 3000 10000
0 10 30 100 300 1000 3000 10000

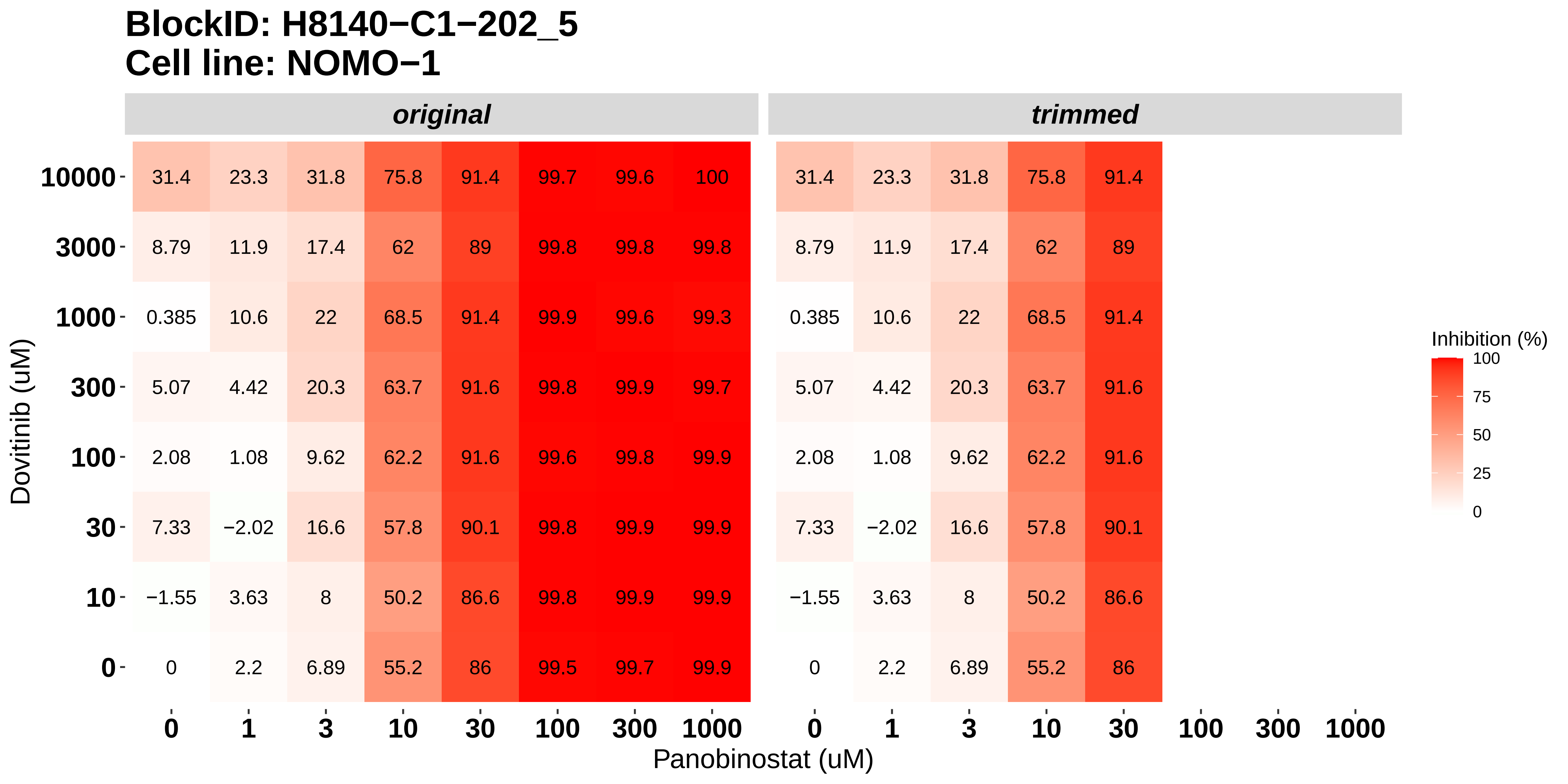
BlockID: H8140−C1−202_5
Cell line: NOMO−1
original
trimmed
Dovitinib (uM)
Panobinostat (uM)
Inhibition (%)
100
75
50
25
0
10000
3000
1000
300
100
30
10
0
1
3
10
30
100
300
1000
31.4 23.3 31.8 75.8 91.4 99.7 99.6 100
8.79 11.9 17.4 62 89 99.8 99.8 99.8
0.385 10.6 22 68.5 91.4 99.9 99.6 99.3
5.07 4.42 20.3 63.7 91.6 99.8 99.9 99.7
2.08 1.08 9.62 62.2 91.6 99.6 99.8 99.9
7.33 −2.02 16.6 57.8 90.1 99.8 99.9 99.9
−1.55 3.63 8 50.2 86.6 99.8 99.9 99.9
0 2.2 6.89 55.2 86 99.5 99.7 99.9
31.4 23.3 31.8 75.8 91.4
8.79 11.9 17.4 62 89
0.385 10.6 22 68.5 91.4
5.07 4.42 20.3 63.7 91.6
2.08 1.08 9.62 62.2 91.6
7.33 −2.02 16.6 57.8 90.1
−1.55 3.63 8 50.2 86.6
0 2.2 6.89 55.2 86

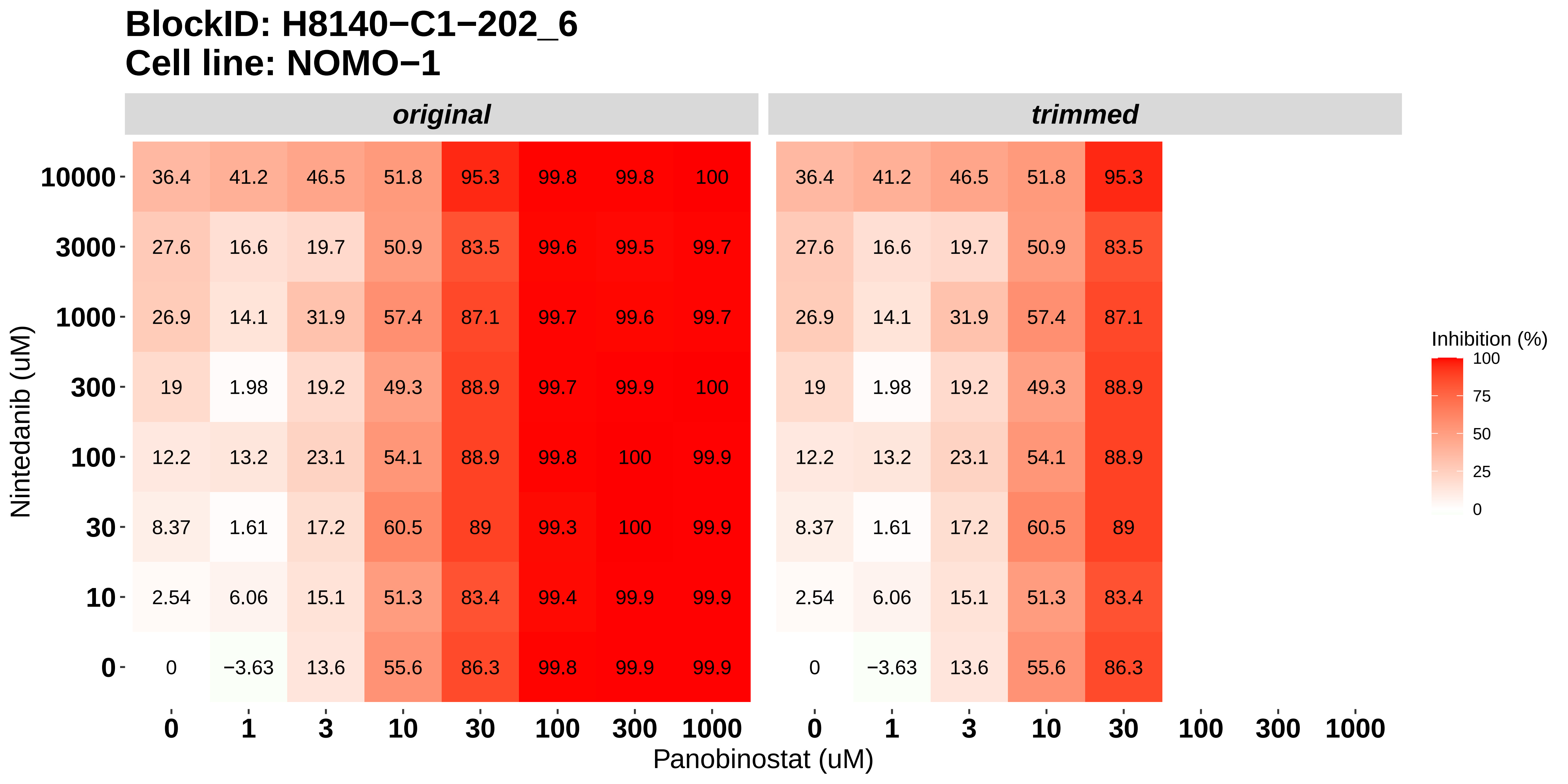
BlockID: H8140−C1−202_6
Cell line: NOMO−1
original
trimmed
Nintedanib (uM)
Panobinostat (uM)
Inhibition (%)
100
75
50
25
0
10000 36.4 41.2 46.5 51.8 95.3 99.8 99.8 100
3000 27.6 16.6 19.7 50.9 83.5 99.6 99.5 99.7
1000 26.9 14.1 31.9 57.4 87.1 99.7 99.6 99.7
300 19 1.98 19.2 49.3 88.9 99.7 99.9 100
100 12.2 13.2 23.1 54.1 88.9 99.8 100 99.9
30 8.37 1.61 17.2 60.5 89 99.3 100 99.9
10 2.54 6.06 15.1 51.3 83.4 99.4 99.9 99.9
0 0 −3.63 13.6 55.6 86.3 99.8 99.9 99.9
0 1 3 10 30 100 300 1000
36.4 41.2 46.5 51.8 95.3
27.6 16.6 19.7 50.9 83.5
26.9 14.1 31.9 57.4 87.1
19 1.98 19.2 49.3 88.9
12.2 13.2 23.1 54.1 88.9
8.37 1.61 17.2 60.5 89
2.54 6.06 15.1 51.3 83.4
0 −3.63 13.6 55.6 86.3
0 1 3 10 30 100 300 1000

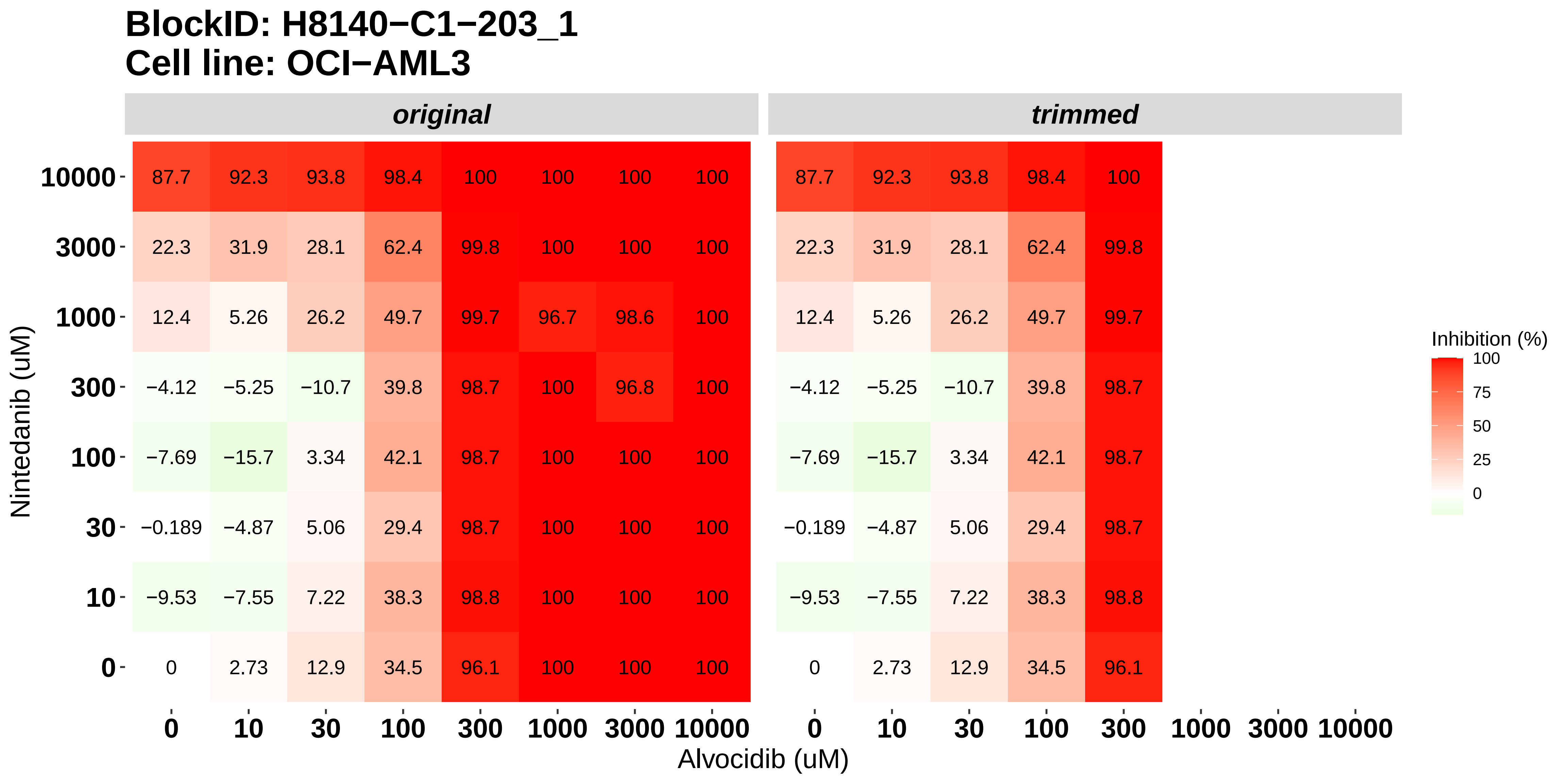
BlockID: H8140−C1−203_1
Cell line: OCI−AML3
original
trimmed
Nintedanib (uM)
Alvocidib (uM)
Inhibition (%)
100
75
50
25
0
10000 87.7 92.3 93.8 98.4 100 100 100 100
3000 22.3 31.9 28.1 62.4 99.8 100 100 100
1000 12.4 5.26 26.2 49.7 99.7 96.7 98.6 100
300 −4.12 −5.25 −10.7 39.8 98.7 100 96.8 100
100 −7.69 −15.7 3.34 42.1 98.7 100 100 100
30 −0.189 −4.87 5.06 29.4 98.7 100 100 100
10 −9.53 −7.55 7.22 38.3 98.8 100 100 100
0 0 2.73 12.9 34.5 96.1 100 100 100
0 10 30 100 300 1000 3000 10000
87.7 92.3 93.8 98.4 100
22.3 31.9 28.1 62.4 99.8
12.4 5.26 26.2 49.7 99.7
−4.12 −5.25 −10.7 39.8 98.7
−7.69 −15.7 3.34 42.1 98.7
−0.189 −4.87 5.06 29.4 98.7
−9.53 −7.55 7.22 38.3 98.8
0 2.73 12.9 34.5 96.1
0 10 30 100 300 1000 3000 10000

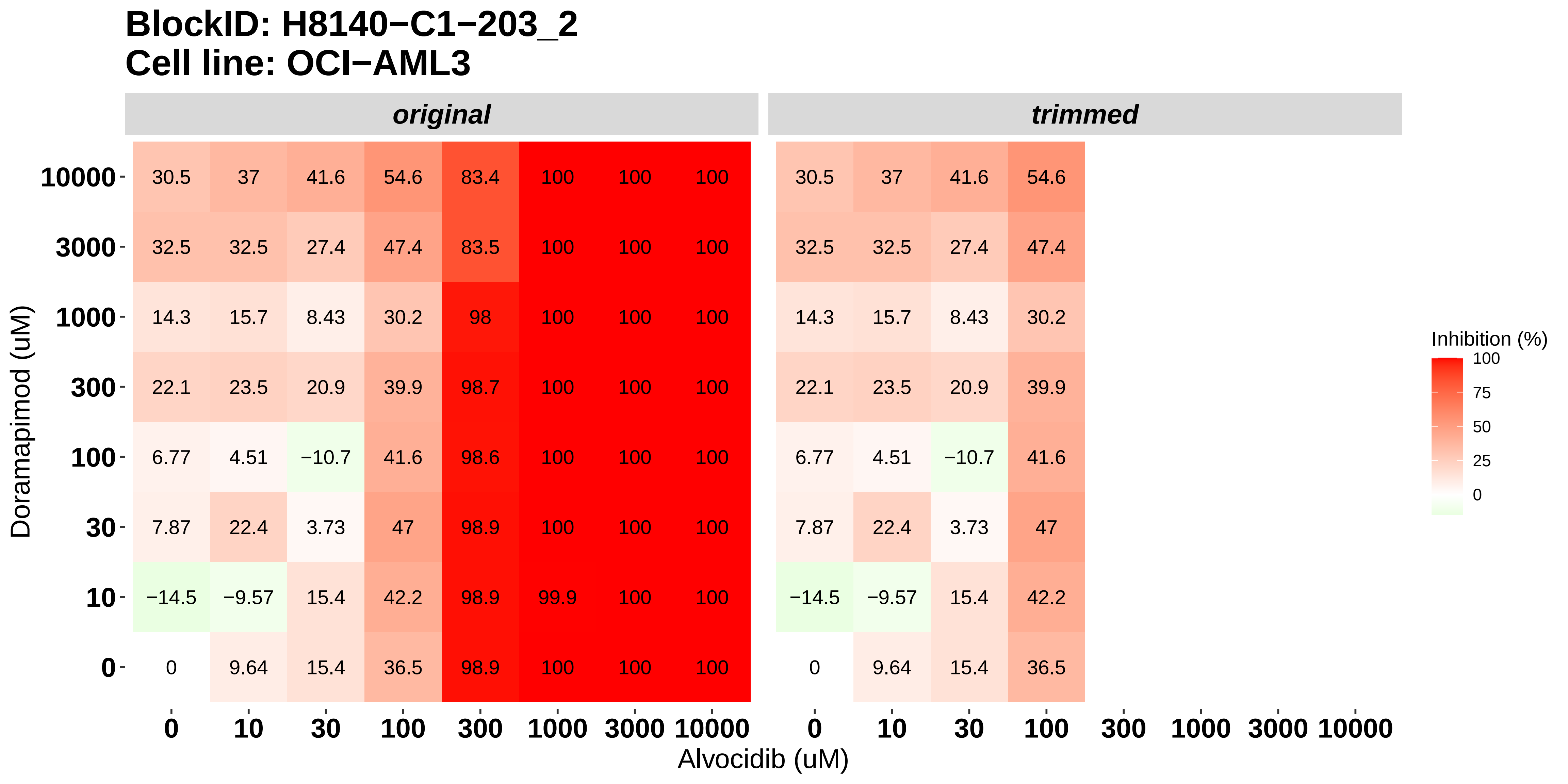
BlockID: H8140−C1−203_2
Cell line: OCI−AML3
original
trimmed
Doramapimod (uM)
10000
3000
1000
300
100
30
10
0
30.5 37 41.6 54.6 83.4 100 100 100
32.5 32.5 27.4 47.4 83.5 100 100 100
14.3 15.7 8.43 30.2 98 100 100 100
22.1 23.5 20.9 39.9 98.7 100 100 100
6.77 4.51 −10.7 41.6 98.6 100 100 100
7.87 22.4 3.73 47 98.9 100 100 100
−14.5 −9.57 15.4 42.2 98.9 99.9 100 100
0 9.64 15.4 36.5 98.9 100 100 100
30.5 37 41.6 54.6
32.5 32.5 27.4 47.4
14.3 15.7 8.43 30.2
22.1 23.5 20.9 39.9
6.77 4.51 −10.7 41.6
7.87 22.4 3.73 47
−14.5 −9.57 15.4 42.2
0 9.64 15.4 36.5
0 10 30 100 300 1000 3000 10000
0 10 30 100 300 1000 3000 10000
Alvocidib (uM)
Inhibition (%)
100
75
50
25
0

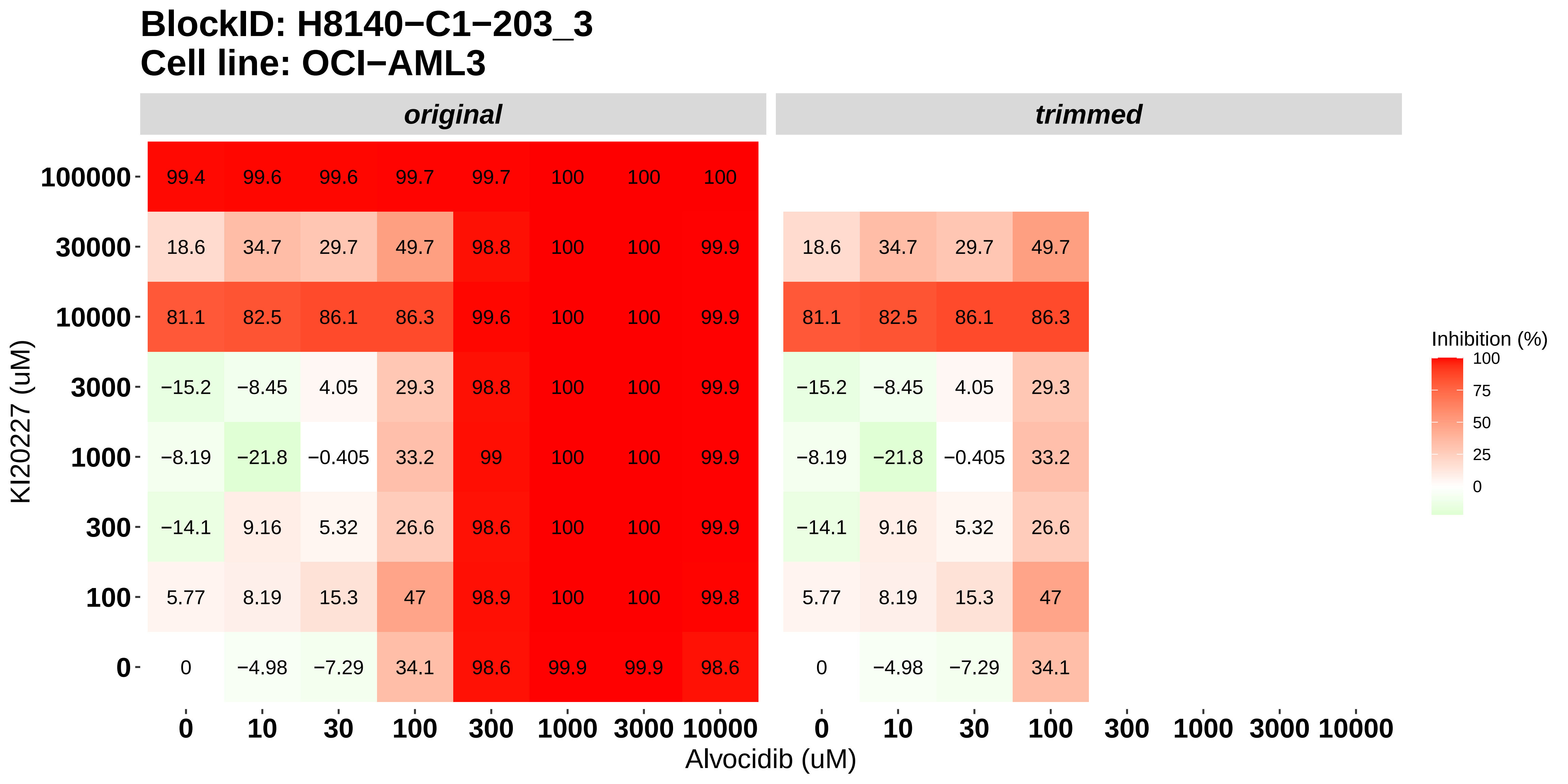

BlockID: H8140−C1−203_3
Cell line: OCI−AML3
original
trimmed
KI20227 (uM)
Alvocidib (uM)
Inhibition (%)
100000
30000
10000
3000
1000
300
100
0
0
10
30
100
300
1000
3000
10000
99.4 99.6 99.6 99.7 99.7 100 100 100
18.6 34.7 29.7 49.7 98.8 100 100 99.9
81.1 82.5 86.1 86.3 99.6 100 100 99.9
−15.2 −8.45 4.05 29.3 98.8 100 100 99.9
−8.19 −21.8 −0.405 33.2 99 100 100 99.9
−14.1 9.16 5.32 26.6 98.6 100 100 99.9
5.77 8.19 15.3 47 98.9 100 100 99.8
0 −4.98 −7.29 34.1 98.6 99.9 99.9 98.6
18.6 34.7 29.7 49.7
81.1 82.5 86.1 86.3
−15.2 −8.45 4.05 29.3
−8.19 −21.8 −0.405 33.2
−14.1 9.16 5.32 26.6
5.77 8.19 15.3 47
0 −4.98 −7.29 34.1
100
75
50
25
0

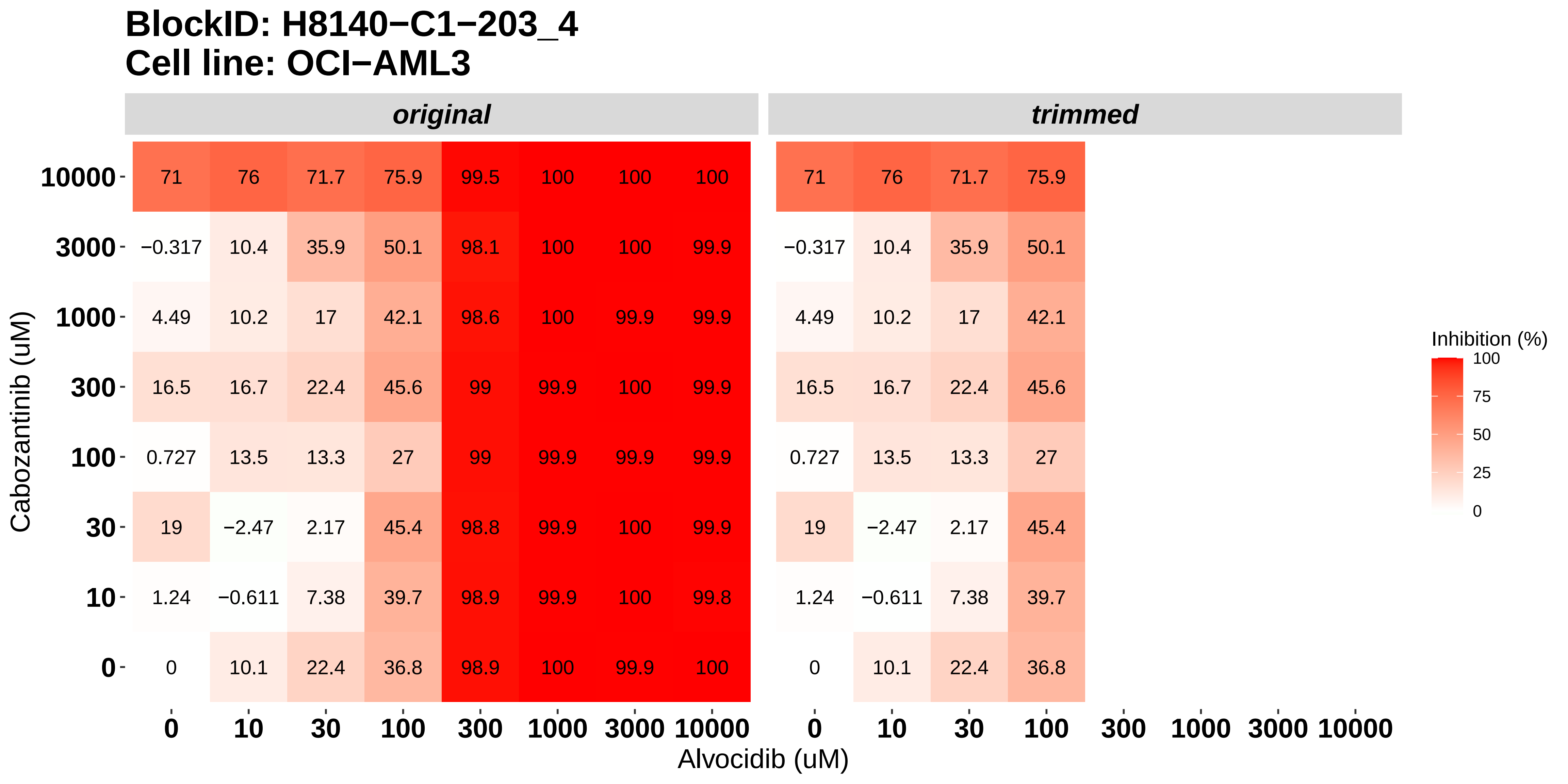
BlockID: H8140−C1−203_4
Cell line: OCI−AML3
original
trimmed
Cabozantinib (uM)
Alvocidib (uM)
10000 3000 1000 300 100 30 10 0
0 10 30 100 300 1000 3000 10000
71 76 71.7 75.9 99.5 100 100 100
−0.317 10.4 35.9 50.1 98.1 100 100 99.9
4.49 10.2 17 42.1 98.6 100 99.9 99.9
16.5 16.7 22.4 45.6 99 99.9 100 99.9
0.727 13.5 13.3 27 99 99.9 99.9 99.9
19 −2.47 2.17 45.4 98.8 99.9 100 99.9
1.24 −0.611 7.38 39.7 98.9 99.9 100 99.8
0 10.1 22.4 36.8 98.9 100 99.9 100
71 76 71.7 75.9
−0.317 10.4 35.9 50.1
4.49 10.2 17 42.1
16.5 16.7 22.4 45.6
0.727 13.5 13.3 27
19 −2.47 2.17 45.4
1.24 −0.611 7.38 39.7
0 10.1 22.4 36.8
Inhibition (%)
100
75
50
25
0

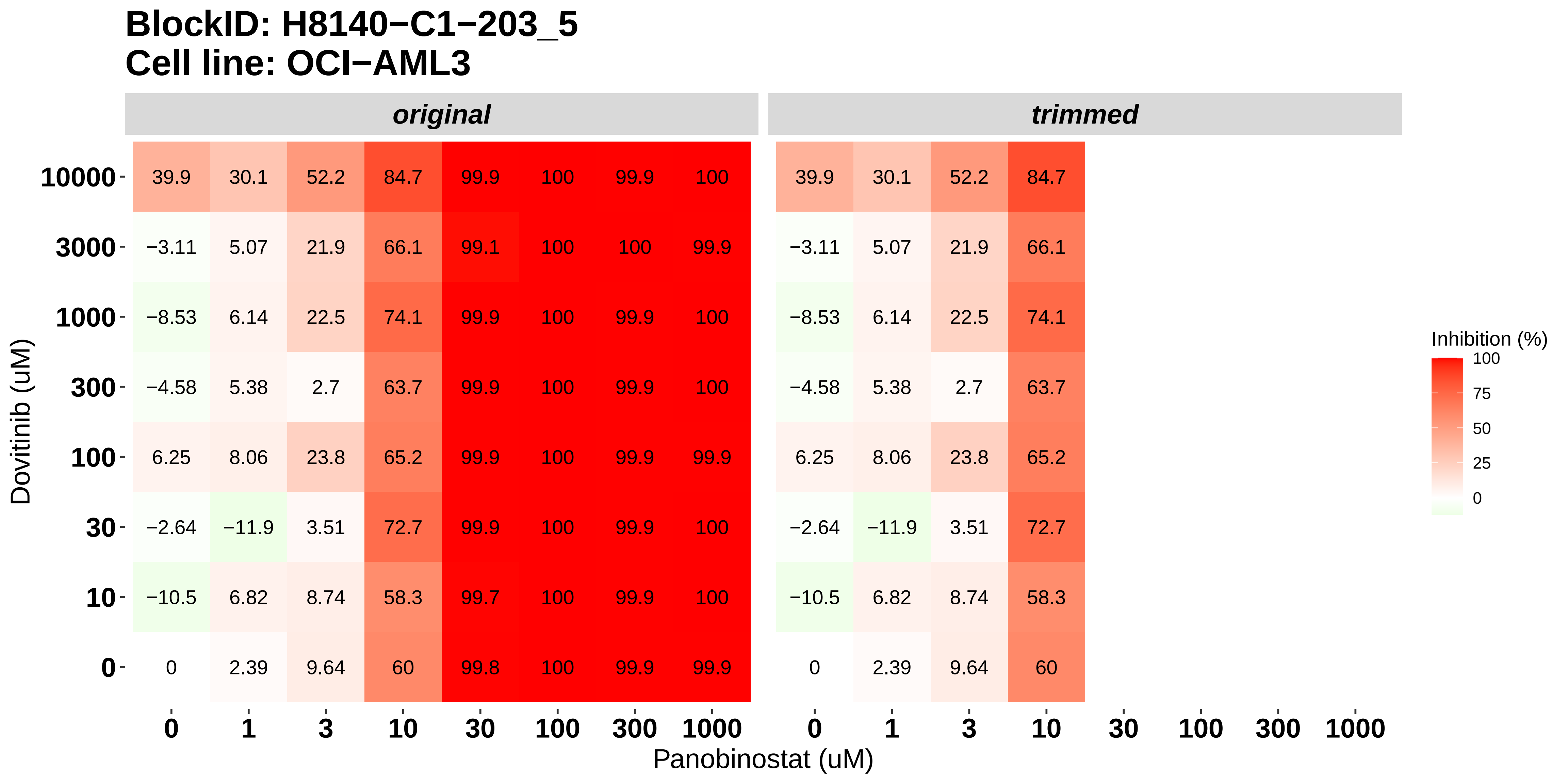
BlockID: H8140−C1−203_5
Cell line: OCI−AML3
original
trimmed
Dovitinib (uM)
Panobinostat (uM)
Inhibition (%)
100
75
50
25
0
10000
3000
1000
300
100
30
10
0
39.9 30.1 52.2 84.7 99.9 100 99.9 100
−3.11 5.07 21.9 66.1 99.1 100 100 99.9
−8.53 6.14 22.5 74.1 99.9 100 99.9 100
−4.58 5.38 2.7 63.7 99.9 100 99.9 100
6.25 8.06 23.8 65.2 99.9 100 99.9 99.9
−2.64 −11.9 3.51 72.7 99.9 100 99.9 100
−10.5 6.82 8.74 58.3 99.7 100 99.9 100
0 2.39 9.64 60 99.8 100 99.9 99.9
39.9 30.1 52.2 84.7
−3.11 5.07 21.9 66.1
−8.53 6.14 22.5 74.1
−4.58 5.38 2.7 63.7
6.25 8.06 23.8 65.2
−2.64 −11.9 3.51 72.7
−10.5 6.82 8.74 58.3
0 2.39 9.64 60
0 1 3 10 30 100 300 1000
0 1 3 10 30 100 300 1000

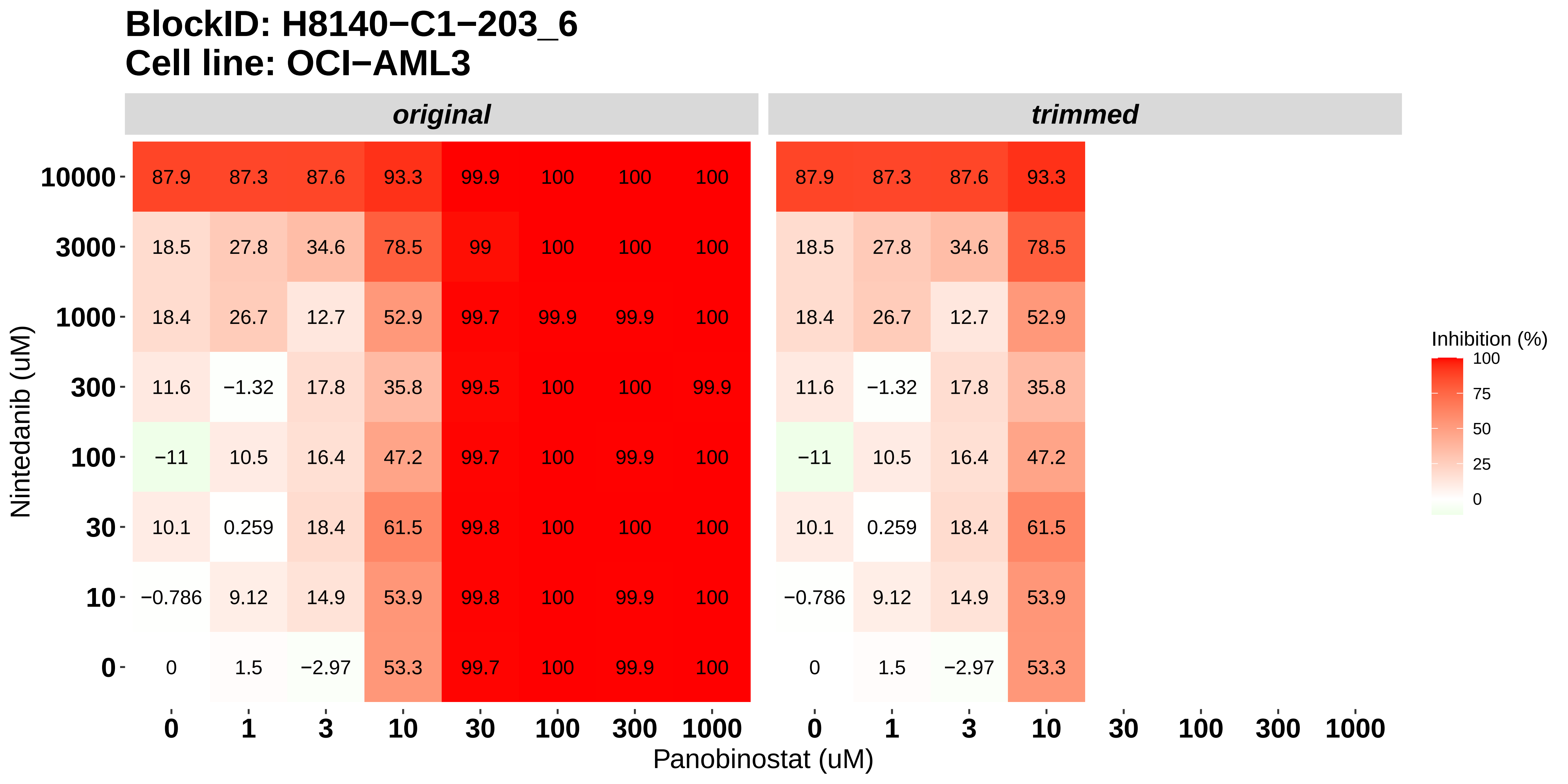
BlockID: H8140−C1−203_6
Cell line: OCI−AML3
original
trimmed
Nintedanib (uM)
Panobinostat (uM)
Inhibition (%)
100
75
50
25
0
10000
3000
1000
300
100
30
10
0
1
3
10
30
100
300
1000
87.9 87.3 87.6 93.3 99.9 100 100 100
18.5 27.8 34.6 78.5 99 100 100 100
18.4 26.7 12.7 52.9 99.7 99.9 99.9 100
11.6 −1.32 17.8 35.8 99.5 100 100 99.9
−11 10.5 16.4 47.2 99.7 100 99.9 100
10.1 0.259 18.4 61.5 99.8 100 100 100
−0.786 9.12 14.9 53.9 99.8 100 99.9 100
0 1.5 −2.97 53.3 99.7 100 99.9 100
87.9 87.3 87.6 93.3
18.5 27.8 34.6 78.5
18.4 26.7 12.7 52.9
11.6 −1.32 17.8 35.8
−11 10.5 16.4 47.2
10.1 0.259 18.4 61.5
−0.786 9.12 14.9 53.9
0 1.5 −2.97 53.3

BlockID: H8140−C1−301_1
Cell line: MOLM−16

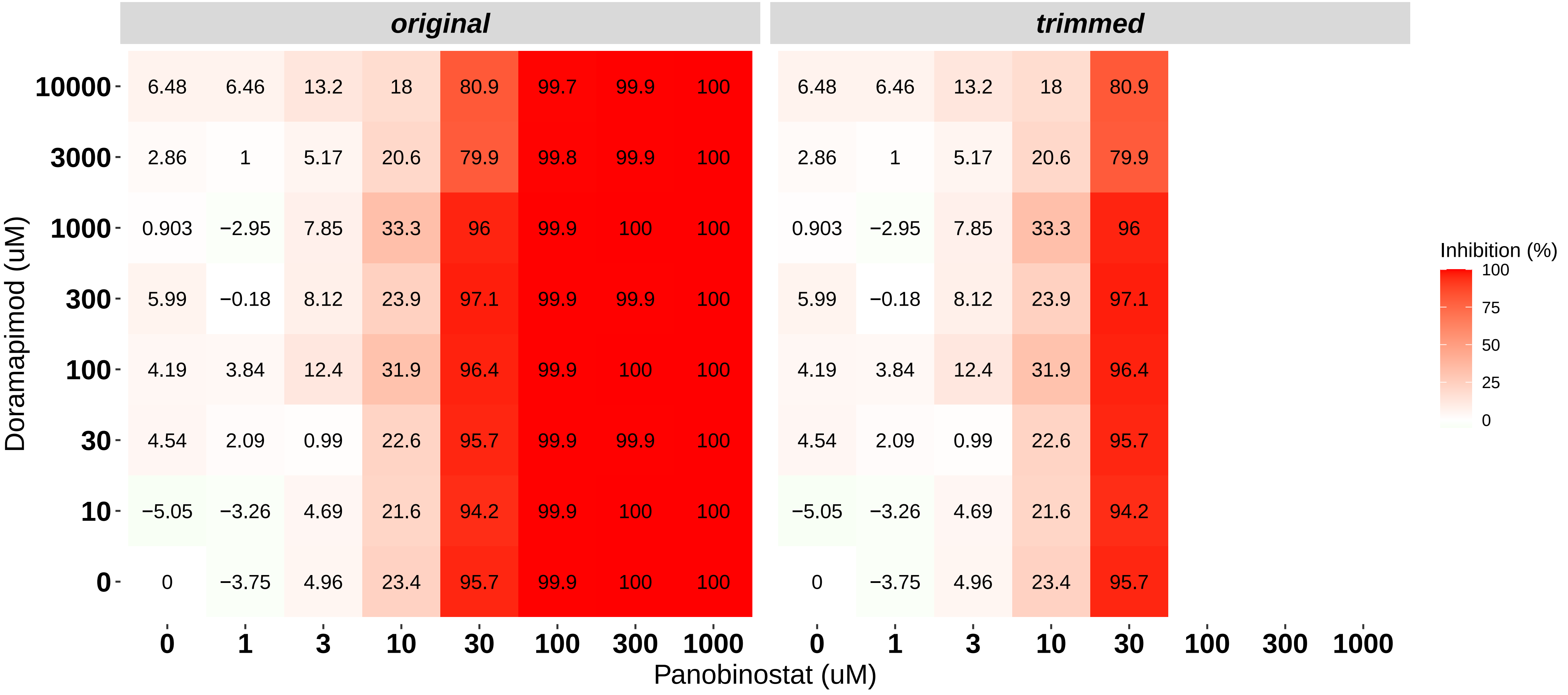

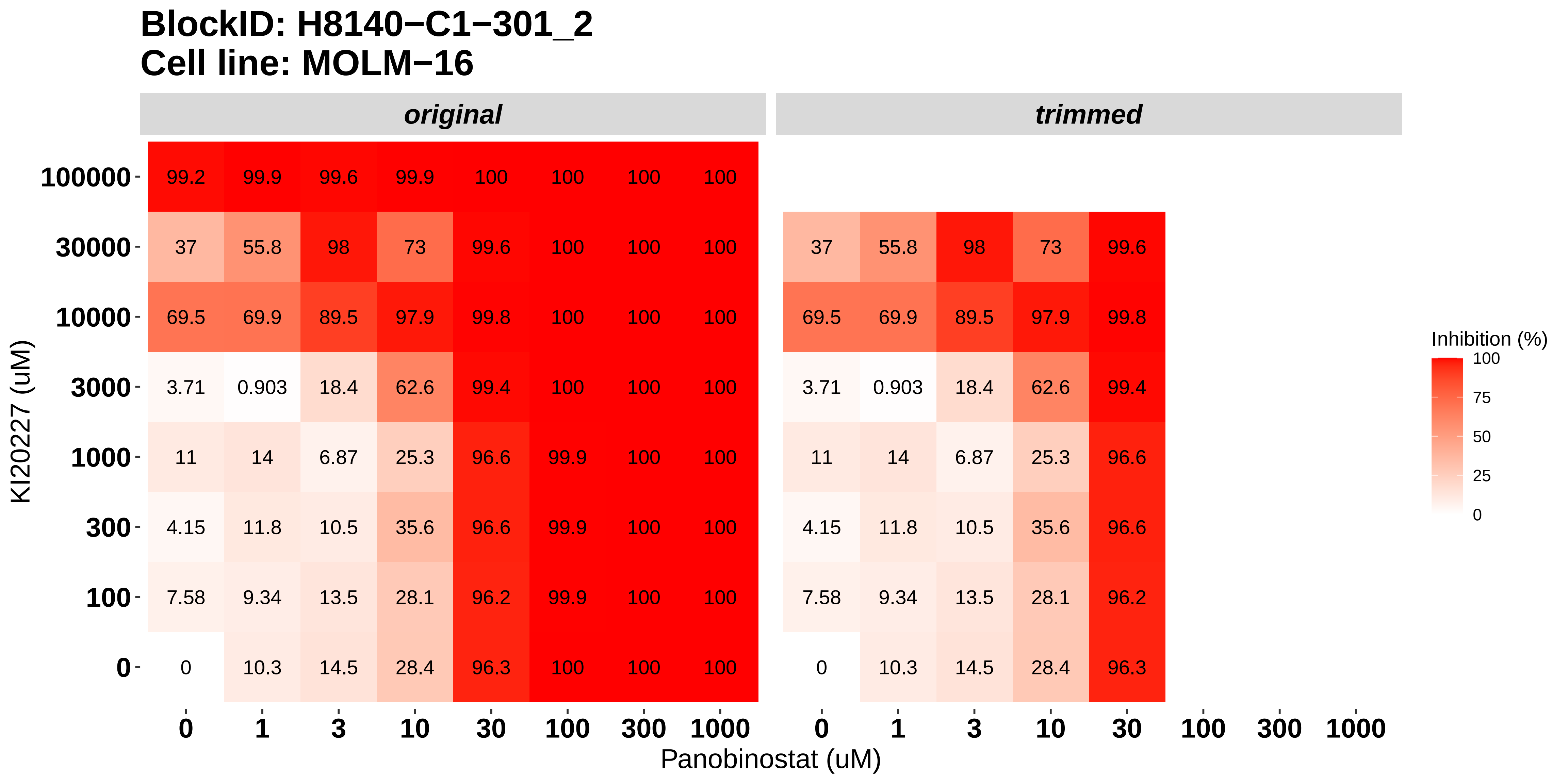

BlockID: H8140−C1−301_2
Cell line: MOLM−16
original
trimmed
KI20227 (uM)
100000
30000
10000
3000
1000
300
100
0
99.2 99.9 99.6 99.9 100 100 100 100
37 55.8 98 73 99.6 100 100 100
69.5 69.9 89.5 97.9 99.8 100 100 100
3.71 0.903 18.4 62.6 99.4 100 100 100
11 14 6.87 25.3 96.6 99.9 100 100
4.15 11.8 10.5 35.6 96.6 99.9 100 100
7.58 9.34 13.5 28.1 96.2 99.9 100 100
0 10.3 14.5 28.4 96.3 100 100 100
37 55.8 98 73 99.6
69.5 69.9 89.5 97.9 99.8
3.71 0.903 18.4 62.6 99.4
11 14 6.87 25.3 96.6
4.15 11.8 10.5 35.6 96.6
7.58 9.34 13.5 28.1 96.2
0 10.3 14.5 28.4 96.3
0 1 3 10 30 100 300 1000
Panobinostat (uM)
Inhibition (%)
100
75
50
25
0

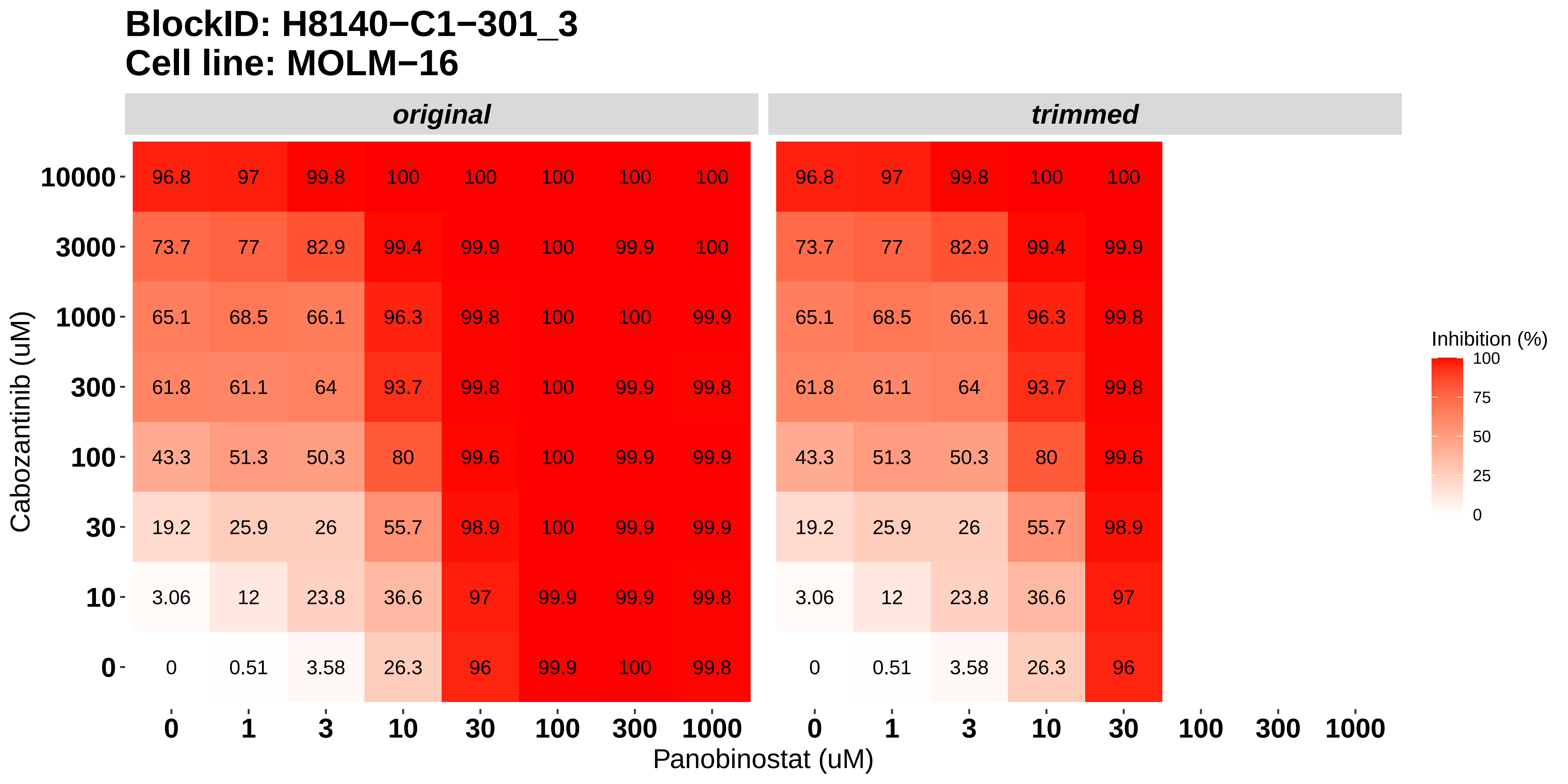
BlockID: H8140−C1−301_3
Cell line: MOLM−16
original
trimmed
Cabozantinib (uM)
Panobinostat (uM)
Inhibition (%)
100
75
50
25
0
10000
3000
1000
300
100
30
10
0
0
1
3
10
30
100
300
1000
96.8 97 99.8 100 100 100 100 100
73.7 77 82.9 99.4 99.9 100 99.9 100
65.1 68.5 66.1 96.3 99.8 100 100 99.9
61.8 61.1 64 93.7 99.8 100 99.9 99.8
43.3 51.3 50.3 80 99.6 100 99.9 99.9
19.2 25.9 26 55.7 98.9 100 99.9 99.9
3.06 12 23.8 36.6 97 99.9 99.9 99.8
0 0.51 3.58 26.3 96 99.9 100 99.8
96.8 97 99.8 100 100
73.7 77 82.9 99.4 99.9
65.1 68.5 66.1 96.3 99.8
61.8 61.1 64 93.7 99.8
43.3 51.3 50.3 80 99.6
19.2 25.9 26 55.7 98.9
3.06 12 23.8 36.6 97
0 0.51 3.58 26.3 96

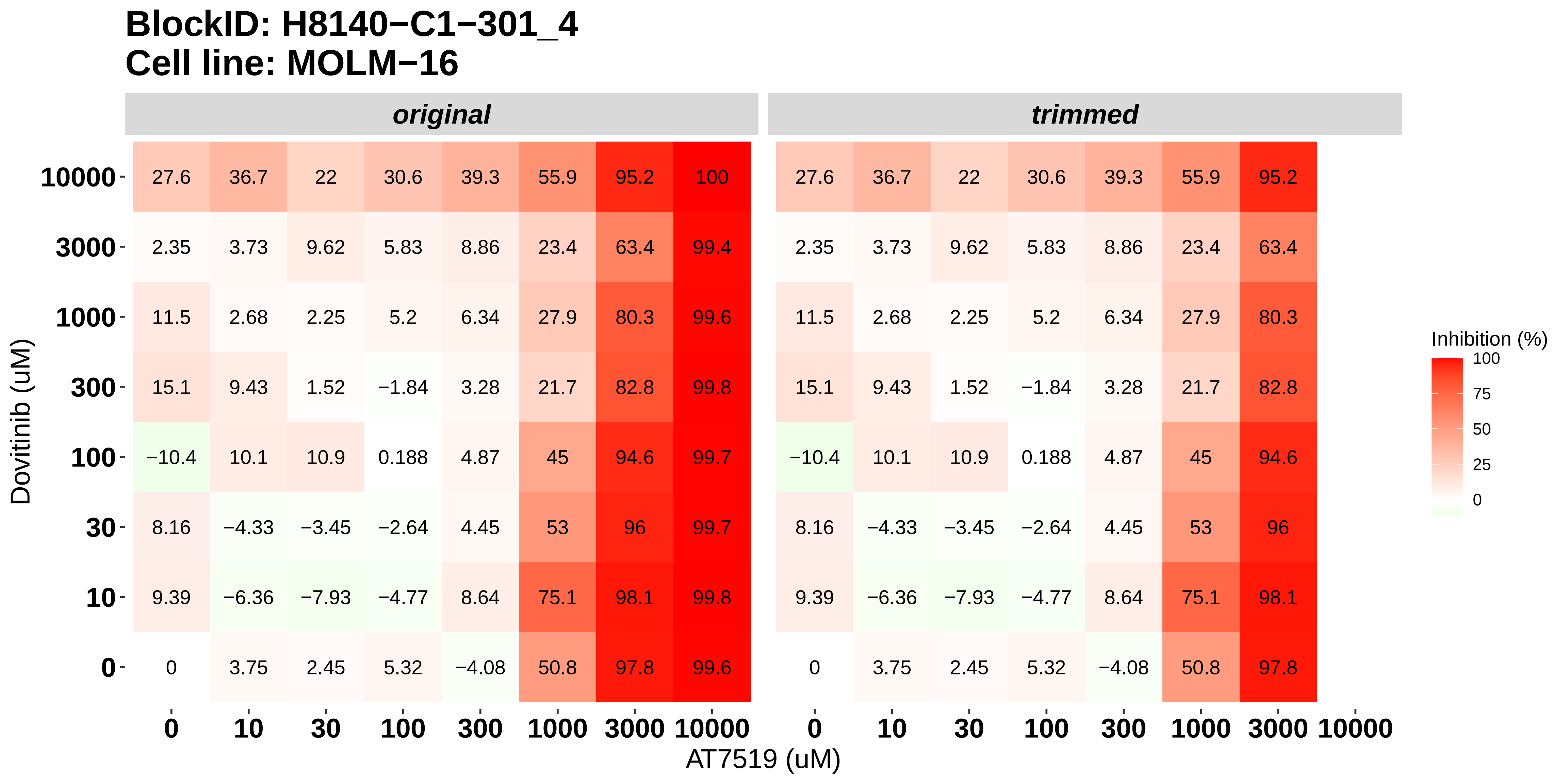

BlockID: H8140−C1−301_4
Cell line: MOLM−16
original
trimmed
Dovitinib (uM)
AT7519 (uM)
Inhibition (%)
10000
3000
1000
300
100
30
10
0
27.6 36.7 22 30.6 39.3 55.9 95.2 100
2.35 3.73 9.62 5.83 8.86 23.4 63.4 99.4
11.5 2.68 2.25 5.2 6.34 27.9 80.3 99.6
15.1 9.43 1.52 −1.84 3.28 21.7 82.8 99.8
−10.4 10.1 10.9 0.188 4.87 45 94.6 99.7
8.16 −4.33 −3.45 −2.64 4.45 53 96 99.7
9.39 −6.36 −7.93 −4.77 8.64 75.1 98.1 99.8
0 3.75 2.45 5.32 −4.08 50.8 97.8 99.6
27.6 36.7 22 30.6 39.3 55.9 95.2
2.35 3.73 9.62 5.83 8.86 23.4 63.4
11.5 2.68 2.25 5.2 6.34 27.9 80.3
15.1 9.43 1.52 −1.84 3.28 21.7 82.8
−10.4 10.1 10.9 0.188 4.87 45 94.6
8.16 −4.33 −3.45 −2.64 4.45 53 96
9.39 −6.36 −7.93 −4.77 8.64 75.1 98.1
0 3.75 2.45 5.32 −4.08 50.8 97.8
0 10 30 100 300 1000 3000 10000
0 10 30 100 300 1000 3000 10000
100
75
50
25
0

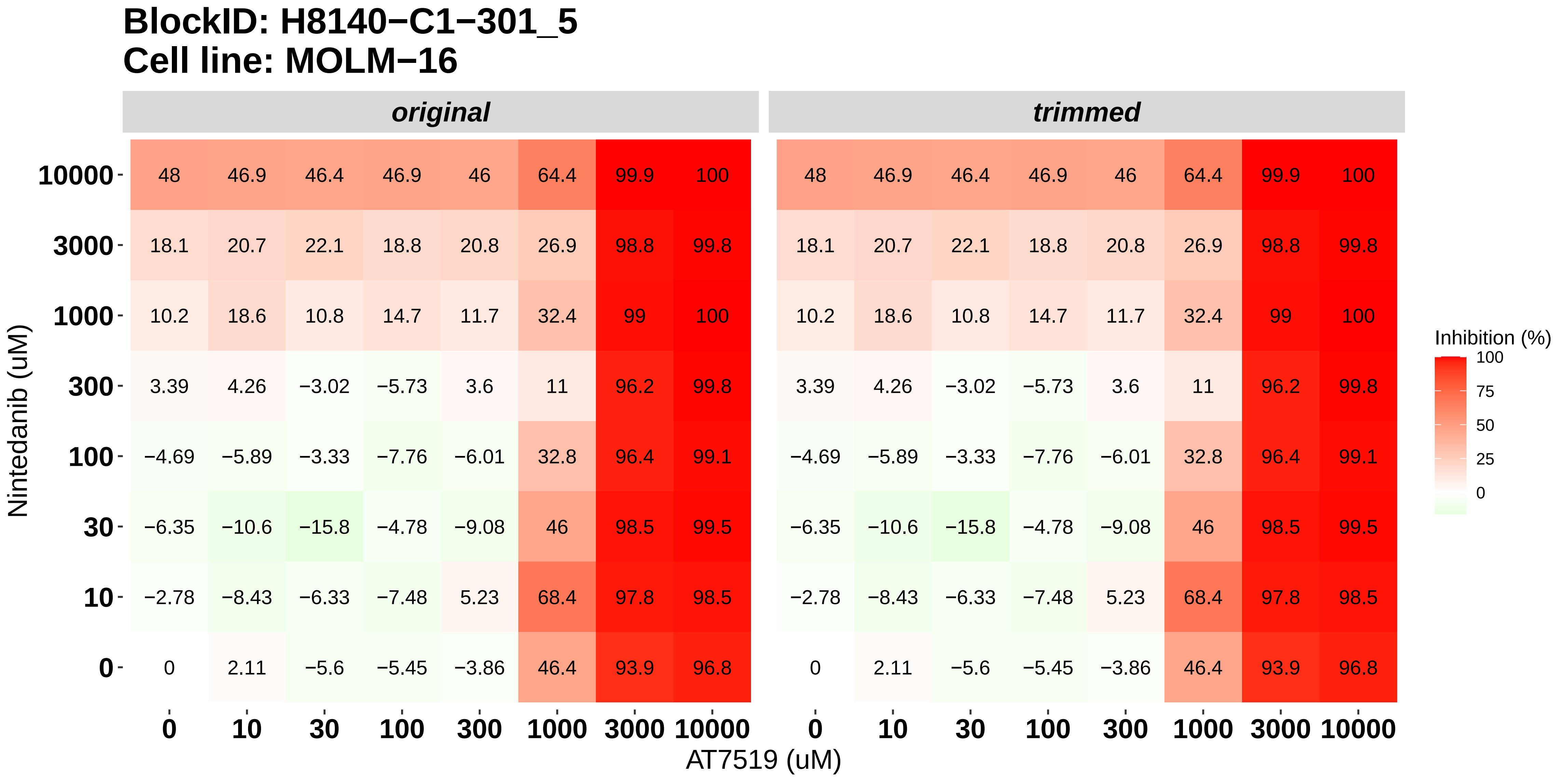
BlockID: H8140−C1−301_5
Cell line: MOLM−16
original
trimmed
Nintedanib (uM)
10000
3000
1000
300
100
30
10
0
48 46.9 46.4 46.9 46 64.4 99.9 100
18.1 20.7 22.1 18.8 20.8 26.9 98.8 99.8
10.2 18.6 10.8 14.7 11.7 32.4 99 100
3.39 4.26 −3.02 −5.73 3.6 11 96.2 99.8
−4.69 −5.89 −3.33 −7.76 −6.01 32.8 96.4 99.1
−6.35 −10.6 −15.8 −4.78 −9.08 46 98.5 99.5
−2.78 −8.43 −6.33 −7.48 5.23 68.4 97.8 98.5
0 2.11 −5.6 −5.45 −3.86 46.4 93.9 96.8
0 10 30 100 300 1000 3000 10000
AT7519 (uM)
Inhibition (%)
100
75
50
25
0

BlockID: H8140−C1−301_6
Cell line: MOLM−16

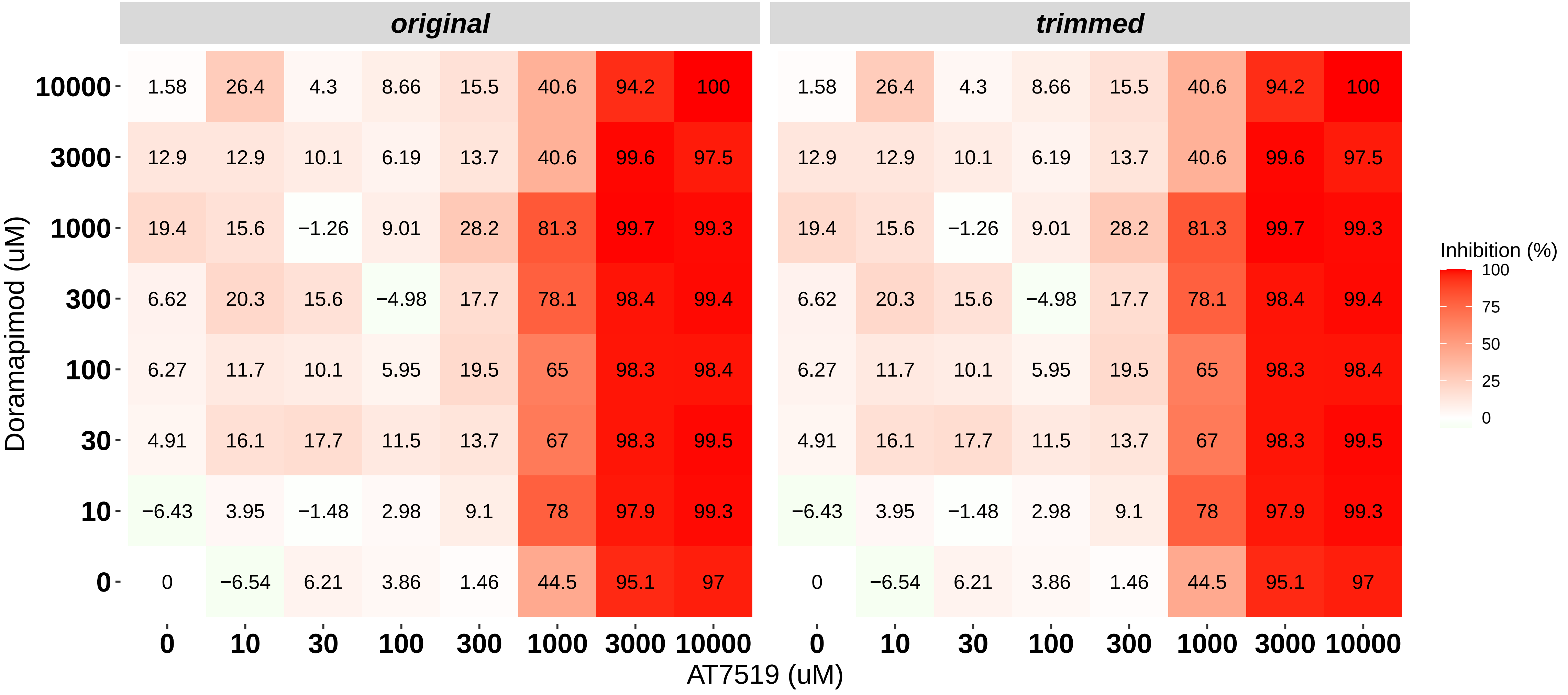

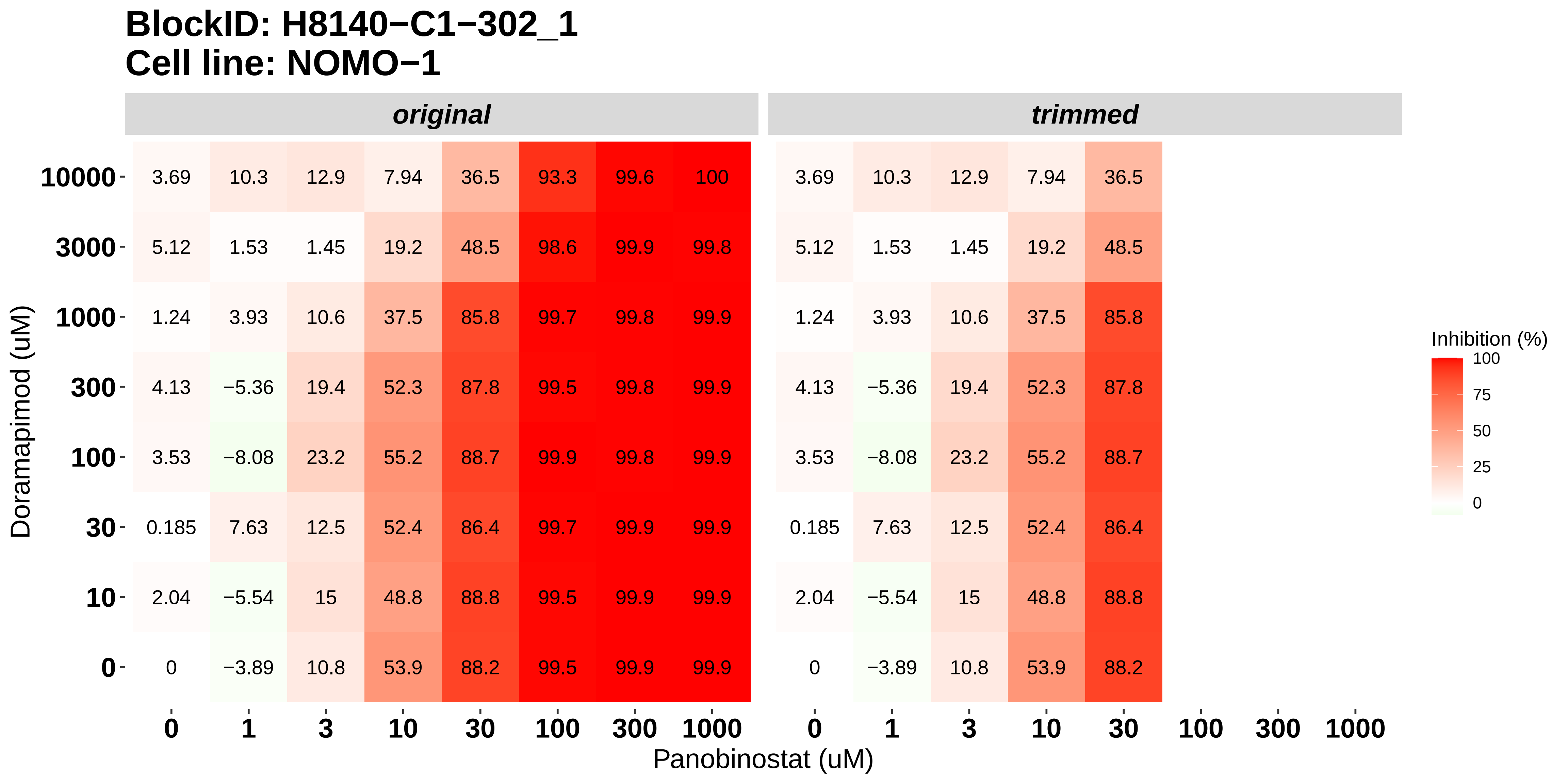
BlockID: H8140−C1−302_1
Cell line: NOMO−1
original
trimmed
Doramapimod (uM)
10000
3000
1000
300
100
30
10
0
Panobinostat (uM)
0
1
3
10
30
100
300
1000
Inhibition (%)
100
75
50
25
0
3.69 10.3 12.9 7.94 36.5 93.3 99.6 100
5.12 1.53 1.45 19.2 48.5 98.6 99.9 99.8
1.24 3.93 10.6 37.5 85.8 99.7 99.8 99.9
4.13 −5.36 19.4 52.3 87.8 99.5 99.8 99.9
3.53 −8.08 23.2 55.2 88.7 99.9 99.8 99.9
0.185 7.63 12.5 52.4 86.4 99.7 99.9 99.9
2.04 −5.54 15 48.8 88.8 99.5 99.9 99.9
0 −3.89 10.8 53.9 88.2 99.5 99.9 99.9
3.69 10.3 12.9 7.94 36.5
5.12 1.53 1.45 19.2 48.5
1.24 3.93 10.6 37.5 85.8
4.13 −5.36 19.4 52.3 87.8
3.53 −8.08 23.2 55.2 88.7
0.185 7.63 12.5 52.4 86.4
2.04 −5.54 15 48.8 88.8
0 −3.89 10.8 53.9 88.2

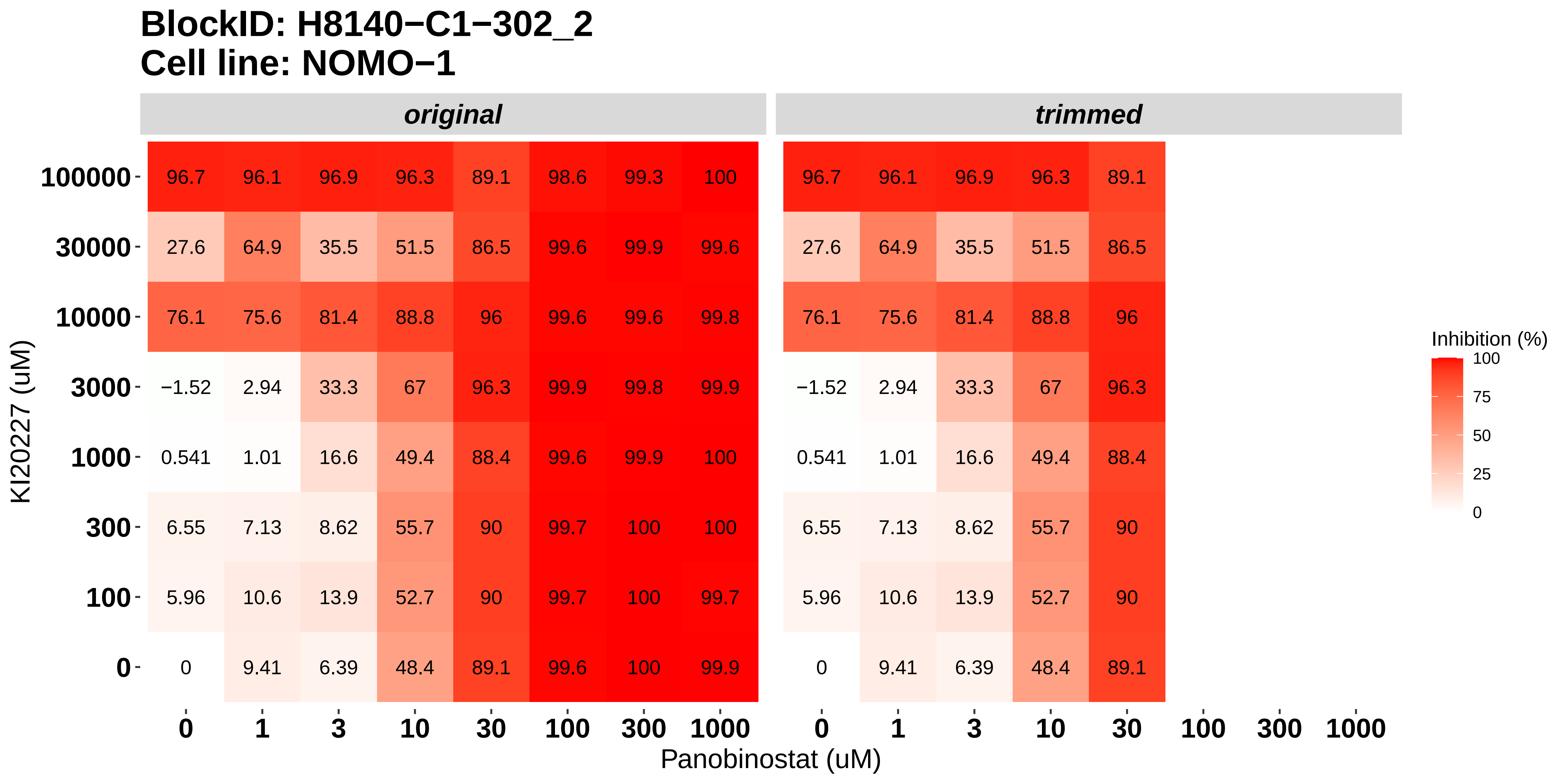

BlockID: H8140−C1−302_2
Cell line: NOMO−1
original
trimmed
KI20227 (uM)
Panobinostat (uM)
Inhibition (%)

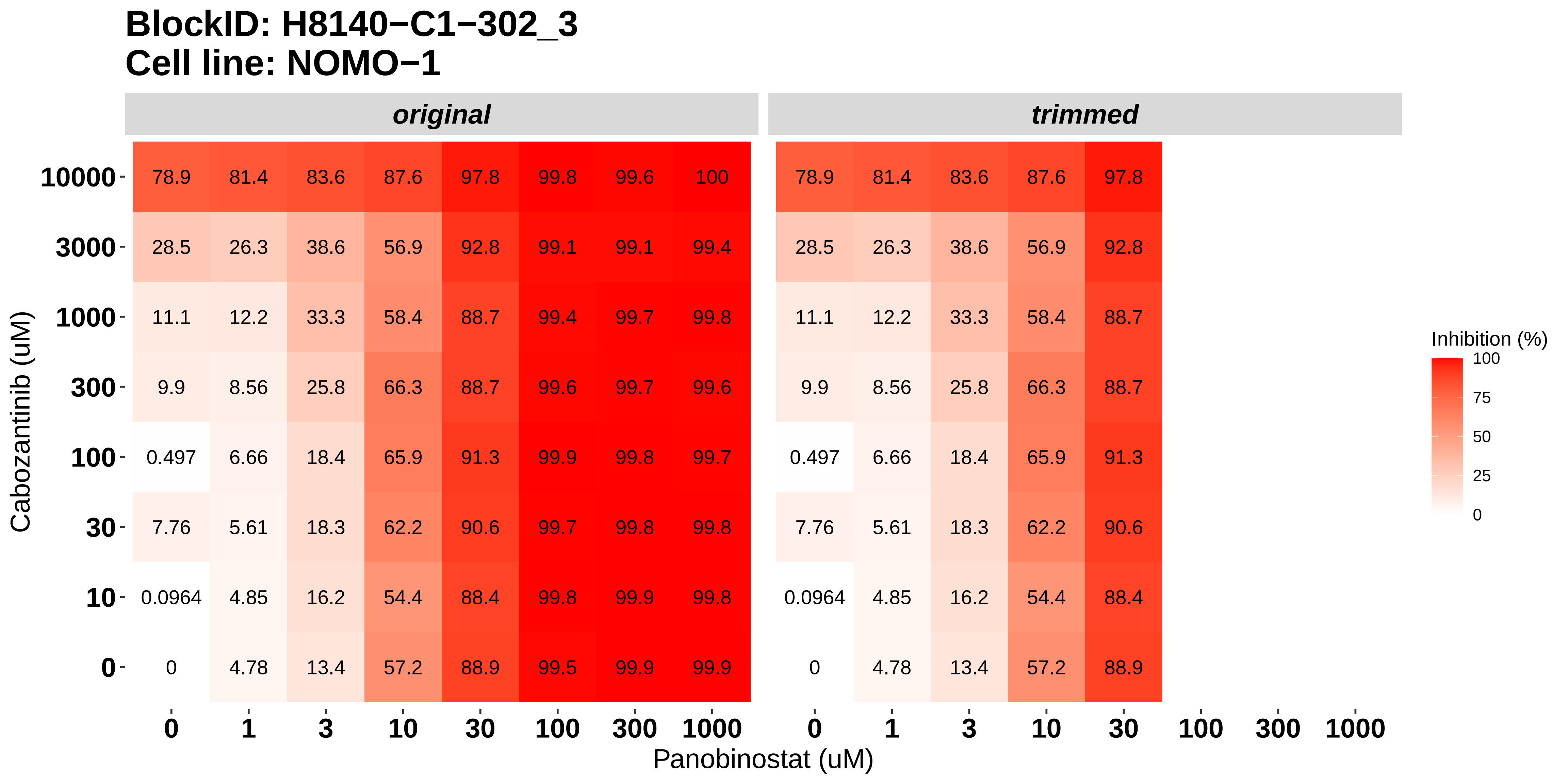
BlockID: H8140−C1−302_3
Cell line: NOMO−1
original
trimmed
Cabozantinib (uM)
Panobinostat (uM)
Inhibition (%)
100
75
50
25
0
10000
3000
1000
300
100
30
10
0
0
1
3
10
30
100
300
1000
78.9 81.4 83.6 87.6 97.8 99.8 99.6 100
28.5 26.3 38.6 56.9 92.8 99.1 99.1 99.4
11.1 12.2 33.3 58.4 88.7 99.4 99.7 99.8
9.9 8.56 25.8 66.3 88.7 99.6 99.7 99.6
0.497 6.66 18.4 65.9 91.3 99.9 99.8 99.7
7.76 5.61 18.3 62.2 90.6 99.7 99.8 99.8
0.0964 4.85 16.2 54.4 88.4 99.8 99.9 99.8
0 4.78 13.4 57.2 88.9 99.5 99.9 99.9
78.9 81.4 83.6 87.6 97.8
28.5 26.3 38.6 56.9 92.8
11.1 12.2 33.3 58.4 88.7
9.9 8.56 25.8 66.3 88.7
0.497 6.66 18.4 65.9 91.3
7.76 5.61 18.3 62.2 90.6
0.0964 4.85 16.2 54.4 88.4
0 4.78 13.4 57.2 88.9

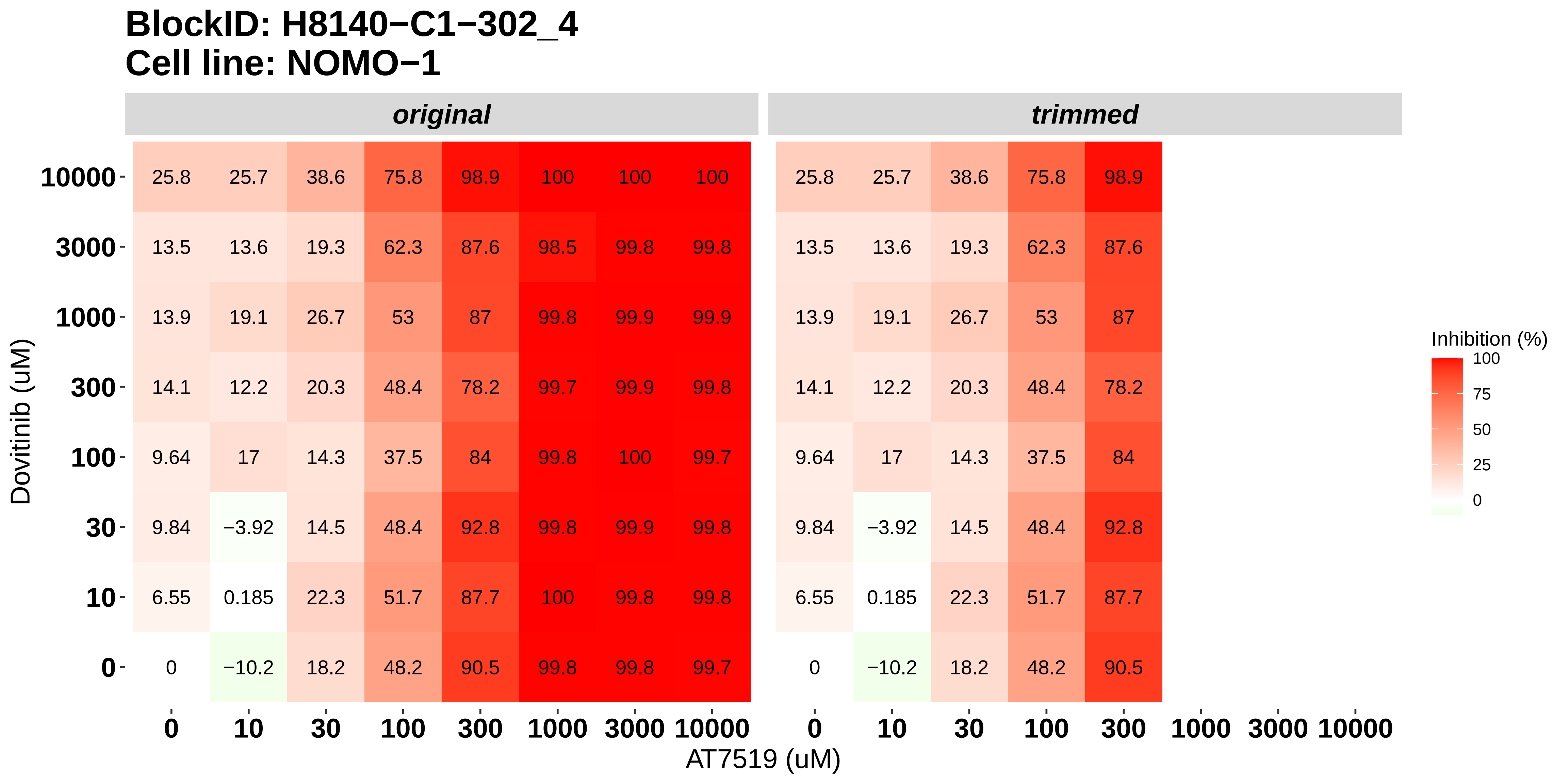
BlockID: H8140−C1−302_4
Cell line: NOMO−1
original
trimmed
Dovitinib (uM)
AT7519 (uM)
Inhibition (%)
100
75
50
25
0
10000
3000
1000
300
100
30
10
0
25.8 25.7 38.6 75.8 98.9 100 100 100
13.5 13.6 19.3 62.3 87.6 98.5 99.8 99.8
13.9 19.1 26.7 53 87 99.8 99.9 99.9
14.1 12.2 20.3 48.4 78.2 99.7 99.9 99.8
9.64 17 14.3 37.5 84 99.8 100 99.7
9.84 −3.92 14.5 48.4 92.8 99.8 99.9 99.8
6.55 0.185 22.3 51.7 87.7 100 99.8 99.8
0 −10.2 18.2 48.2 90.5 99.8 99.8 99.7
25.8 25.7 38.6 75.8 98.9
13.5 13.6 19.3 62.3 87.6
13.9 19.1 26.7 53 87
14.1 12.2 20.3 48.4 78.2
9.64 17 14.3 37.5 84
9.84 −3.92 14.5 48.4 92.8
6.55 0.185 22.3 51.7 87.7
0 −10.2 18.2 48.2 90.5
0 10 30 100 300 1000 3000 10000
0 10 30 100 300 1000 3000 10000

BlockID: H8140−C1−302_5
Cell line: NOMO−1

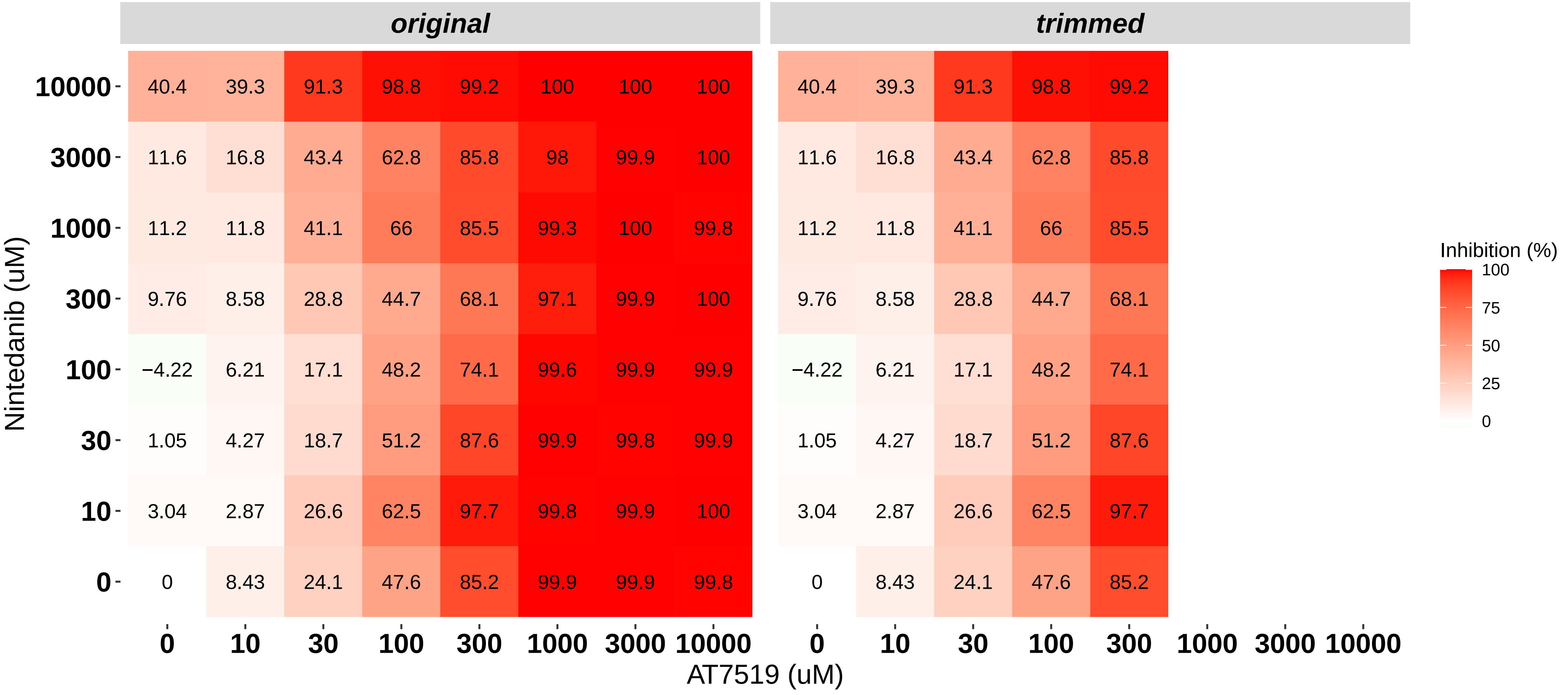

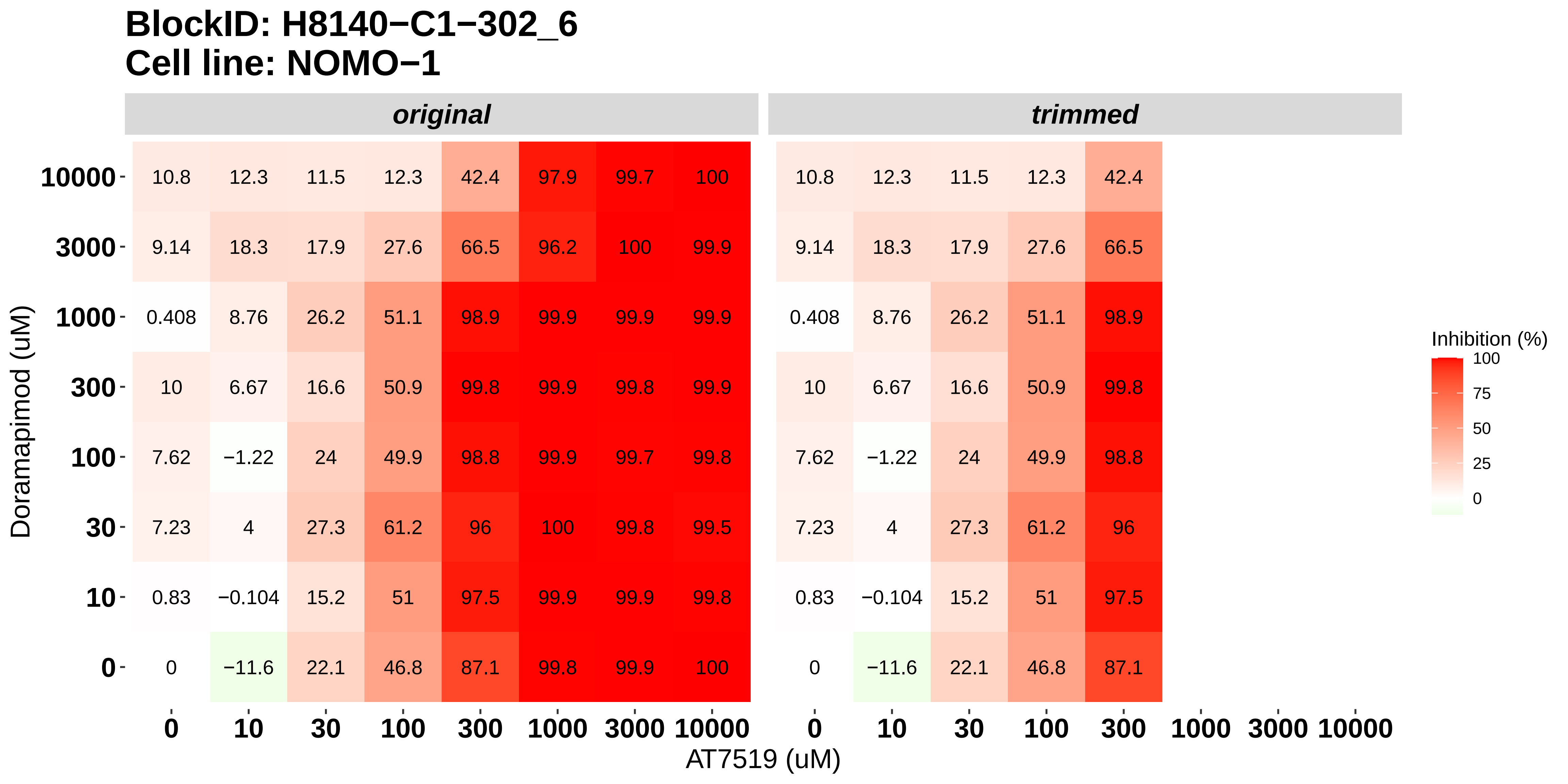

BlockID: H8140−C1−302_6
Cell line: NOMO−1
original
trimmed
Doramapimod (uM)
AT7519 (uM)
Inhibition (%)
10000
3000
1000
300
100
30
10
0
10.8 12.3 11.5 12.3 42.4 97.9 99.7 100
9.14 18.3 17.9 27.6 66.5 96.2 100 99.9
0.408 8.76 26.2 51.1 98.9 99.9 99.9 99.9
10 6.67 16.6 50.9 99.8 99.9 99.8 99.9
7.62 −1.22 24 49.9 98.8 99.9 99.7 99.8
7.23 4 27.3 61.2 96 100 99.8 99.5
0.83 −0.104 15.2 51 97.5 99.9 99.9 99.8
0 −11.6 22.1 46.8 87.1 99.8 99.9 100
10.8 12.3 11.5 12.3 42.4
9.14 18.3 17.9 27.6 66.5
0.408 8.76 26.2 51.1 98.9
10 6.67 16.6 50.9 99.8
7.62 −1.22 24 49.9 98.8
7.23 4 27.3 61.2 96
0.83 −0.104 15.2 51 97.5
0 −11.6 22.1 46.8 87.1
0 10 30 100 300 1000 3000 10000
0 10 30 100 300 1000 3000 10000
100 75 50 25 0

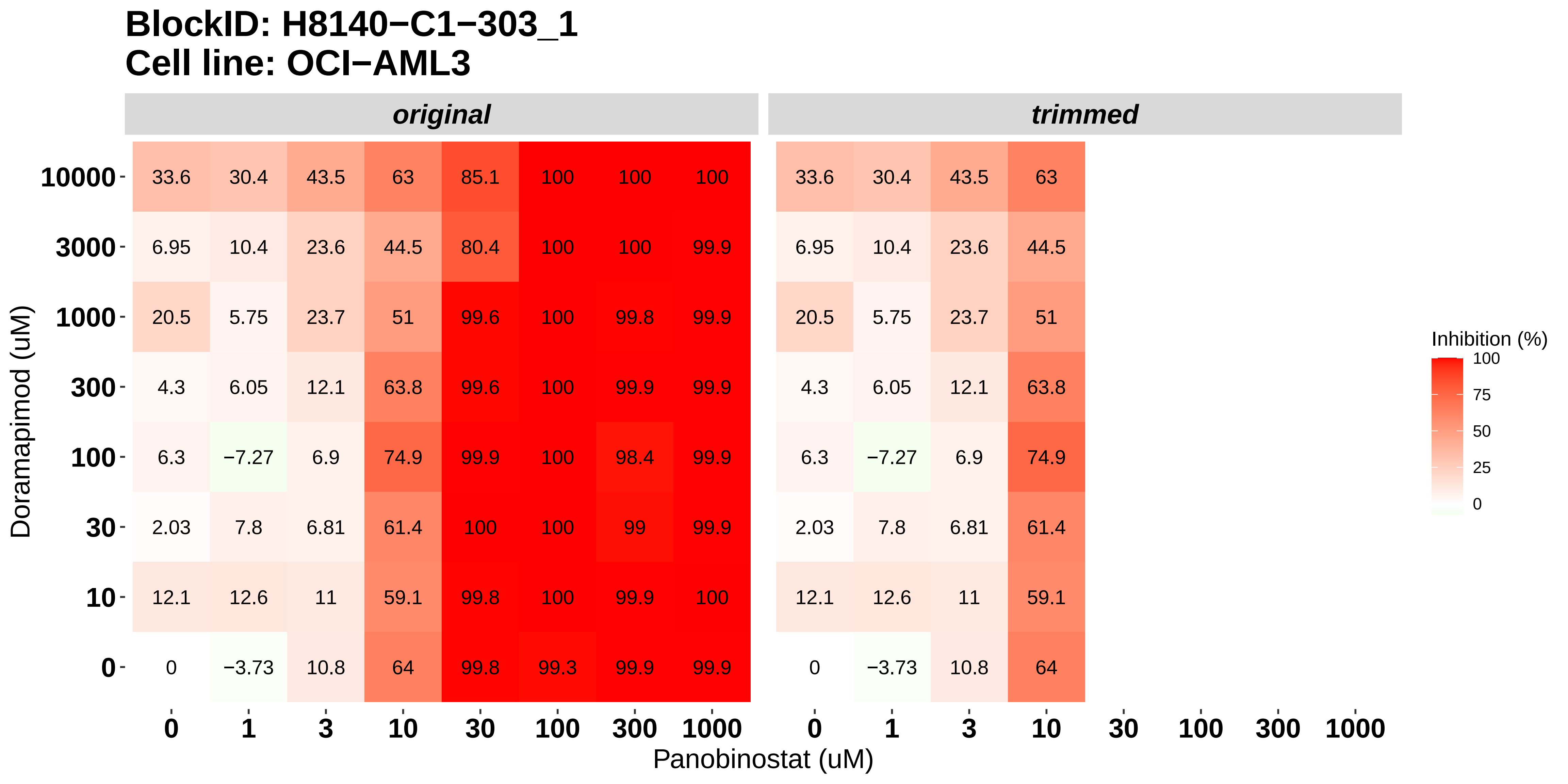

BlockID: H8140−C1−303_1
Cell line: OCI−AML3
original
trimmed
Doramapimod (uM)
Panobinostat (uM)
Inhibition (%)
10000
3000
1000
300
100
30
10
0
1
3
33.6 30.4 43.5 63 85.1 100 100 100
6.95 10.4 23.6 44.5 80.4 100 100 99.9
20.5 5.75 23.7 51 99.6 100 99.8 99.9
4.3 6.05 12.1 63.8 99.6 100 99.9 99.9
6.3 −7.27 6.9 74.9 99.9 100 98.4 99.9
2.03 7.8 6.81 61.4 100 100 99 99.9
12.1 12.6 11 59.1 99.8 100 99.9 100
0 −3.73 10.8 64 99.8 99.3 99.9 99.9
33.6 30.4 43.5 63
6.95 10.4 23.6 44.5
20.5 5.75 23.7 51
4.3 6.05 12.1 63.8
6.3 −7.27 6.9 74.9
2.03 7.8 6.81 61.4
12.1 12.6 11 59.1
0 −3.73 10.8 64

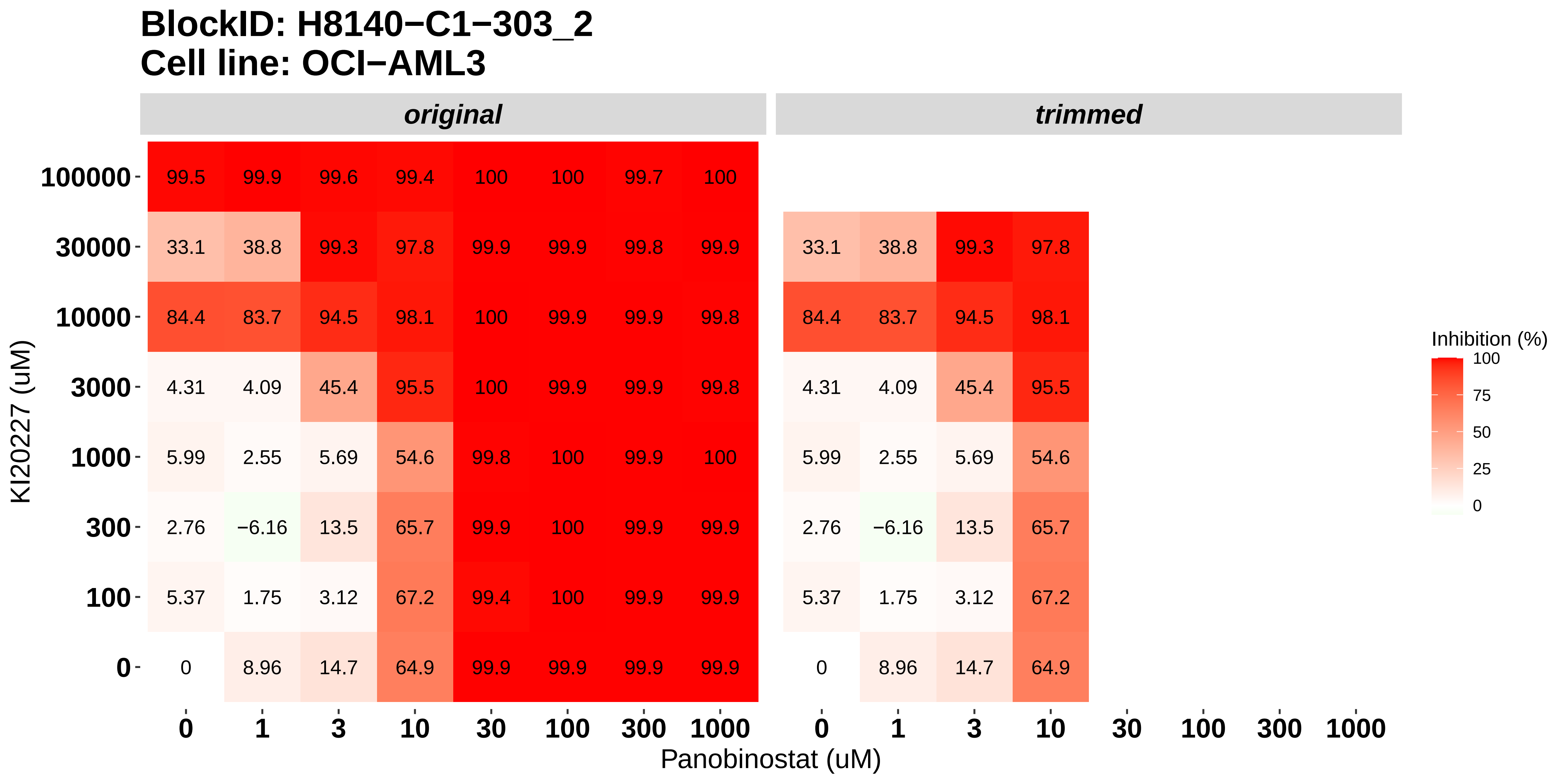

BlockID: H8140−C1−303_2
Cell line: OCI−AML3
original
trimmed
KI20227 (uM)
Panobinostat (uM)
Inhibition (%)
100000
30000
10000
3000
1000
300
100
0
0
1
3
10
30
100
300
1000
99.5 99.9 99.6 99.4 100 100 99.7 100
33.1 38.8 99.3 97.8 99.9 99.9 99.8 99.9
84.4 83.7 94.5 98.1 100 99.9 99.9 99.8
4.31 4.09 45.4 95.5 100 99.9 99.9 99.8
5.99 2.55 5.69 54.6 99.8 100 99.9 100
2.76 −6.16 13.5 65.7 99.9 100 99.9 99.9
5.37 1.75 3.12 67.2 99.4 100 99.9 99.9
0 8.96 14.7 64.9 99.9 99.9 99.9 99.9
33.1 38.8 99.3 97.8
84.4 83.7 94.5 98.1
4.31 4.09 45.4 95.5
5.99 2.55 5.69 54.6
2.76 −6.16 13.5 65.7
5.37 1.75 3.12 67.2
0 8.96 14.7 64.9
100
75
50
25
0

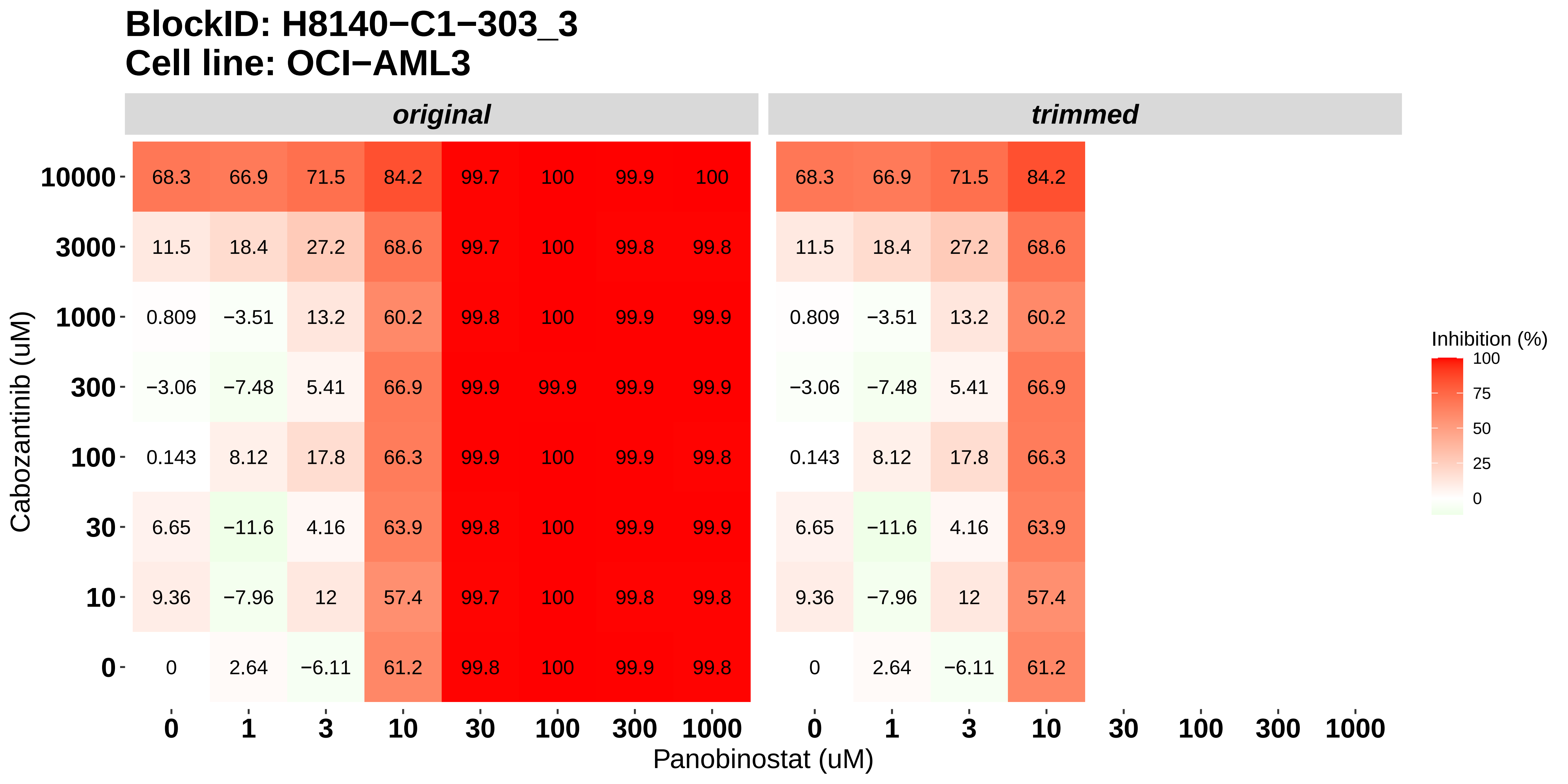
BlockID: H8140−C1−303_3
Cell line: OCI−AML3
original
trimmed
Cabozantinib (uM)
Panobinostat (uM)
Inhibition (%)
100
75
50
25
0
10000: 68.3 66.9 71.5 84.2 99.7 100 99.9 100 | 68.3 66.9 71.5 84.2
3000: 11.5 18.4 27.2 68.6 99.7 100 99.8 99.8 | 11.5 18.4 27.2 68.6
1000: 0.809 −3.51 13.2 60.2 99.8 100 99.9 99.9 | 0.809 −3.51 13.2 60.2
300: −3.06 −7.48 5.41 66.9 99.9 99.9 99.9 99.9 | −3.06 −7.48 5.41 66.9
100: 0.143 8.12 17.8 66.3 99.9 100 99.9 99.8 | 0.143 8.12 17.8 66.3
30: 6.65 −11.6 4.16 63.9 99.8 100 99.9 99.9 | 6.65 −11.6 4.16 63.9
10: 9.36 −7.96 12 57.4 99.7 100 99.8 99.8 | 9.36 −7.96 12 57.4
0: 0 2.64 −6.11 61.2 99.8 100 99.9 99.8 | 0 2.64 −6.11 61.2
0 1 3 10 30 100 300 1000 | 0 1 3 10 30 100 300 1000

BlockID: H8140−C1−303_4
Cell line: OCI−AML3

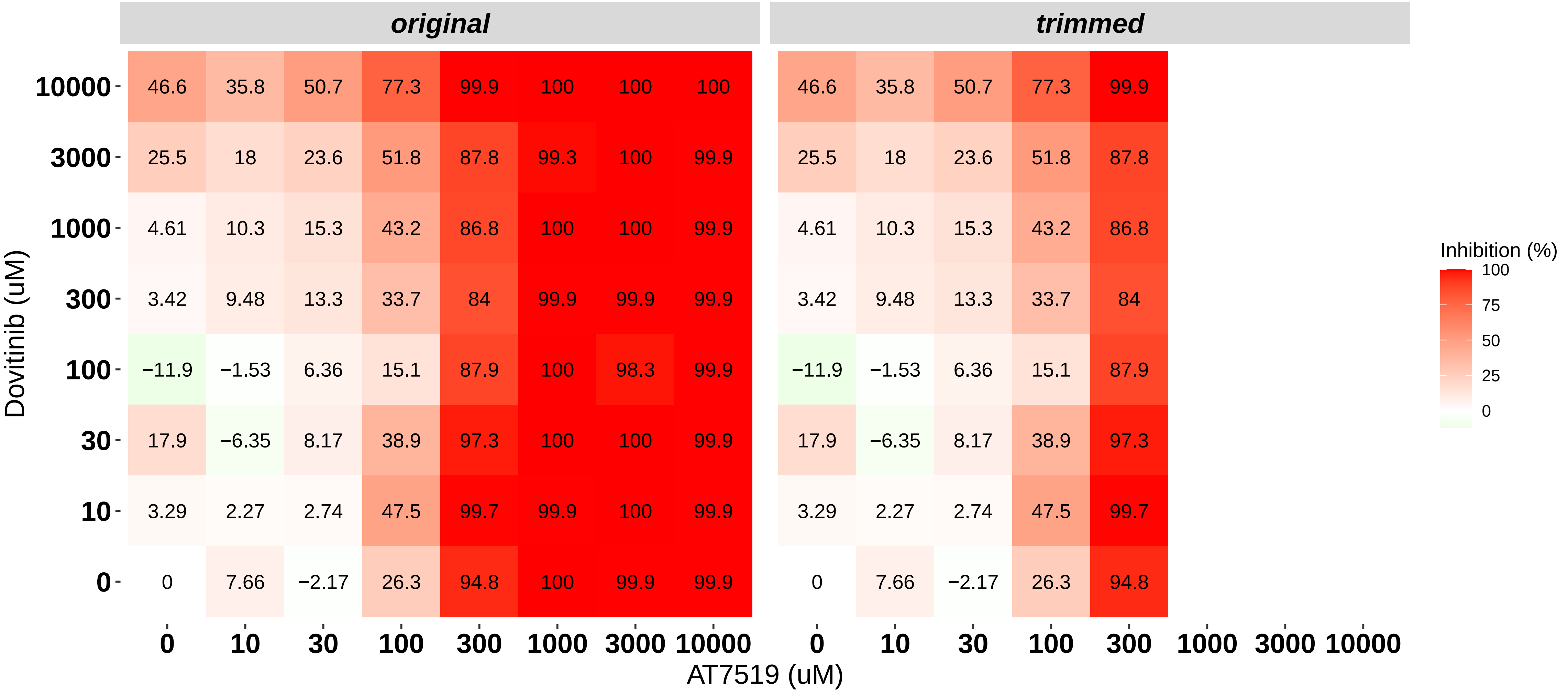

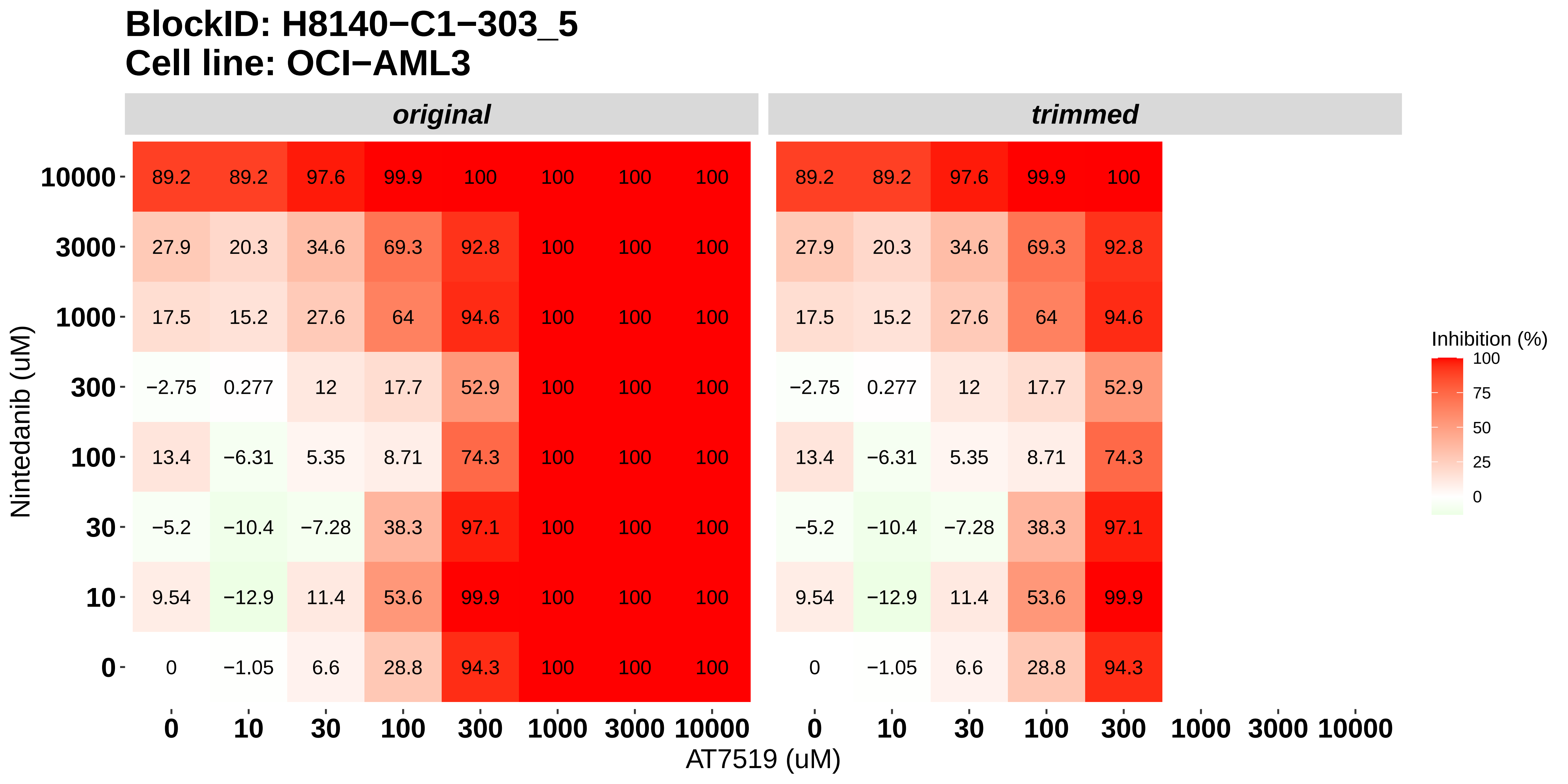
BlockID: H8140−C1−303_5
Cell line: OCI−AML3
original
trimmed
Nintedanib (uM)
AT7519 (uM)
Inhibition (%)
100
75
50
25
0
10000 89.2 89.2 97.6 99.9 100 100 100 100
3000 27.9 20.3 34.6 69.3 92.8 100 100 100
1000 17.5 15.2 27.6 64 94.6 100 100 100
300 −2.75 0.277 12 17.7 52.9 100 100 100
100 13.4 −6.31 5.35 8.71 74.3 100 100 100
30 −5.2 −10.4 −7.28 38.3 97.1 100 100 100
10 9.54 −12.9 11.4 53.6 99.9 100 100 100
0 0 −1.05 6.6 28.8 94.3 100 100 100
0 10 30 100 300 1000 3000 10000
89.2 89.2 97.6 99.9 100
27.9 20.3 34.6 69.3 92.8
17.5 15.2 27.6 64 94.6
−2.75 0.277 12 17.7 52.9
13.4 −6.31 5.35 8.71 74.3
−5.2 −10.4 −7.28 38.3 97.1
9.54 −12.9 11.4 53.6 99.9
0 −1.05 6.6 28.8 94.3
0 10 30 100 300 1000 3000 10000

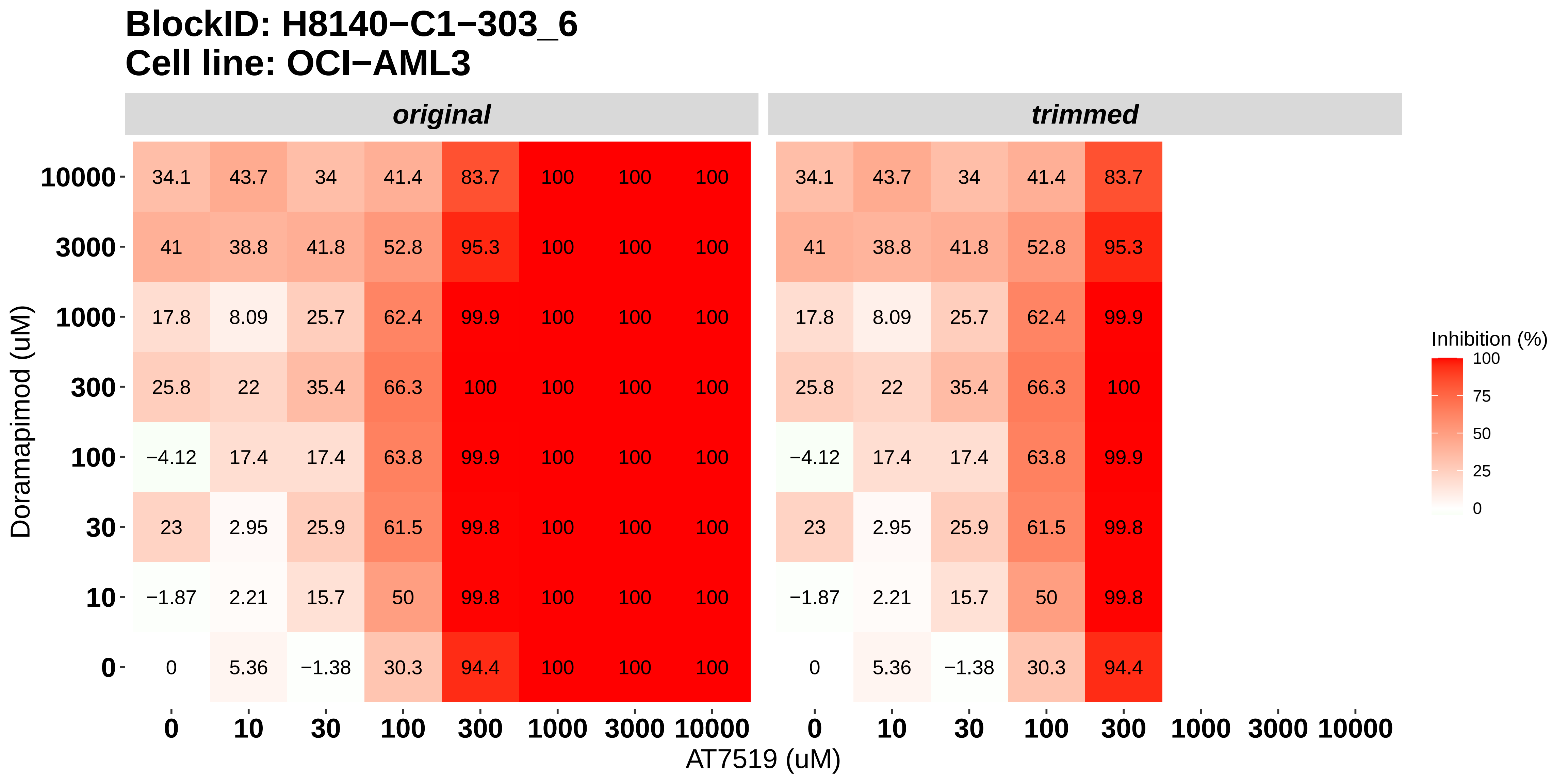
BlockID: H8140−C1−303_6
Cell line: OCI−AML3
original
trimmed
Doramapimod (uM)
10000
3000
1000
300
100
30
10
0
34.1 43.7 34 41.4 83.7 100 100 100
41 38.8 41.8 52.8 95.3 100 100 100
17.8 8.09 25.7 62.4 99.9 100 100 100
25.8 22 35.4 66.3 100 100 100 100
−4.12 17.4 17.4 63.8 99.9 100 100 100
23 2.95 25.9 61.5 99.8 100 100 100
−1.87 2.21 15.7 50 99.8 100 100 100
0 5.36 −1.38 30.3 94.4 100 100 100
34.1 43.7 34 41.4 83.7
41 38.8 41.8 52.8 95.3
17.8 8.09 25.7 62.4 99.9
25.8 22 35.4 66.3 100
−4.12 17.4 17.4 63.8 99.9
23 2.95 25.9 61.5 99.8
−1.87 2.21 15.7 50 99.8
0 5.36 −1.38 30.3 94.4
0 10 30 100 300 1000 3000 10000
0 10 30 100 300 1000 3000 10000
AT7519 (uM)
Inhibition (%)
100
75
50
25
0

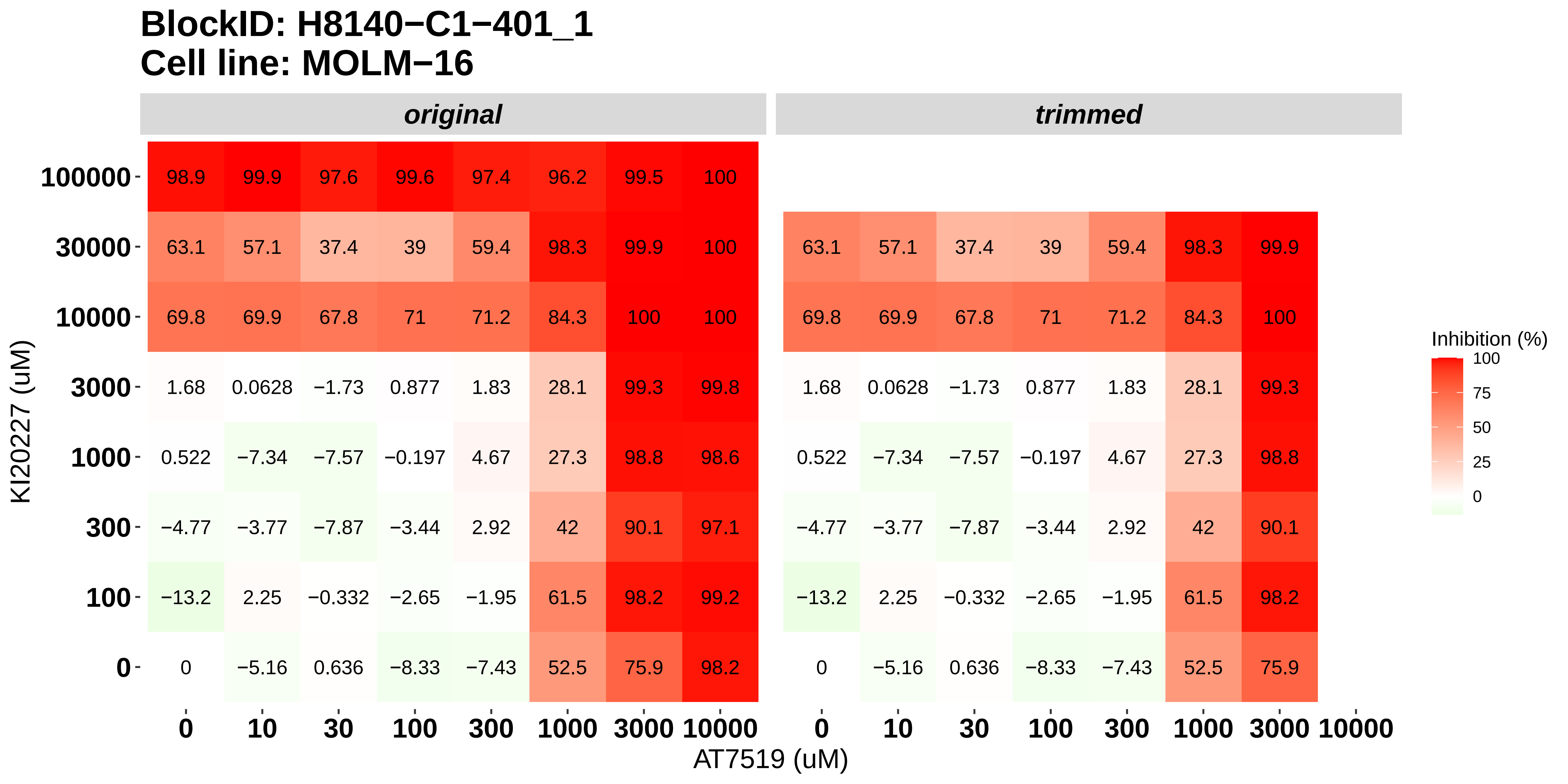

BlockID: H8140−C1−401_1
Cell line: MOLM−16
original
trimmed
KI20227 (uM)
AT7519 (uM)
Inhibition (%)
100
75
50
25
0

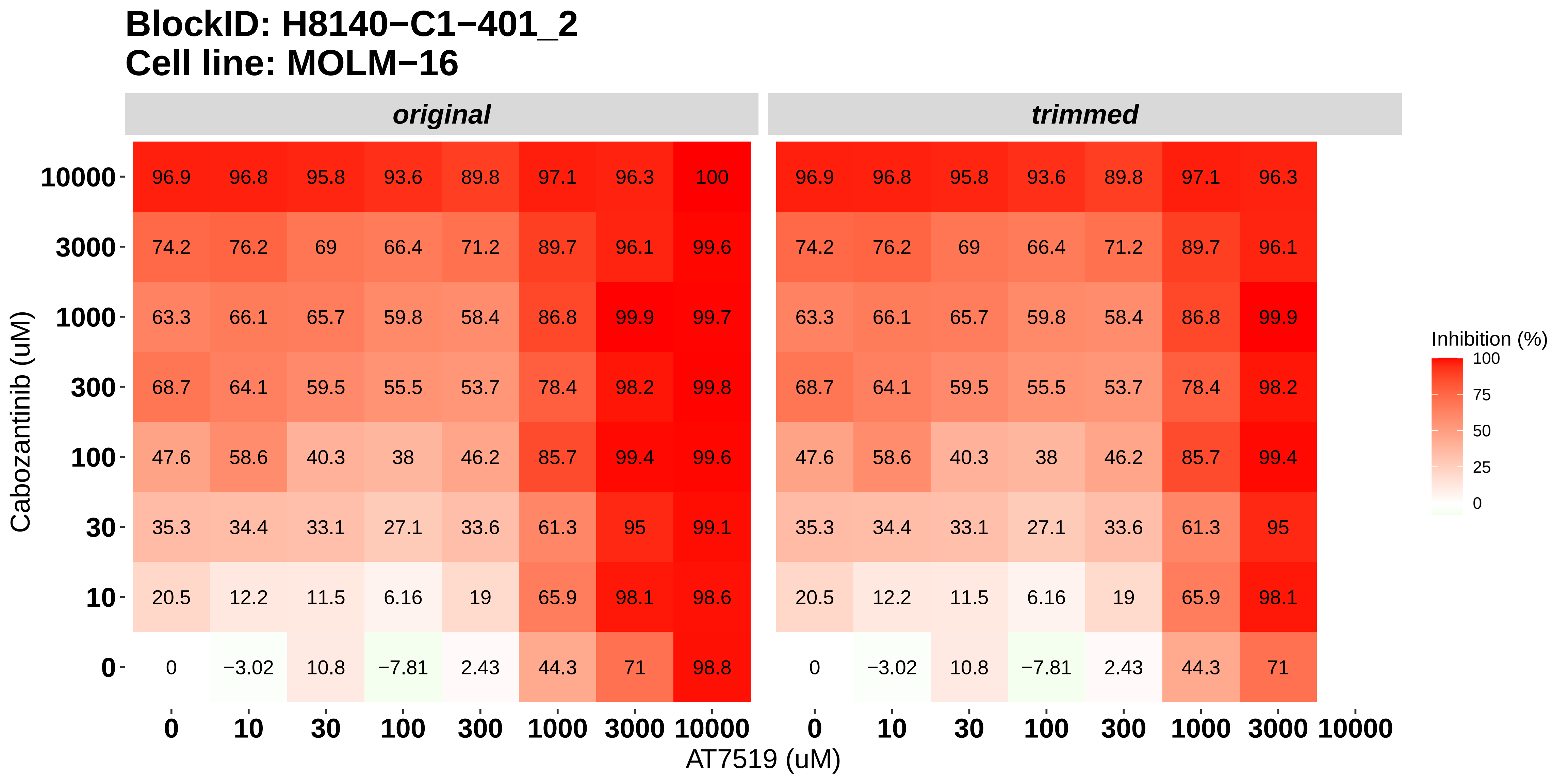
BlockID: H8140−C1−401_2
Cell line: MOLM−16
original
trimmed
Cabozantinib (uM)
AT7519 (uM)
Inhibition (%)
10000
3000
1000
300
100
30
10
0
96.9 96.8 95.8 93.6 89.8 97.1 96.3 100
74.2 76.2 69 66.4 71.2 89.7 96.1 99.6
63.3 66.1 65.7 59.8 58.4 86.8 99.9 99.7
68.7 64.1 59.5 55.5 53.7 78.4 98.2 99.8
47.6 58.6 40.3 38 46.2 85.7 99.4 99.6
35.3 34.4 33.1 27.1 33.6 61.3 95 99.1
20.5 12.2 11.5 6.16 19 65.9 98.1 98.6
0 −3.02 10.8 −7.81 2.43 44.3 71 98.8
96.9 96.8 95.8 93.6 89.8 97.1 96.3
74.2 76.2 69 66.4 71.2 89.7 96.1
63.3 66.1 65.7 59.8 58.4 86.8 99.9
68.7 64.1 59.5 55.5 53.7 78.4 98.2
47.6 58.6 40.3 38 46.2 85.7 99.4
35.3 34.4 33.1 27.1 33.6 61.3 95
20.5 12.2 11.5 6.16 19 65.9 98.1
0 −3.02 10.8 −7.81 2.43 44.3 71
0 10 30 100 300 1000 3000 10000
100 75 50 25 0

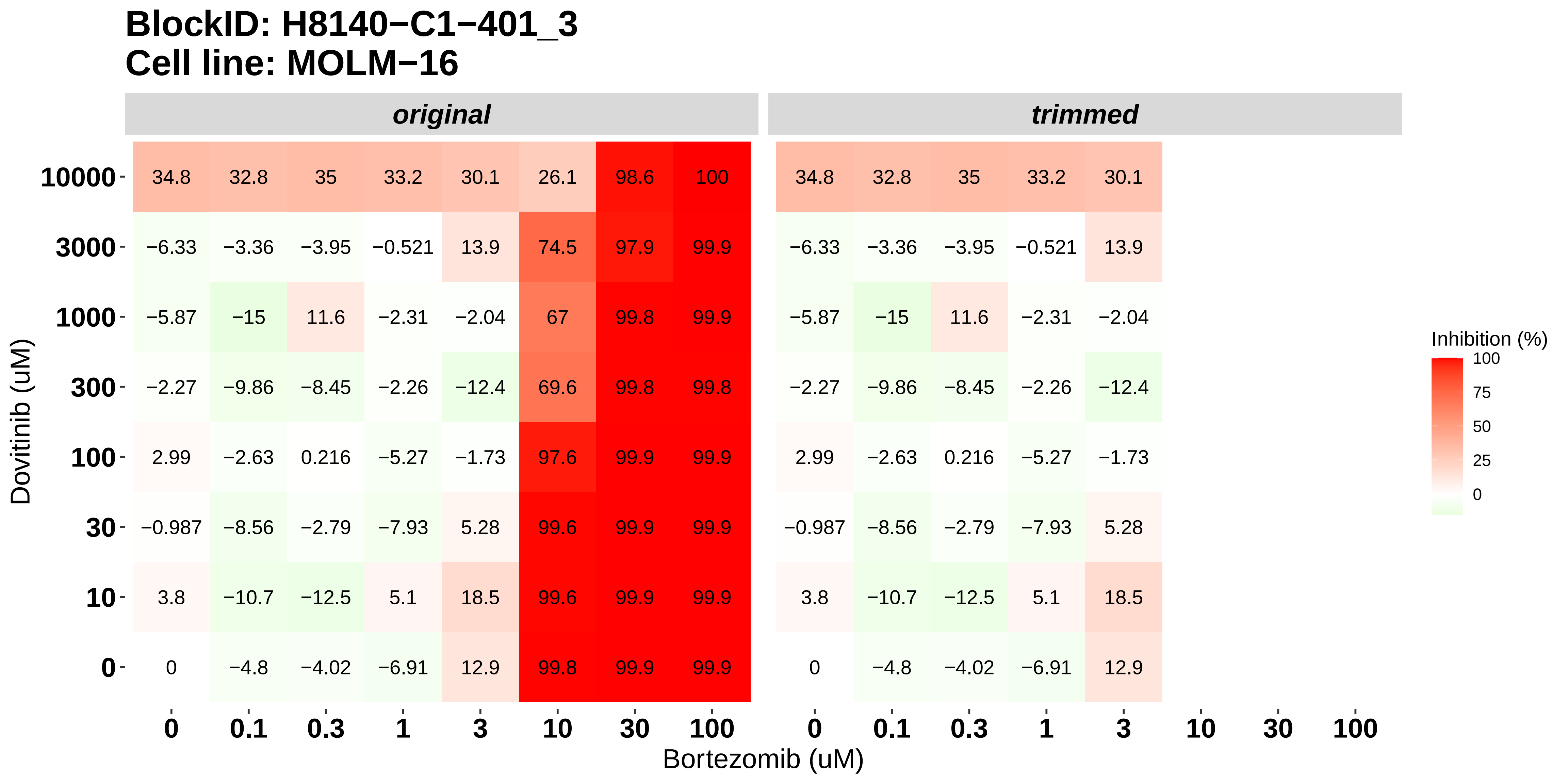

BlockID: H8140−C1−401_3
Cell line: MOLM−16
original
trimmed
Inhibition (%)
100
75
50
25
0
Dovitinib (uM)
10000
3000
1000
300
100
30
10
0
Bortezomib (uM)
0
0.1
0.3
1
3
10
30
100
34.8 32.8 35 33.2 30.1 26.1 98.6 100
−6.33 −3.36 −3.95 −0.521 13.9 74.5 97.9 99.9
−5.87 −15 11.6 −2.31 −2.04 67 99.8 99.9
−2.27 −9.86 −8.45 −2.26 −12.4 69.6 99.8 99.8
2.99 −2.63 0.216 −5.27 −1.73 97.6 99.9 99.9
−0.987 −8.56 −2.79 −7.93 5.28 99.6 99.9 99.9
3.8 −10.7 −12.5 5.1 18.5 99.6 99.9 99.9
0 −4.8 −4.02 −6.91 12.9 99.8 99.9 99.9
34.8 32.8 35 33.2 30.1
−6.33 −3.36 −3.95 −0.521 13.9
−5.87 −15 11.6 −2.31 −2.04
−2.27 −9.86 −8.45 −2.26 −12.4
2.99 −2.63 0.216 −5.27 −1.73
−0.987 −8.56 −2.79 −7.93 5.28
3.8 −10.7 −12.5 5.1 18.5
0 −4.8 −4.02 −6.91 12.9

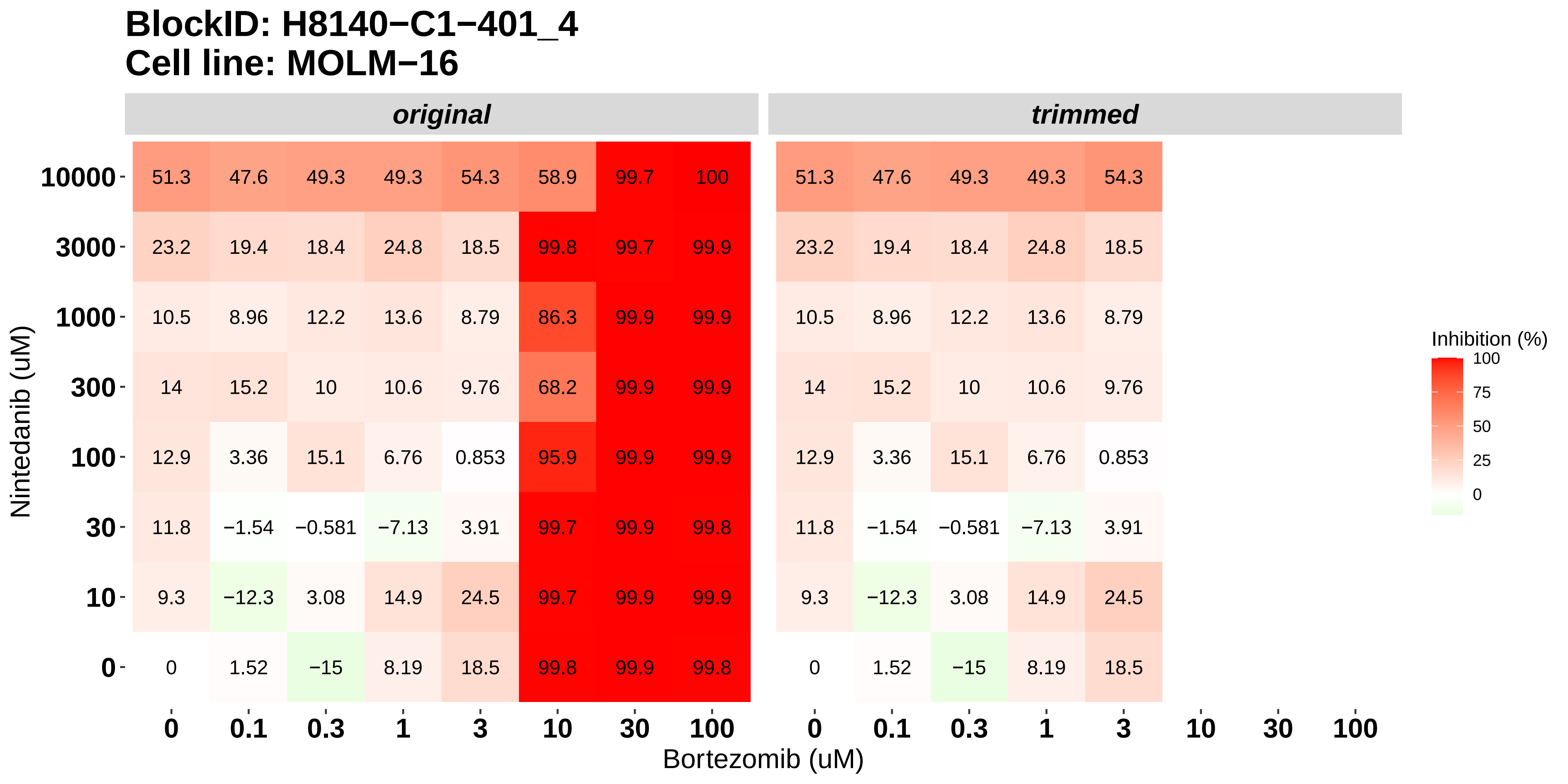
BlockID: H8140−C1−401_4
Cell line: MOLM−16
original
trimmed
Nintedanib (uM)
10000
3000
1000
300
100
30
10
0
51.3 47.6 49.3 49.3 54.3 58.9 99.7 100
23.2 19.4 18.4 24.8 18.5 99.8 99.7 99.9
10.5 8.96 12.2 13.6 8.79 86.3 99.9 99.9
14 15.2 10 10.6 9.76 68.2 99.9 99.9
12.9 3.36 15.1 6.76 0.853 95.9 99.9 99.9
11.8 −1.54 −0.581 −7.13 3.91 99.7 99.9 99.8
9.3 −12.3 3.08 14.9 24.5 99.7 99.9 99.9
0 1.52 −15 8.19 18.5 99.8 99.9 99.8
51.3 47.6 49.3 49.3 54.3
23.2 19.4 18.4 24.8 18.5
10.5 8.96 12.2 13.6 8.79
14 15.2 10 10.6 9.76
12.9 3.36 15.1 6.76 0.853
11.8 −1.54 −0.581 −7.13 3.91
9.3 −12.3 3.08 14.9 24.5
0 1.52 −15 8.19 18.5
0 0.1 0.3 1 3 10 30 100
0 0.1 0.3 1 3 10 30 100
Bortezomib (uM)
Inhibition (%)
100
75
50
25
0

BlockID: H8140−C1−401_5
Cell line: MOLM−16

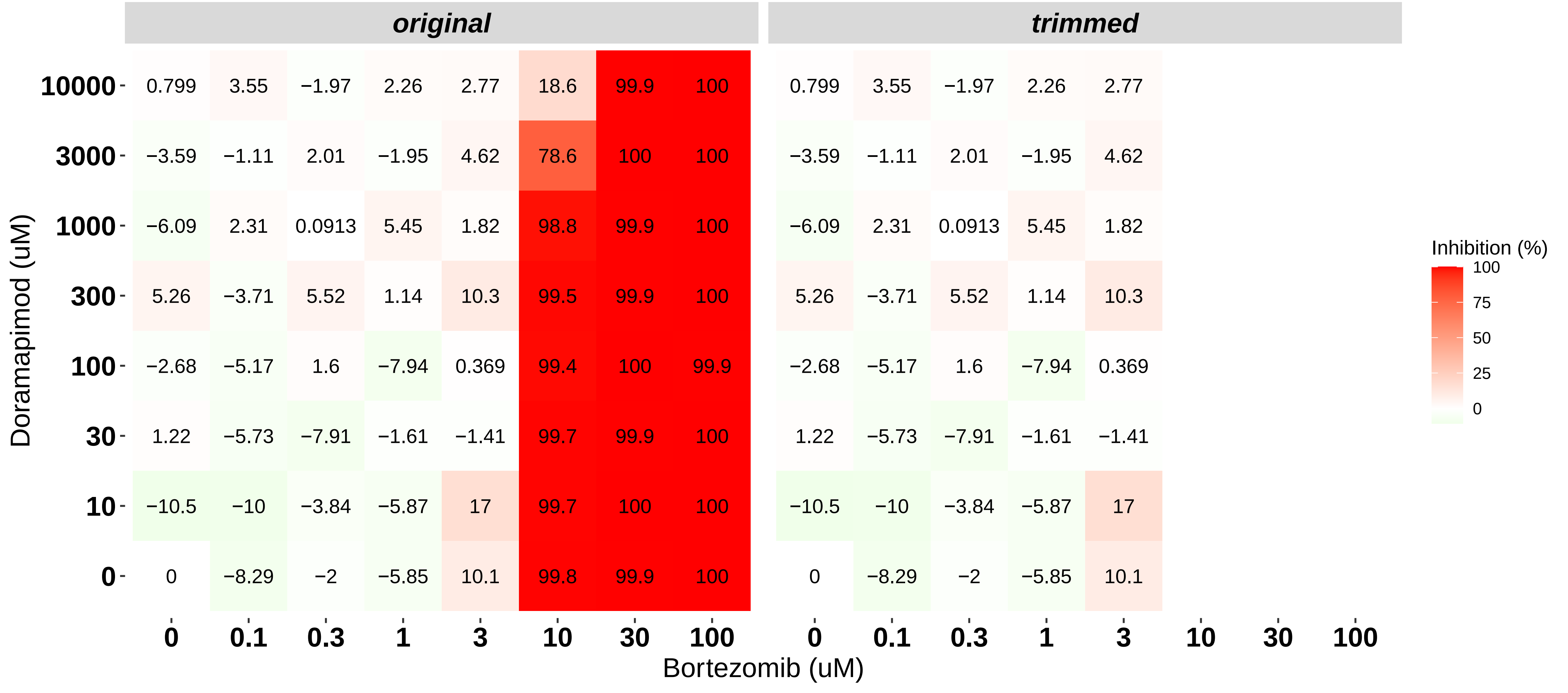

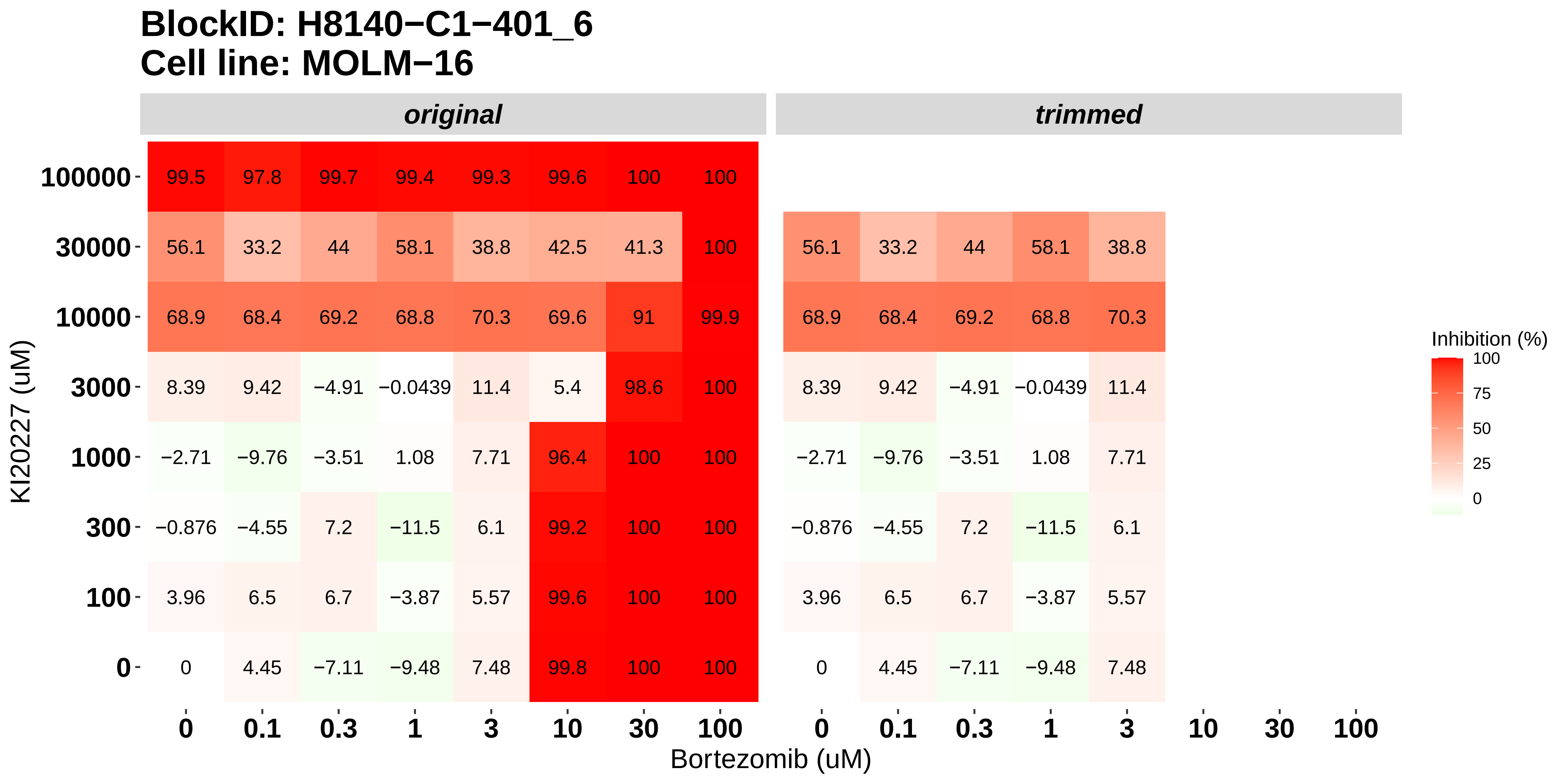
BlockID: H8140−C1−401_6
Cell line: MOLM−16
original
trimmed
KI20227 (uM)
Bortezomib (uM)
Inhibition (%)
100000
30000
10000
3000
1000
300
100
0
0.1
0.3
1
3
10
30
100
99.5 97.8 99.7 99.4 99.3 99.6 100 100
56.1 33.2 44 58.1 38.8 42.5 41.3 100
68.9 68.4 69.2 68.8 70.3 69.6 91 99.9
8.39 9.42 −4.91 −0.0439 11.4 5.4 98.6 100
−2.71 −9.76 −3.51 1.08 7.71 96.4 100 100
−0.876 −4.55 7.2 −11.5 6.1 99.2 100 100
3.96 6.5 6.7 −3.87 5.57 99.6 100 100
0 4.45 −7.11 −9.48 7.48 99.8 100 100
56.1 33.2 44 58.1 38.8
68.9 68.4 69.2 68.8 70.3
8.39 9.42 −4.91 −0.0439 11.4
−2.71 −9.76 −3.51 1.08 7.71
−0.876 −4.55 7.2 −11.5 6.1
3.96 6.5 6.7 −3.87 5.57
0 4.45 −7.11 −9.48 7.48
75
50
25

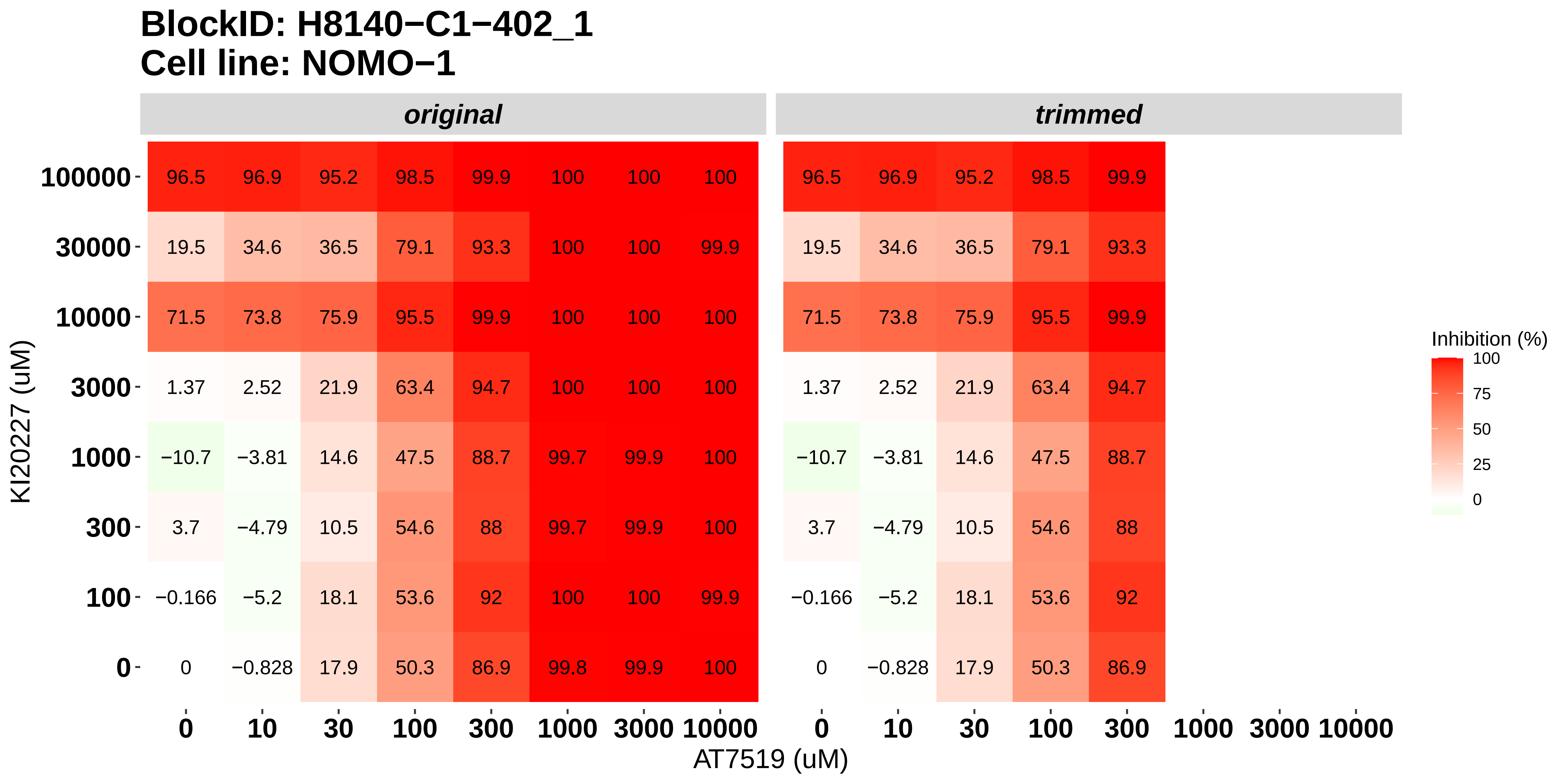
BlockID: H8140−C1−402_1
Cell line: NOMO−1
original
trimmed
KI20227 (uM)
AT7519 (uM)
Inhibition (%)
100
75
50
25
0
100000: 96.5 96.9 95.2 98.5 99.9 100 100 100 | 96.5 96.9 95.2 98.5 99.9
30000: 19.5 34.6 36.5 79.1 93.3 100 100 99.9 | 19.5 34.6 36.5 79.1 93.3
10000: 71.5 73.8 75.9 95.5 99.9 100 100 100 | 71.5 73.8 75.9 95.5 99.9
3000: 1.37 2.52 21.9 63.4 94.7 100 100 100 | 1.37 2.52 21.9 63.4 94.7
1000: −10.7 −3.81 14.6 47.5 88.7 99.7 99.9 100 | −10.7 −3.81 14.6 47.5 88.7
300: 3.7 −4.79 10.5 54.6 88 99.7 99.9 100 | 3.7 −4.79 10.5 54.6 88
100: −0.166 −5.2 18.1 53.6 92 100 100 99.9 | −0.166 −5.2 18.1 53.6 92
0: 0 −0.828 17.9 50.3 86.9 99.8 99.9 100 | 0 −0.828 17.9 50.3 86.9
0 10 30 100 300 1000 3000 10000
0 10 30 100 300 1000 3000 10000

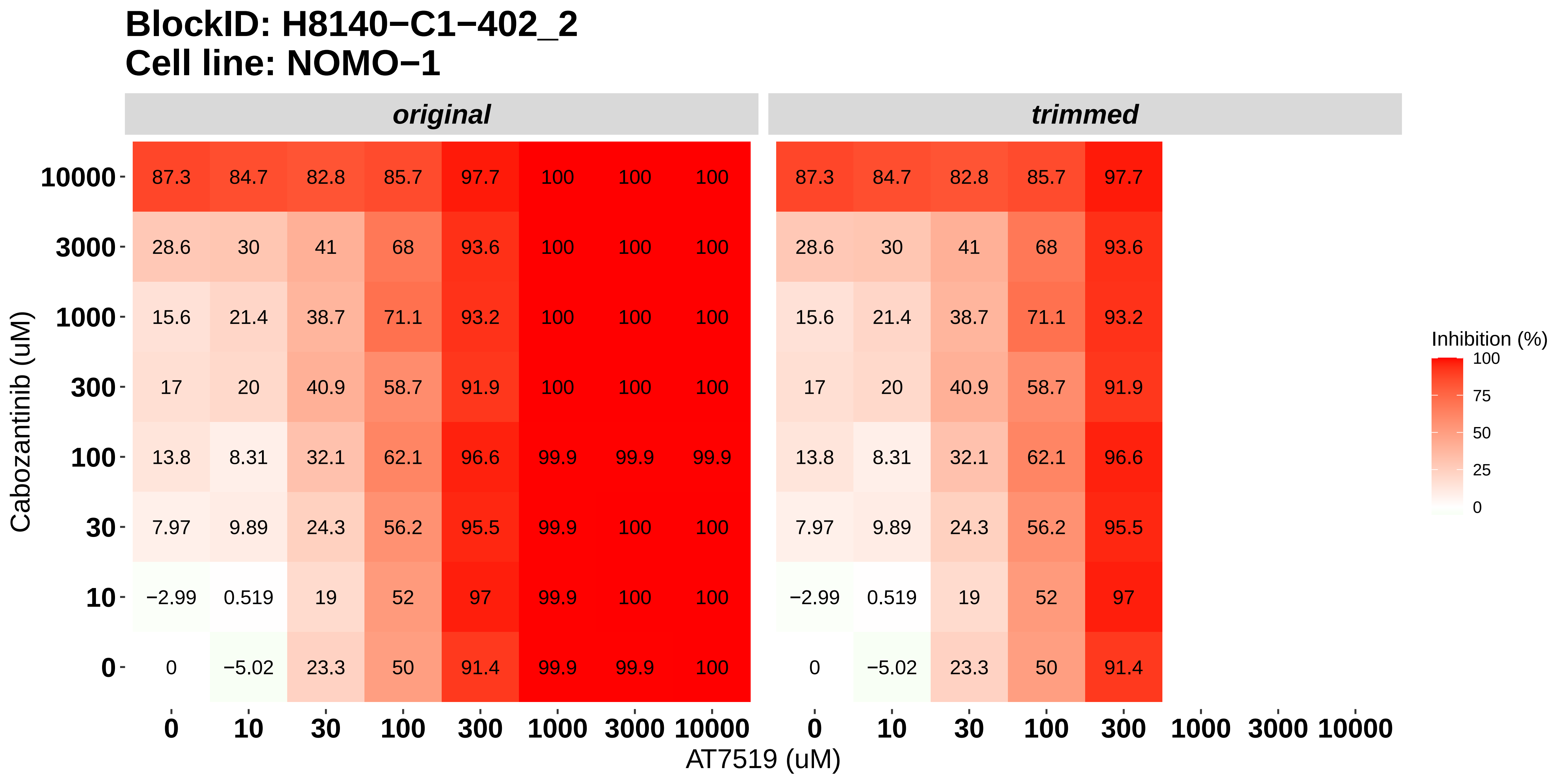

BlockID: H8140−C1−402_2
Cell line: NOMO−1
original
trimmed
Cabozantinib (uM)
AT7519 (uM)
Inhibition (%)

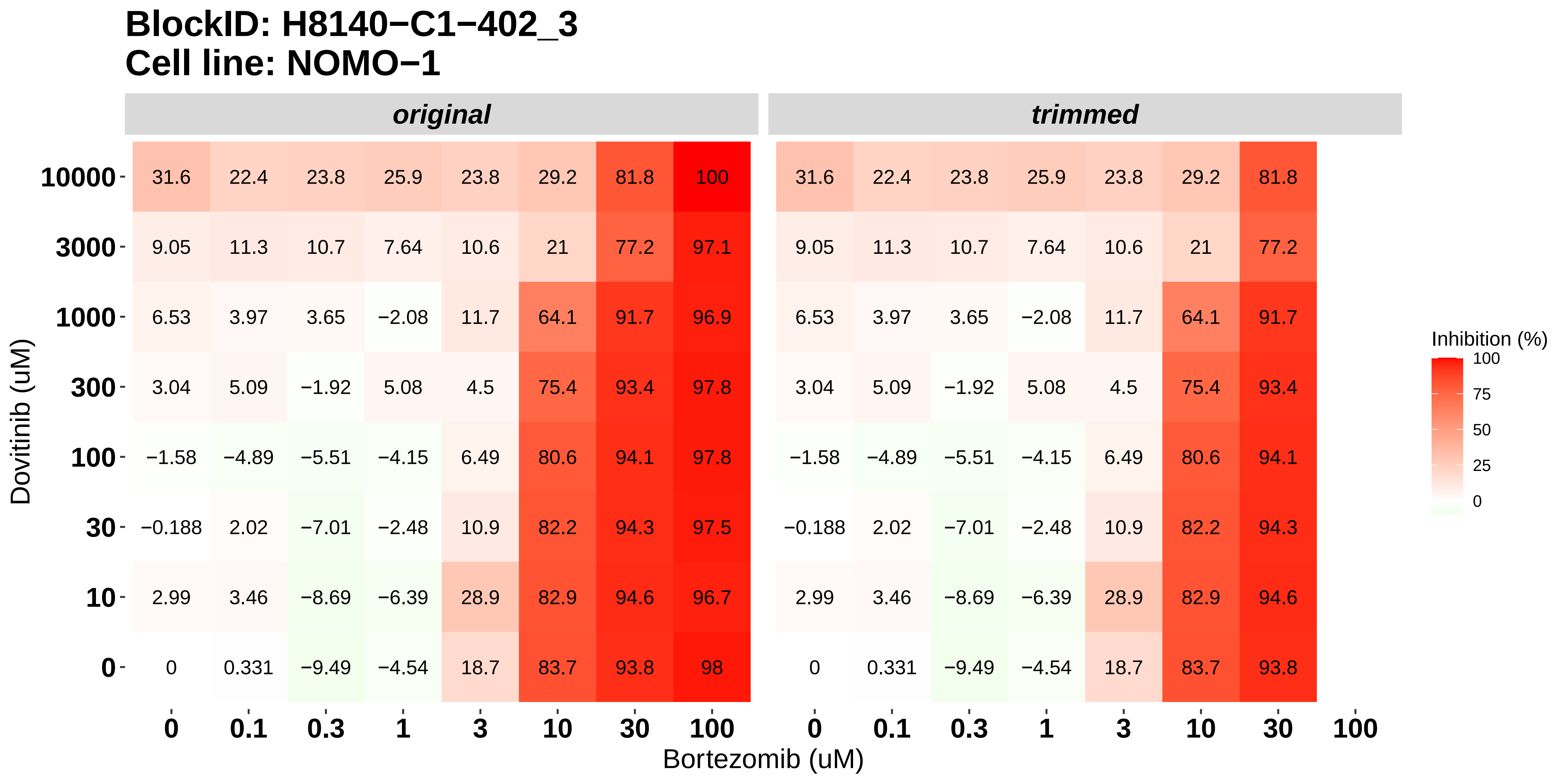
BlockID: H8140−C1−402_3
Cell line: NOMO−1
original
trimmed
Dovitinib (uM)
10000
3000
1000
300
100
30
10
0
Bortezomib (uM)
0 0.1 0.3 1 3 10 30 100
0 0.1 0.3 1 3 10 30 100
31.6 22.4 23.8 25.9 23.8 29.2 81.8 100
9.05 11.3 10.7 7.64 10.6 21 77.2 97.1
6.53 3.97 3.65 −2.08 11.7 64.1 91.7 96.9
3.04 5.09 −1.92 5.08 4.5 75.4 93.4 97.8
−1.58 −4.89 −5.51 −4.15 6.49 80.6 94.1 97.8
−0.188 2.02 −7.01 −2.48 10.9 82.2 94.3 97.5
2.99 3.46 −8.69 −6.39 28.9 82.9 94.6 96.7
0 0.331 −9.49 −4.54 18.7 83.7 93.8 98
31.6 22.4 23.8 25.9 23.8 29.2 81.8
9.05 11.3 10.7 7.64 10.6 21 77.2
6.53 3.97 3.65 −2.08 11.7 64.1 91.7
3.04 5.09 −1.92 5.08 4.5 75.4 93.4
−1.58 −4.89 −5.51 −4.15 6.49 80.6 94.1
−0.188 2.02 −7.01 −2.48 10.9 82.2 94.3
2.99 3.46 −8.69 −6.39 28.9 82.9 94.6
0 0.331 −9.49 −4.54 18.7 83.7 93.8
Inhibition (%)
100
75
50
25
0

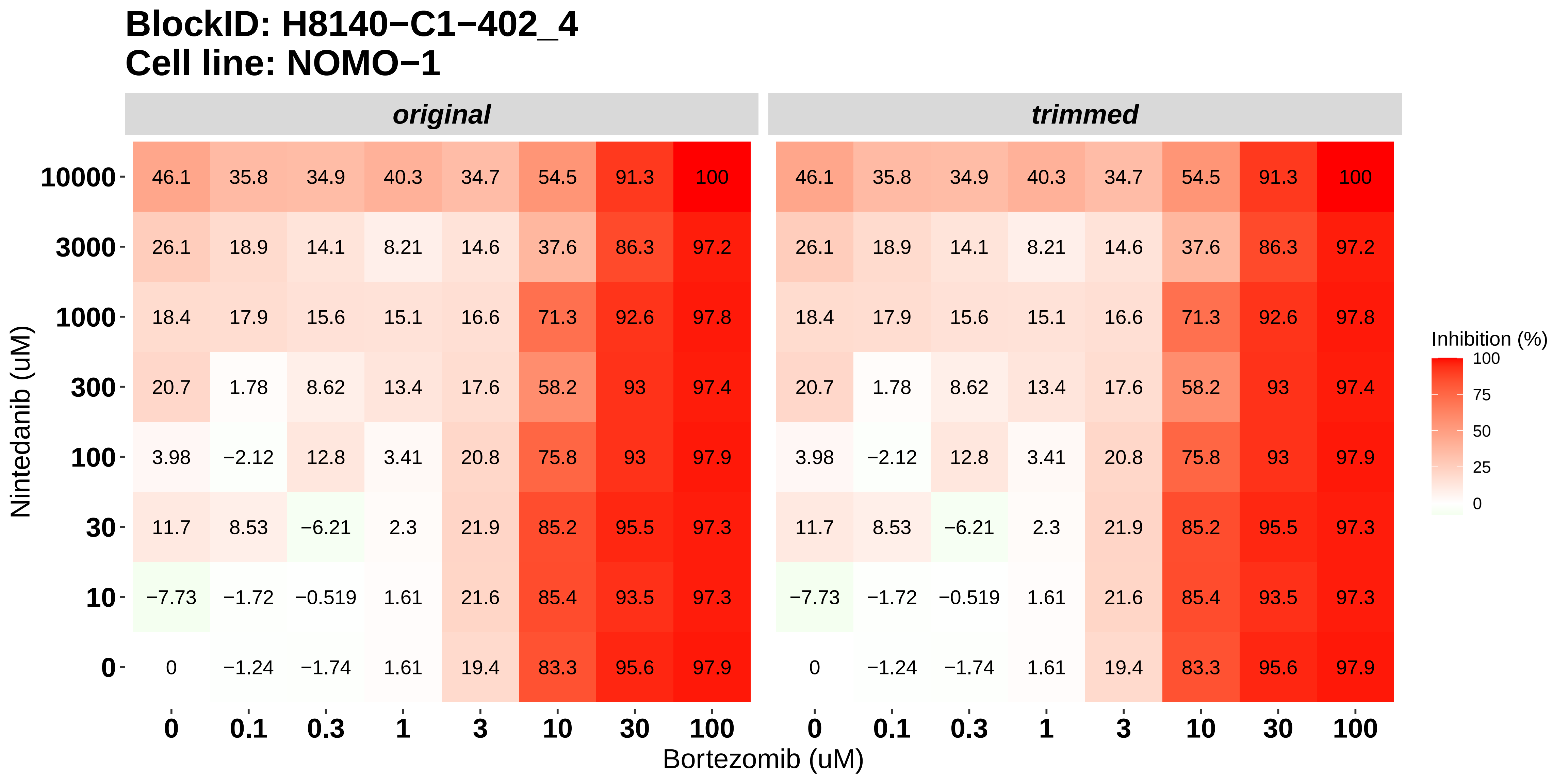

BlockID: H8140−C1−402_4
Cell line: NOMO−1
original
trimmed
Nintedanib (uM)
10000
3000
1000
300
100
30
10
0
46.1 35.8 34.9 40.3 34.7 54.5 91.3 100
26.1 18.9 14.1 8.21 14.6 37.6 86.3 97.2
18.4 17.9 15.6 15.1 16.6 71.3 92.6 97.8
20.7 1.78 8.62 13.4 17.6 58.2 93 97.4
3.98 −2.12 12.8 3.41 20.8 75.8 93 97.9
11.7 8.53 −6.21 2.3 21.9 85.2 95.5 97.3
−7.73 −1.72 −0.519 1.61 21.6 85.4 93.5 97.3
0 −1.24 −1.74 1.61 19.4 83.3 95.6 97.9
0 0.1 0.3 1 3 10 30 100
Bortezomib (uM)
Inhibition (%)
100
75
50
25
0

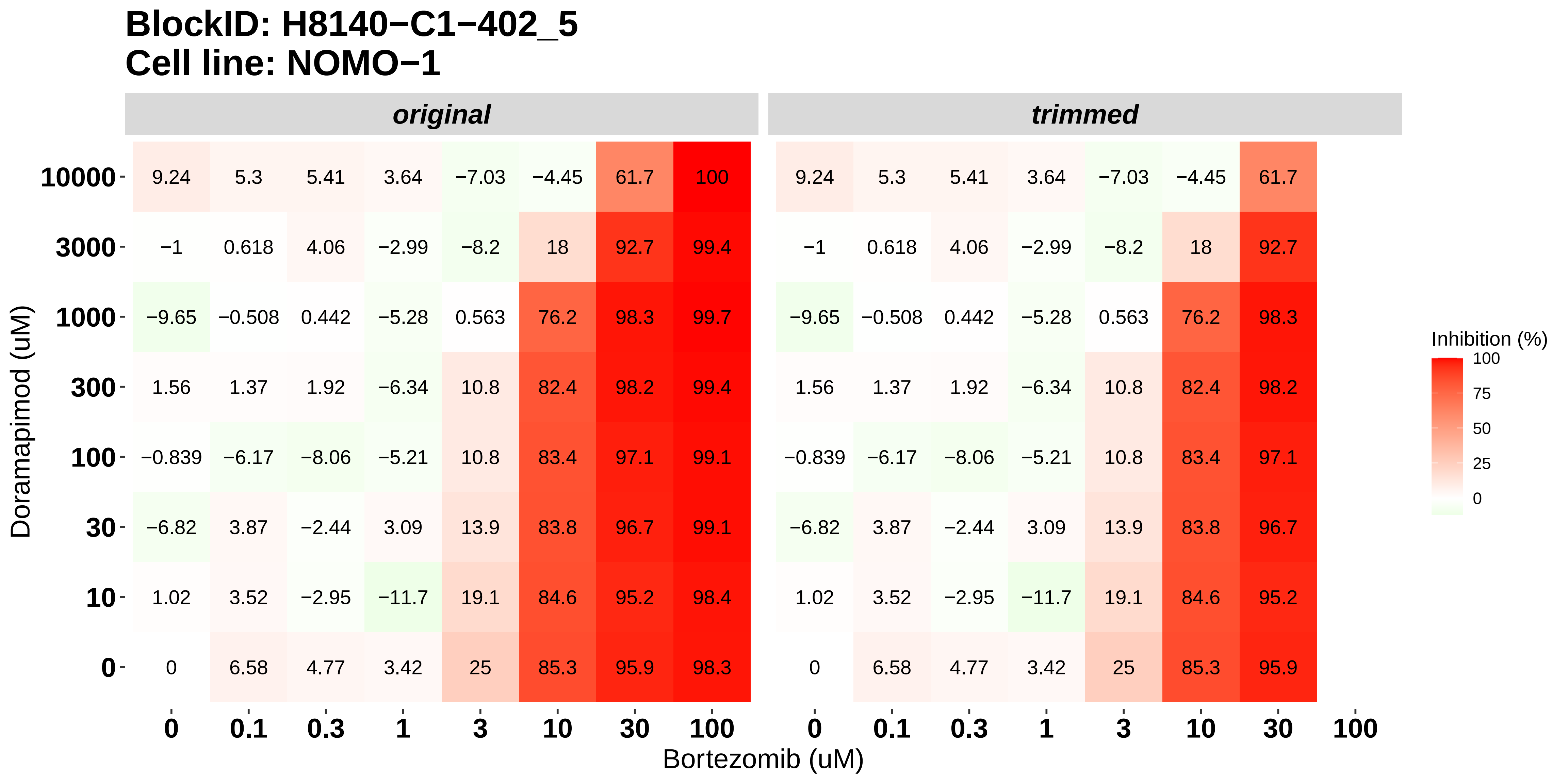

BlockID: H8140−C1−402_5
Cell line: NOMO−1
original
trimmed
Doramapimod (uM)
Bortezomib (uM)
Inhibition (%)
100
75
50
25
0
10000
3000
1000
300
100
30
10
0
0
0.1
0.3
1
3
10
30
100
9.24 5.3 5.41 3.64 −7.03 −4.45 61.7 100
−1 0.618 4.06 −2.99 −8.2 18 92.7 99.4
−9.65 −0.508 0.442 −5.28 0.563 76.2 98.3 99.7
1.56 1.37 1.92 −6.34 10.8 82.4 98.2 99.4
−0.839 −6.17 −8.06 −5.21 10.8 83.4 97.1 99.1
−6.82 3.87 −2.44 3.09 13.9 83.8 96.7 99.1
1.02 3.52 −2.95 −11.7 19.1 84.6 95.2 98.4
0 6.58 4.77 3.42 25 85.3 95.9 98.3
9.24 5.3 5.41 3.64 −7.03 −4.45 61.7
−1 0.618 4.06 −2.99 −8.2 18 92.7
−9.65 −0.508 0.442 −5.28 0.563 76.2 98.3
1.56 1.37 1.92 −6.34 10.8 82.4 98.2
−0.839 −6.17 −8.06 −5.21 10.8 83.4 97.1
−6.82 3.87 −2.44 3.09 13.9 83.8 96.7
1.02 3.52 −2.95 −11.7 19.1 84.6 95.2
0 6.58 4.77 3.42 25 85.3 95.9

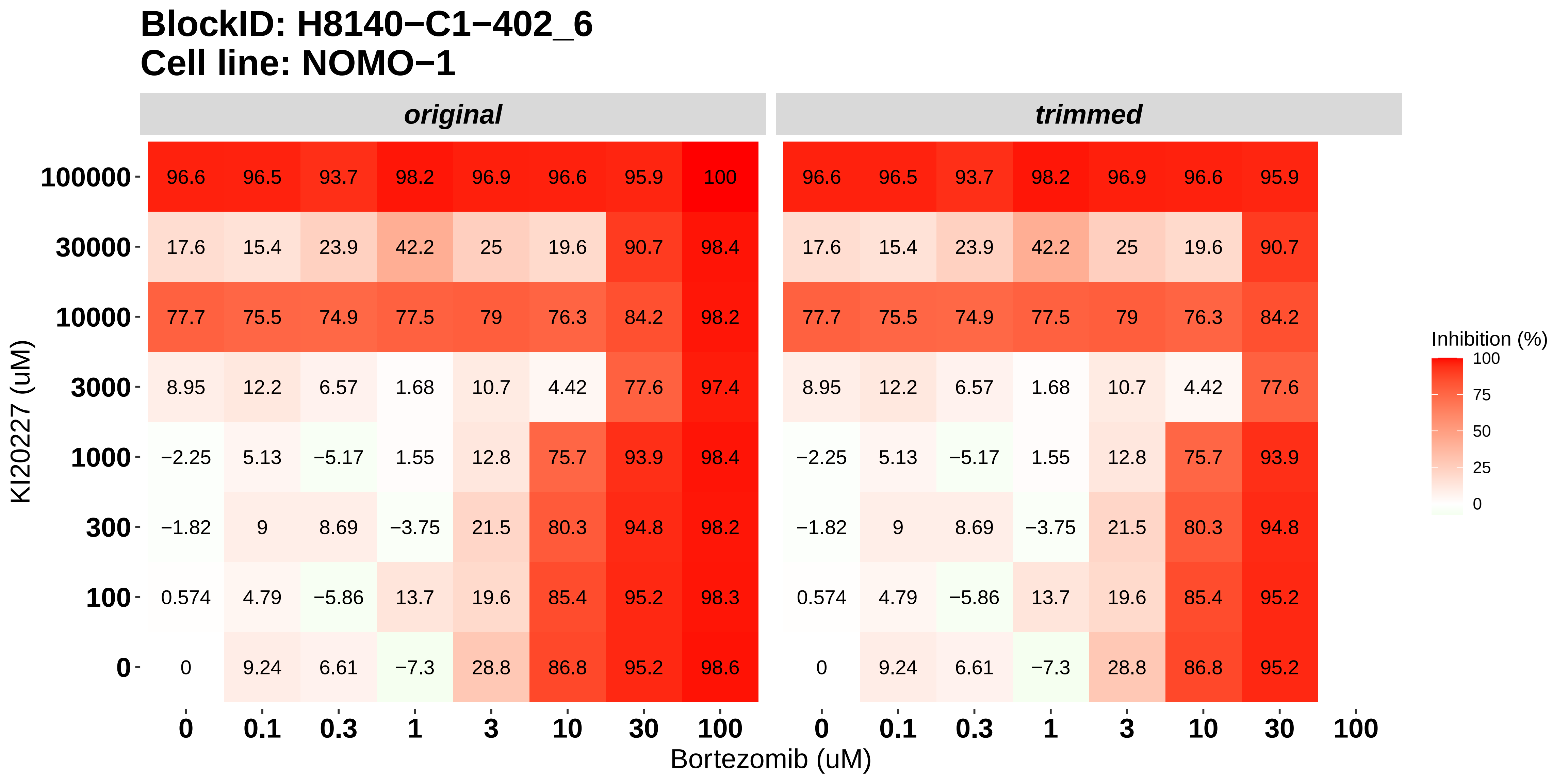
BlockID: H8140−C1−402_6
Cell line: NOMO−1
original
trimmed
KI20227 (uM)
100000
30000
10000
3000
1000
300
100
0
Bortezomib (uM)
0 0.1 0.3 1 3 10 30 100
0 0.1 0.3 1 3 10 30 100
Inhibition (%)
100
75
50
25
0
96.6 96.5 93.7 98.2 96.9 96.6 95.9 100
17.6 15.4 23.9 42.2 25 19.6 90.7 98.4
77.7 75.5 74.9 77.5 79 76.3 84.2 98.2
8.95 12.2 6.57 1.68 10.7 4.42 77.6 97.4
−2.25 5.13 −5.17 1.55 12.8 75.7 93.9 98.4
−1.82 9 8.69 −3.75 21.5 80.3 94.8 98.2
0.574 4.79 −5.86 13.7 19.6 85.4 95.2 98.3
0 9.24 6.61 −7.3 28.8 86.8 95.2 98.6
96.6 96.5 93.7 98.2 96.9 96.6 95.9
17.6 15.4 23.9 42.2 25 19.6 90.7
77.7 75.5 74.9 77.5 79 76.3 84.2
8.95 12.2 6.57 1.68 10.7 4.42 77.6
−2.25 5.13 −5.17 1.55 12.8 75.7 93.9
−1.82 9 8.69 −3.75 21.5 80.3 94.8
0.574 4.79 −5.86 13.7 19.6 85.4 95.2
0 9.24 6.61 −7.3 28.8 86.8 95.2

BlockID: H8140−C1−403_1
Cell line: OCI−AML3

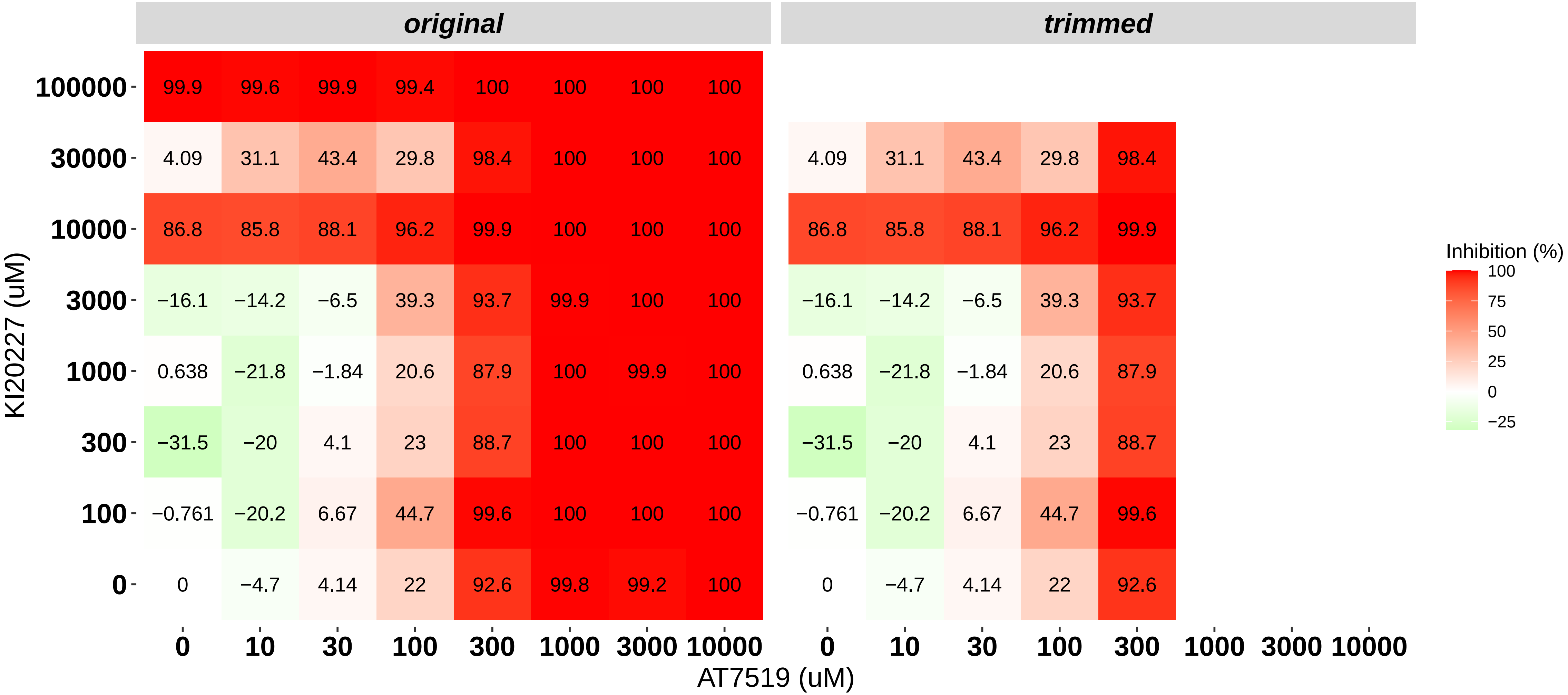

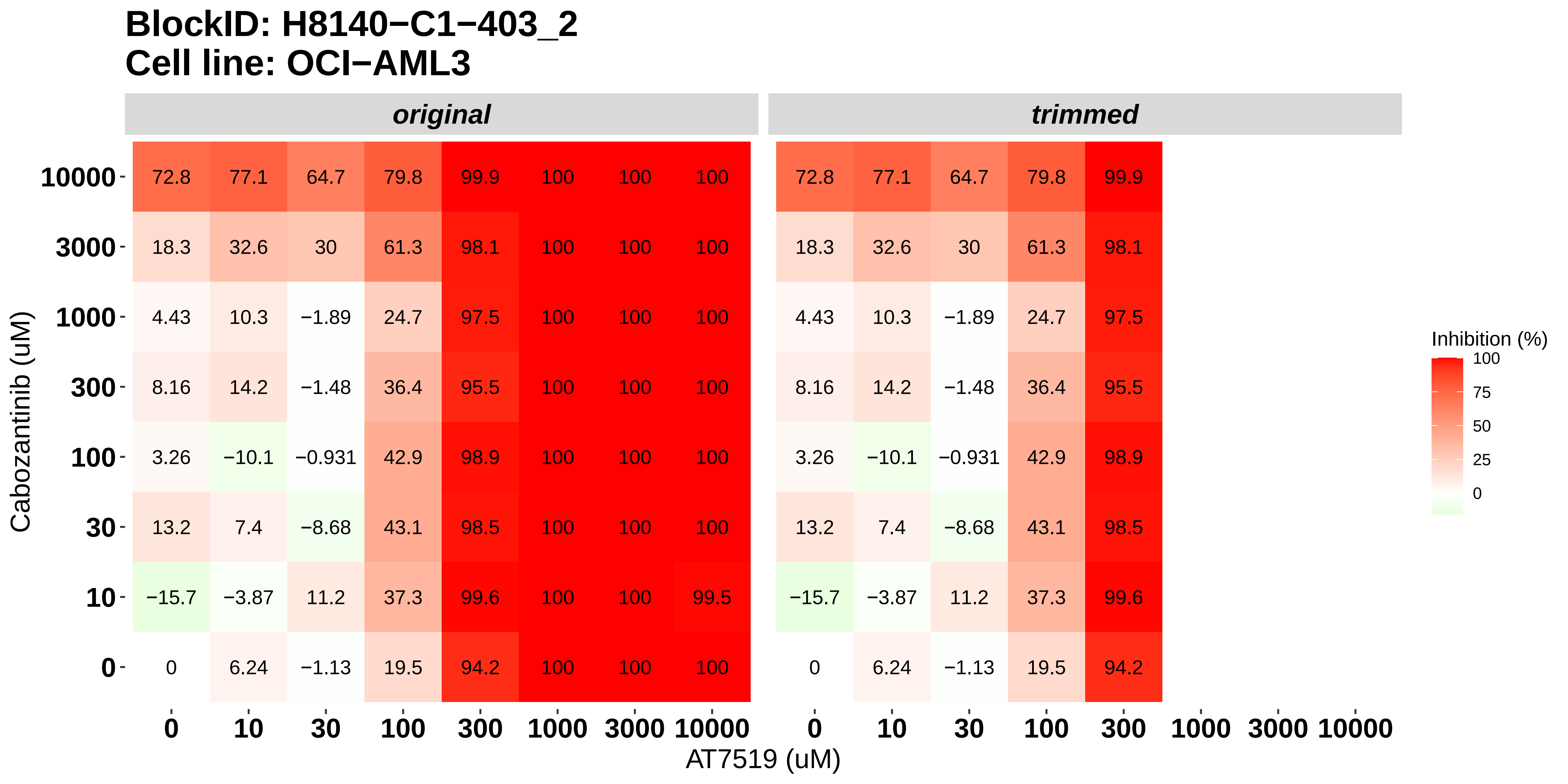

BlockID: H8140−C1−403_2
Cell line: OCI−AML3
original
trimmed
Cabozantinib (uM)
AT7519 (uM)
Inhibition (%)
10000
3000
1000
300
100
30
10
0
72.8 77.1 64.7 79.8 99.9 100 100 100
18.3 32.6 30 61.3 98.1 100 100 100
4.43 10.3 −1.89 24.7 97.5 100 100 100
8.16 14.2 −1.48 36.4 95.5 100 100 100
3.26 −10.1 −0.931 42.9 98.9 100 100 100
13.2 7.4 −8.68 43.1 98.5 100 100 100
−15.7 −3.87 11.2 37.3 99.6 100 100 99.5
0 6.24 −1.13 19.5 94.2 100 100 100
72.8 77.1 64.7 79.8 99.9
18.3 32.6 30 61.3 98.1
4.43 10.3 −1.89 24.7 97.5
8.16 14.2 −1.48 36.4 95.5
3.26 −10.1 −0.931 42.9 98.9
13.2 7.4 −8.68 43.1 98.5
−15.7 −3.87 11.2 37.3 99.6
0 6.24 −1.13 19.5 94.2
0 10 30 100 300 1000 3000 10000
100 75 50 25 0

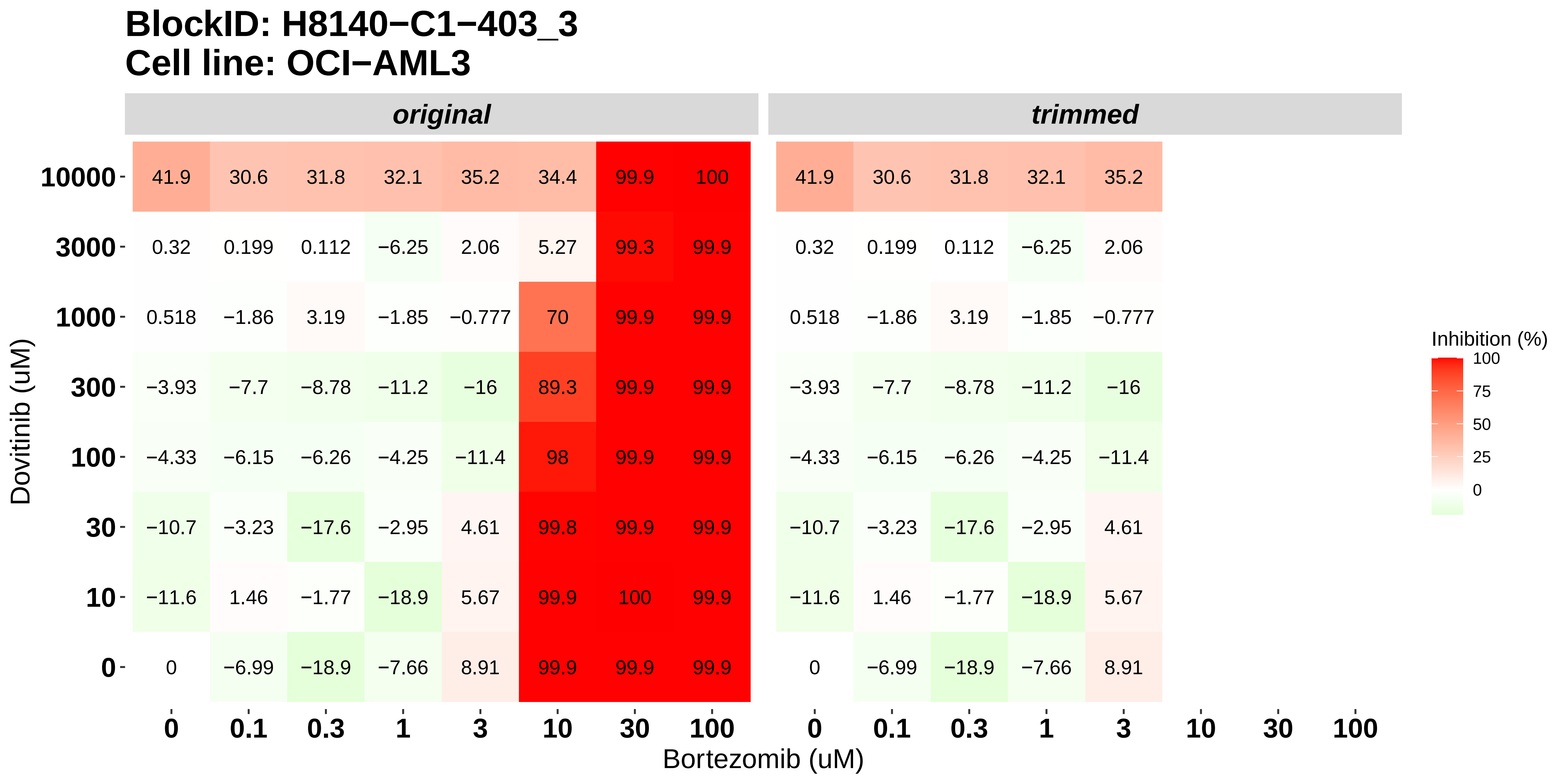
BlockID: H8140−C1−403_3
Cell line: OCI−AML3
original
trimmed
10000
3000
1000
300
100
30
10
0
41.9 30.6 31.8 32.1 35.2 34.4 99.9 100
0.32 0.199 0.112 −6.25 2.06 5.27 99.3 99.9
0.518 −1.86 3.19 −1.85 −0.777 70 99.9 99.9
−3.93 −7.7 −8.78 −11.2 −16 89.3 99.9 99.9
−4.33 −6.15 −6.26 −4.25 −11.4 98 99.9 99.9
−10.7 −3.23 −17.6 −2.95 4.61 99.8 99.9 99.9
−11.6 1.46 −1.77 −18.9 5.67 99.9 100 99.9
0 −6.99 −18.9 −7.66 8.91 99.9 99.9 99.9
41.9 30.6 31.8 32.1 35.2
0.32 0.199 0.112 −6.25 2.06
0.518 −1.86 3.19 −1.85 −0.777
−3.93 −7.7 −8.78 −11.2 −16
−4.33 −6.15 −6.26 −4.25 −11.4
−10.7 −3.23 −17.6 −2.95 4.61
−11.6 1.46 −1.77 −18.9 5.67
0 −6.99 −18.9 −7.66 8.91
0 0.1 0.3 1 3 10 30 100
0 0.1 0.3 1 3 10 30 100
Dovitinib (uM)
Bortezomib (uM)
Inhibition (%)
100
75
50
25
0

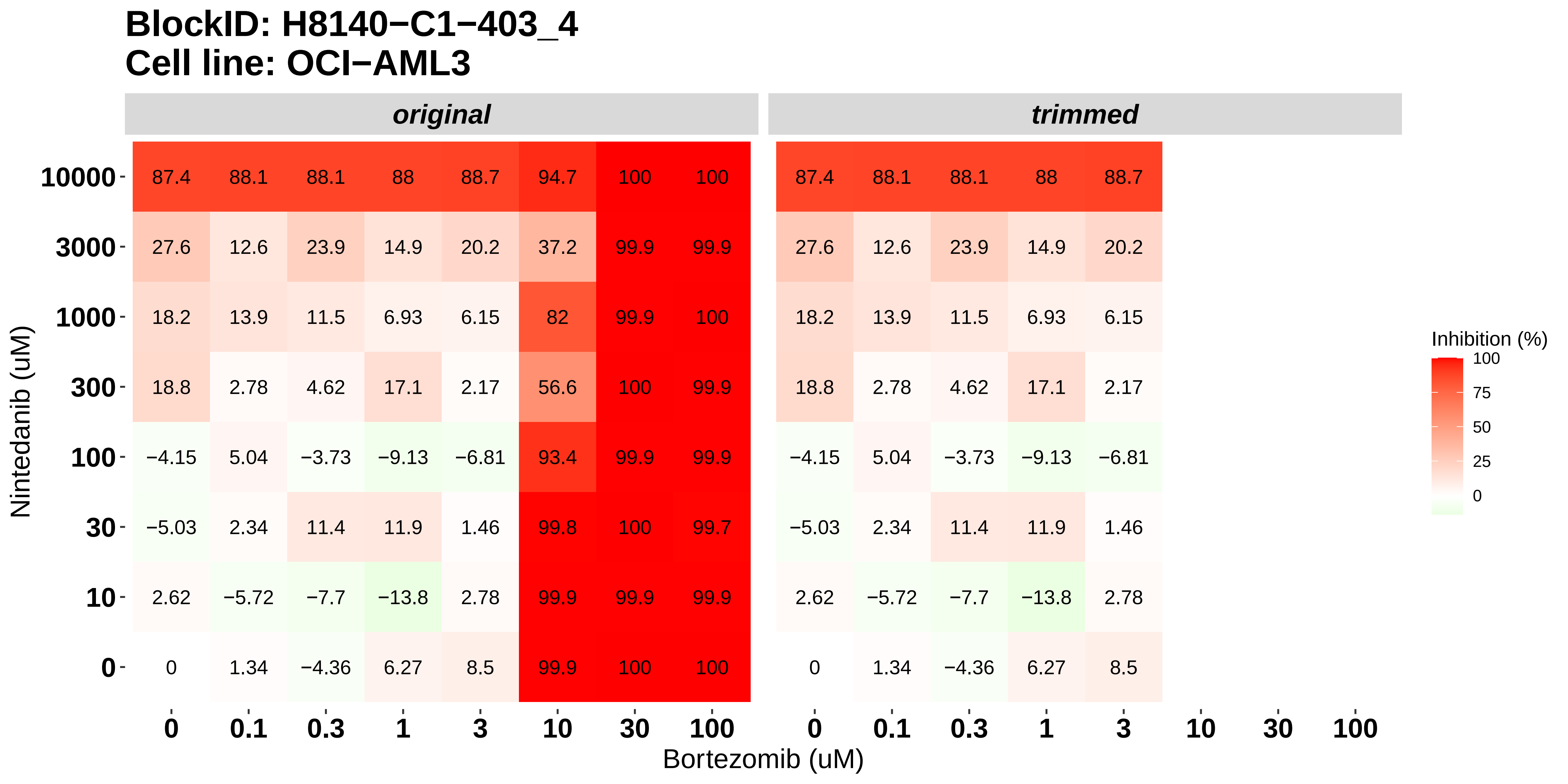

BlockID: H8140−C1−403_4
Cell line: OCI−AML3
original
trimmed
Nintedanib (uM)
Bortezomib (uM)
Inhibition (%)
10000
3000
1000
300
100
30
10
0
0
0.1
0.3
1
3
10
30
100
87.4 88.1 88.1 88 88.7 94.7 100 100
27.6 12.6 23.9 14.9 20.2 37.2 99.9 99.9
18.2 13.9 11.5 6.93 6.15 82 99.9 100
18.8 2.78 4.62 17.1 2.17 56.6 100 99.9
−4.15 5.04 −3.73 −9.13 −6.81 93.4 99.9 99.9
−5.03 2.34 11.4 11.9 1.46 99.8 100 99.7
2.62 −5.72 −7.7 −13.8 2.78 99.9 99.9 99.9
0 1.34 −4.36 6.27 8.5 99.9 100 100
87.4 88.1 88.1 88 88.7
27.6 12.6 23.9 14.9 20.2
18.2 13.9 11.5 6.93 6.15
18.8 2.78 4.62 17.1 2.17
−4.15 5.04 −3.73 −9.13 −6.81
−5.03 2.34 11.4 11.9 1.46
2.62 −5.72 −7.7 −13.8 2.78
0 1.34 −4.36 6.27 8.5
100
75
50
25
0

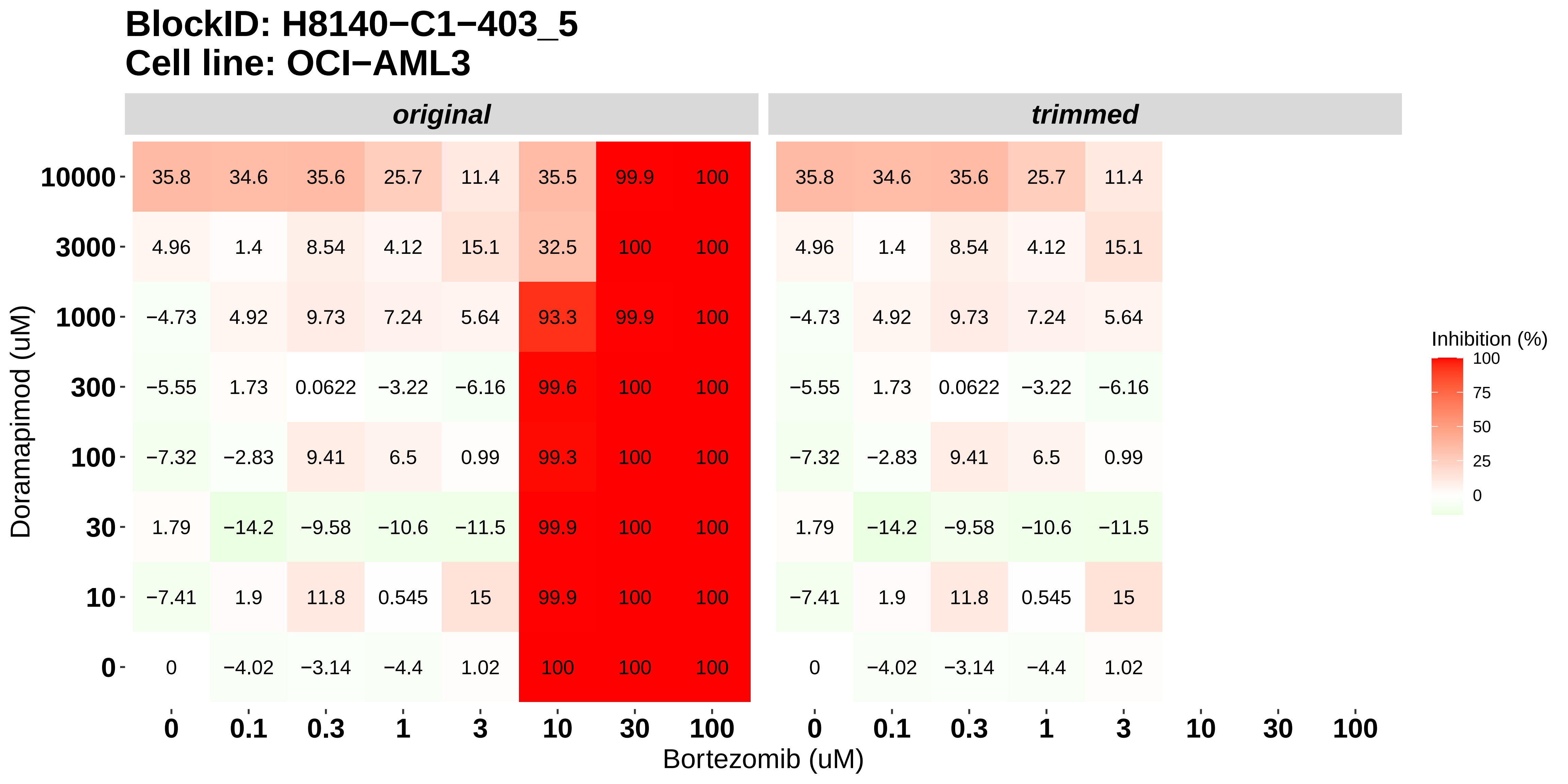
BlockID: H8140−C1−403_5
Cell line: OCI−AML3
original
trimmed
Doramapimod (uM)
Bortezomib (uM)
Inhibition (%)
10000
3000
1000
300
100
30
10
0
0.1
0.3
1
3
10
30
100
35.8 34.6 35.6 25.7 11.4 35.5 99.9 100
4.96 1.4 8.54 4.12 15.1 32.5 100 100
−4.73 4.92 9.73 7.24 5.64 93.3 99.9 100
−5.55 1.73 0.0622 −3.22 −6.16 99.6 100 100
−7.32 −2.83 9.41 6.5 0.99 99.3 100 100
1.79 −14.2 −9.58 −10.6 −11.5 99.9 100 100
−7.41 1.9 11.8 0.545 15 99.9 100 100
0 −4.02 −3.14 −4.4 1.02 100 100 100
35.8 34.6 35.6 25.7 11.4
4.96 1.4 8.54 4.12 15.1
−4.73 4.92 9.73 7.24 5.64
−5.55 1.73 0.0622 −3.22 −6.16
−7.32 −2.83 9.41 6.5 0.99
1.79 −14.2 −9.58 −10.6 −11.5
−7.41 1.9 11.8 0.545 15
0 −4.02 −3.14 −4.4 1.02
100
75
50
25
0

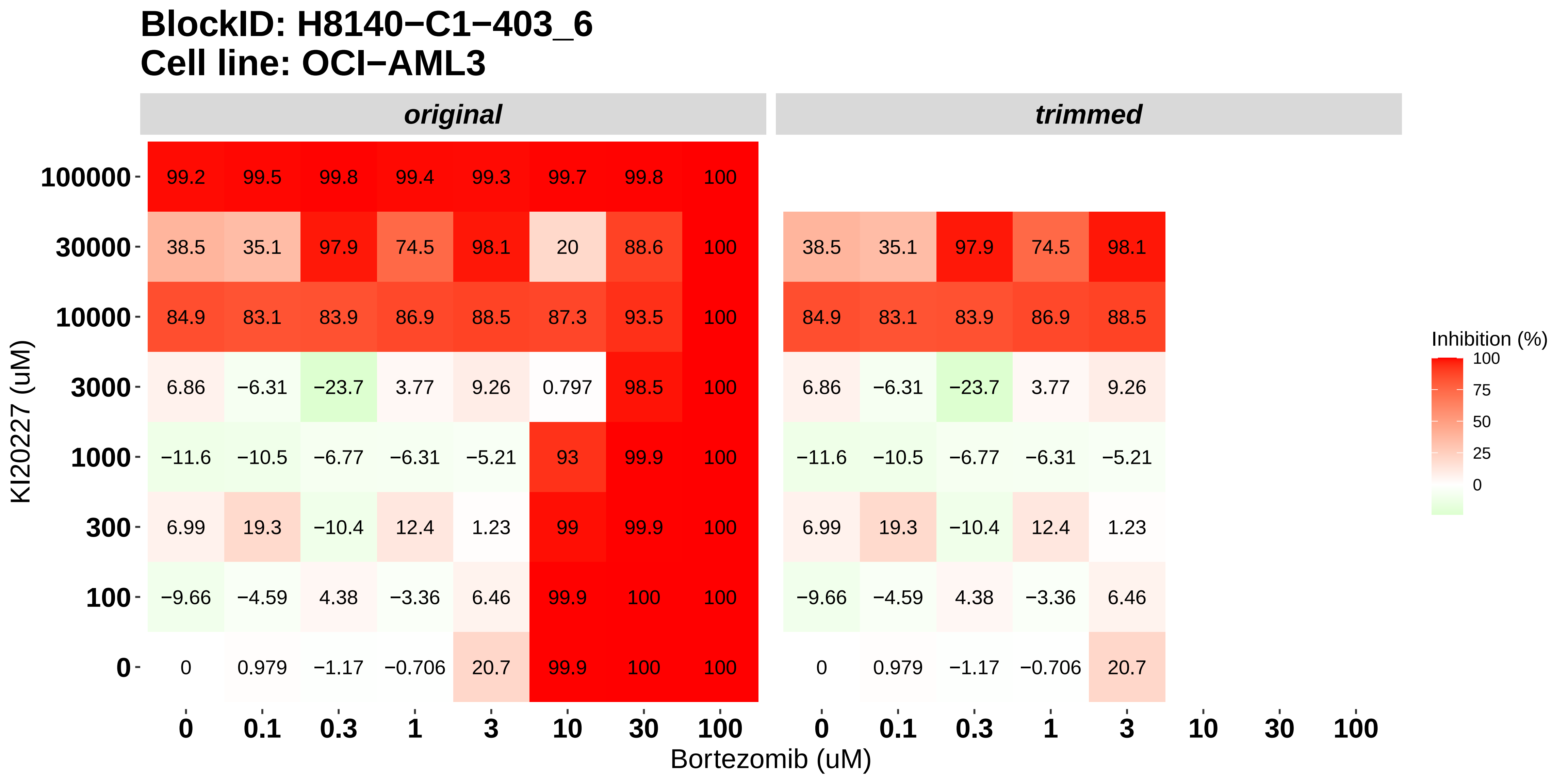
BlockID: H8140−C1−403_6
Cell line: OCI−AML3
original
trimmed
KI20227 (uM)
100000
30000
10000
3000
1000
300
100
0
99.2 99.5 99.8 99.4 99.3 99.7 99.8 100
38.5 35.1 97.9 74.5 98.1 20 88.6 100
84.9 83.1 83.9 86.9 88.5 87.3 93.5 100
6.86 −6.31 −23.7 3.77 9.26 0.797 98.5 100
−11.6 −10.5 −6.77 −6.31 −5.21 93 99.9 100
6.99 19.3 −10.4 12.4 1.23 99 99.9 100
−9.66 −4.59 4.38 −3.36 6.46 99.9 100 100
0 0.979 −1.17 −0.706 20.7 99.9 100 100
38.5 35.1 97.9 74.5 98.1
84.9 83.1 83.9 86.9 88.5
6.86 −6.31 −23.7 3.77 9.26
−11.6 −10.5 −6.77 −6.31 −5.21
6.99 19.3 −10.4 12.4 1.23
−9.66 −4.59 4.38 −3.36 6.46
0 0.979 −1.17 −0.706 20.7
0 0.1 0.3 1 3 10 30 100
0 0.1 0.3 1 3 10 30 100
Bortezomib (uM)
Inhibition (%)
100
75
50
25
0

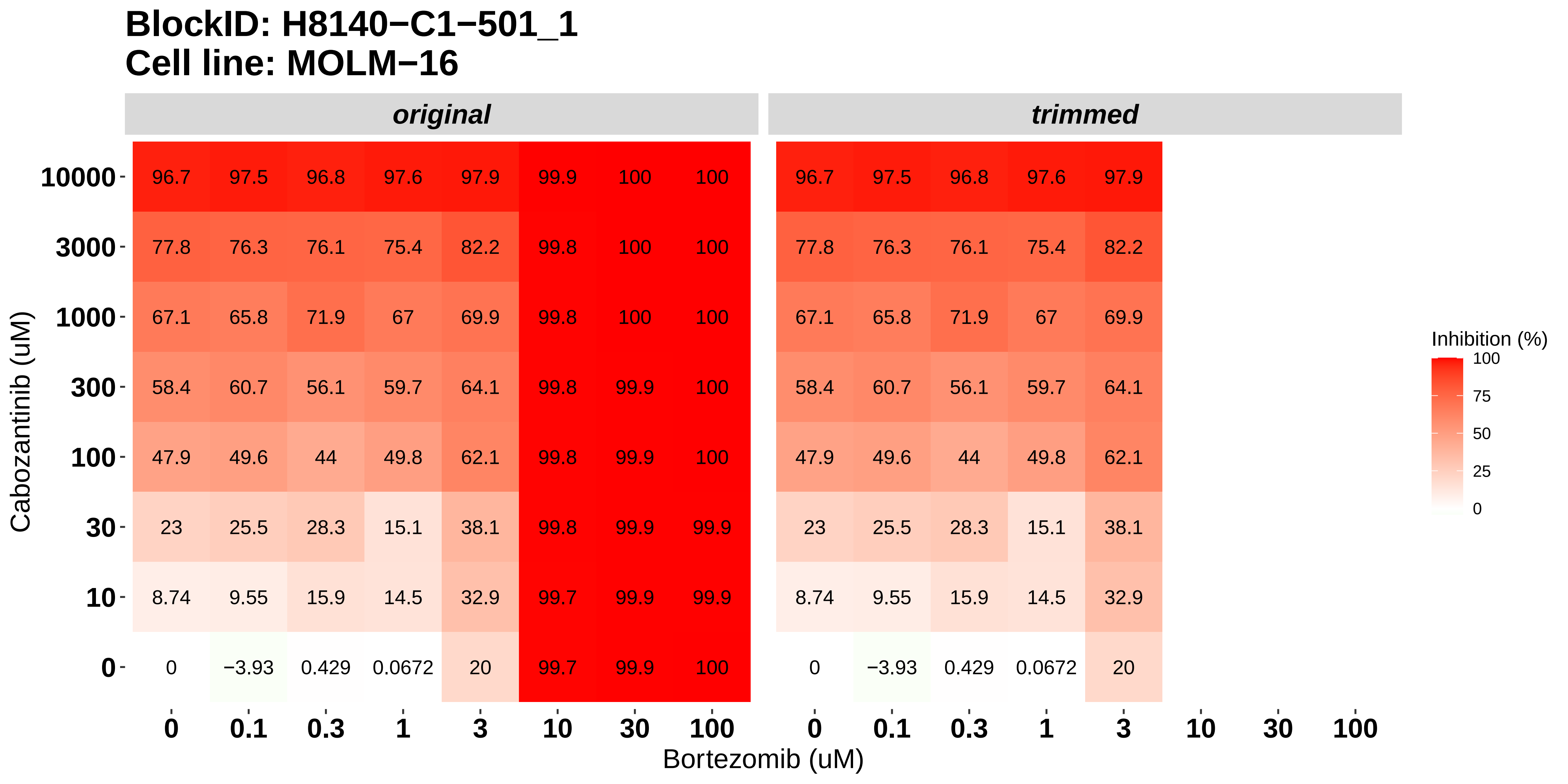
BlockID: H8140−C1−501_1
Cell line: MOLM−16
original
trimmed
Cabozantinib (uM)
10000
3000
1000
300
100
30
10
0
96.7
97.5
96.8
97.6
97.9
99.9
100
100
77.8
76.3
76.1
75.4
82.2
99.8
67.1
65.8
71.9
67
69.9
58.4
60.7
56.1
59.7
64.1
99.9
47.9
49.6
44
49.8
62.1
23
25.5
28.3
15.1
38.1
8.74
9.55
15.9
14.5
32.9
99.7
−3.93
0.429
0.0672
20
0.1
0.3
1
3
Bortezomib (uM)
Inhibition (%)
75
50
25

BlockID: H8140−C1−501_2
Cell line: MOLM−16

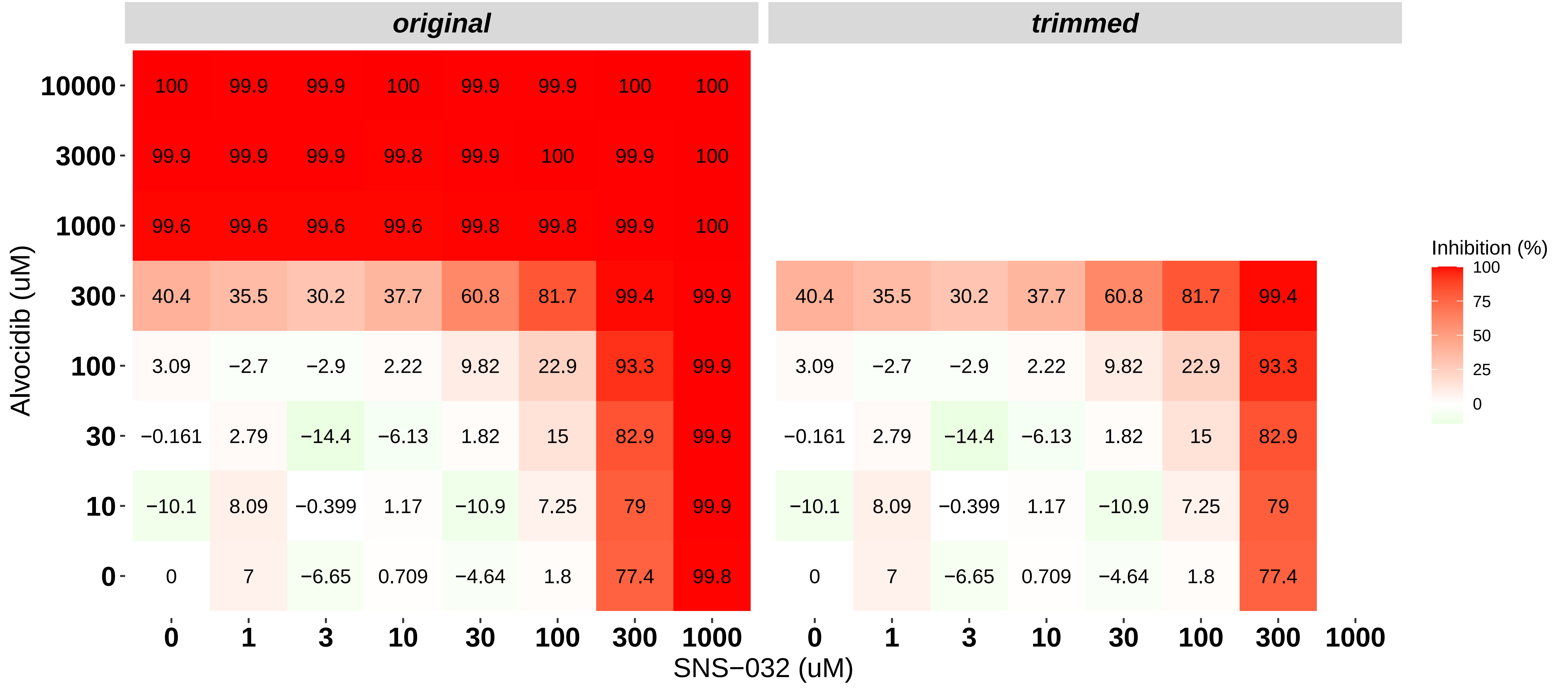

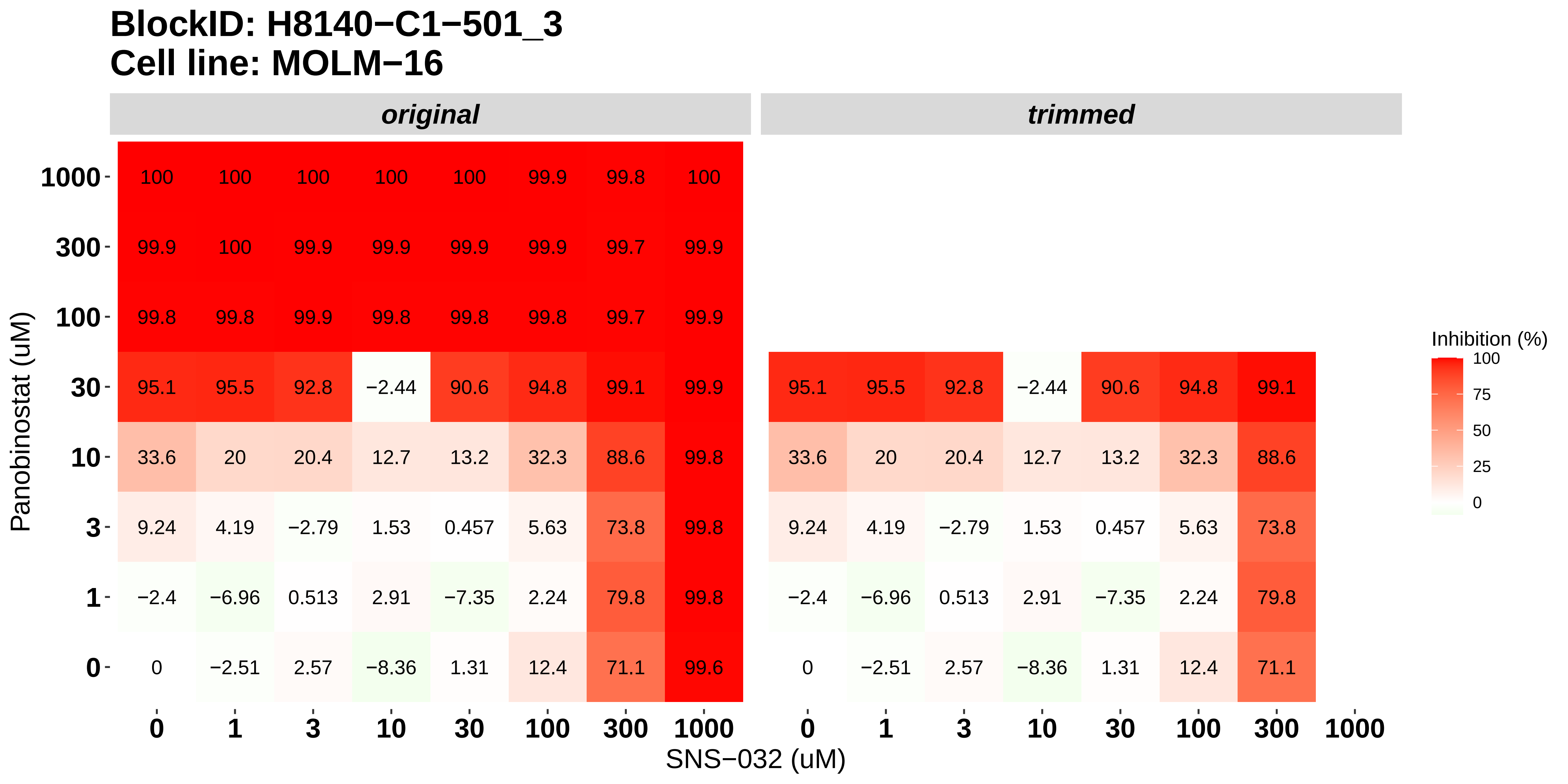
BlockID: H8140−C1−501_3
Cell line: MOLM−16
original
trimmed
Panobinostat (uM)
SNS−032 (uM)
Inhibition (%)
1000
300
100
30
10
3
1
0
100 100 100 100 100 99.9 99.8 100
99.9 100 99.9 99.9 99.9 99.9 99.7 99.9
99.8 99.8 99.9 99.8 99.8 99.8 99.7 99.9
95.1 95.5 92.8 −2.44 90.6 94.8 99.1 99.9
33.6 20 20.4 12.7 13.2 32.3 88.6 99.8
9.24 4.19 −2.79 1.53 0.457 5.63 73.8 99.8
−2.4 −6.96 0.513 2.91 −7.35 2.24 79.8 99.8
0 −2.51 2.57 −8.36 1.31 12.4 71.1 99.6
95.1 95.5 92.8 −2.44 90.6 94.8 99.1
33.6 20 20.4 12.7 13.2 32.3 88.6
9.24 4.19 −2.79 1.53 0.457 5.63 73.8
−2.4 −6.96 0.513 2.91 −7.35 2.24 79.8
0 −2.51 2.57 −8.36 1.31 12.4 71.1
0 1 3 10 30 100 300 1000
0 1 3 10 30 100 300 1000
100
75
50
25
0

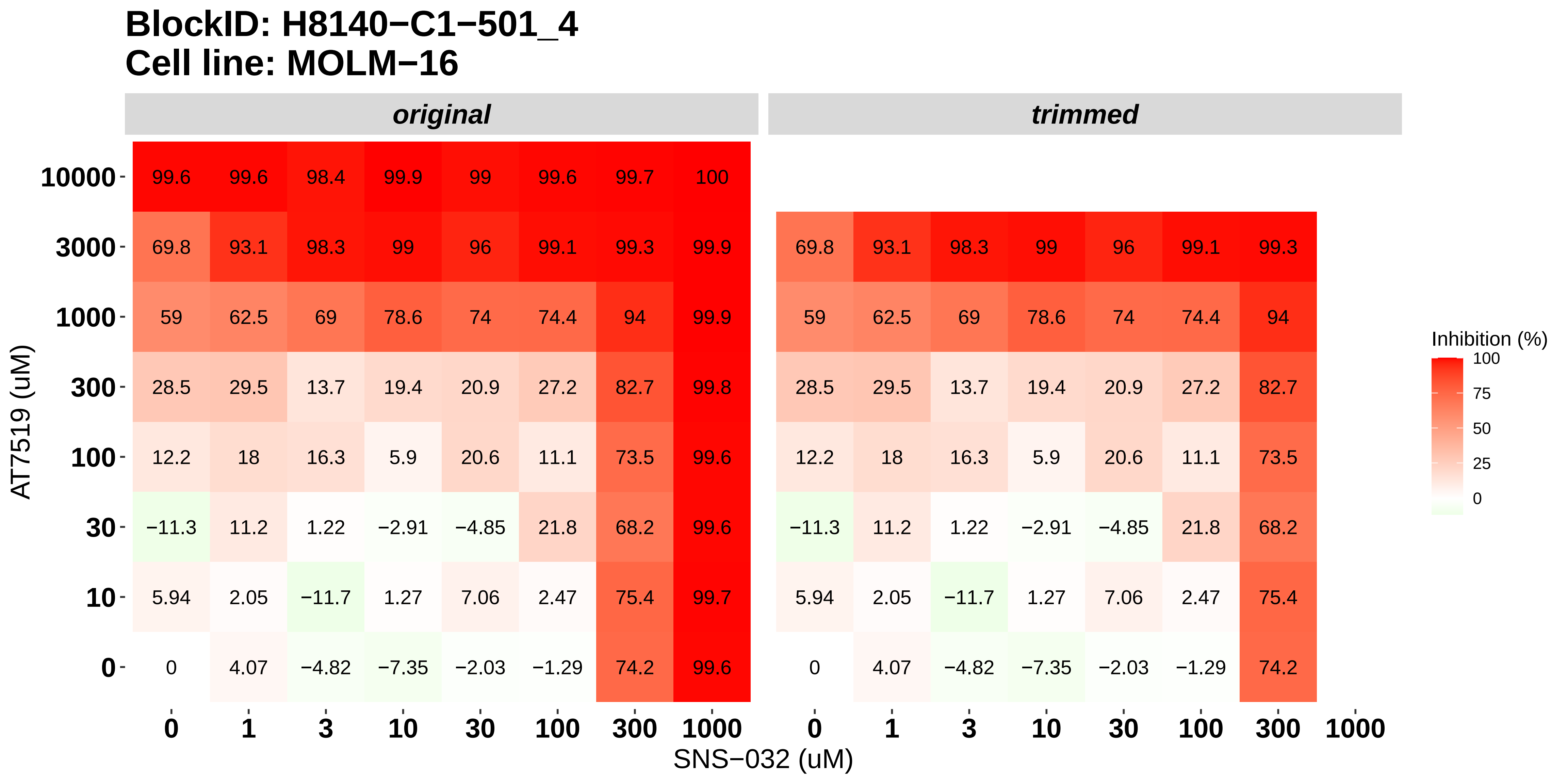
BlockID: H8140−C1−501_4
Cell line: MOLM−16
original
trimmed
AT7519 (uM)
10000
3000
1000
300
100
30
10
0
SNS−032 (uM)
0
1
3
10
30
100
300
1000
Inhibition (%)
100
75
50
25
0
99.6 99.6 98.4 99.9 99 99.6 99.7 100
69.8 93.1 98.3 99 96 99.1 99.3 99.9
59 62.5 69 78.6 74 74.4 94 99.9
28.5 29.5 13.7 19.4 20.9 27.2 82.7 99.8
12.2 18 16.3 5.9 20.6 11.1 73.5 99.6
−11.3 11.2 1.22 −2.91 −4.85 21.8 68.2 99.6
5.94 2.05 −11.7 1.27 7.06 2.47 75.4 99.7
0 4.07 −4.82 −7.35 −2.03 −1.29 74.2 99.6
69.8 93.1 98.3 99 96 99.1 99.3
59 62.5 69 78.6 74 74.4 94
28.5 29.5 13.7 19.4 20.9 27.2 82.7
12.2 18 16.3 5.9 20.6 11.1 73.5
−11.3 11.2 1.22 −2.91 −4.85 21.8 68.2
5.94 2.05 −11.7 1.27 7.06 2.47 75.4
0 4.07 −4.82 −7.35 −2.03 −1.29 74.2

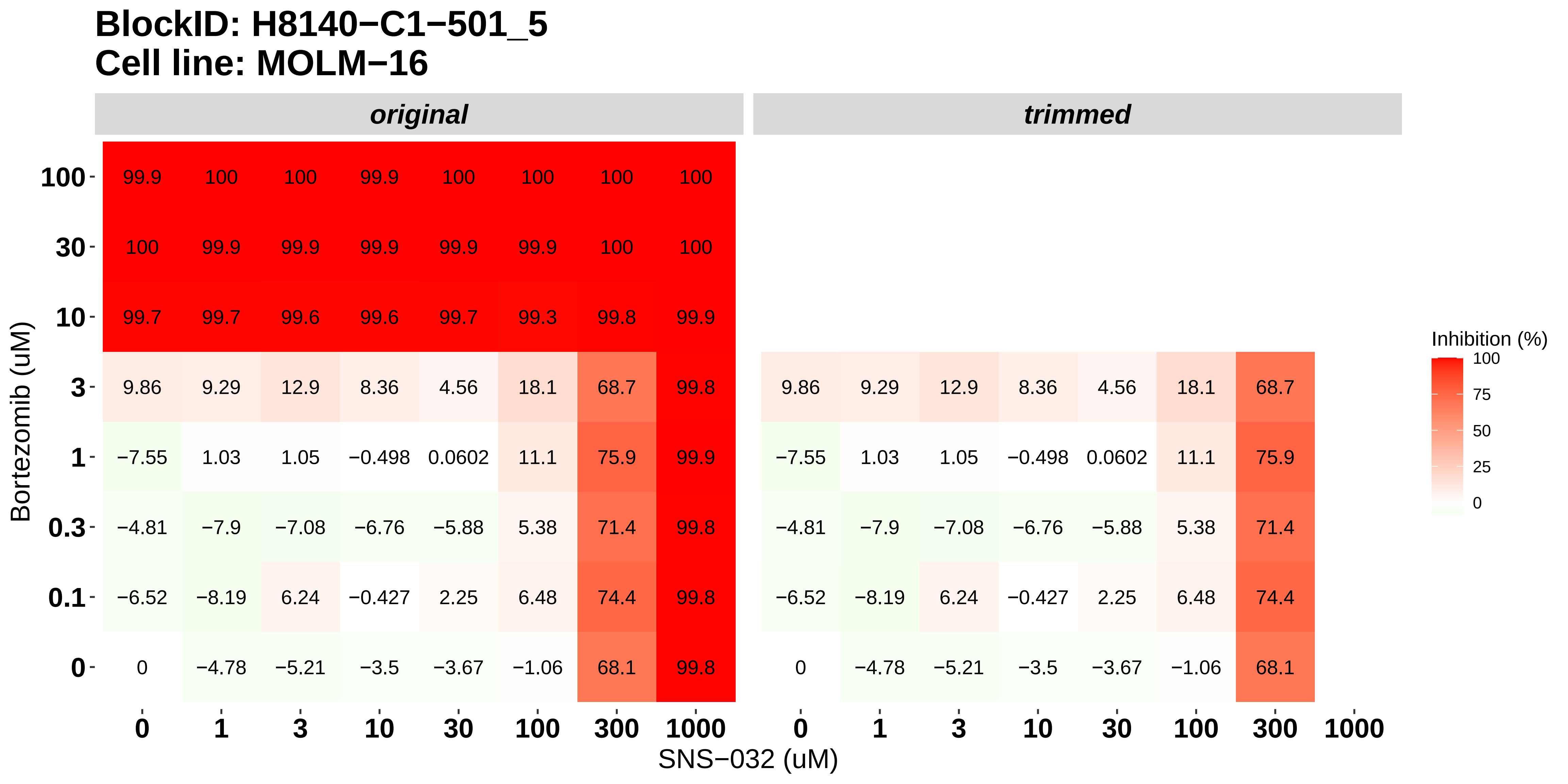
BlockID: H8140−C1−501_5
Cell line: MOLM−16
original
trimmed
Bortezomib (uM)
100
30
10
3
1
0.3
0.1
0
99.9 100 100 99.9 100 100 100 100
100 99.9 99.9 99.9 99.9 99.9 100 100
99.7 99.7 99.6 99.6 99.7 99.3 99.8 99.9
9.86 9.29 12.9 8.36 4.56 18.1 68.7 99.8
−7.55 1.03 1.05 −0.498 0.0602 11.1 75.9 99.9
−4.81 −7.9 −7.08 −6.76 −5.88 5.38 71.4 99.8
−6.52 −8.19 6.24 −0.427 2.25 6.48 74.4 99.8
0 −4.78 −5.21 −3.5 −3.67 −1.06 68.1 99.8
9.86 9.29 12.9 8.36 4.56 18.1 68.7
−7.55 1.03 1.05 −0.498 0.0602 11.1 75.9
−4.81 −7.9 −7.08 −6.76 −5.88 5.38 71.4
−6.52 −8.19 6.24 −0.427 2.25 6.48 74.4
0 −4.78 −5.21 −3.5 −3.67 −1.06 68.1
0 1 3 10 30 100 300 1000
0 1 3 10 30 100 300 1000
SNS−032 (uM)
Inhibition (%)
100
75
50
25
0

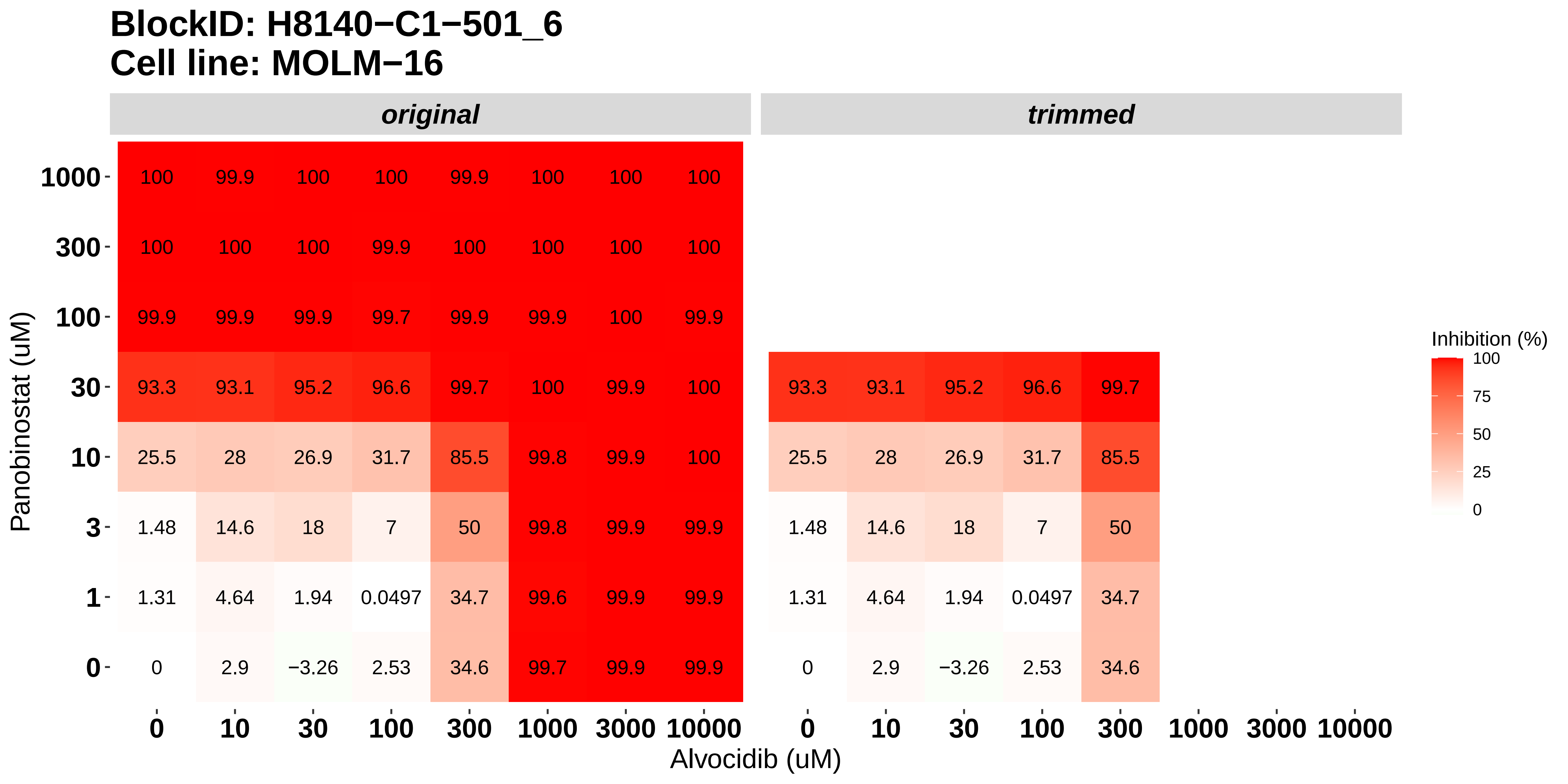
BlockID: H8140−C1−501_6
Cell line: MOLM−16
original
trimmed
Panobinostat (uM)
Alvocidib (uM)
Inhibition (%)
1000
300
100
30
10
3
1
0
0
10
30
100
300
1000
3000
10000
100 99.9 100 100 99.9 100 100 100
100 100 100 99.9 100 100 100 100
99.9 99.9 99.9 99.7 99.9 99.9 100 99.9
93.3 93.1 95.2 96.6 99.7 100 99.9 100
25.5 28 26.9 31.7 85.5 99.8 99.9 100
1.48 14.6 18 7 50 99.8 99.9 99.9
1.31 4.64 1.94 0.0497 34.7 99.6 99.9 99.9
0 2.9 −3.26 2.53 34.6 99.7 99.9 99.9
93.3 93.1 95.2 96.6 99.7
25.5 28 26.9 31.7 85.5
1.48 14.6 18 7 50
1.31 4.64 1.94 0.0497 34.7
0 2.9 −3.26 2.53 34.6
100
75
50
25
0

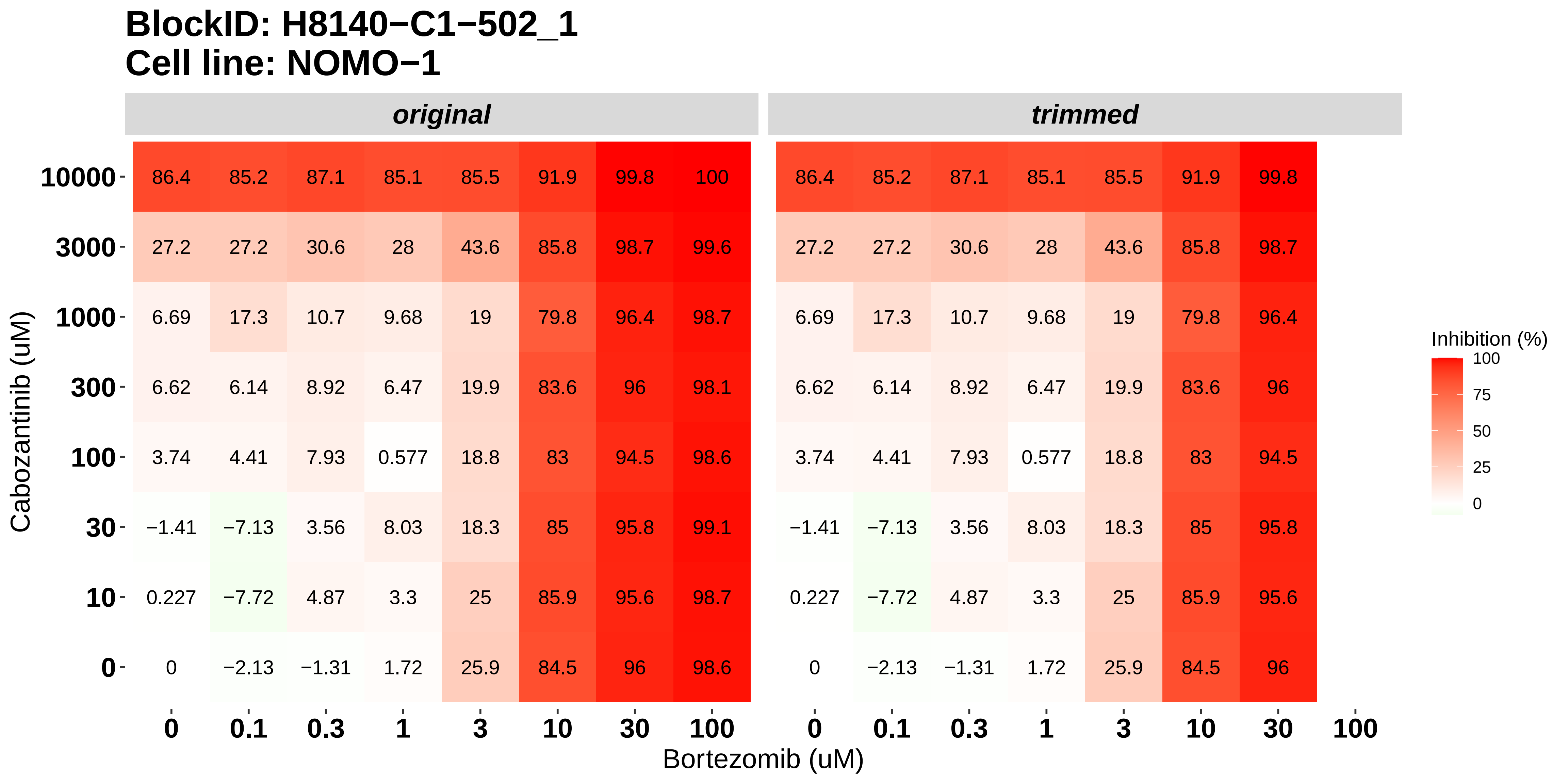

BlockID: H8140−C1−502_1
Cell line: NOMO−1
original
trimmed
Cabozantinib (uM)
Bortezomib (uM)
Inhibition (%)
10000
3000
1000
300
100
30
10
0
0.1
0.3
1
3
10
30
100
86.4 85.2 87.1 85.1 85.5 91.9 99.8 100
27.2 27.2 30.6 28 43.6 85.8 98.7 99.6
6.69 17.3 10.7 9.68 19 79.8 96.4 98.7
6.62 6.14 8.92 6.47 19.9 83.6 96 98.1
3.74 4.41 7.93 0.577 18.8 83 94.5 98.6
−1.41 −7.13 3.56 8.03 18.3 85 95.8 99.1
0.227 −7.72 4.87 3.3 25 85.9 95.6 98.7
0 −2.13 −1.31 1.72 25.9 84.5 96 98.6
86.4 85.2 87.1 85.1 85.5 91.9 99.8
27.2 27.2 30.6 28 43.6 85.8 98.7
6.69 17.3 10.7 9.68 19 79.8 96.4
6.62 6.14 8.92 6.47 19.9 83.6 96
3.74 4.41 7.93 0.577 18.8 83 94.5
−1.41 −7.13 3.56 8.03 18.3 85 95.8
0.227 −7.72 4.87 3.3 25 85.9 95.6
0 −2.13 −1.31 1.72 25.9 84.5 96
100
75
50
25
0

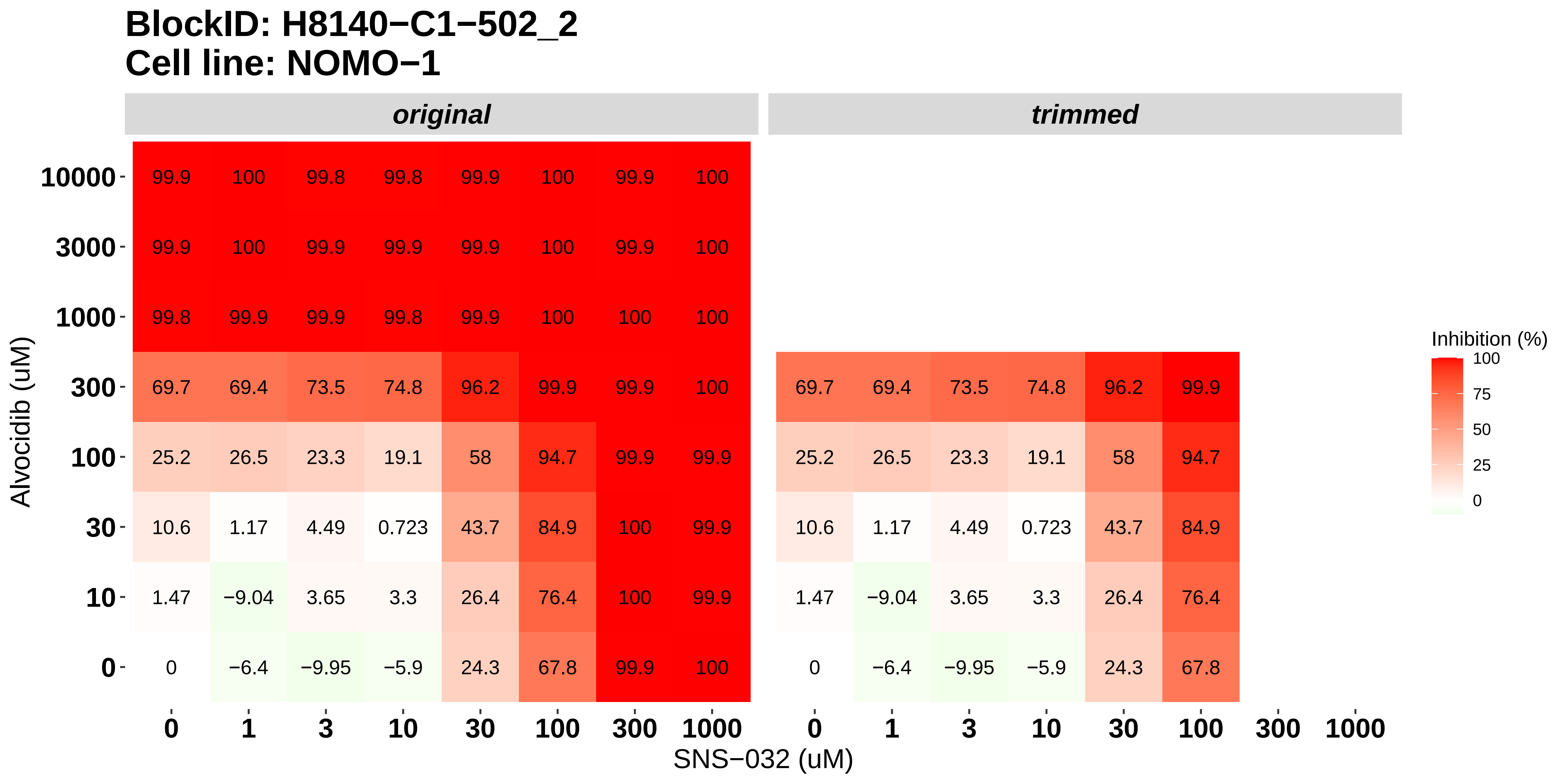

BlockID: H8140−C1−502_2
Cell line: NOMO−1
original
trimmed
Alvocidib (uM)
SNS−032 (uM)
Inhibition (%)
10000
3000
1000
300
100
30
10
0
0
1
3
10
30
100
300
1000
99.9 100 99.8 99.8 99.9 100 99.9 100
99.9 100 99.9 99.9 99.9 100 99.9 100
99.8 99.9 99.9 99.8 99.9 100 100 100
69.7 69.4 73.5 74.8 96.2 99.9 99.9 100
25.2 26.5 23.3 19.1 58 94.7 99.9 99.9
10.6 1.17 4.49 0.723 43.7 84.9 100 99.9
1.47 −9.04 3.65 3.3 26.4 76.4 100 99.9
0 −6.4 −9.95 −5.9 24.3 67.8 99.9 100
69.7 69.4 73.5 74.8 96.2 99.9
25.2 26.5 23.3 19.1 58 94.7
10.6 1.17 4.49 0.723 43.7 84.9
1.47 −9.04 3.65 3.3 26.4 76.4
0 −6.4 −9.95 −5.9 24.3 67.8
100
75
50
25
0

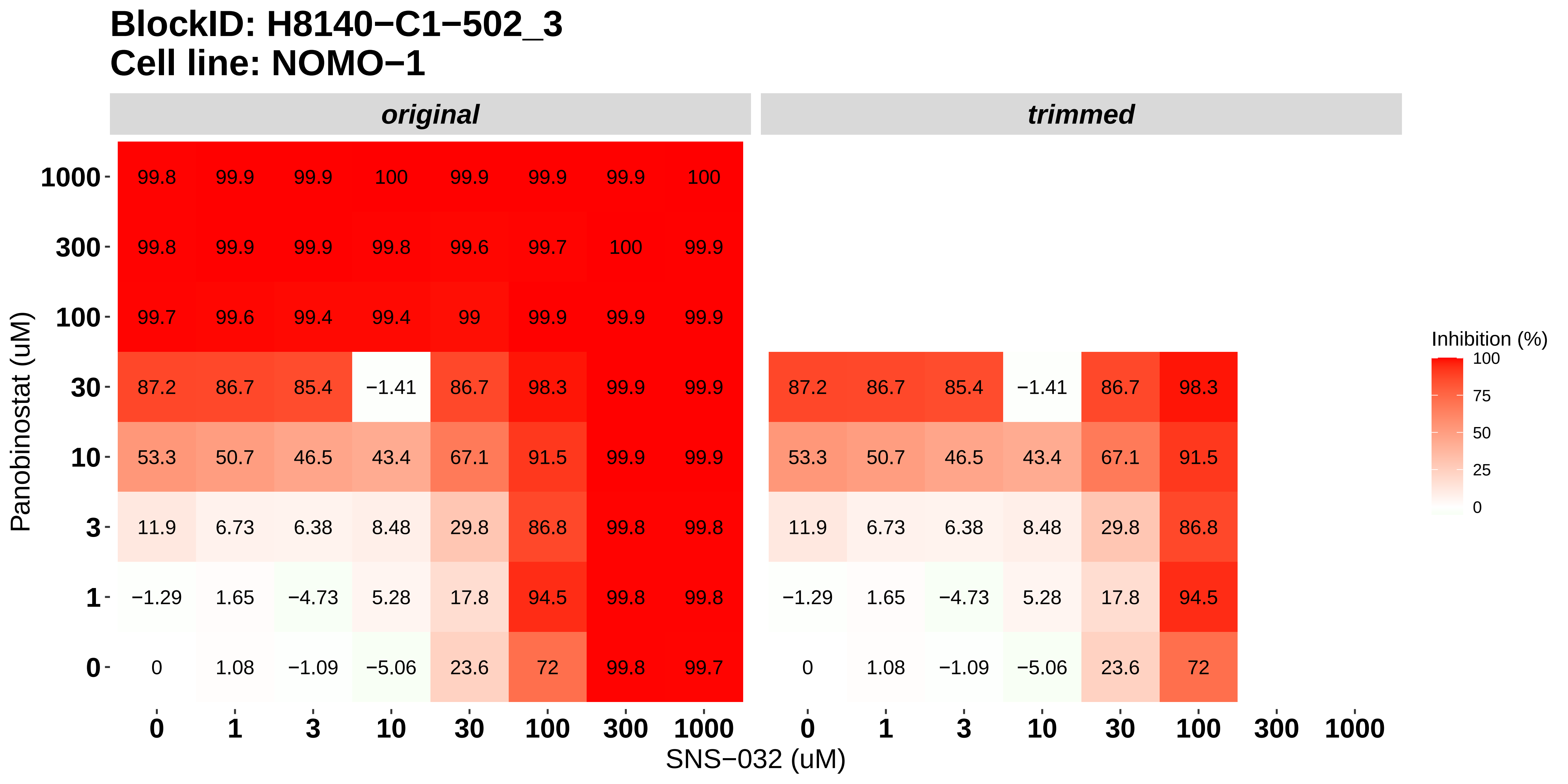

BlockID: H8140−C1−502_3
Cell line: NOMO−1
original
trimmed
Panobinostat (uM)
SNS−032 (uM)
Inhibition (%)
100
75
50
25
0
1000 99.8 99.9 99.9 100 99.9 99.9 99.9 100
300 99.8 99.9 99.9 99.8 99.6 99.7 100 99.9
100 99.7 99.6 99.4 99.4 99 99.9 99.9 99.9
30 87.2 86.7 85.4 −1.41 86.7 98.3 99.9 99.9
10 53.3 50.7 46.5 43.4 67.1 91.5 99.9 99.9
3 11.9 6.73 6.38 8.48 29.8 86.8 99.8 99.8
1 −1.29 1.65 −4.73 5.28 17.8 94.5 99.8 99.8
0 0 1.08 −1.09 −5.06 23.6 72 99.8 99.7
0 1 3 10 30 100 300 1000
87.2 86.7 85.4 −1.41 86.7 98.3
53.3 50.7 46.5 43.4 67.1 91.5
11.9 6.73 6.38 8.48 29.8 86.8
−1.29 1.65 −4.73 5.28 17.8 94.5
0 1.08 −1.09 −5.06 23.6 72
0 1 3 10 30 100 300 1000

BlockID: H8140−C1−502_4
Cell line: NOMO−1

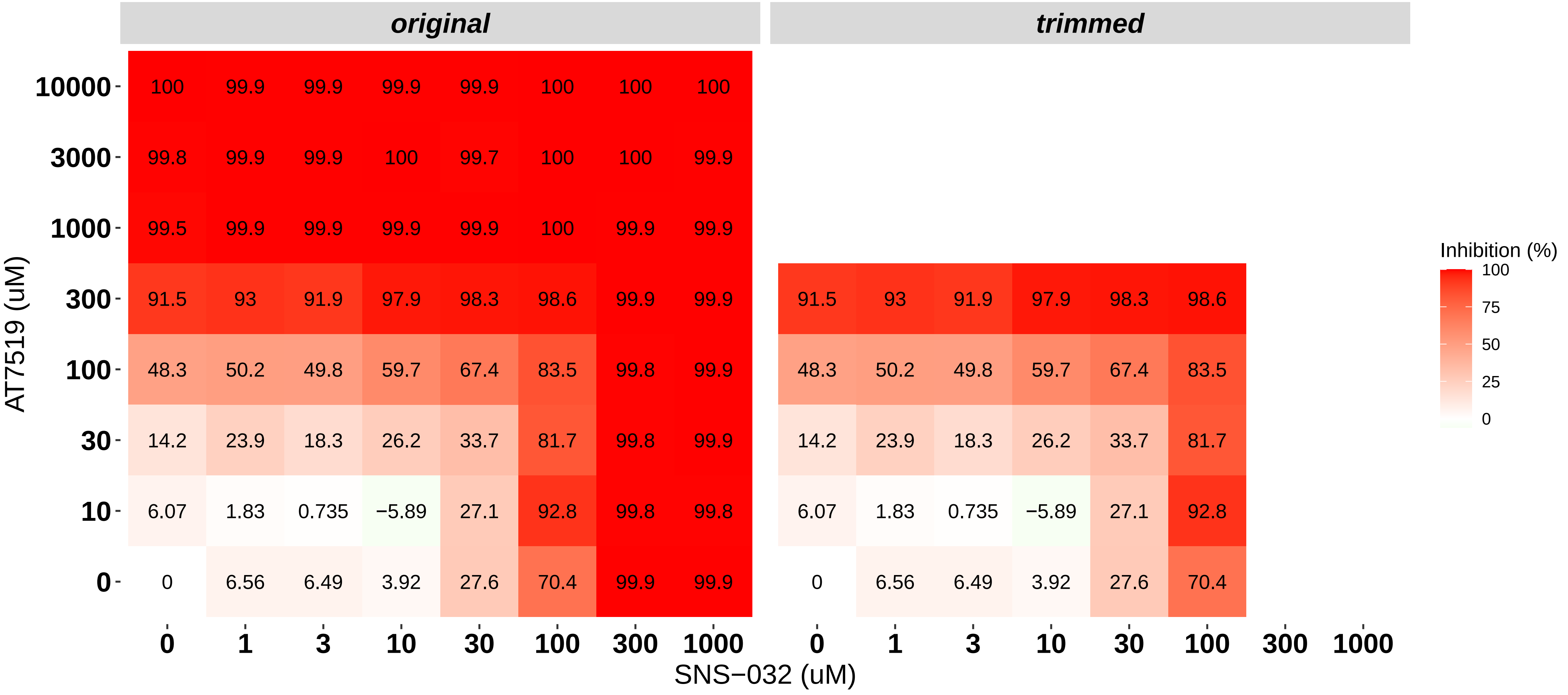

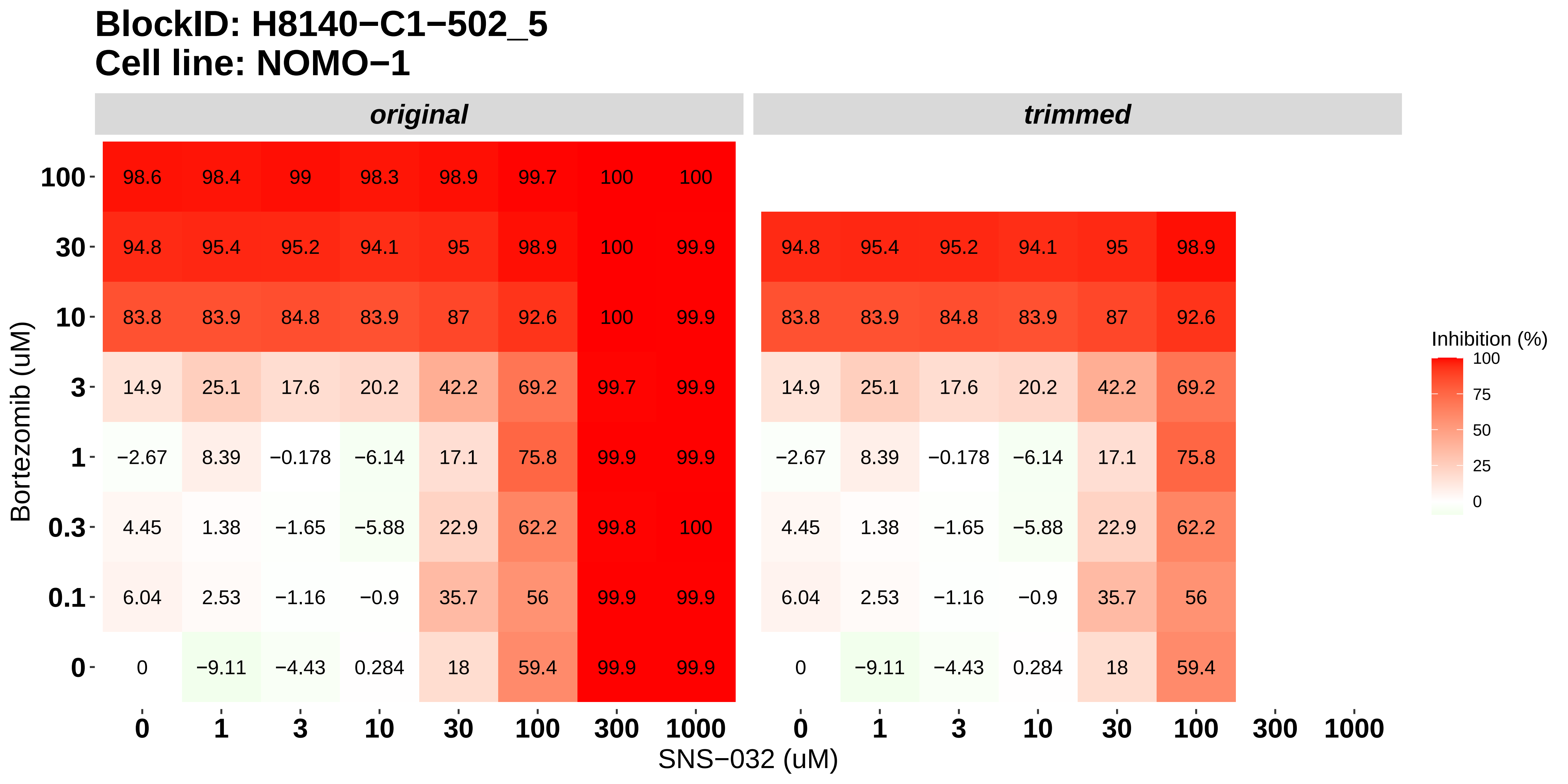
BlockID: H8140−C1−502_5
Cell line: NOMO−1
original
trimmed
Bortezomib (uM)
SNS−032 (uM)
Inhibition (%)

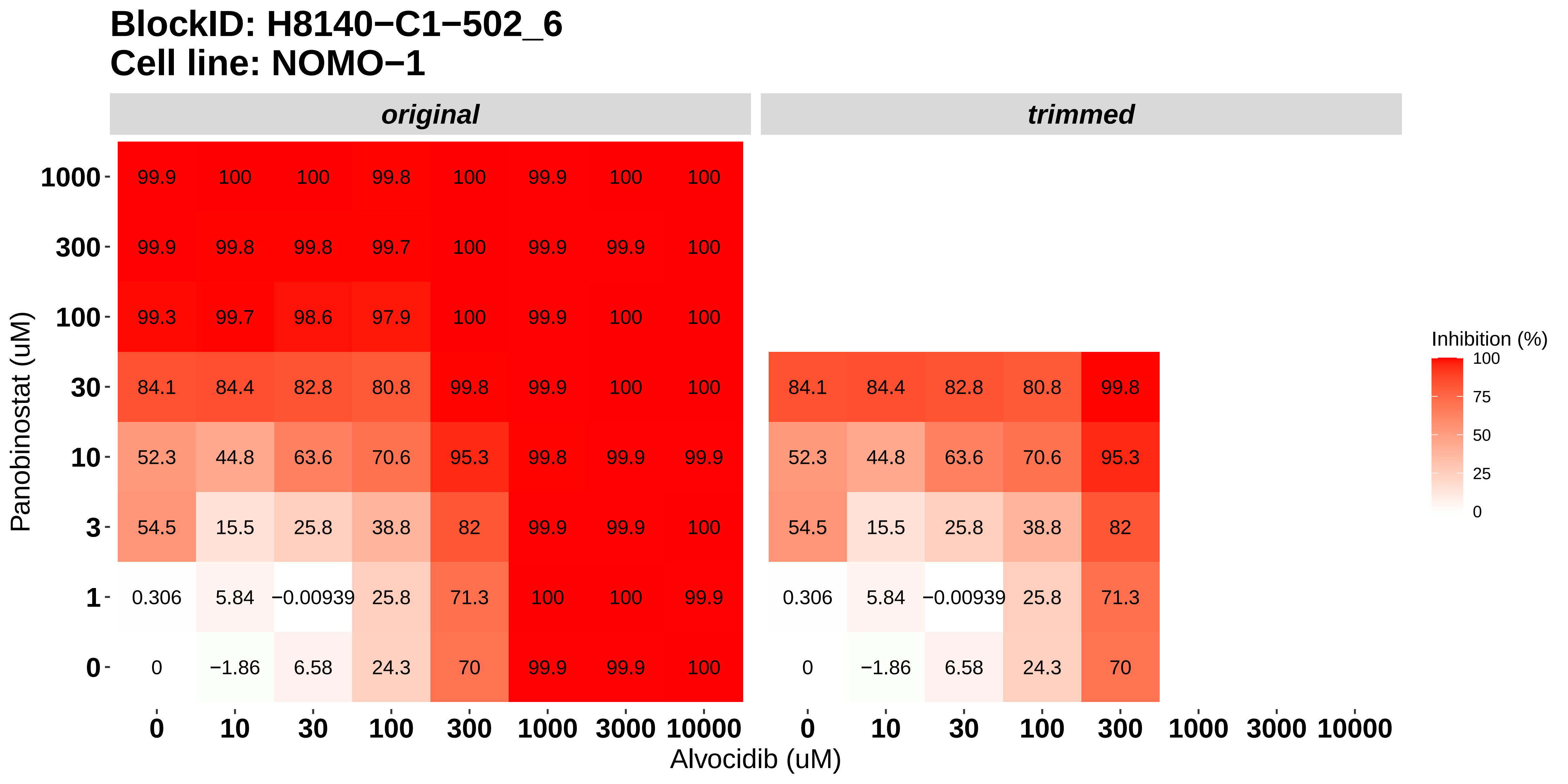

BlockID: H8140−C1−502_6
Cell line: NOMO−1
original
trimmed
Panobinostat (uM)
1000
300
100
30
10
3
1
0
Alvocidib (uM)
0
10
30
100
300
1000
3000
10000
Inhibition (%)
100
75
50
25
0
99.9 100 100 99.8 100 99.9 100 100
99.9 99.8 99.8 99.7 100 99.9 99.9 100
99.3 99.7 98.6 97.9 100 99.9 100 100
84.1 84.4 82.8 80.8 99.8 99.9 100 100
52.3 44.8 63.6 70.6 95.3 99.8 99.9 99.9
54.5 15.5 25.8 38.8 82 99.9 99.9 100
0.306 5.84 −0.00939 25.8 71.3 100 100 99.9
0 −1.86 6.58 24.3 70 99.9 99.9 100
84.1 84.4 82.8 80.8 99.8
52.3 44.8 63.6 70.6 95.3
54.5 15.5 25.8 38.8 82
0.306 5.84 −0.00939 25.8 71.3
0 −1.86 6.58 24.3 70

BlockID: H8140−C1−503_1
Cell line: OCI−AML3

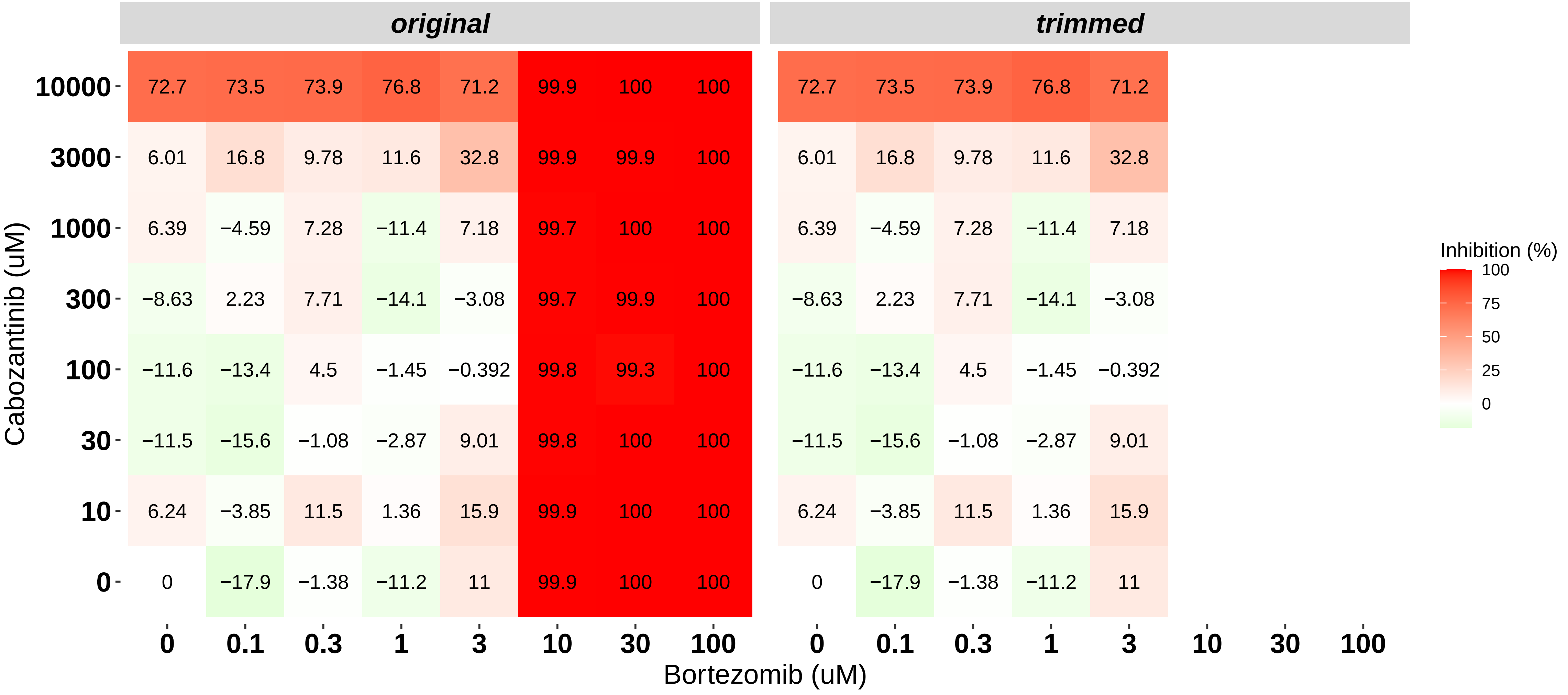

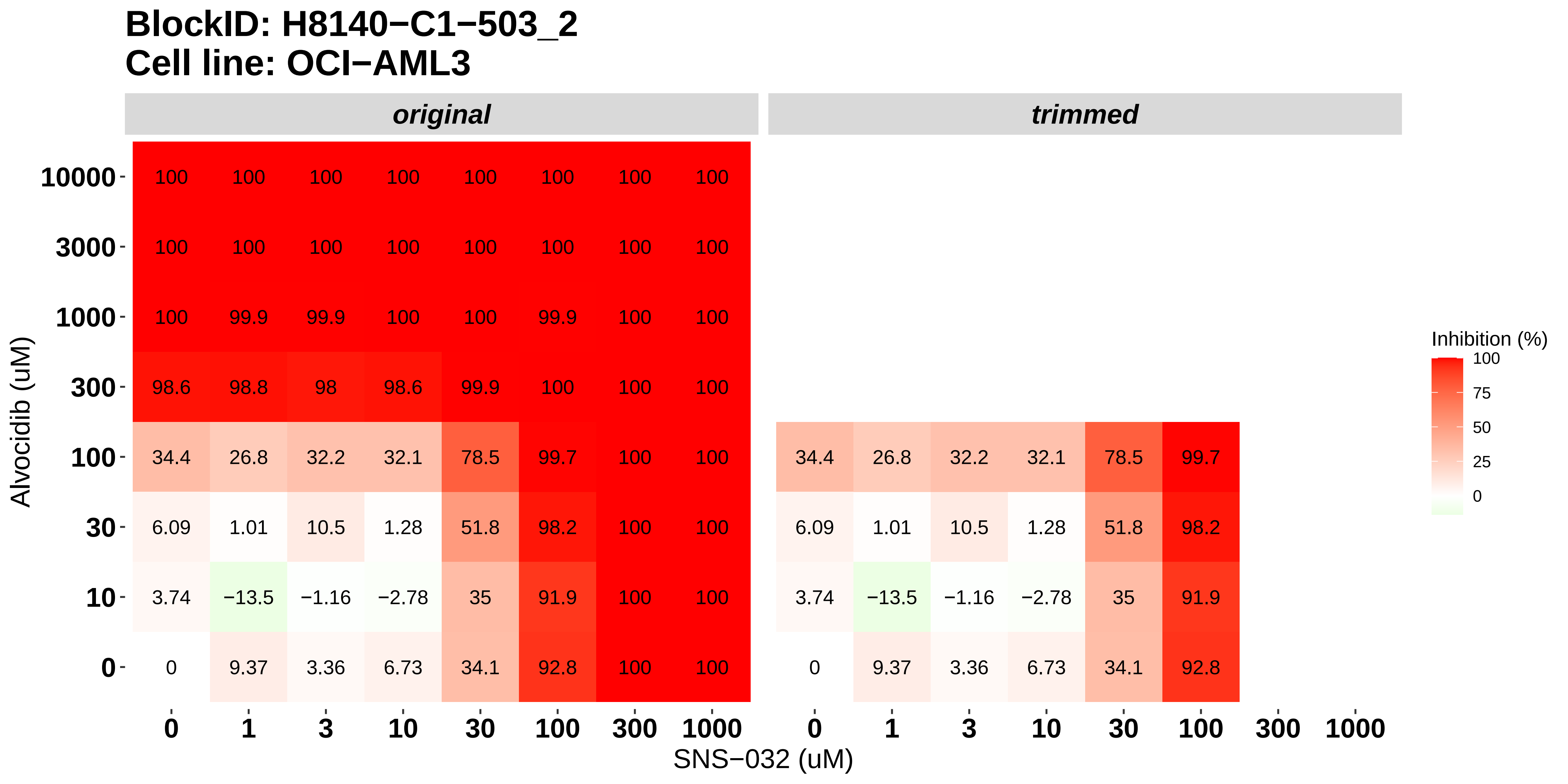

BlockID: H8140−C1−503_2
Cell line: OCI−AML3
original
trimmed
Alvocidib (uM)
SNS−032 (uM)
Inhibition (%)
10000
3000
1000
300
100
30
10
0
1
3
10
30
100
300
1000
100 100 100 100 100 100 100 100
100 100 100 100 100 100 100 100
100 99.9 99.9 100 100 99.9 100 100
98.6 98.8 98 98.6 99.9 100 100 100
34.4 26.8 32.2 32.1 78.5 99.7 100 100
6.09 1.01 10.5 1.28 51.8 98.2 100 100
3.74 −13.5 −1.16 −2.78 35 91.9 100 100
0 9.37 3.36 6.73 34.1 92.8 100 100
34.4 26.8 32.2 32.1 78.5 99.7
6.09 1.01 10.5 1.28 51.8 98.2
3.74 −13.5 −1.16 −2.78 35 91.9
0 9.37 3.36 6.73 34.1 92.8
100
75
50
25
0

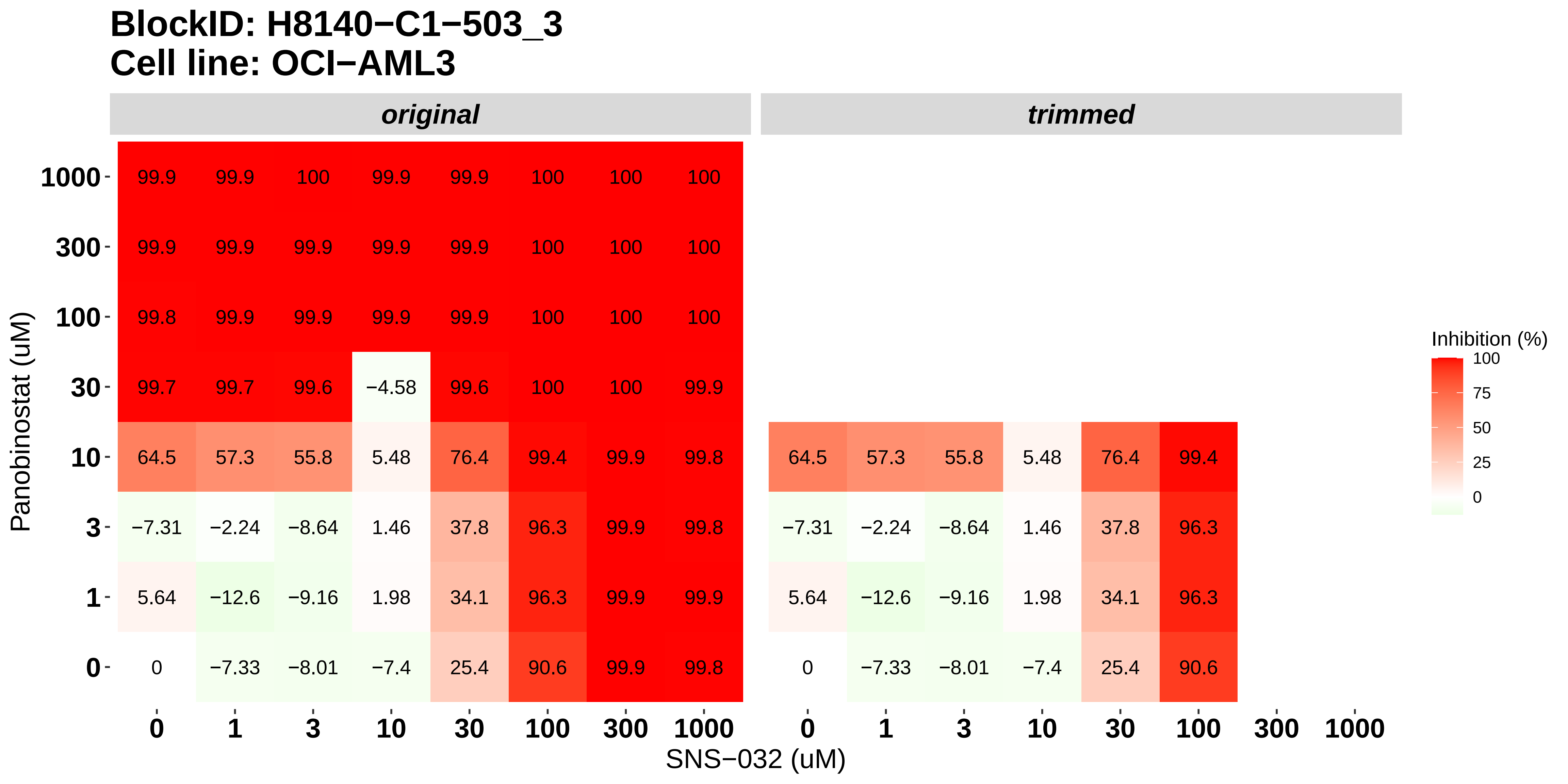
BlockID: H8140−C1−503_3
Cell line: OCI−AML3
original
trimmed
Panobinostat (uM)
SNS−032 (uM)
Inhibition (%)
1000
300
100
30
10
3
1
0
99.9 99.9 100 99.9 99.9 100 100 100
99.9 99.9 99.9 99.9 99.9 100 100 100
99.8 99.9 99.9 99.9 99.9 100 100 100
99.7 99.7 99.6 −4.58 99.6 100 100 99.9
64.5 57.3 55.8 5.48 76.4 99.4 99.9 99.8
−7.31 −2.24 −8.64 1.46 37.8 96.3 99.9 99.8
5.64 −12.6 −9.16 1.98 34.1 96.3 99.9 99.9
0 −7.33 −8.01 −7.4 25.4 90.6 99.9 99.8
64.5 57.3 55.8 5.48 76.4 99.4
−7.31 −2.24 −8.64 1.46 37.8 96.3
5.64 −12.6 −9.16 1.98 34.1 96.3
0 −7.33 −8.01 −7.4 25.4 90.6
0 1 3 10 30 100 300 1000
100
75
50
25
0

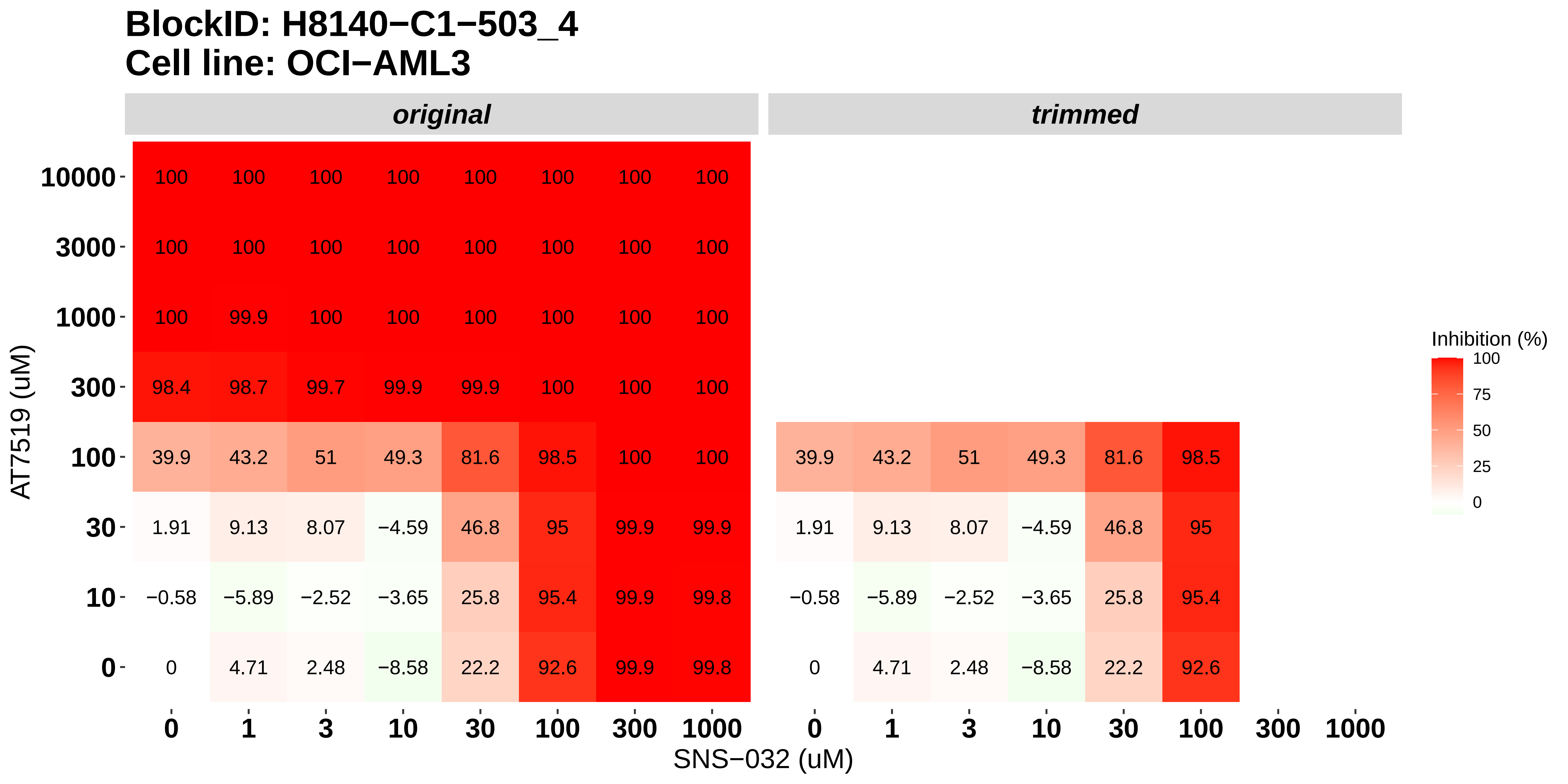
BlockID: H8140−C1−503_4
Cell line: OCI−AML3
original
trimmed
AT7519 (uM)
SNS−032 (uM)
Inhibition (%)
10000
3000
1000
300
100
30
10
0
0
1
3
10
30
100
300
1000
100 100 100 100 100 100 100 100
100 100 100 100 100 100 100 100
100 99.9 100 100 100 100 100 100
98.4 98.7 99.7 99.9 99.9 100 100 100
39.9 43.2 51 49.3 81.6 98.5 100 100
1.91 9.13 8.07 −4.59 46.8 95 99.9 99.9
−0.58 −5.89 −2.52 −3.65 25.8 95.4 99.9 99.8
0 4.71 2.48 −8.58 22.2 92.6 99.9 99.8
39.9 43.2 51 49.3 81.6 98.5
1.91 9.13 8.07 −4.59 46.8 95
−0.58 −5.89 −2.52 −3.65 25.8 95.4
0 4.71 2.48 −8.58 22.2 92.6
100
75
50
25
0

BlockID: H8140−C1−503_5
Cell line: OCI−AML3

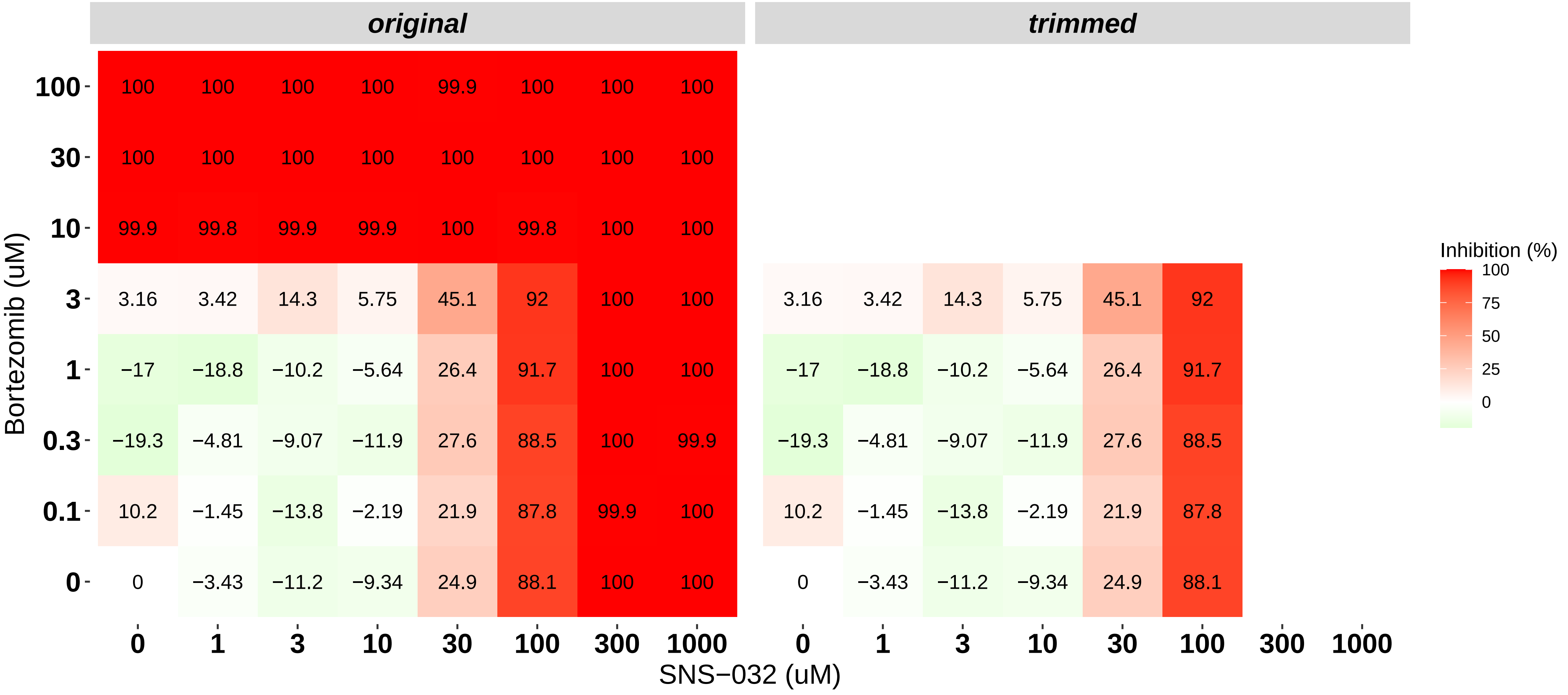

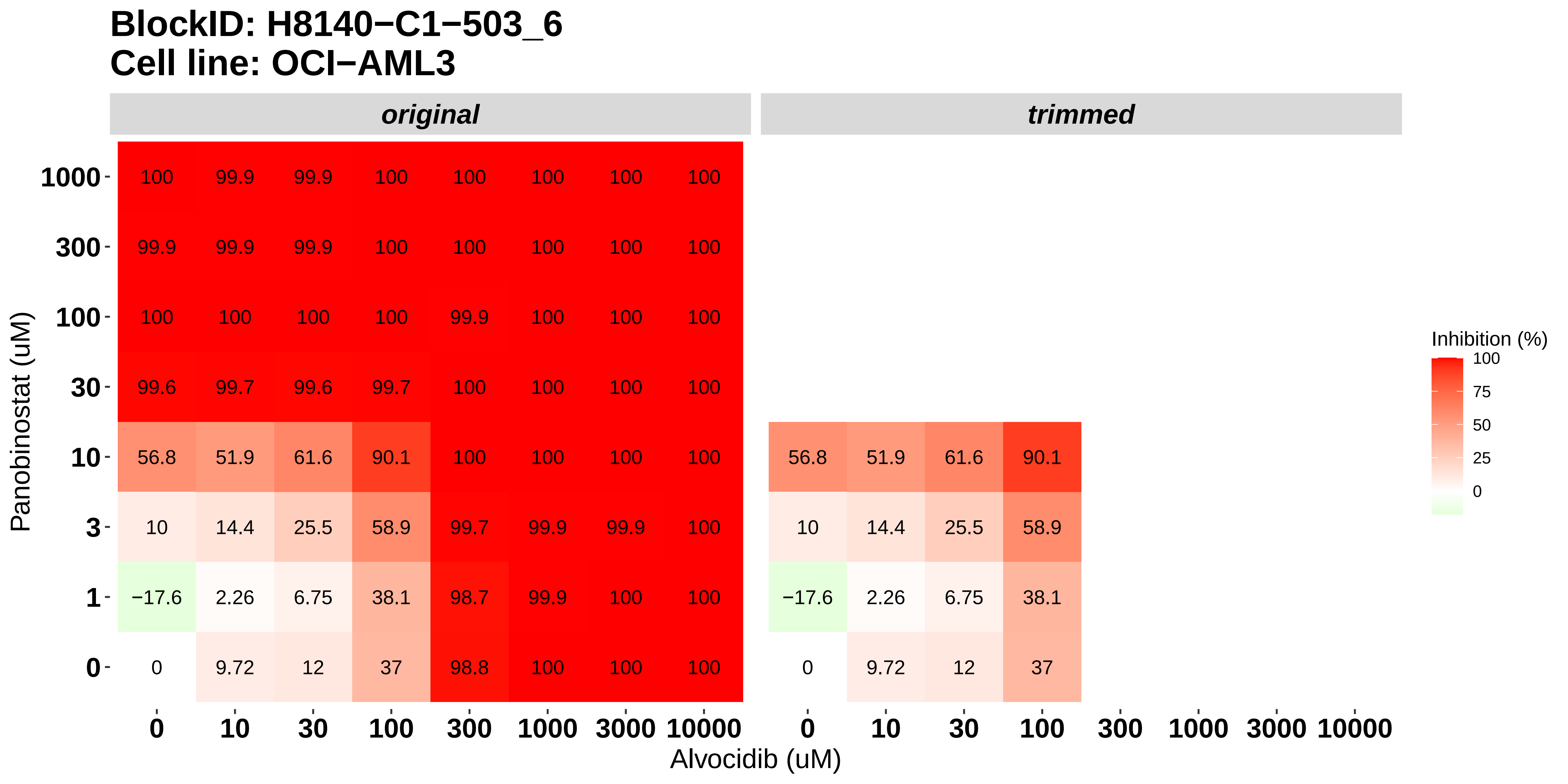
BlockID: H8140−C1−503_6
Cell line: OCI−AML3
original
trimmed
Panobinostat (uM)
Alvocidib (uM)
Inhibition (%)
1000
300
100
30
10
3
1
0
10
30
100
300
1000
3000
10000
100
99.9
99.6
99.7
56.8
51.9
61.6
90.1
14.4
25.5
58.9
98.7
−17.6
2.26
6.75
38.1
9.72
12
37
98.8
75
50
25

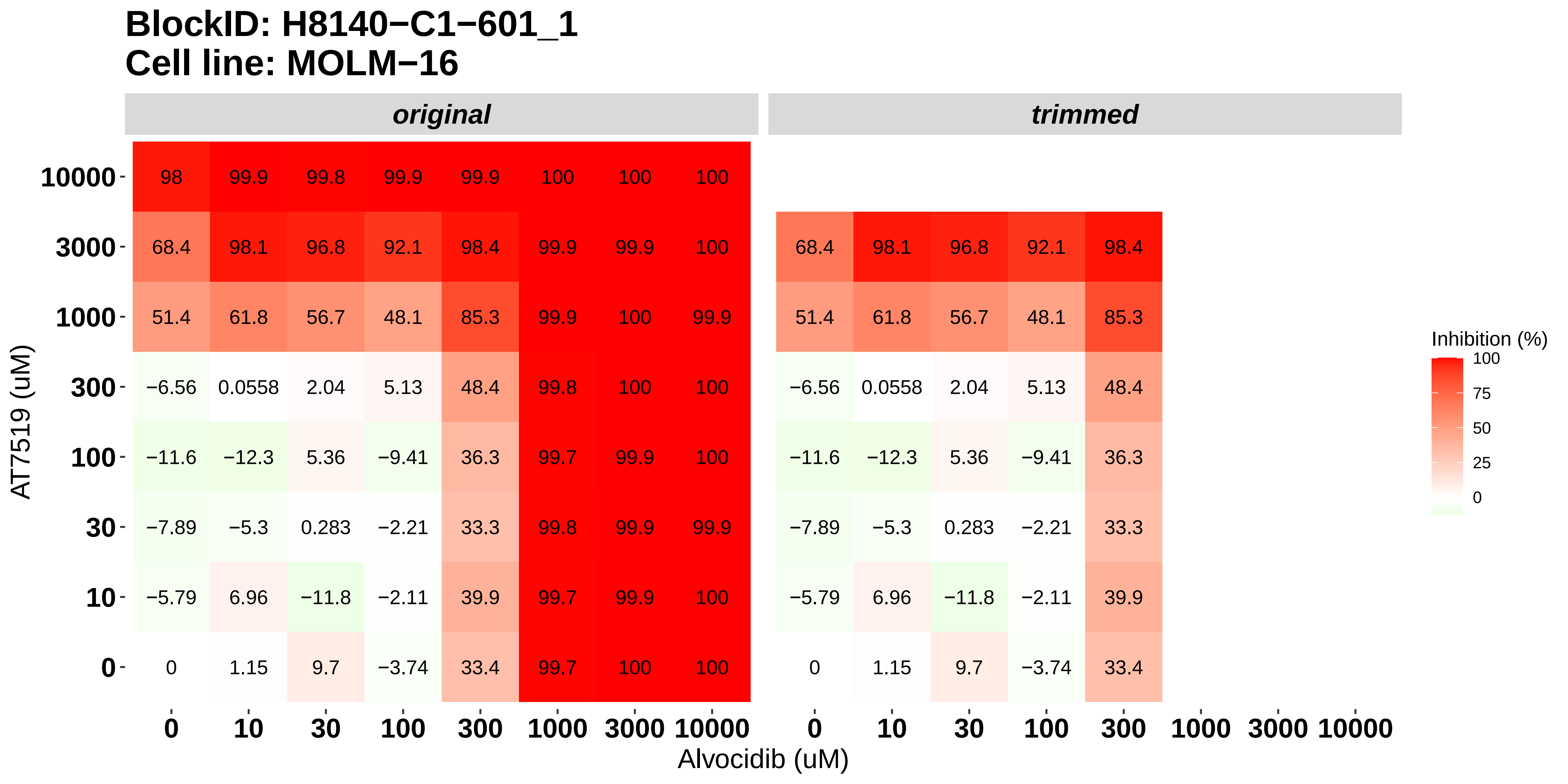

BlockID: H8140−C1−601_1
Cell line: MOLM−16
original
trimmed
AT7519 (uM)
Alvocidib (uM)
Inhibition (%)

BlockID: H8140−C1−601_2
Cell line: MOLM−16

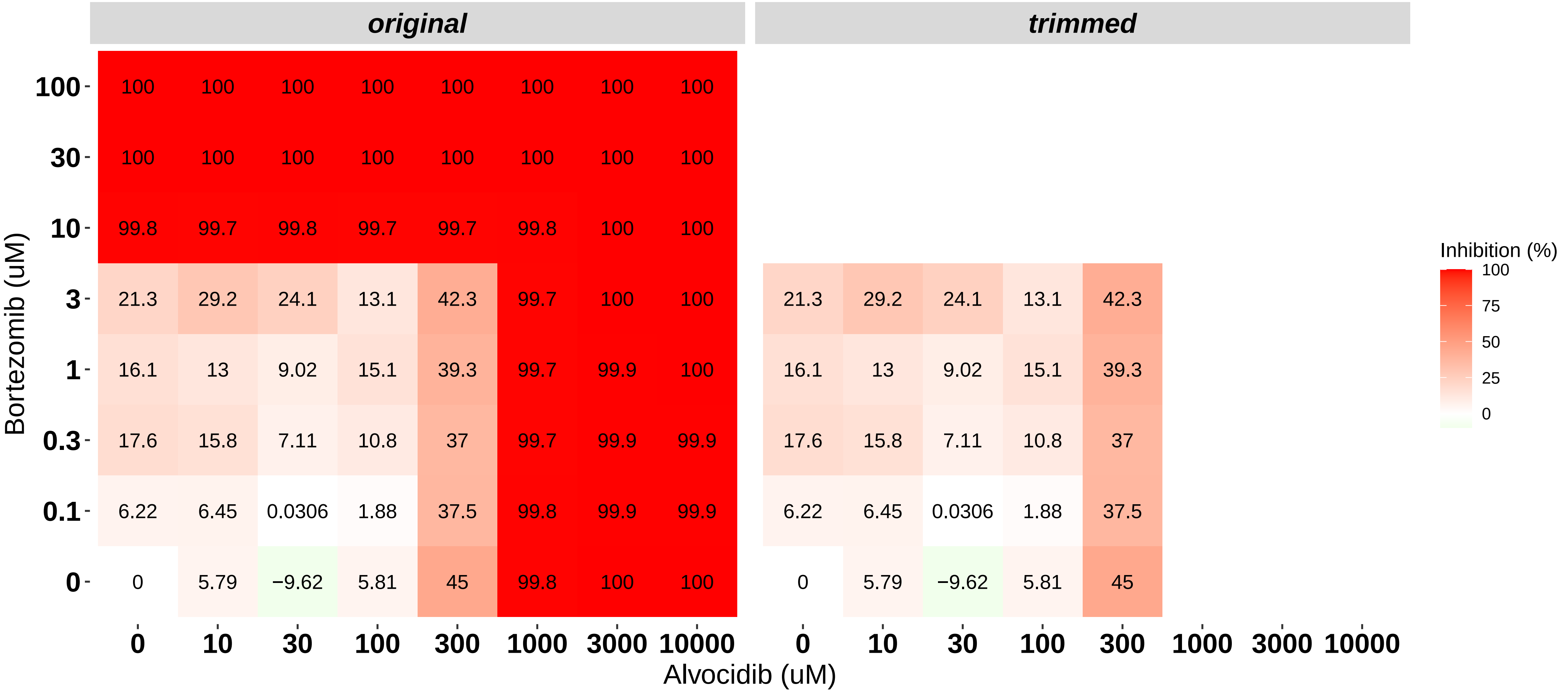

Bortezomib (uM) \ Alvocidib (uM)	0	10	30	100	300	1000	3000	10000
100	100	100	100	100	100	100	100	100
30	100	100	100	100	100	100	100	100
10	99.8	99.7	99.8	99.7	99.7	99.8	100	100
3	21.3	29.2	24.1	13.1	42.3	99.7	100	100
1	16.1	13	9.02	15.1	39.3	99.7	99.9	100
0.3	17.6	15.8	7.11	10.8	37	99.7	99.9	99.9
0.1	6.22	6.45	0.0306	1.88	37.5	99.8	99.9	99.9
0	0	5.79	−9.62	5.81	45	99.8	100	100

Bortezomib (uM) \ Alvocidib (uM)	0	10	30	100	300	1000	3000	10000
3	21.3	29.2	24.1	13.1	42.3			
1	16.1	13	9.02	15.1	39.3			
0.3	17.6	15.8	7.11	10.8	37			
0.1	6.22	6.45	0.0306	1.88	37.5			
0	0	5.79	−9.62	5.81	45			

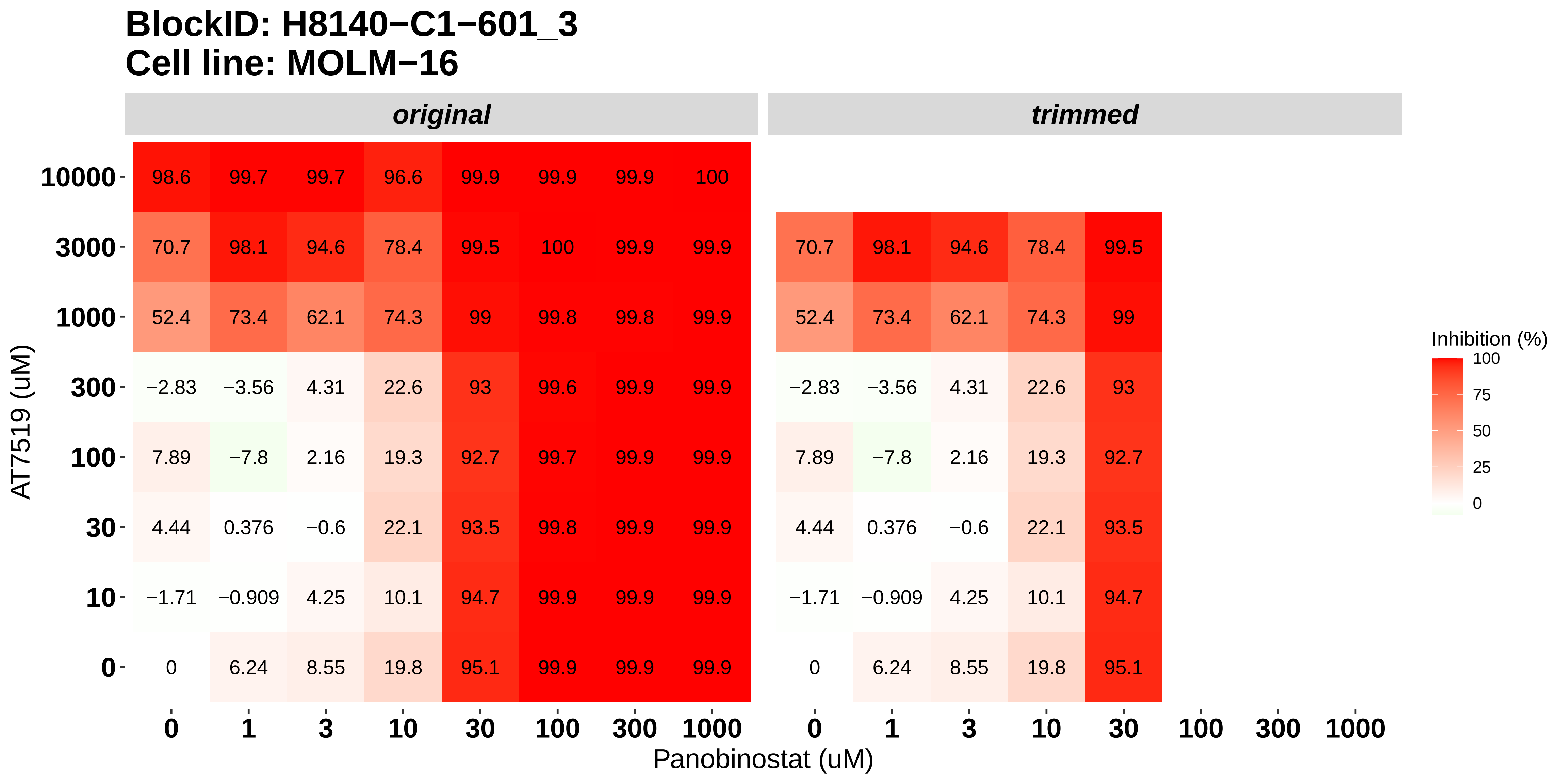

BlockID: H8140−C1−601_3
Cell line: MOLM−16
original
trimmed
AT7519 (uM)
Panobinostat (uM)
Inhibition (%)
100
75
50
25
0
10000
3000
1000
300
100
30
10
0
0
1
3
10
30
100
300
1000
98.6 99.7 99.7 96.6 99.9 99.9 99.9 100
70.7 98.1 94.6 78.4 99.5 100 99.9 99.9
52.4 73.4 62.1 74.3 99 99.8 99.8 99.9
−2.83 −3.56 4.31 22.6 93 99.6 99.9 99.9
7.89 −7.8 2.16 19.3 92.7 99.7 99.9 99.9
4.44 0.376 −0.6 22.1 93.5 99.8 99.9 99.9
−1.71 −0.909 4.25 10.1 94.7 99.9 99.9 99.9
0 6.24 8.55 19.8 95.1 99.9 99.9 99.9
70.7 98.1 94.6 78.4 99.5
52.4 73.4 62.1 74.3 99
−2.83 −3.56 4.31 22.6 93
7.89 −7.8 2.16 19.3 92.7
4.44 0.376 −0.6 22.1 93.5
−1.71 −0.909 4.25 10.1 94.7
0 6.24 8.55 19.8 95.1

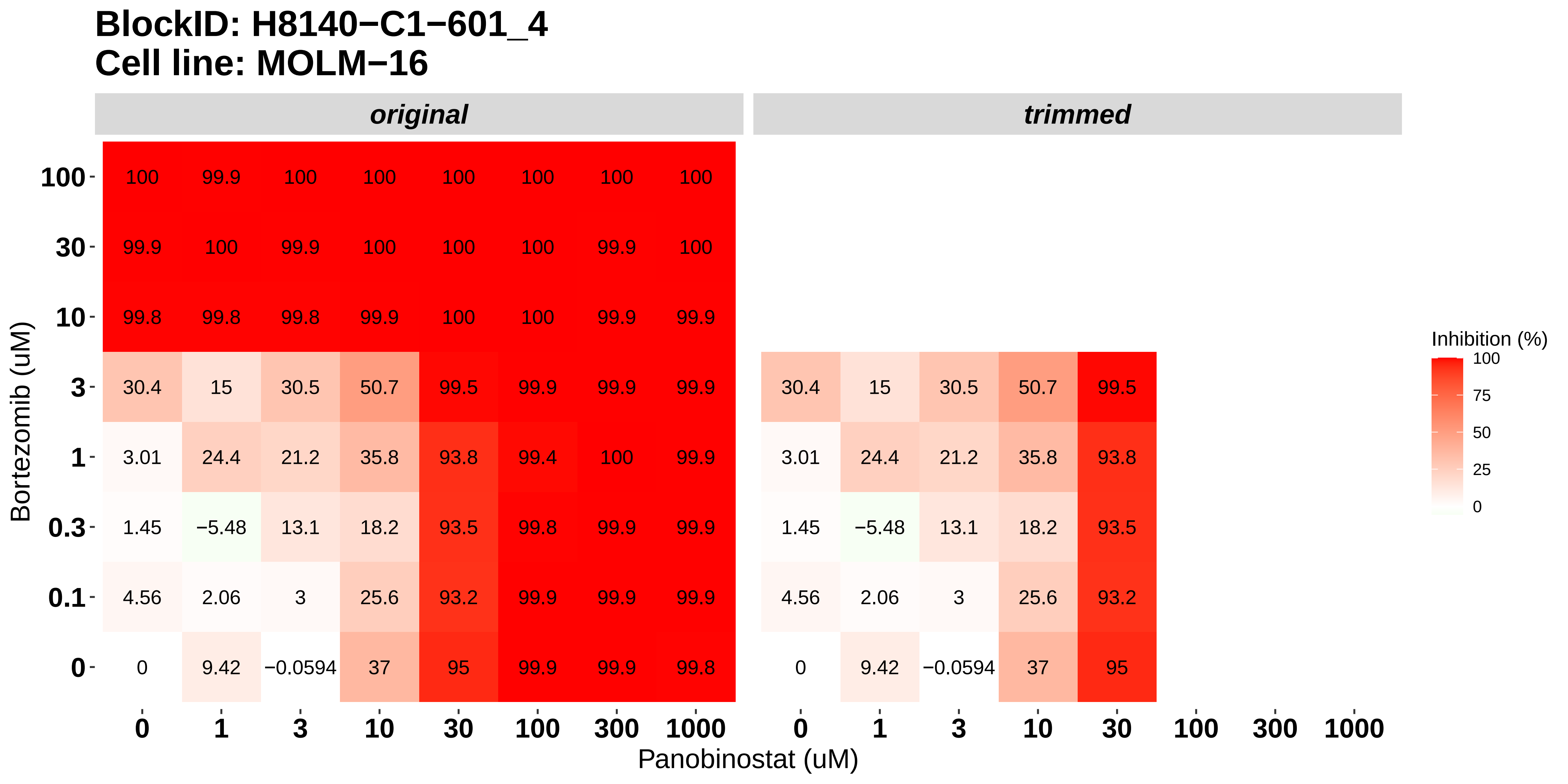

BlockID: H8140−C1−601_4
Cell line: MOLM−16
original
trimmed
Bortezomib (uM)
Panobinostat (uM)
Inhibition (%)
100
75
50
25
0
100 30 10 3 1 0.3 0.1 0
0 1 3 10 30 100 300 1000
100 99.9 100 100 100 100 100 100
99.9 100 99.9 100 100 100 99.9 100
99.8 99.8 99.8 99.9 100 100 99.9 99.9
30.4 15 30.5 50.7 99.5 99.9 99.9 99.9
3.01 24.4 21.2 35.8 93.8 99.4 100 99.9
1.45 −5.48 13.1 18.2 93.5 99.8 99.9 99.9
4.56 2.06 3 25.6 93.2 99.9 99.9 99.9
0 9.42 −0.0594 37 95 99.9 99.9 99.8
30.4 15 30.5 50.7 99.5
3.01 24.4 21.2 35.8 93.8
1.45 −5.48 13.1 18.2 93.5
4.56 2.06 3 25.6 93.2
0 9.42 −0.0594 37 95

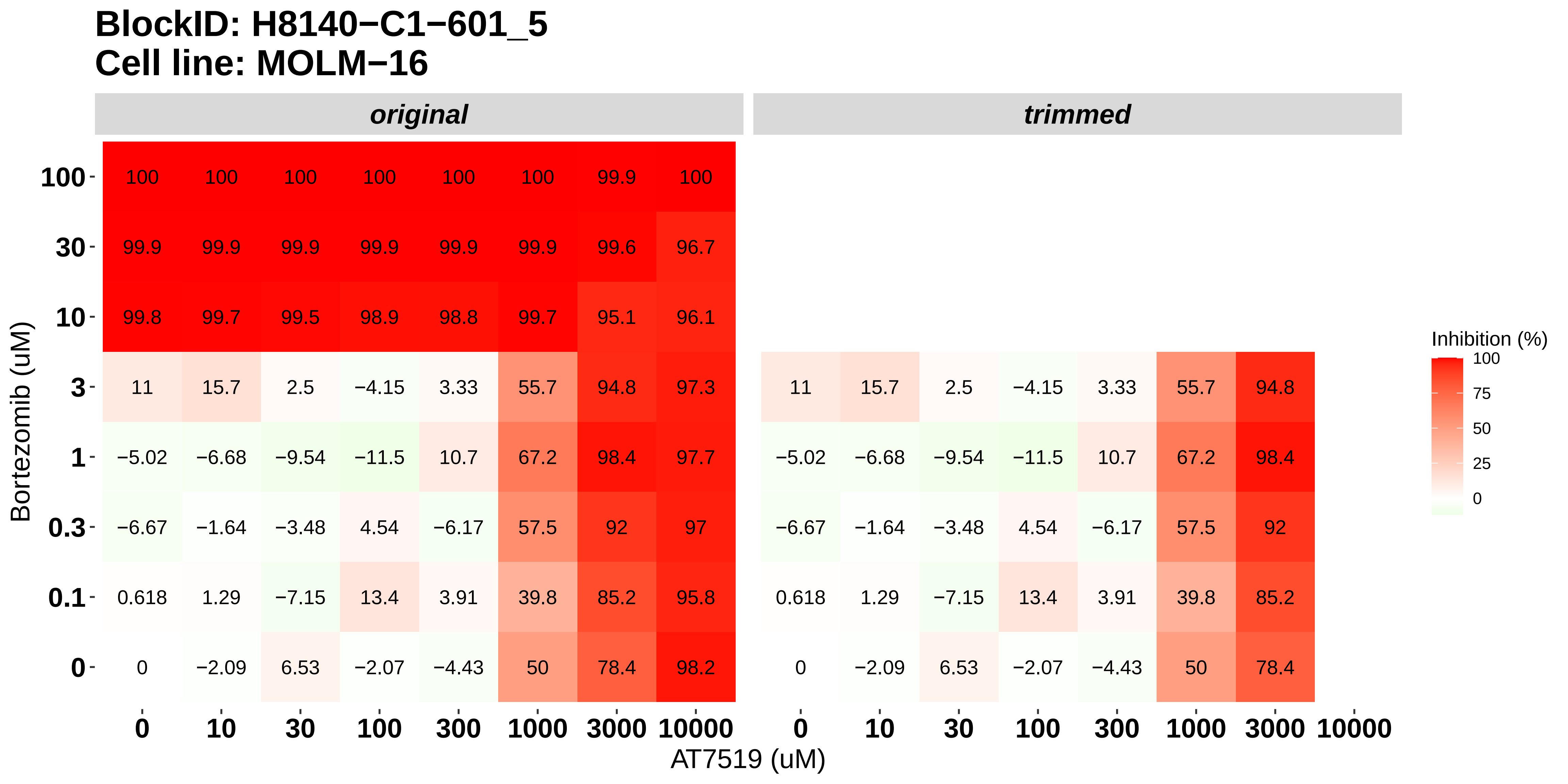

BlockID: H8140−C1−601_5
Cell line: MOLM−16
original
trimmed
Bortezomib (uM)
AT7519 (uM)
Inhibition (%)
100
30
10
3
1
0.3
0.1
0
10
30
100
300
1000
3000
10000
75
50
25
100 100 100 100 100 100 99.9 100
99.9 99.9 99.9 99.9 99.9 99.9 99.6 96.7
99.8 99.7 99.5 98.9 98.8 99.7 95.1 96.1
11 15.7 2.5 −4.15 3.33 55.7 94.8 97.3
−5.02 −6.68 −9.54 −11.5 10.7 67.2 98.4 97.7
−6.67 −1.64 −3.48 4.54 −6.17 57.5 92 97
0.618 1.29 −7.15 13.4 3.91 39.8 85.2 95.8
0 −2.09 6.53 −2.07 −4.43 50 78.4 98.2
11 15.7 2.5 −4.15 3.33 55.7 94.8
−5.02 −6.68 −9.54 −11.5 10.7 67.2 98.4
−6.67 −1.64 −3.48 4.54 −6.17 57.5 92
0.618 1.29 −7.15 13.4 3.91 39.8 85.2
0 −2.09 6.53 −2.07 −4.43 50 78.4

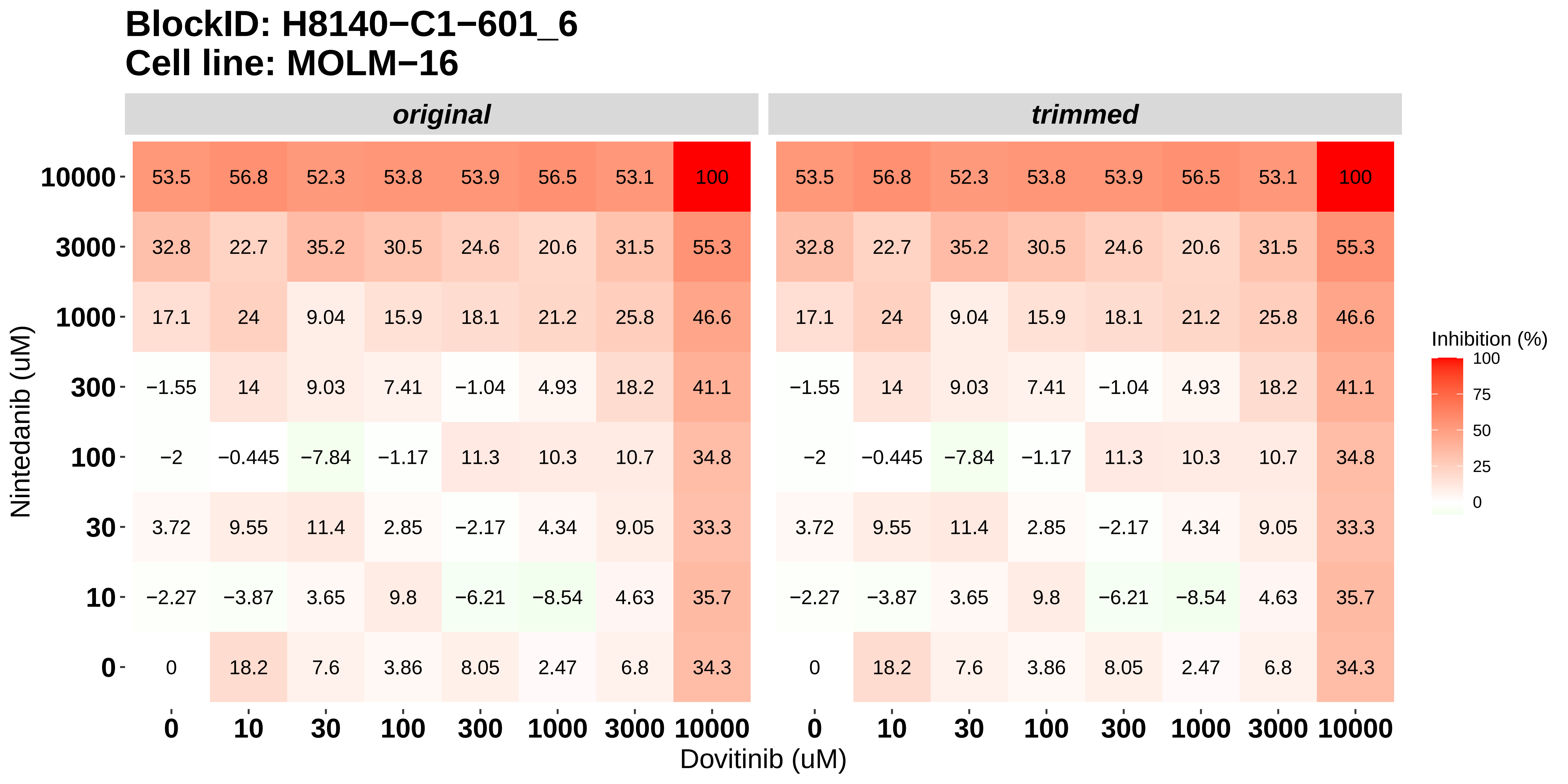
BlockID: H8140−C1−601_6
Cell line: MOLM−16
original
trimmed
Nintedanib (uM)
Dovitinib (uM)
Inhibition (%)
10000
3000
1000
300
100
30
10
0
53.5 56.8 52.3 53.8 53.9 56.5 53.1 100
32.8 22.7 35.2 30.5 24.6 20.6 31.5 55.3
17.1 24 9.04 15.9 18.1 21.2 25.8 46.6
−1.55 14 9.03 7.41 −1.04 4.93 18.2 41.1
−2 −0.445 −7.84 −1.17 11.3 10.3 10.7 34.8
3.72 9.55 11.4 2.85 −2.17 4.34 9.05 33.3
−2.27 −3.87 3.65 9.8 −6.21 −8.54 4.63 35.7
0 18.2 7.6 3.86 8.05 2.47 6.8 34.3
0 10 30 100 300 1000 3000 10000
100
75
50
25
0

BlockID: H8140−C1−602_1
Cell line: NOMO−1

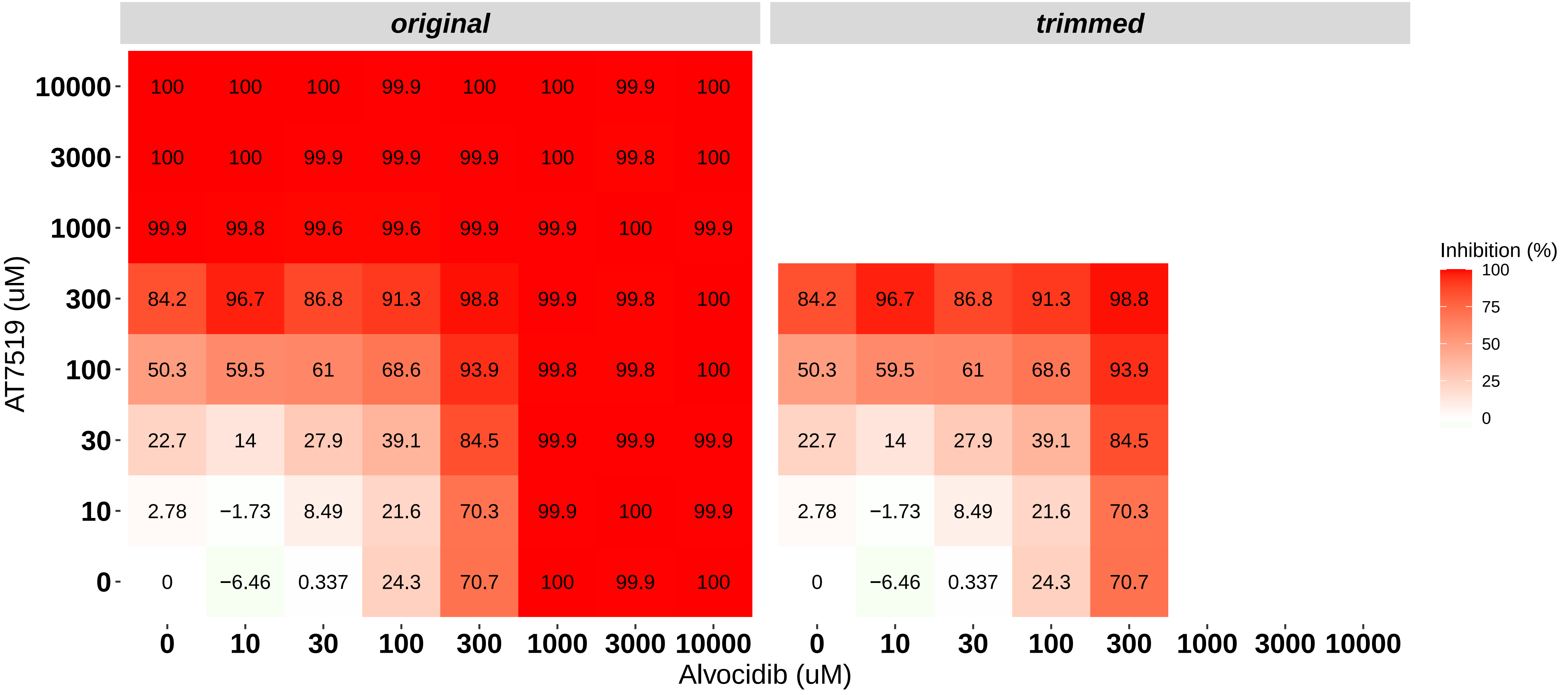

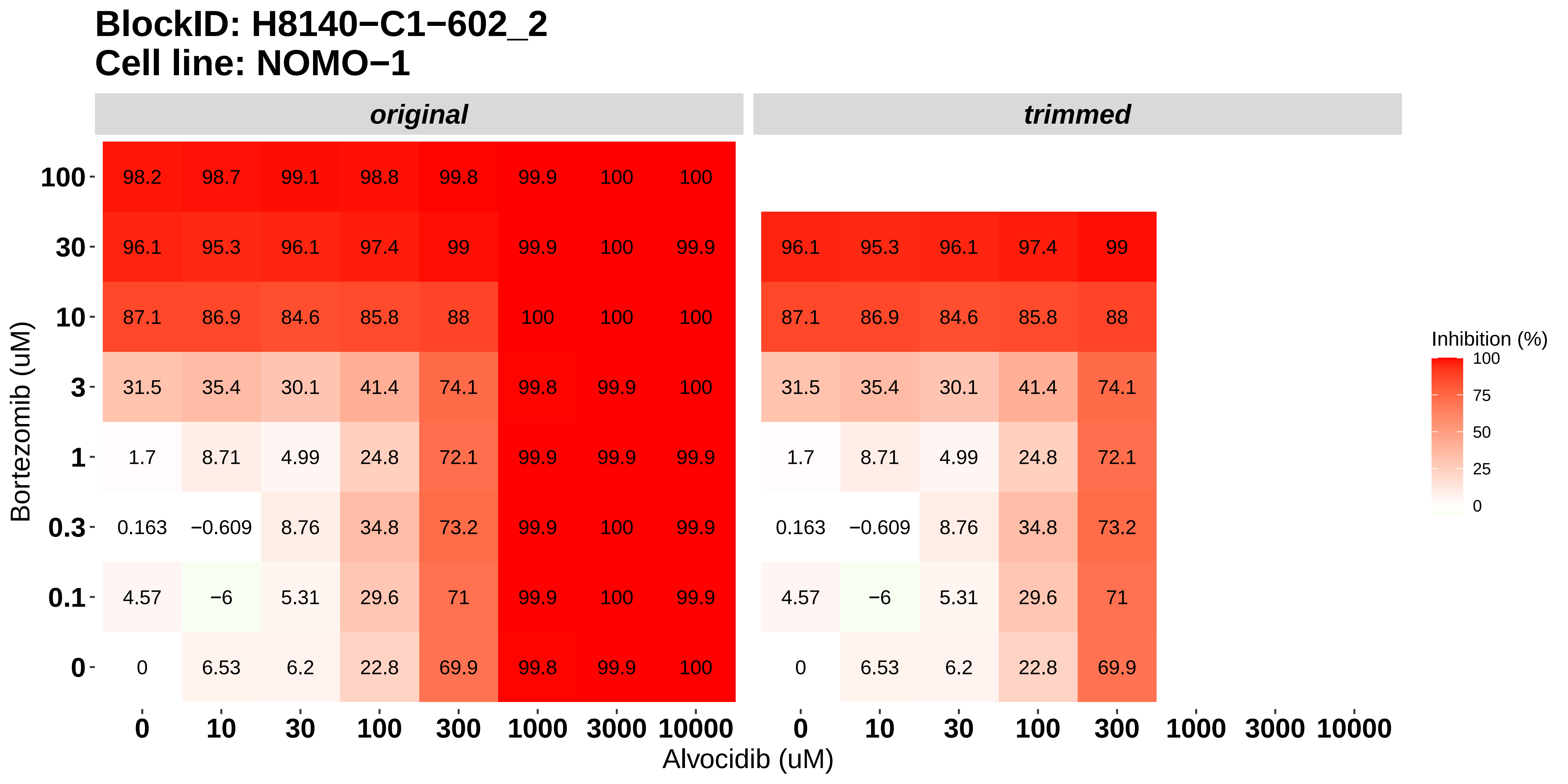
BlockID: H8140−C1−602_2
Cell line: NOMO−1
original
trimmed
98.2 98.7 99.1 98.8 99.8 99.9 100 100
96.1 95.3 96.1 97.4 99 99.9 100 99.9
87.1 86.9 84.6 85.8 88 100 100 100
31.5 35.4 30.1 41.4 74.1 99.8 99.9 100
1.7 8.71 4.99 24.8 72.1 99.9 99.9 99.9
0.163 −0.609 8.76 34.8 73.2 99.9 100 99.9
4.57 −6 5.31 29.6 71 99.9 100 99.9
0 6.53 6.2 22.8 69.9 99.8 99.9 100
96.1 95.3 96.1 97.4 99
87.1 86.9 84.6 85.8 88
31.5 35.4 30.1 41.4 74.1
1.7 8.71 4.99 24.8 72.1
0.163 −0.609 8.76 34.8 73.2
4.57 −6 5.31 29.6 71
0 6.53 6.2 22.8 69.9
100
30
10
3
1
0.3
0.1
0
Bortezomib (uM)
0 10 30 100 300 1000 3000 10000
0 10 30 100 300 1000 3000 10000
Alvocidib (uM)
Inhibition (%)
100
75
50
25
0

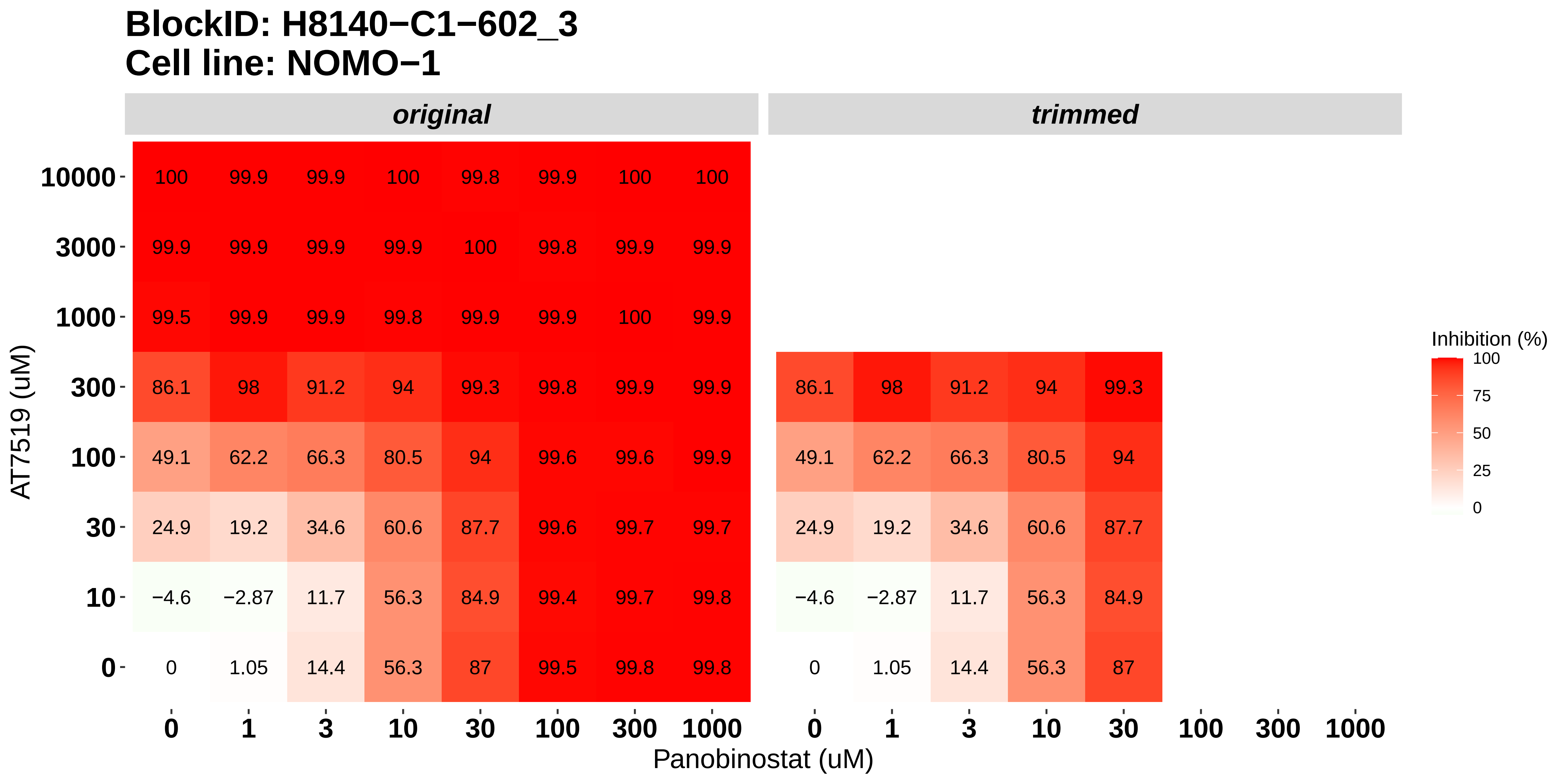
BlockID: H8140−C1−602_3
Cell line: NOMO−1
original
trimmed
AT7519 (uM)
Panobinostat (uM)
Inhibition (%)
10000
3000
1000
300
100
30
10
0
1
3
100
99.9
99.8
99.5
86.1
98
91.2
94
99.3
49.1
62.2
66.3
80.5
99.6
24.9
19.2
34.6
60.6
87.7
99.7
−4.6
−2.87
11.7
56.3
84.9
99.4
1.05
14.4
87
75
50
25

BlockID: H8140−C1−602_4
Cell line: NOMO−1

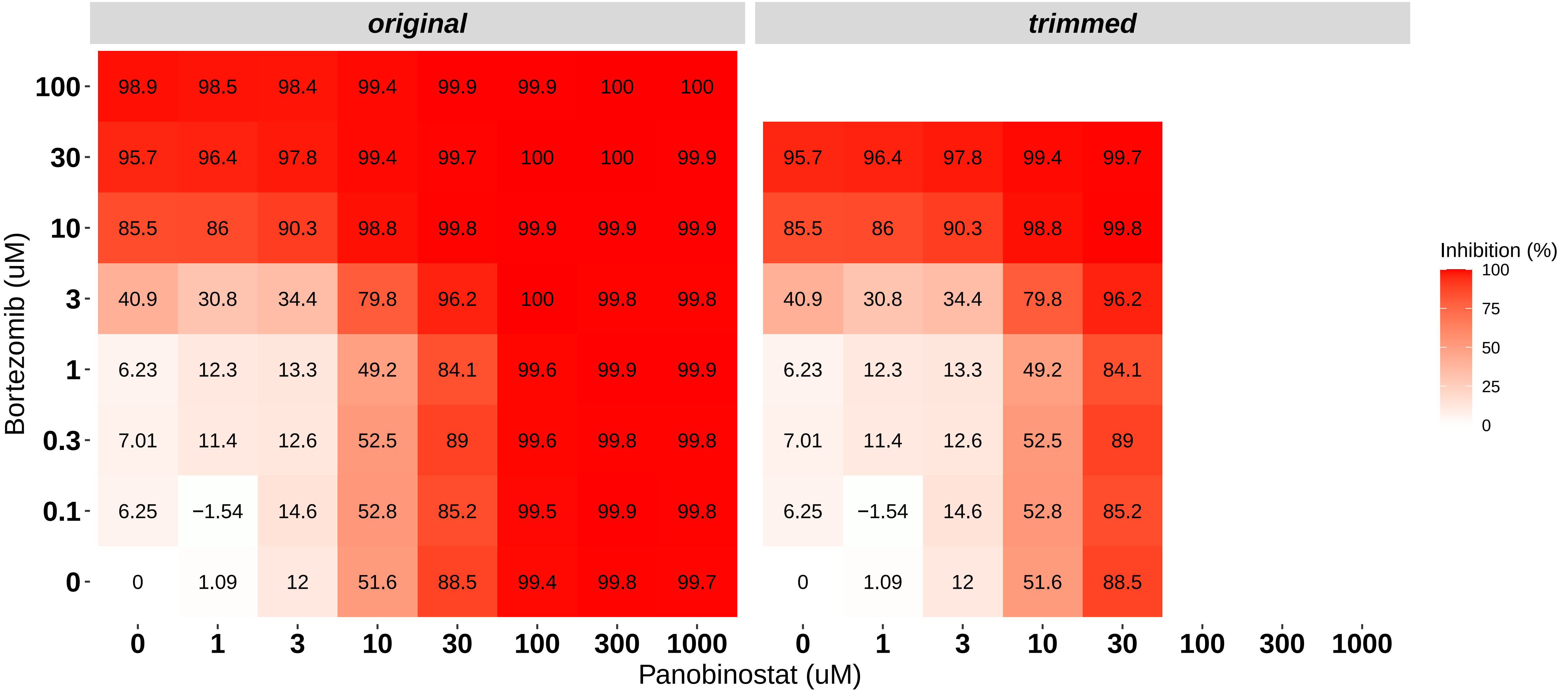

BlockID: H8140−C1−602_5
Cell line: NOMO−1

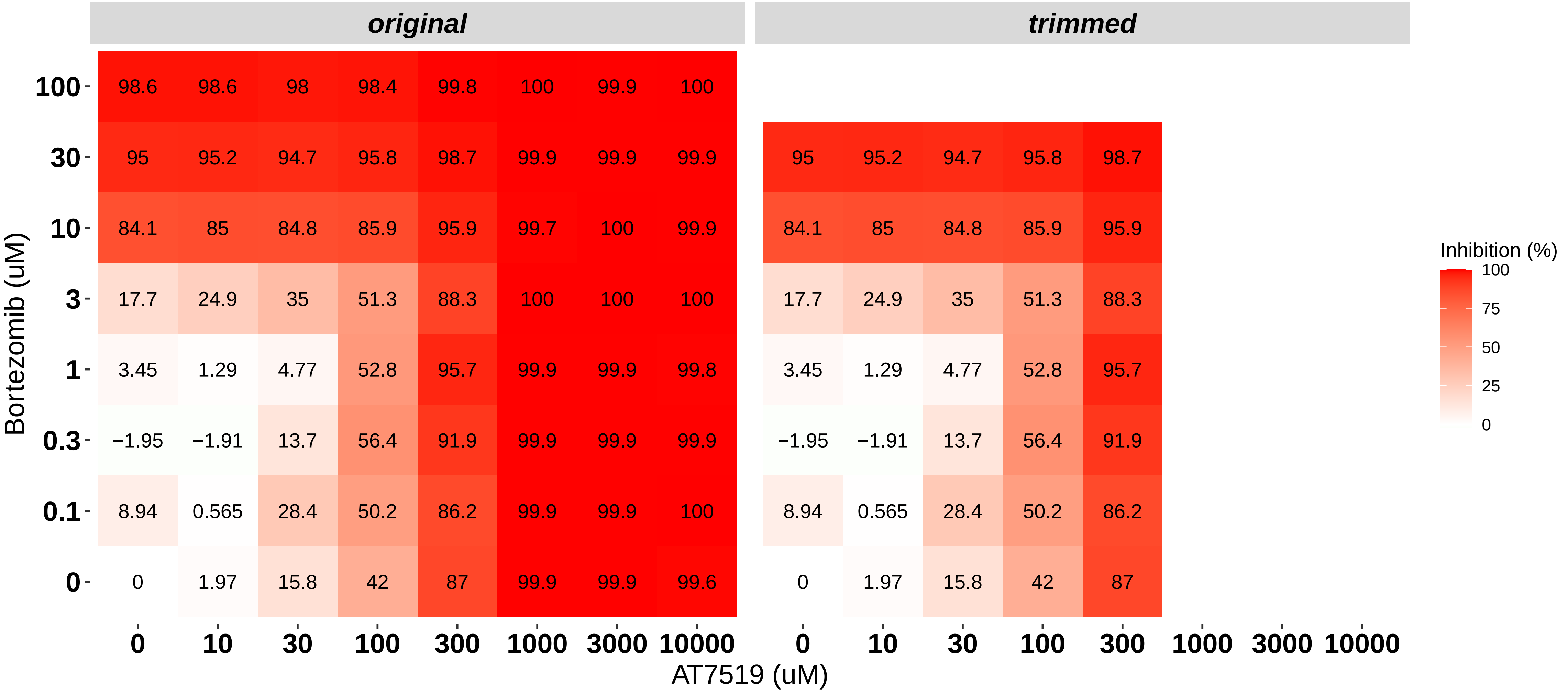

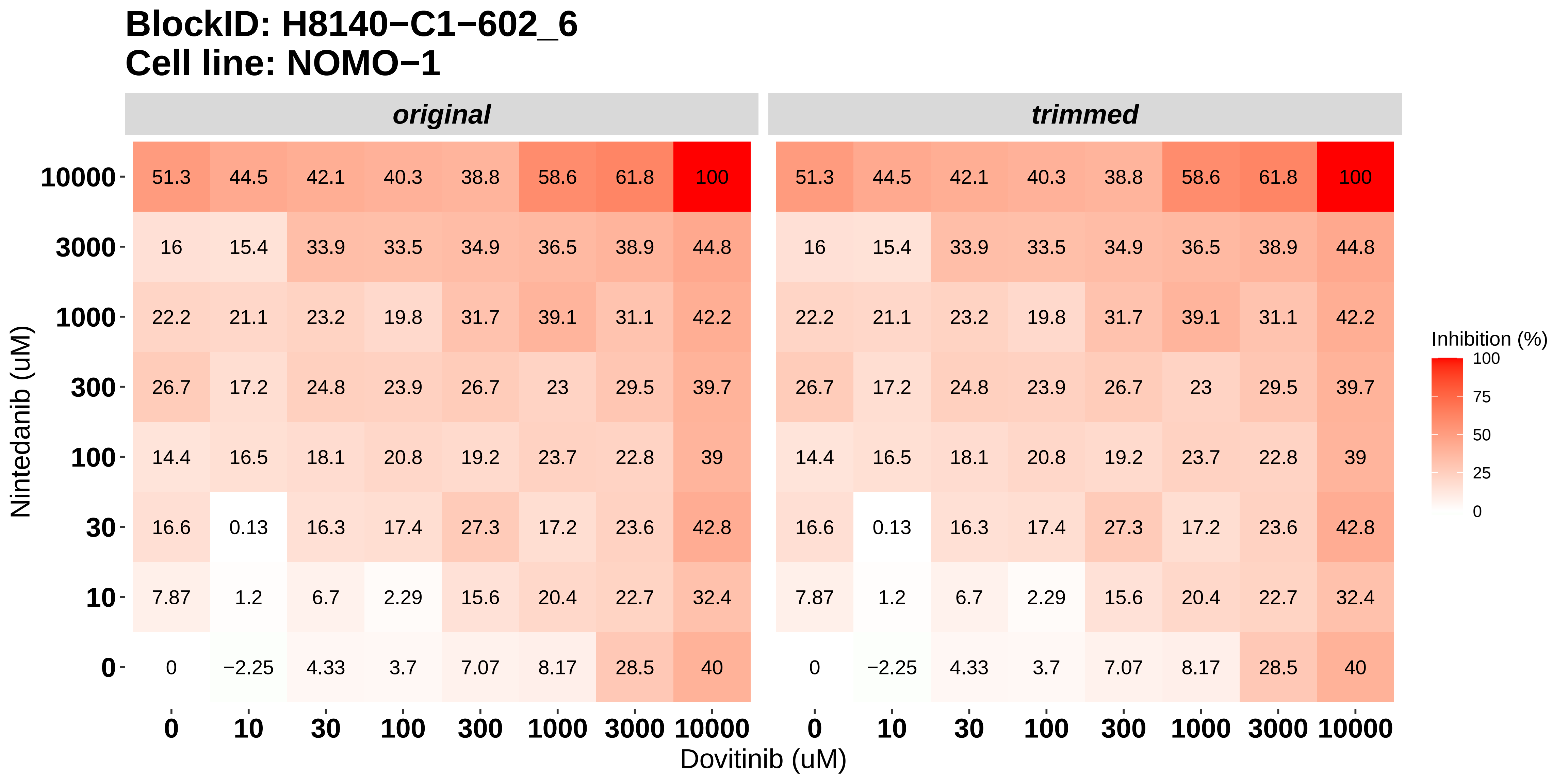
BlockID: H8140−C1−602_6
Cell line: NOMO−1
original
trimmed
Nintedanib (uM)
Dovitinib (uM)
Inhibition (%)
100
75
50
25
0
10000 51.3 44.5 42.1 40.3 38.8 58.6 61.8 100
3000 16 15.4 33.9 33.5 34.9 36.5 38.9 44.8
1000 22.2 21.1 23.2 19.8 31.7 39.1 31.1 42.2
300 26.7 17.2 24.8 23.9 26.7 23 29.5 39.7
100 14.4 16.5 18.1 20.8 19.2 23.7 22.8 39
30 16.6 0.13 16.3 17.4 27.3 17.2 23.6 42.8
10 7.87 1.2 6.7 2.29 15.6 20.4 22.7 32.4
0 0 −2.25 4.33 3.7 7.07 8.17 28.5 40
0 10 30 100 300 1000 3000 10000
51.3 44.5 42.1 40.3 38.8 58.6 61.8 100
16 15.4 33.9 33.5 34.9 36.5 38.9 44.8
22.2 21.1 23.2 19.8 31.7 39.1 31.1 42.2
26.7 17.2 24.8 23.9 26.7 23 29.5 39.7
14.4 16.5 18.1 20.8 19.2 23.7 22.8 39
16.6 0.13 16.3 17.4 27.3 17.2 23.6 42.8
7.87 1.2 6.7 2.29 15.6 20.4 22.7 32.4
0 −2.25 4.33 3.7 7.07 8.17 28.5 40
0 10 30 100 300 1000 3000 10000

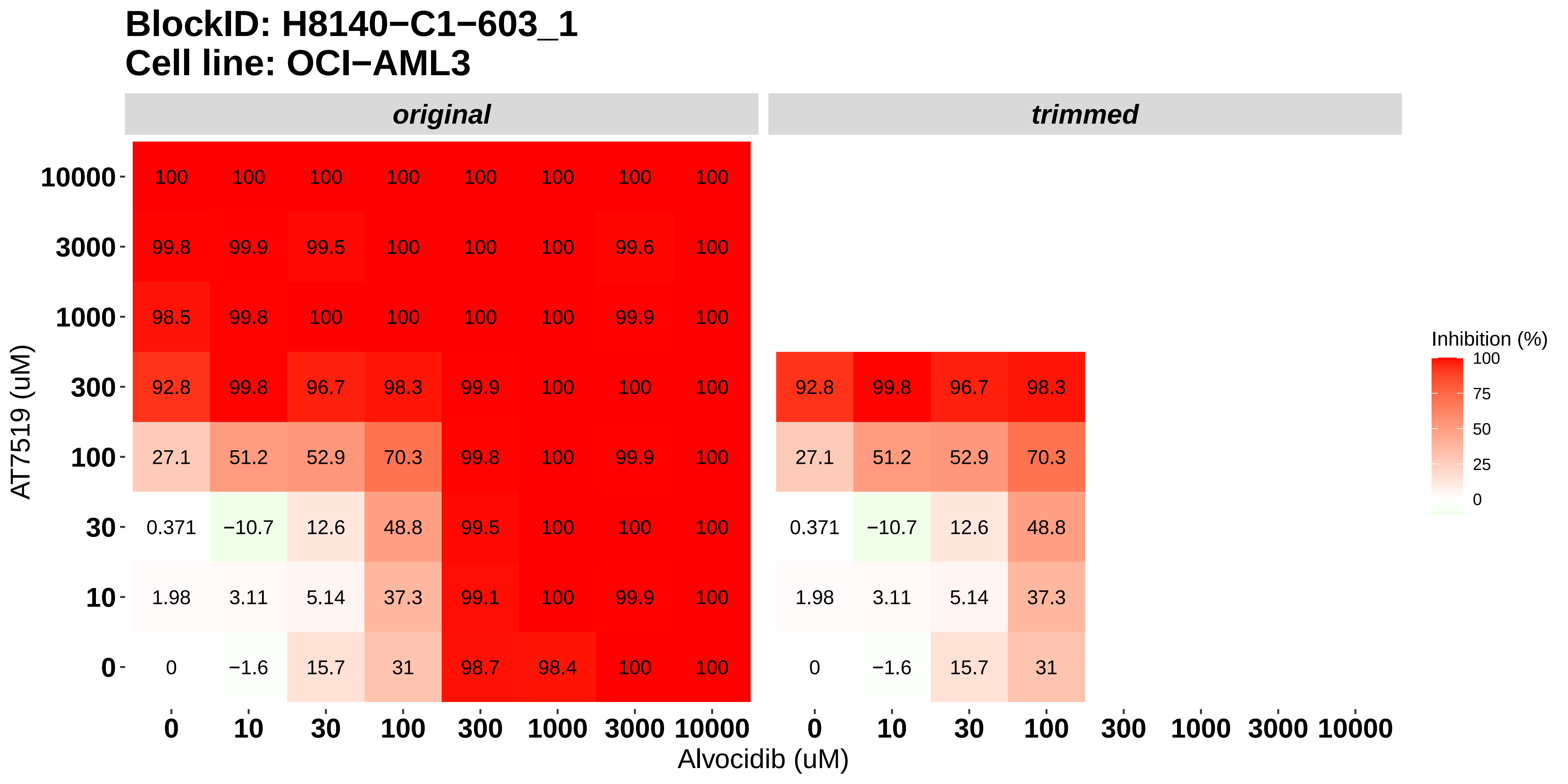

BlockID: H8140−C1−603_1
Cell line: OCI−AML3
original
trimmed
AT7519 (uM)
Alvocidib (uM)
Inhibition (%)
10000
3000
1000
300
100
30
10
0
100 100 100 100 100 100 100 100
99.8 99.9 99.5 100 100 100 99.6 100
98.5 99.8 100 100 100 100 99.9 100
92.8 99.8 96.7 98.3 99.9 100 100 100
27.1 51.2 52.9 70.3 99.8 100 99.9 100
0.371 −10.7 12.6 48.8 99.5 100 100 100
1.98 3.11 5.14 37.3 99.1 100 99.9 100
0 −1.6 15.7 31 98.7 98.4 100 100
92.8 99.8 96.7 98.3
27.1 51.2 52.9 70.3
0.371 −10.7 12.6 48.8
1.98 3.11 5.14 37.3
0 −1.6 15.7 31
0 10 30 100 300 1000 3000 10000
0 10 30 100 300 1000 3000 10000
100
75
50
25
0

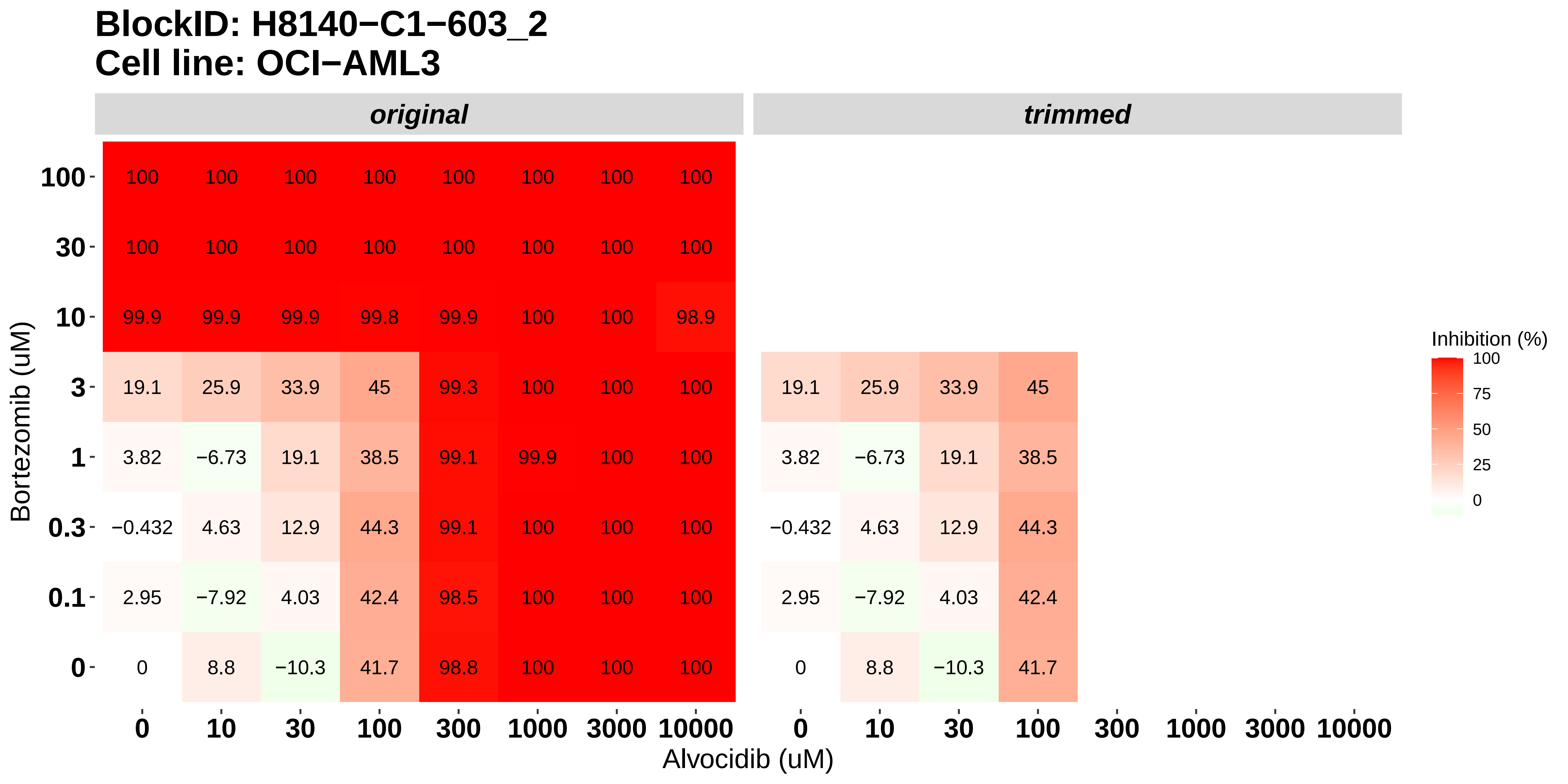

BlockID: H8140−C1−603_2
Cell line: OCI−AML3
original
trimmed
Bortezomib (uM)
Alvocidib (uM)
Inhibition (%)
100
75
50
25
0
100 100 100 100 100 100 100 100 100
30 100 100 100 100 100 100 100 100
10 99.9 99.9 99.9 99.8 99.9 100 100 98.9
3 19.1 25.9 33.9 45 99.3 100 100 100
1 3.82 −6.73 19.1 38.5 99.1 99.9 100 100
0.3 −0.432 4.63 12.9 44.3 99.1 100 100 100
0.1 2.95 −7.92 4.03 42.4 98.5 100 100 100
0 0 8.8 −10.3 41.7 98.8 100 100 100
0 10 30 100 300 1000 3000 10000
19.1 25.9 33.9 45
3.82 −6.73 19.1 38.5
−0.432 4.63 12.9 44.3
2.95 −7.92 4.03 42.4
0 8.8 −10.3 41.7
0 10 30 100 300 1000 3000 10000

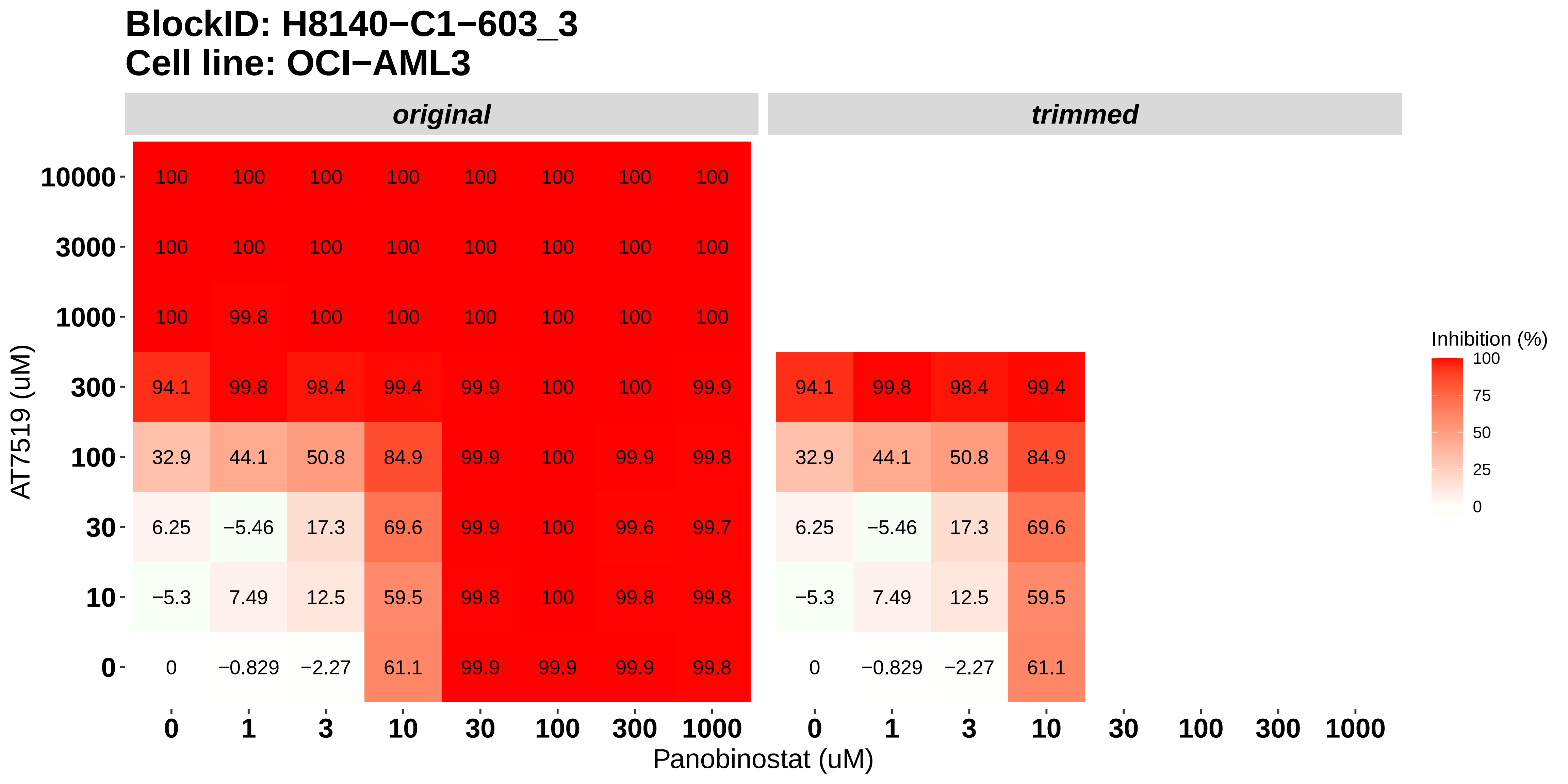
BlockID: H8140−C1−603_3
Cell line: OCI−AML3
original
trimmed
AT7519 (uM)
Panobinostat (uM)
Inhibition (%)
10000
3000
1000
300
100
30
10
0
0
1
3
10
30
100
300
1000
100 100 100 100 100 100 100 100
100 100 100 100 100 100 100 100
100 99.8 100 100 100 100 100 100
94.1 99.8 98.4 99.4 99.9 100 100 99.9
32.9 44.1 50.8 84.9 99.9 100 99.9 99.8
6.25 −5.46 17.3 69.6 99.9 100 99.6 99.7
−5.3 7.49 12.5 59.5 99.8 100 99.8 99.8
0 −0.829 −2.27 61.1 99.9 99.9 99.9 99.8
94.1 99.8 98.4 99.4
32.9 44.1 50.8 84.9
6.25 −5.46 17.3 69.6
−5.3 7.49 12.5 59.5
0 −0.829 −2.27 61.1
100
75
50
25
0

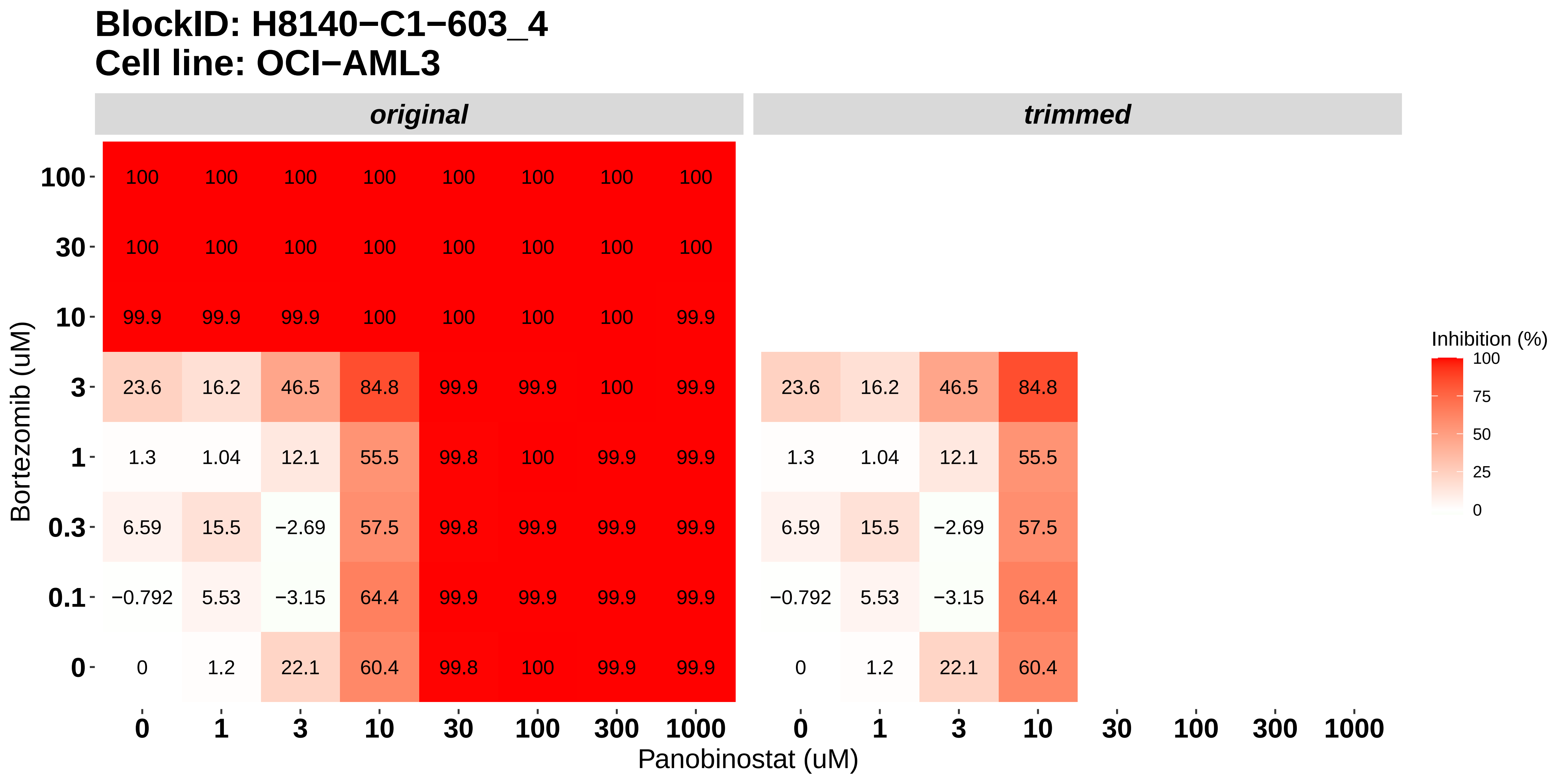
BlockID: H8140−C1−603_4
Cell line: OCI−AML3
original
trimmed
100 100 100 100 100 100 100 100
100 100 100 100 100 100 100 100
99.9 99.9 99.9 100 100 100 100 99.9
23.6 16.2 46.5 84.8 99.9 99.9 100 99.9
1.3 1.04 12.1 55.5 99.8 100 99.9 99.9
6.59 15.5 −2.69 57.5 99.8 99.9 99.9 99.9
−0.792 5.53 −3.15 64.4 99.9 99.9 99.9 99.9
0 1.2 22.1 60.4 99.8 100 99.9 99.9
23.6 16.2 46.5 84.8
1.3 1.04 12.1 55.5
6.59 15.5 −2.69 57.5
−0.792 5.53 −3.15 64.4
0 1.2 22.1 60.4
Bortezomib (uM)
100
30
10
3
1
0.3
0.1
0
Panobinostat (uM)
0 1 3 10 30 100 300 1000
0 1 3 10 30 100 300 1000
Inhibition (%)
100
75
50
25
0

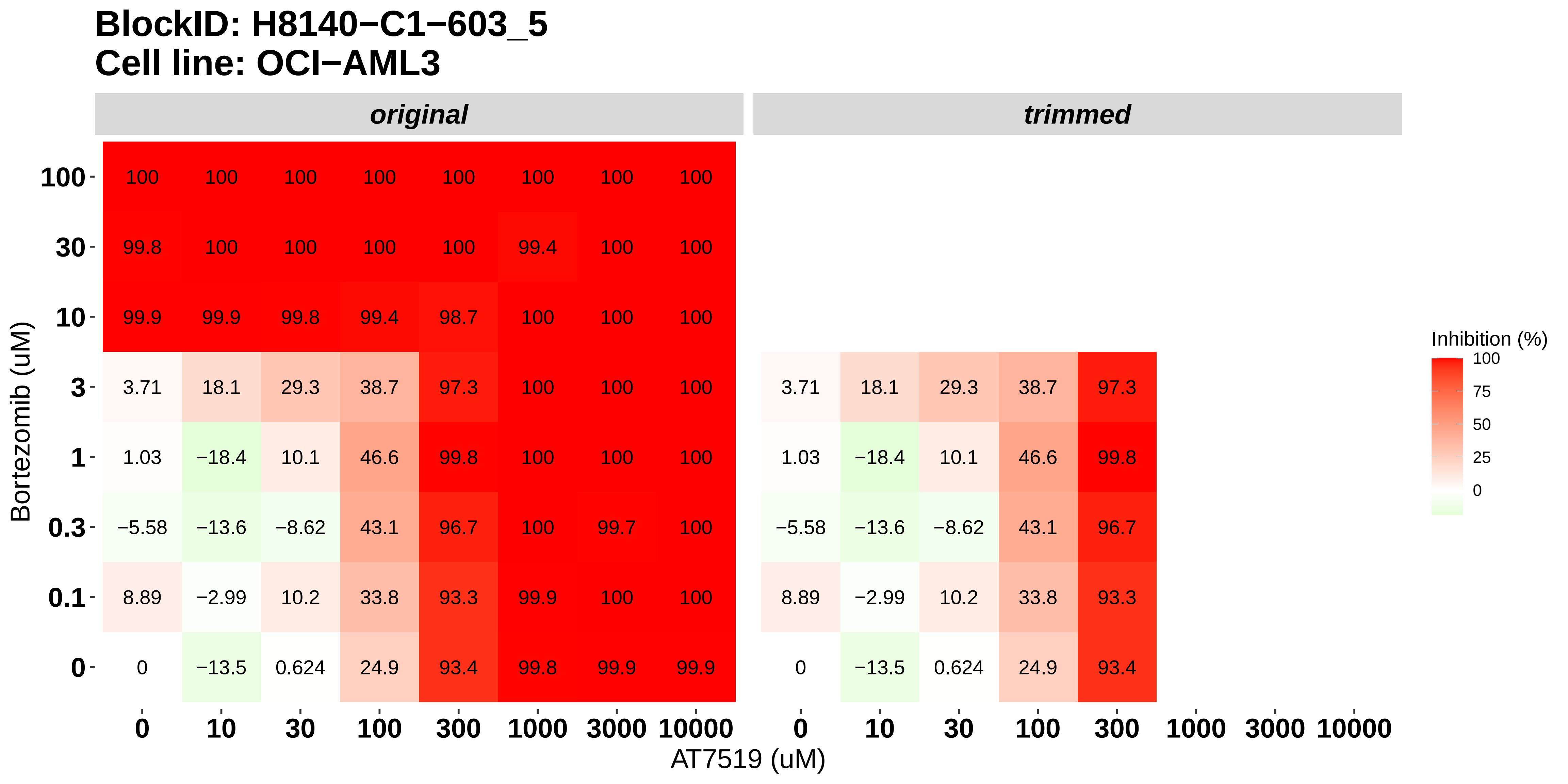
BlockID: H8140−C1−603_5
Cell line: OCI−AML3
original
trimmed
Bortezomib (uM)
100
30
10
3
1
0.3
0.1
0
100 100 100 100 100 100 100 100
99.8 100 100 100 100 99.4 100 100
99.9 99.9 99.8 99.4 98.7 100 100 100
3.71 18.1 29.3 38.7 97.3 100 100 100
1.03 −18.4 10.1 46.6 99.8 100 100 100
−5.58 −13.6 −8.62 43.1 96.7 100 99.7 100
8.89 −2.99 10.2 33.8 93.3 99.9 100 100
0 −13.5 0.624 24.9 93.4 99.8 99.9 99.9
3.71 18.1 29.3 38.7 97.3
1.03 −18.4 10.1 46.6 99.8
−5.58 −13.6 −8.62 43.1 96.7
8.89 −2.99 10.2 33.8 93.3
0 −13.5 0.624 24.9 93.4
0 10 30 100 300 1000 3000 10000
0 10 30 100 300 1000 3000 10000
AT7519 (uM)
Inhibition (%)
100
75
50
25
0

BlockID: H8140−C1−603_6
Cell line: OCI−AML3

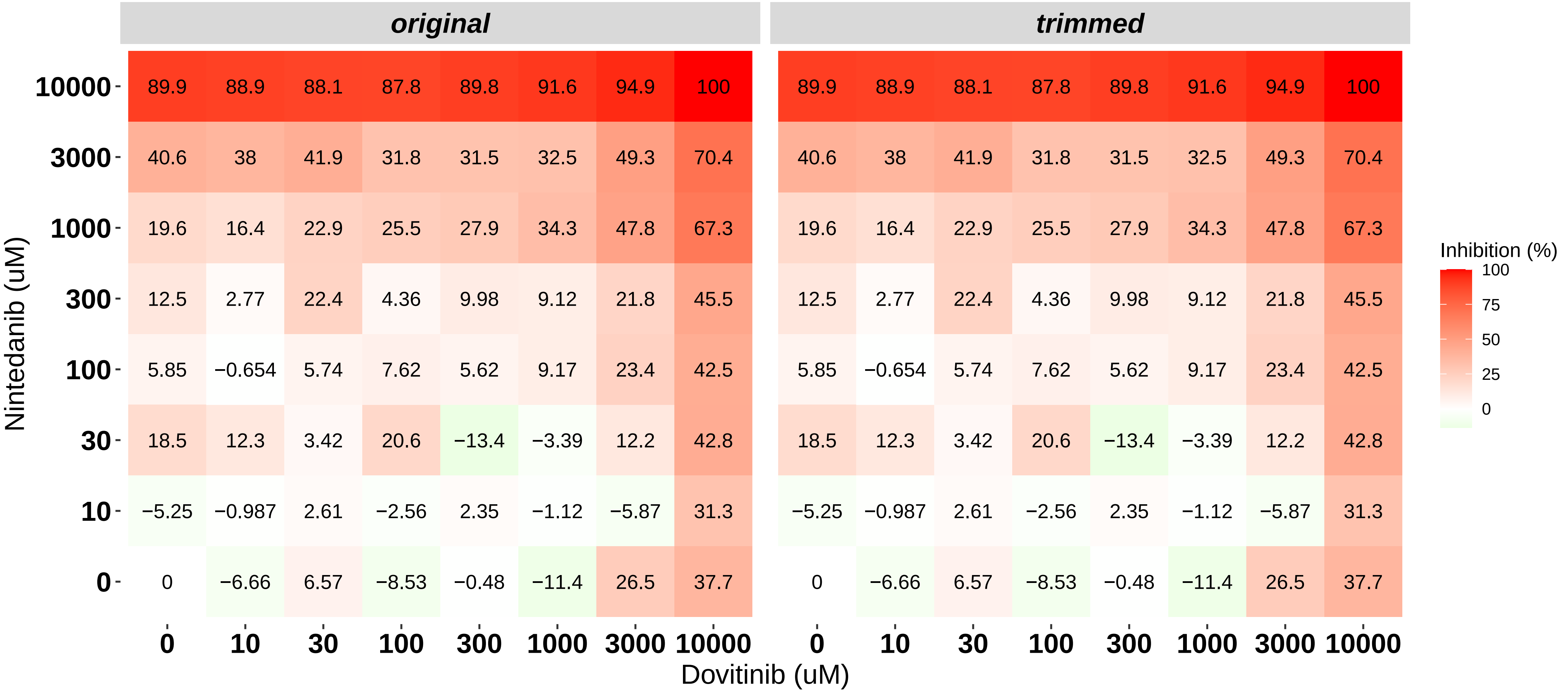

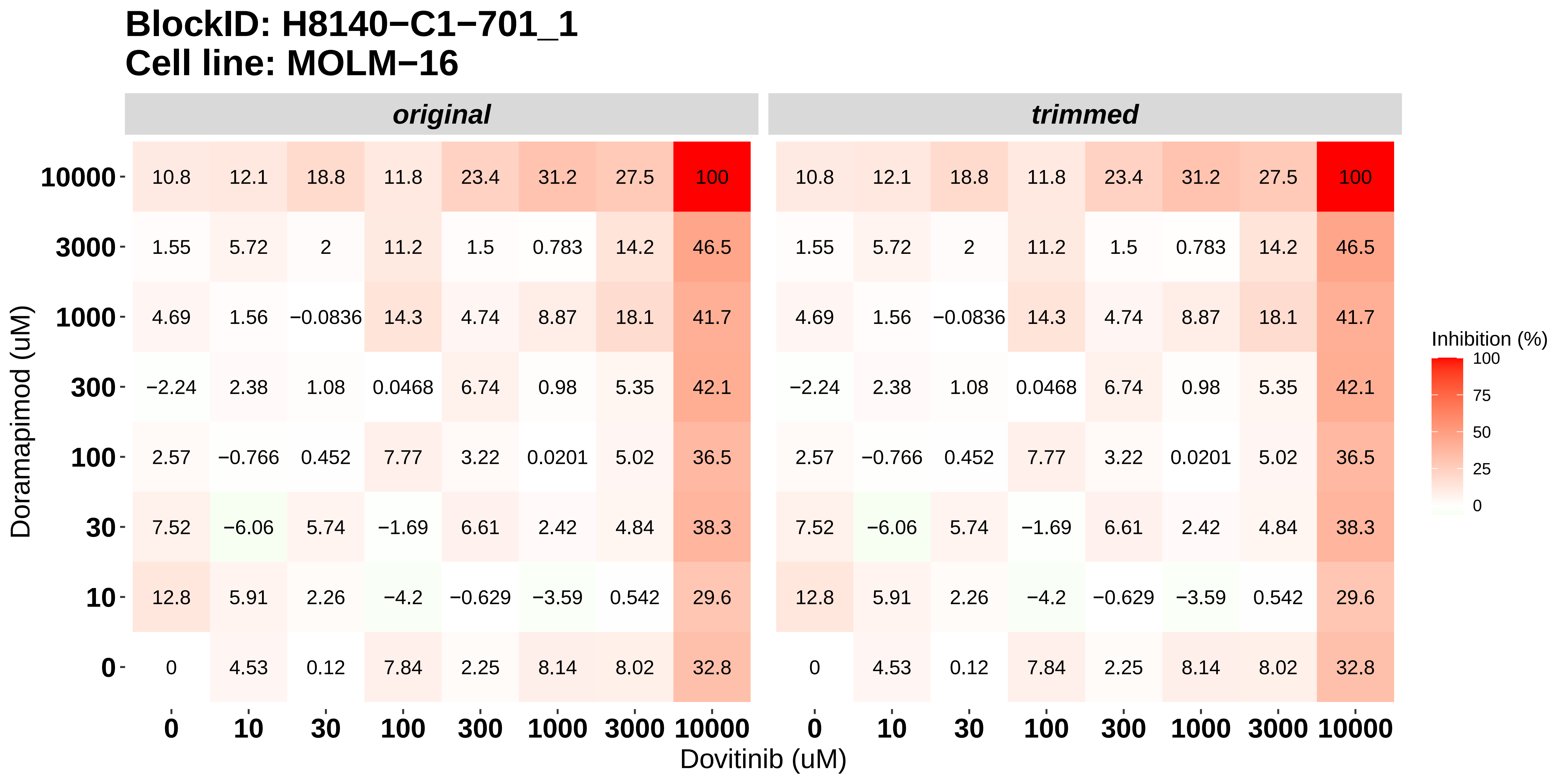
BlockID: H8140−C1−701_1
Cell line: MOLM−16
original
trimmed
Doramapimod (uM)
Dovitinib (uM)
Inhibition (%)
10000
3000
1000
300
100
30
10
0
10.8 12.1 18.8 11.8 23.4 31.2 27.5 100
1.55 5.72 2 11.2 1.5 0.783 14.2 46.5
4.69 1.56 −0.0836 14.3 4.74 8.87 18.1 41.7
−2.24 2.38 1.08 0.0468 6.74 0.98 5.35 42.1
2.57 −0.766 0.452 7.77 3.22 0.0201 5.02 36.5
7.52 −6.06 5.74 −1.69 6.61 2.42 4.84 38.3
12.8 5.91 2.26 −4.2 −0.629 −3.59 0.542 29.6
0 4.53 0.12 7.84 2.25 8.14 8.02 32.8
100
75
50
25
0

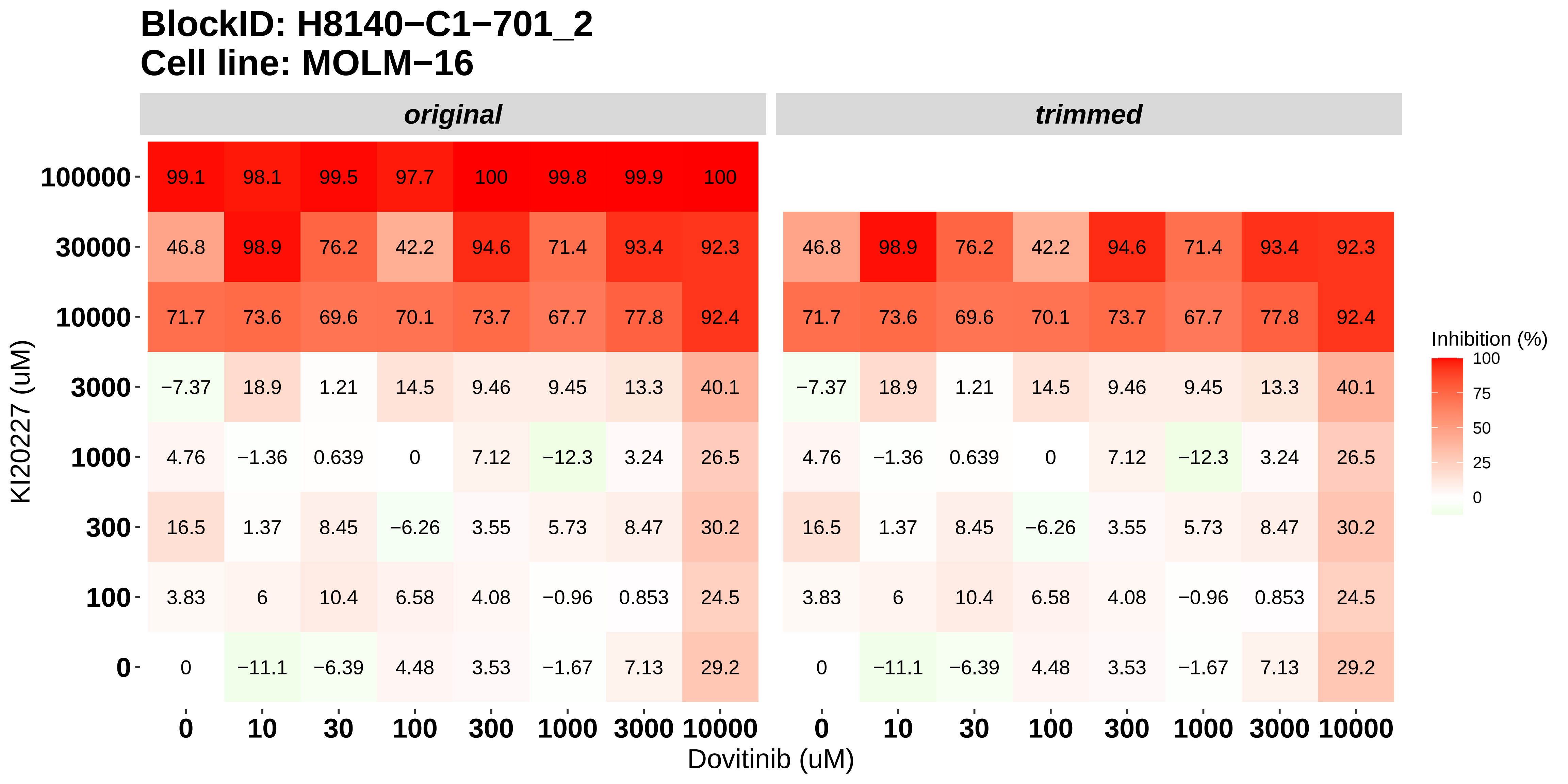

BlockID: H8140−C1−701_2
Cell line: MOLM−16
original
trimmed
KI20227 (uM)
Dovitinib (uM)
Inhibition (%)
100000
30000
10000
3000
1000
300
100
0
0
10
30
100
300
1000
3000
10000
99.1 98.1 99.5 97.7 100 99.8 99.9 100
46.8 98.9 76.2 42.2 94.6 71.4 93.4 92.3
71.7 73.6 69.6 70.1 73.7 67.7 77.8 92.4
−7.37 18.9 1.21 14.5 9.46 9.45 13.3 40.1
4.76 −1.36 0.639 0 7.12 −12.3 3.24 26.5
16.5 1.37 8.45 −6.26 3.55 5.73 8.47 30.2
3.83 6 10.4 6.58 4.08 −0.96 0.853 24.5
0 −11.1 −6.39 4.48 3.53 −1.67 7.13 29.2
100
75
50
25
0

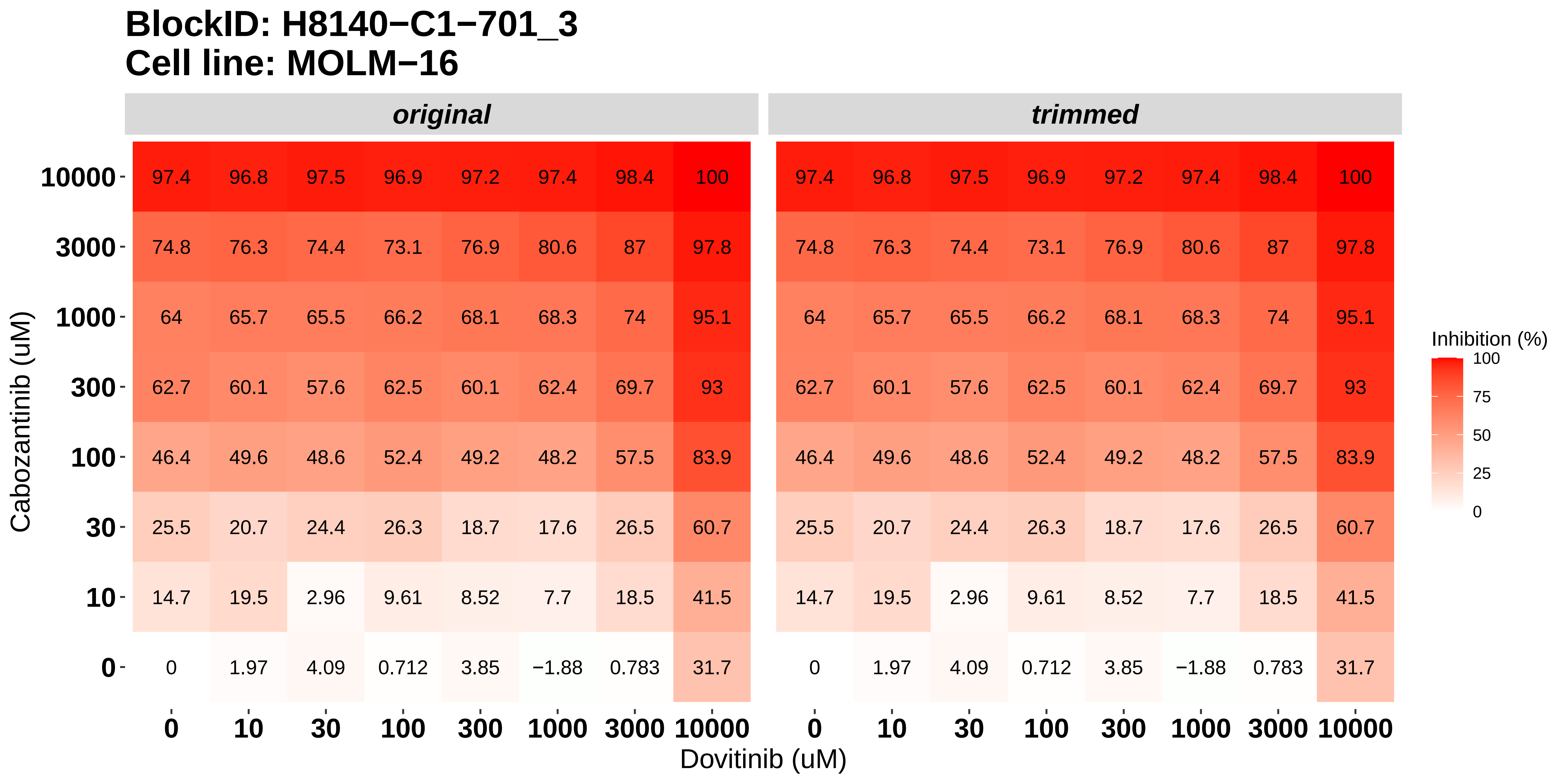
BlockID: H8140−C1−701_3
Cell line: MOLM−16
original
trimmed
Cabozantinib (uM)
Dovitinib (uM)
Inhibition (%)
100
75
50
25
0
10000 97.4 96.8 97.5 96.9 97.2 97.4 98.4 100
3000 74.8 76.3 74.4 73.1 76.9 80.6 87 97.8
1000 64 65.7 65.5 66.2 68.1 68.3 74 95.1
300 62.7 60.1 57.6 62.5 60.1 62.4 69.7 93
100 46.4 49.6 48.6 52.4 49.2 48.2 57.5 83.9
30 25.5 20.7 24.4 26.3 18.7 17.6 26.5 60.7
10 14.7 19.5 2.96 9.61 8.52 7.7 18.5 41.5
0 0 1.97 4.09 0.712 3.85 −1.88 0.783 31.7
0 10 30 100 300 1000 3000 10000

BlockID: H8140−C1−701_4
Cell line: MOLM−16

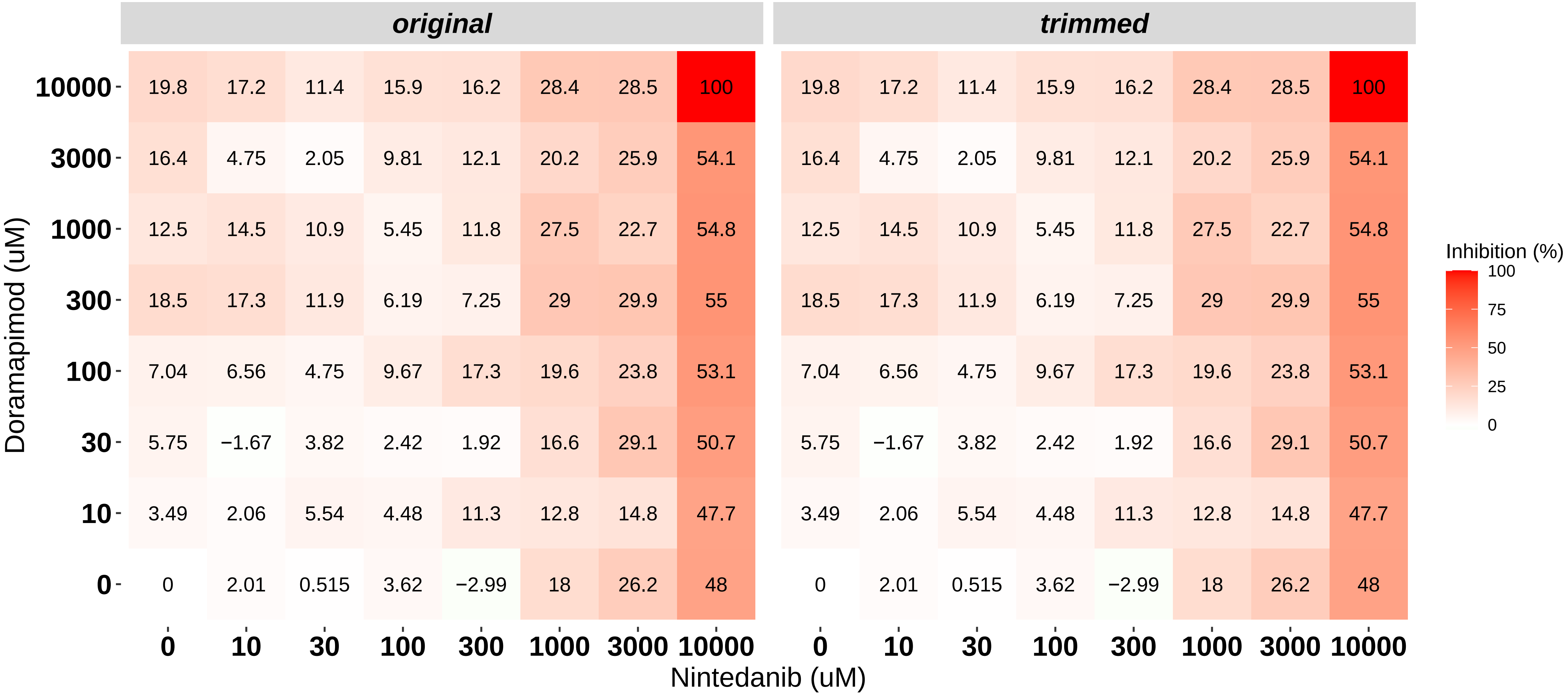

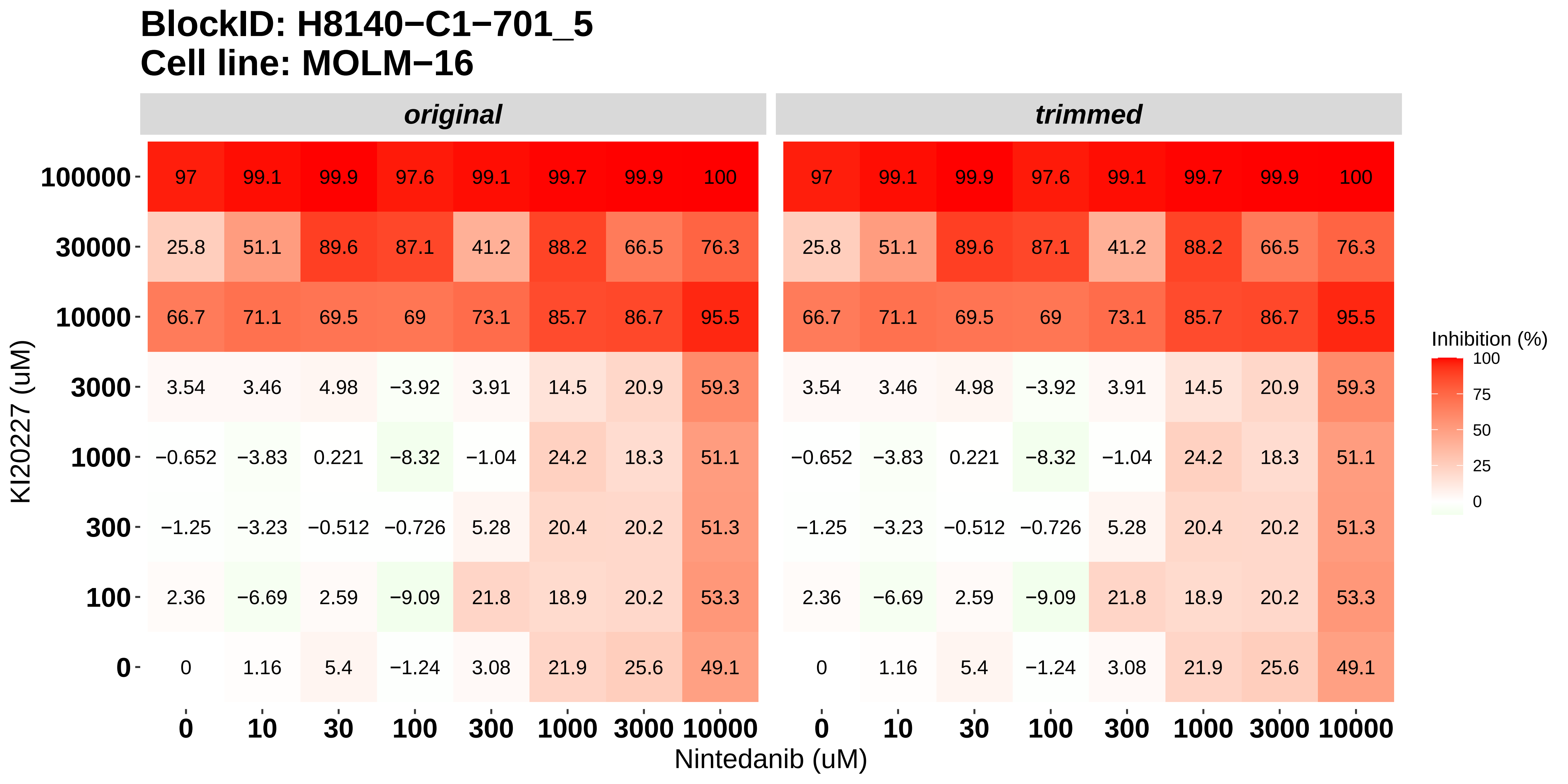
BlockID: H8140−C1−701_5
Cell line: MOLM−16
original
trimmed
KI20227 (uM)
Nintedanib (uM)
Inhibition (%)
100000
30000
10000
3000
1000
300
100
0
0
10
30
100
300
1000
3000
10000
97 99.1 99.9 97.6 99.1 99.7 99.9 100
25.8 51.1 89.6 87.1 41.2 88.2 66.5 76.3
66.7 71.1 69.5 69 73.1 85.7 86.7 95.5
3.54 3.46 4.98 −3.92 3.91 14.5 20.9 59.3
−0.652 −3.83 0.221 −8.32 −1.04 24.2 18.3 51.1
−1.25 −3.23 −0.512 −0.726 5.28 20.4 20.2 51.3
2.36 −6.69 2.59 −9.09 21.8 18.9 20.2 53.3
0 1.16 5.4 −1.24 3.08 21.9 25.6 49.1
100
75
50
25
0

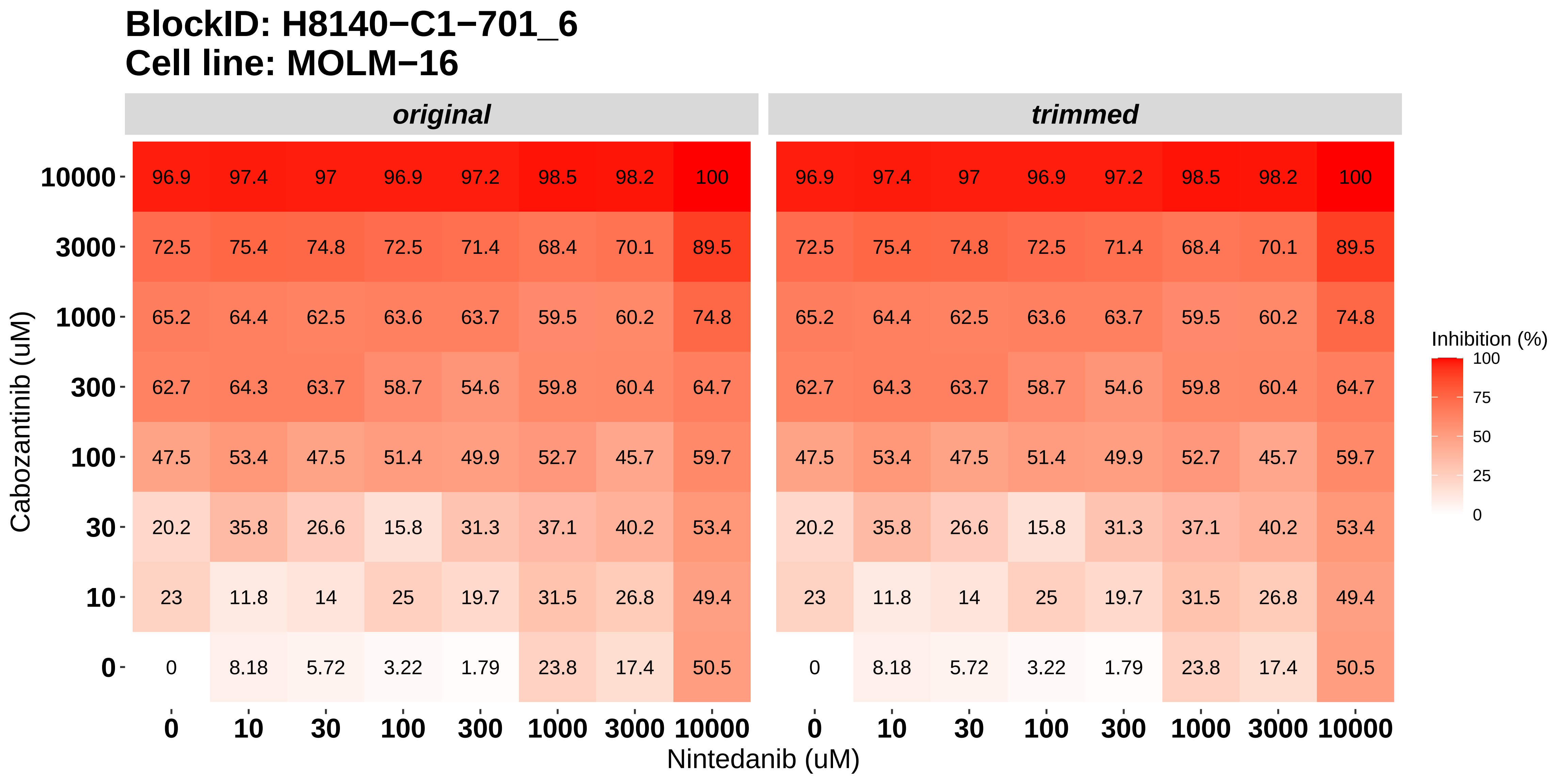
BlockID: H8140−C1−701_6
Cell line: MOLM−16
original
trimmed
Cabozantinib (uM)
10000
3000
1000
300
100
30
10
0
96.9 97.4 97 96.9 97.2 98.5 98.2 100
72.5 75.4 74.8 72.5 71.4 68.4 70.1 89.5
65.2 64.4 62.5 63.6 63.7 59.5 60.2 74.8
62.7 64.3 63.7 58.7 54.6 59.8 60.4 64.7
47.5 53.4 47.5 51.4 49.9 52.7 45.7 59.7
20.2 35.8 26.6 15.8 31.3 37.1 40.2 53.4
23 11.8 14 25 19.7 31.5 26.8 49.4
0 8.18 5.72 3.22 1.79 23.8 17.4 50.5
0 10 30 100 300 1000 3000 10000
Nintedanib (uM)
Inhibition (%)
100
75
50
25
0

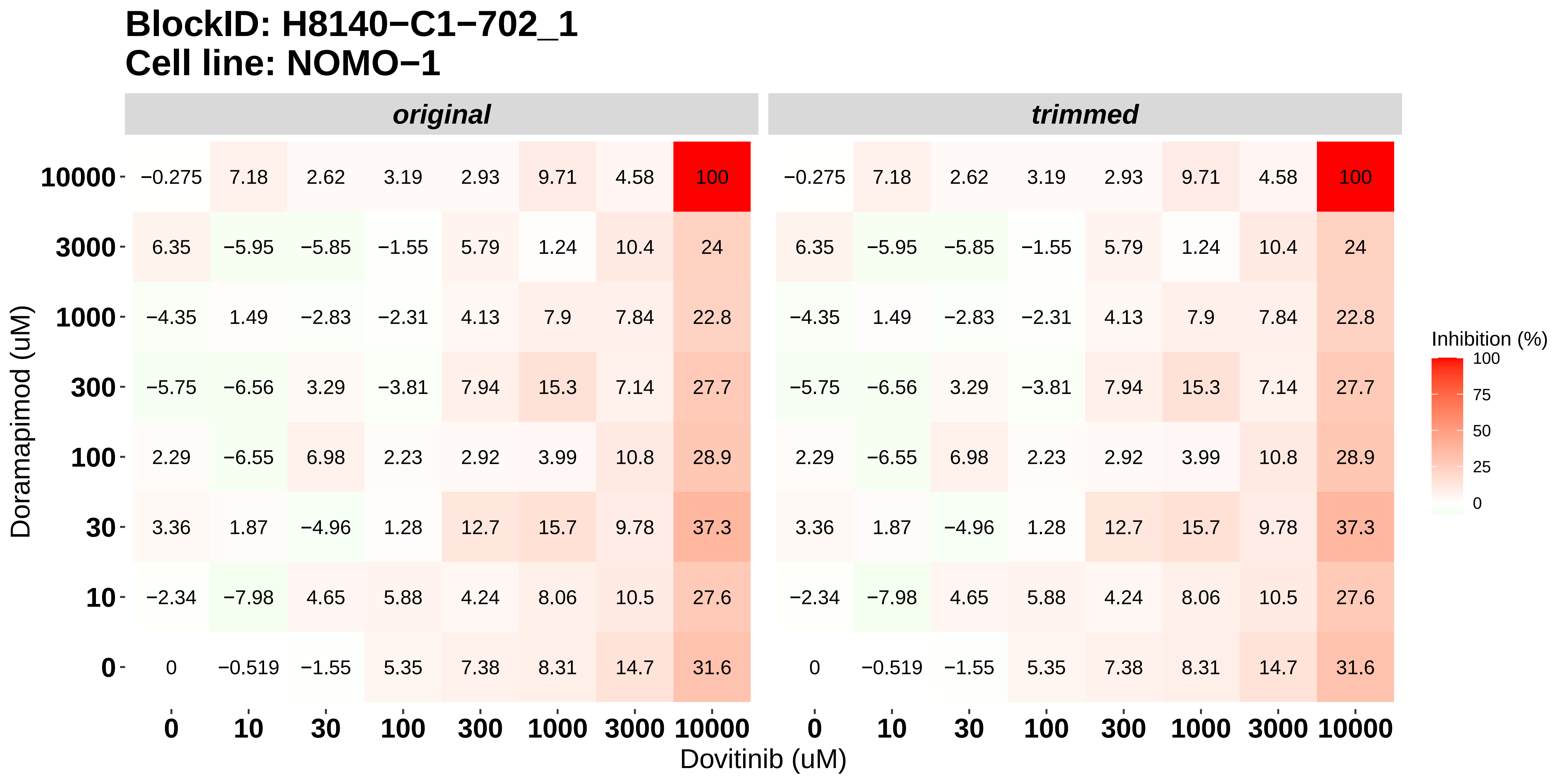
BlockID: H8140−C1−702_1
Cell line: NOMO−1
original
trimmed
Doramapimod (uM)
Dovitinib (uM)
Inhibition (%)
100
75
50
25
0
10000
3000
1000
300
100
30
10
0
−0.275 7.18 2.62 3.19 2.93 9.71 4.58 100
6.35 −5.95 −5.85 −1.55 5.79 1.24 10.4 24
−4.35 1.49 −2.83 −2.31 4.13 7.9 7.84 22.8
−5.75 −6.56 3.29 −3.81 7.94 15.3 7.14 27.7
2.29 −6.55 6.98 2.23 2.92 3.99 10.8 28.9
3.36 1.87 −4.96 1.28 12.7 15.7 9.78 37.3
−2.34 −7.98 4.65 5.88 4.24 8.06 10.5 27.6
0 −0.519 −1.55 5.35 7.38 8.31 14.7 31.6
0 10 30 100 300 1000 3000 10000

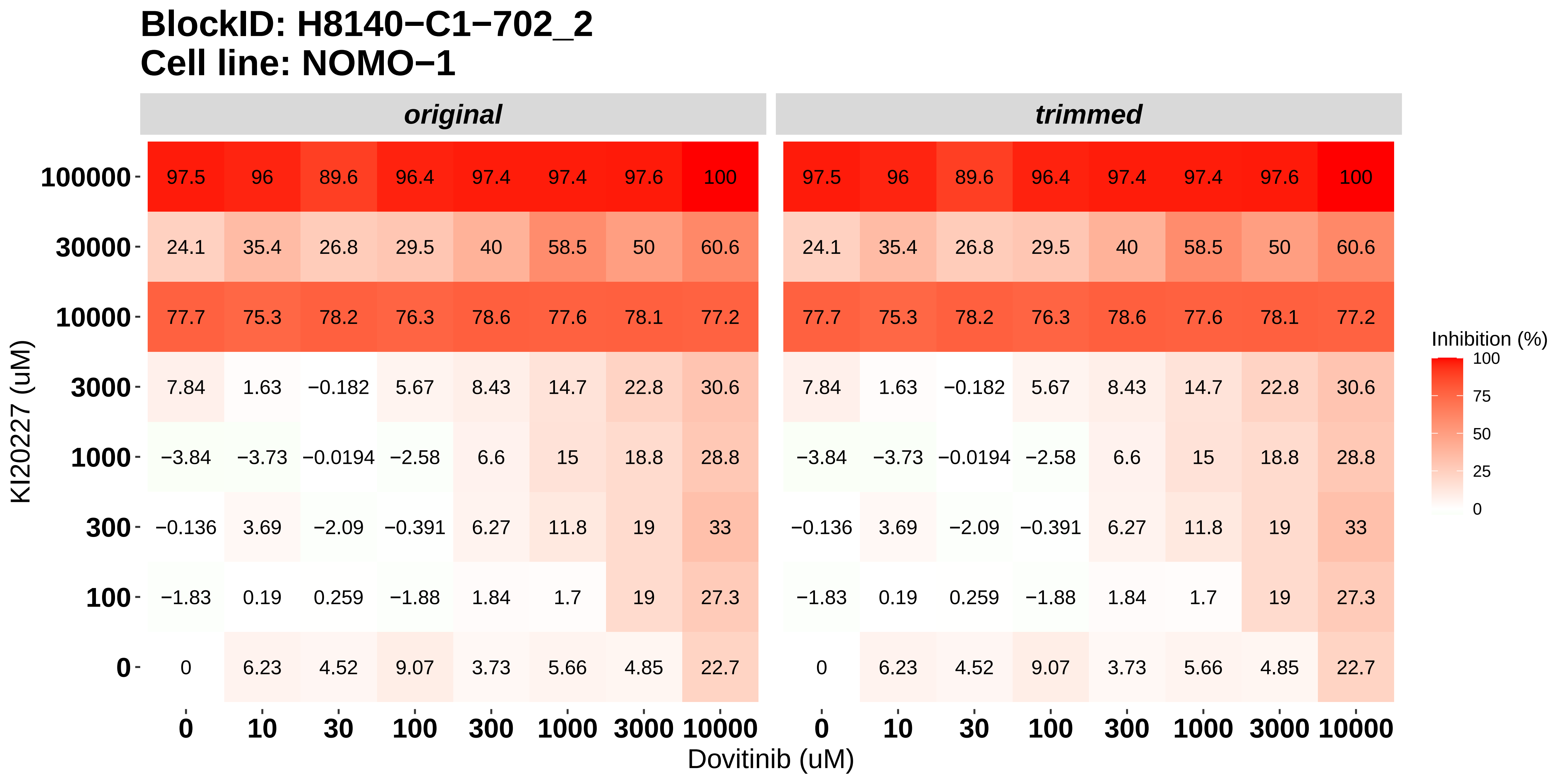
BlockID: H8140−C1−702_2
Cell line: NOMO−1
original
trimmed
100000
30000
10000
3000
1000
300
100
0
KI20227 (uM)
97.5 96 89.6 96.4 97.4 97.4 97.6 100
24.1 35.4 26.8 29.5 40 58.5 50 60.6
77.7 75.3 78.2 76.3 78.6 77.6 78.1 77.2
7.84 1.63 −0.182 5.67 8.43 14.7 22.8 30.6
−3.84 −3.73 −0.0194 −2.58 6.6 15 18.8 28.8
−0.136 3.69 −2.09 −0.391 6.27 11.8 19 33
−1.83 0.19 0.259 −1.88 1.84 1.7 19 27.3
0 6.23 4.52 9.07 3.73 5.66 4.85 22.7
0 10 30 100 300 1000 3000 10000
Dovitinib (uM)
Inhibition (%)
100
75
50
25
0

BlockID: H8140−C1−702_3
Cell line: NOMO−1

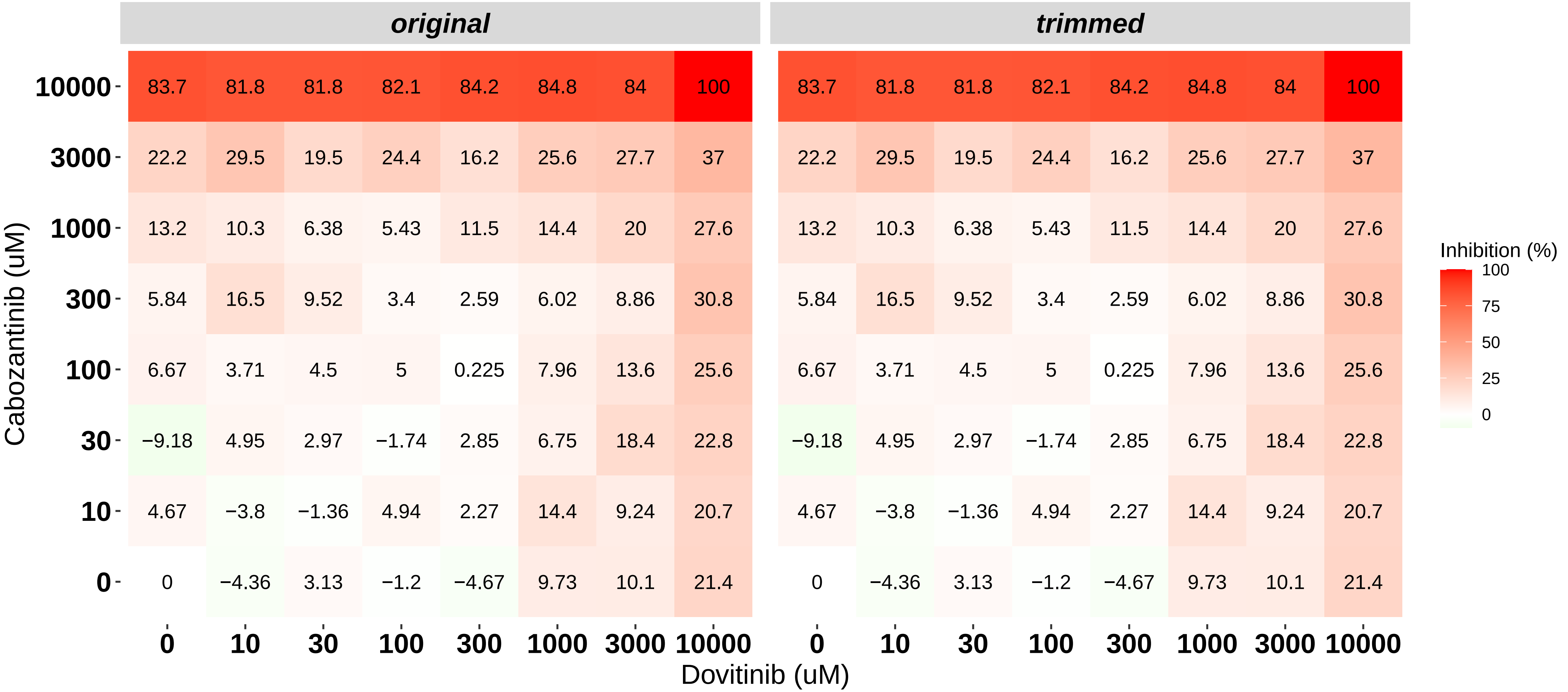

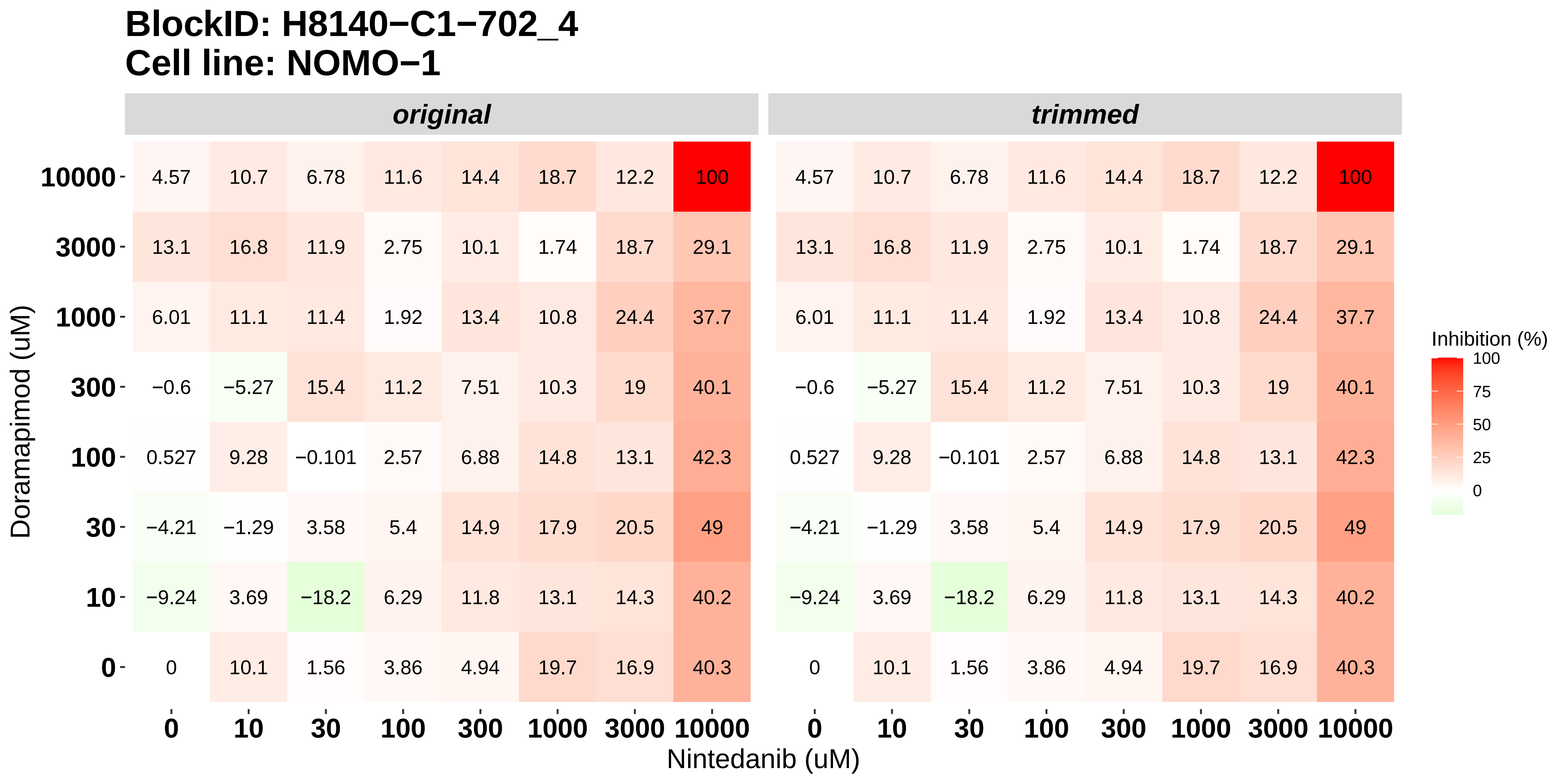

BlockID: H8140−C1−702_4
Cell line: NOMO−1
original
trimmed
Doramapimod (uM)
Nintedanib (uM)
Inhibition (%)
0
10
30
100
300
1000
3000
10000
4.57 10.7 6.78 11.6 14.4 18.7 12.2 100
13.1 16.8 11.9 2.75 10.1 1.74 18.7 29.1
6.01 11.1 11.4 1.92 13.4 10.8 24.4 37.7
−0.6 −5.27 15.4 11.2 7.51 10.3 19 40.1
0.527 9.28 −0.101 2.57 6.88 14.8 13.1 42.3
−4.21 −1.29 3.58 5.4 14.9 17.9 20.5 49
−9.24 3.69 −18.2 6.29 11.8 13.1 14.3 40.2
0 10.1 1.56 3.86 4.94 19.7 16.9 40.3
100
75
50
25
0

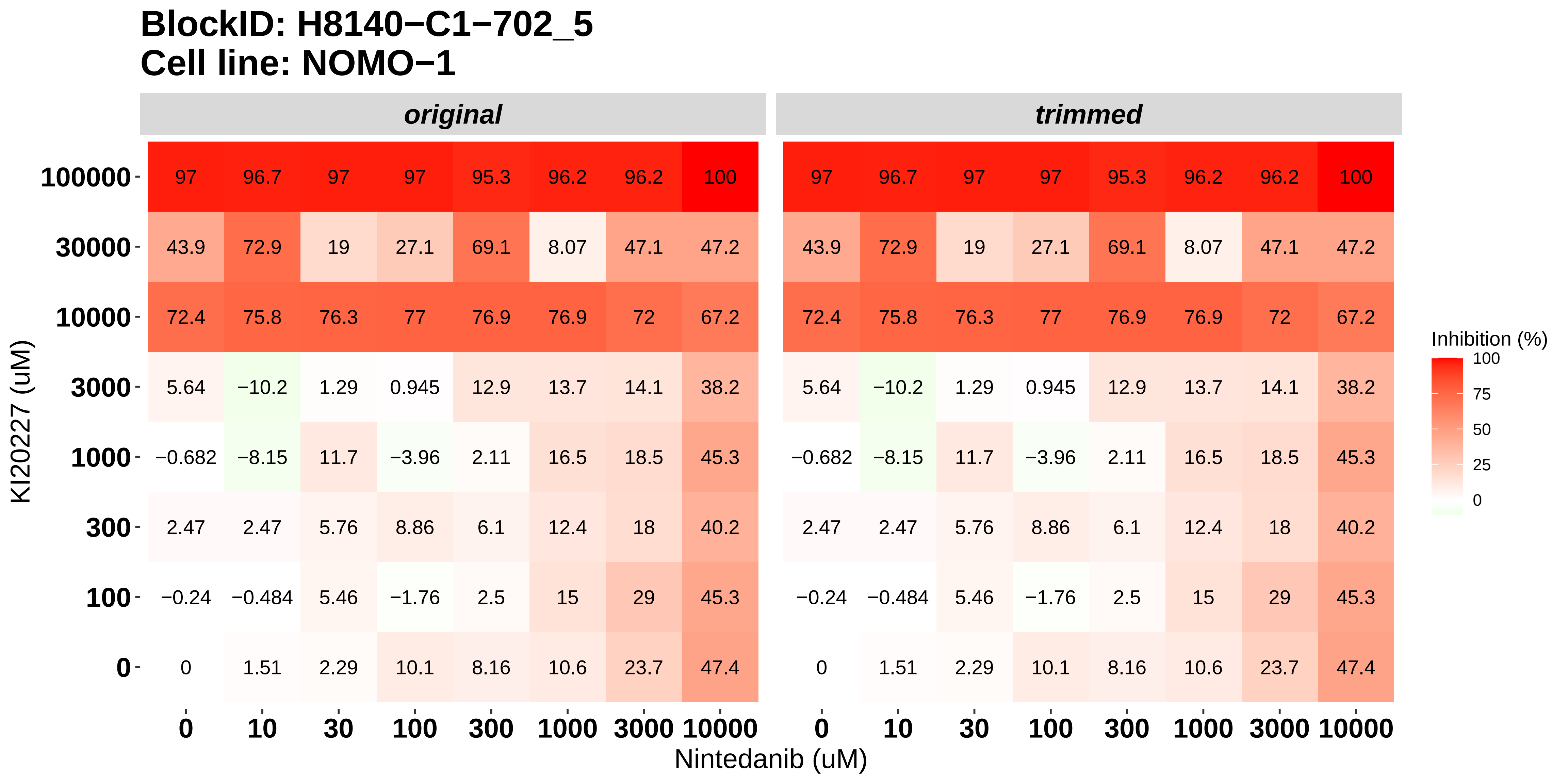
BlockID: H8140−C1−702_5
Cell line: NOMO−1
original
trimmed
KI20227 (uM)
Nintedanib (uM)
Inhibition (%)
100000
30000
10000
3000
1000
300
100
0
0
10
30
100
300
1000
3000
10000
97 96.7 97 97 95.3 96.2 96.2 100
43.9 72.9 19 27.1 69.1 8.07 47.1 47.2
72.4 75.8 76.3 77 76.9 76.9 72 67.2
5.64 −10.2 1.29 0.945 12.9 13.7 14.1 38.2
−0.682 −8.15 11.7 −3.96 2.11 16.5 18.5 45.3
2.47 2.47 5.76 8.86 6.1 12.4 18 40.2
−0.24 −0.484 5.46 −1.76 2.5 15 29 45.3
0 1.51 2.29 10.1 8.16 10.6 23.7 47.4
100
75
50
25
0

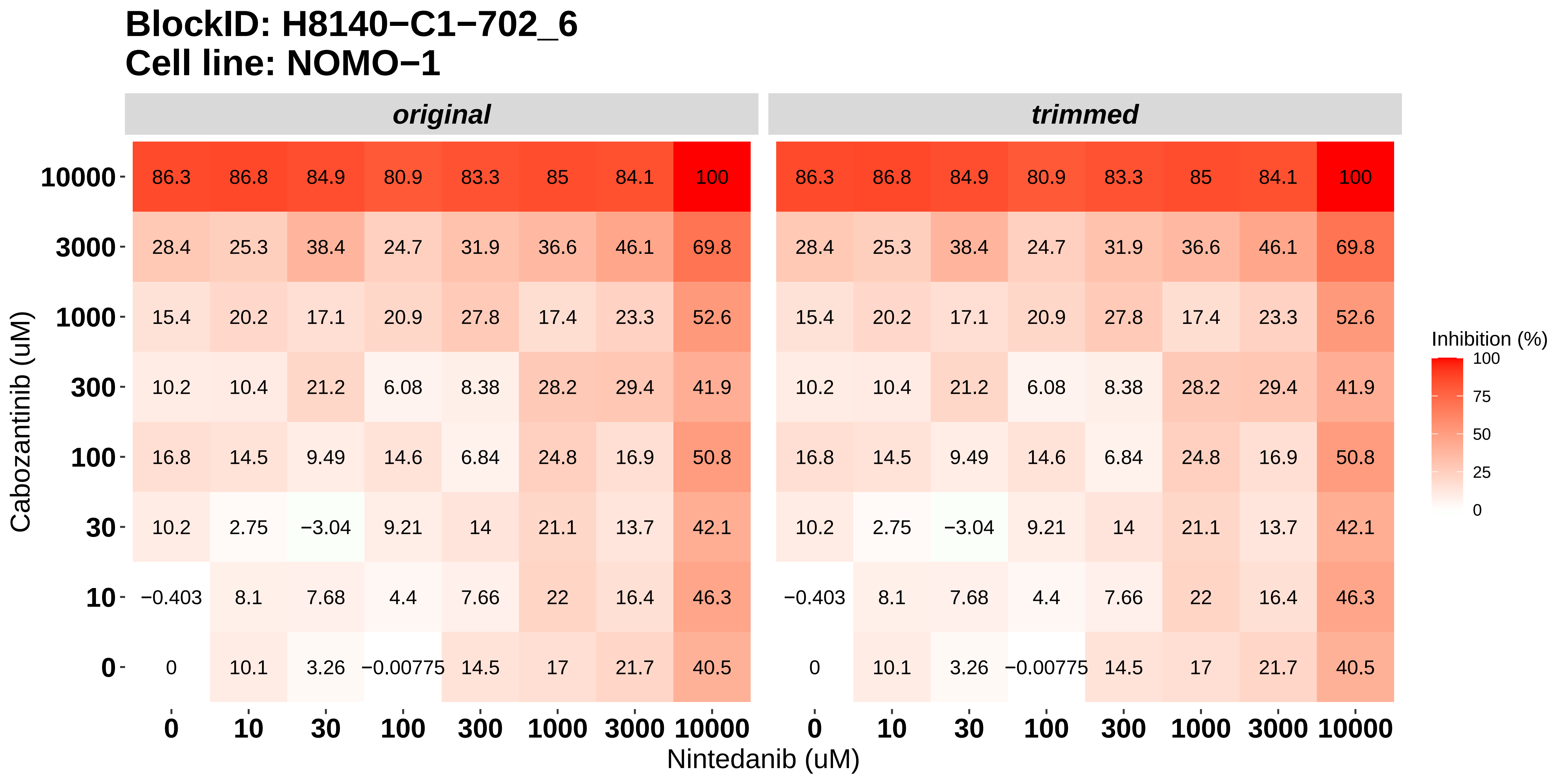
BlockID: H8140−C1−702_6
Cell line: NOMO−1
original
trimmed
Cabozantinib (uM)
Nintedanib (uM)
Inhibition (%)
100
75
50
25
0
10000 86.3 86.8 84.9 80.9 83.3 85 84.1 100
3000 28.4 25.3 38.4 24.7 31.9 36.6 46.1 69.8
1000 15.4 20.2 17.1 20.9 27.8 17.4 23.3 52.6
300 10.2 10.4 21.2 6.08 8.38 28.2 29.4 41.9
100 16.8 14.5 9.49 14.6 6.84 24.8 16.9 50.8
30 10.2 2.75 −3.04 9.21 14 21.1 13.7 42.1
10 −0.403 8.1 7.68 4.4 7.66 22 16.4 46.3
0 0 10.1 3.26 −0.00775 14.5 17 21.7 40.5
0 10 30 100 300 1000 3000 10000
86.3 86.8 84.9 80.9 83.3 85 84.1 100
28.4 25.3 38.4 24.7 31.9 36.6 46.1 69.8
15.4 20.2 17.1 20.9 27.8 17.4 23.3 52.6
10.2 10.4 21.2 6.08 8.38 28.2 29.4 41.9
16.8 14.5 9.49 14.6 6.84 24.8 16.9 50.8
10.2 2.75 −3.04 9.21 14 21.1 13.7 42.1
−0.403 8.1 7.68 4.4 7.66 22 16.4 46.3
0 10.1 3.26 −0.00775 14.5 17 21.7 40.5
0 10 30 100 300 1000 3000 10000

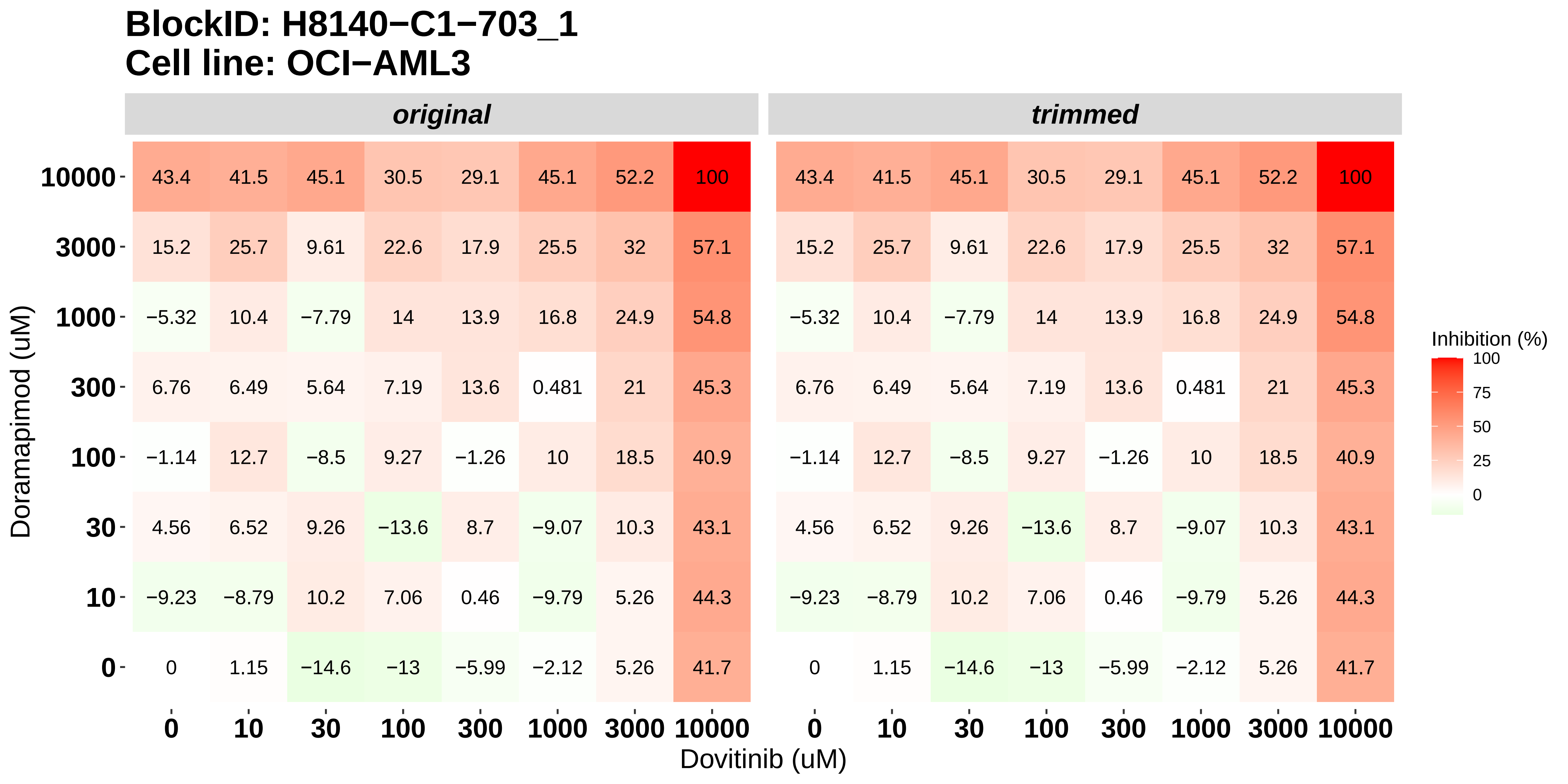
BlockID: H8140−C1−703_1
Cell line: OCI−AML3
original
trimmed
Doramapimod (uM)
Dovitinib (uM)
Inhibition (%)

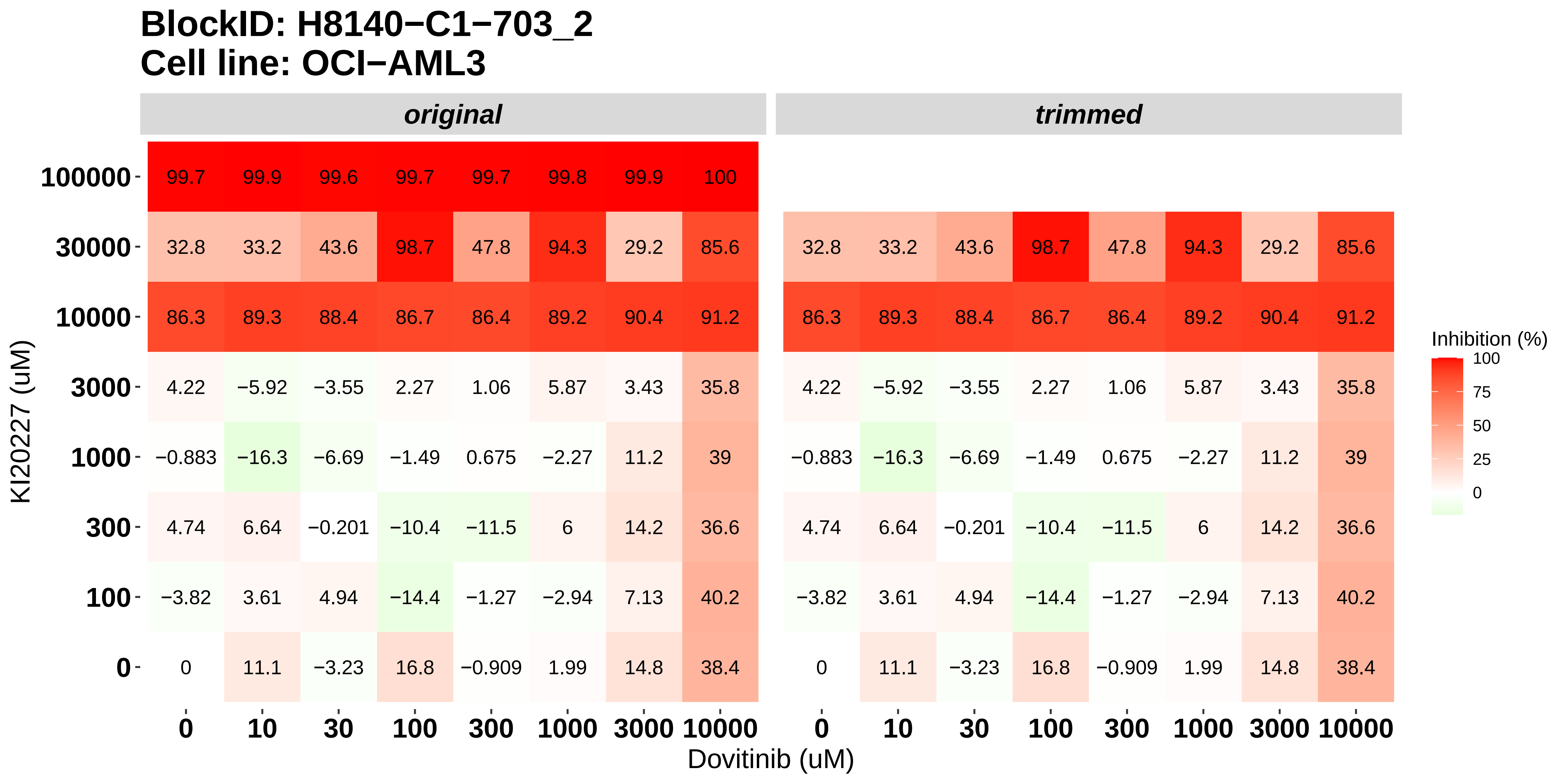
BlockID: H8140−C1−703_2
Cell line: OCI−AML3
original
trimmed
KI20227 (uM)
Dovitinib (uM)
Inhibition (%)
100000
30000
10000
3000
1000
300
100
0
0
10
30
100
300
1000
3000
10000
99.7 99.9 99.6 99.7 99.7 99.8 99.9 100
32.8 33.2 43.6 98.7 47.8 94.3 29.2 85.6
86.3 89.3 88.4 86.7 86.4 89.2 90.4 91.2
4.22 −5.92 −3.55 2.27 1.06 5.87 3.43 35.8
−0.883 −16.3 −6.69 −1.49 0.675 −2.27 11.2 39
4.74 6.64 −0.201 −10.4 −11.5 6 14.2 36.6
−3.82 3.61 4.94 −14.4 −1.27 −2.94 7.13 40.2
0 11.1 −3.23 16.8 −0.909 1.99 14.8 38.4
100
75
50
25
0

BlockID: H8140−C1−703_3
Cell line: OCI−AML3

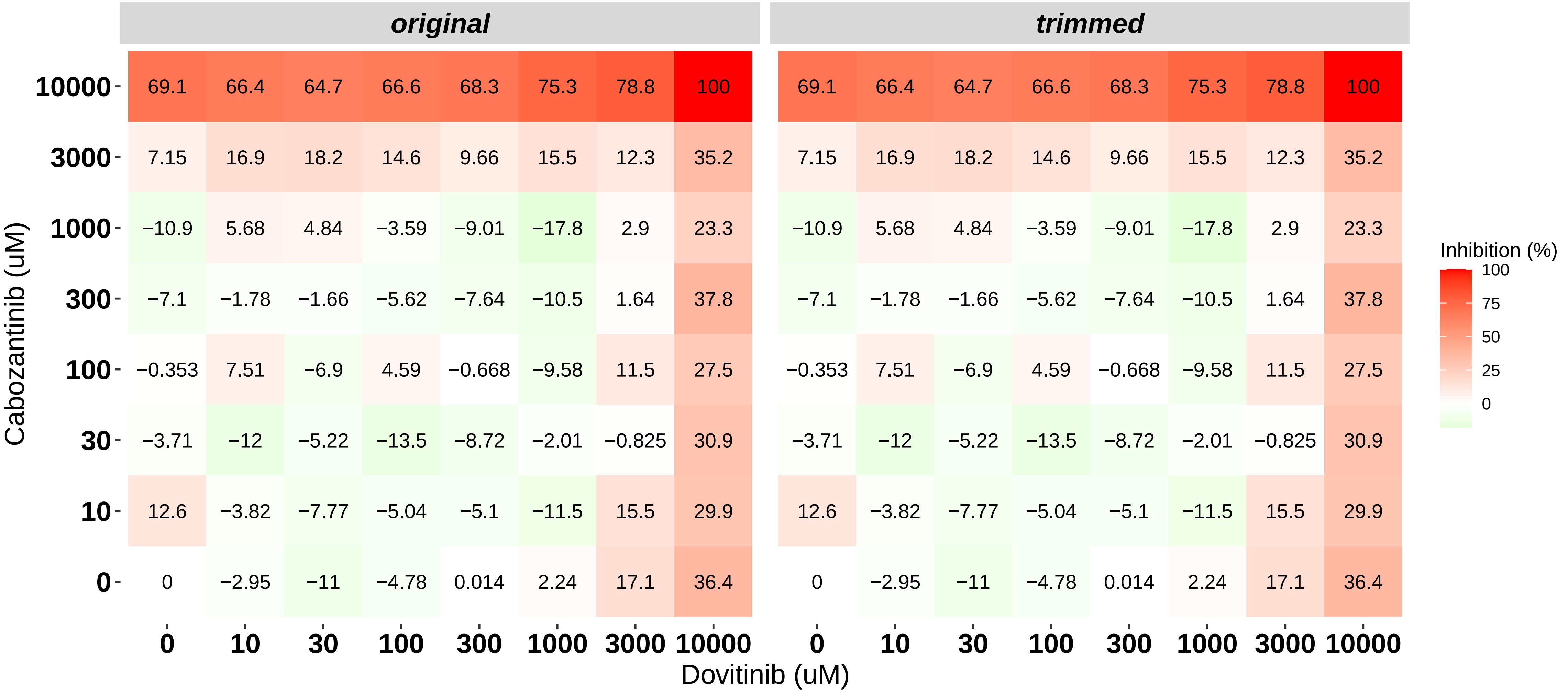

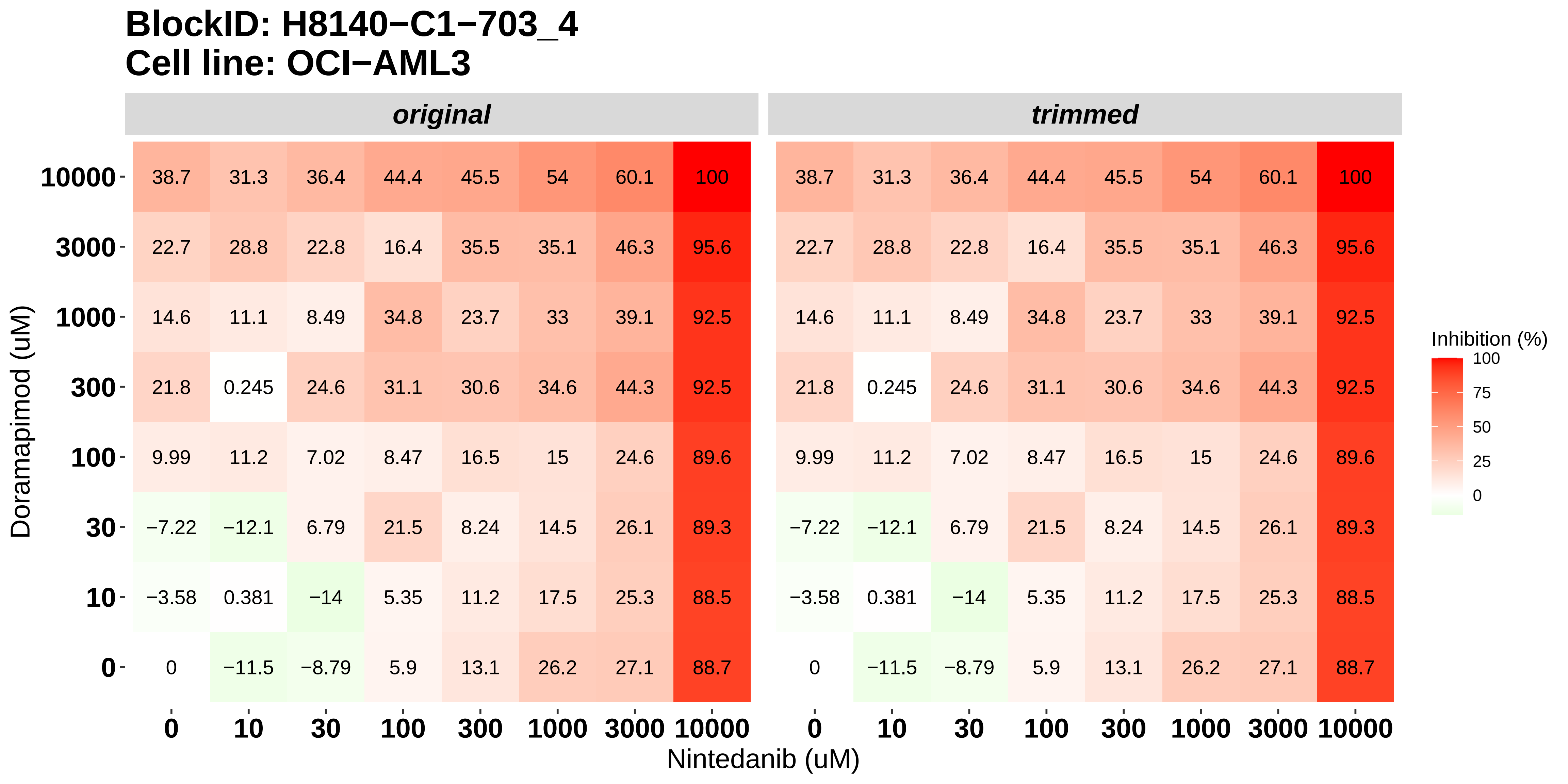
BlockID: H8140−C1−703_4
Cell line: OCI−AML3
original
trimmed
Doramapimod (uM)
Nintedanib (uM)
Inhibition (%)
10000
3000
1000
300
100
30
10
0
38.7 31.3 36.4 44.4 45.5 54 60.1 100
22.7 28.8 22.8 16.4 35.5 35.1 46.3 95.6
14.6 11.1 8.49 34.8 23.7 33 39.1 92.5
21.8 0.245 24.6 31.1 30.6 34.6 44.3 92.5
9.99 11.2 7.02 8.47 16.5 15 24.6 89.6
−7.22 −12.1 6.79 21.5 8.24 14.5 26.1 89.3
−3.58 0.381 −14 5.35 11.2 17.5 25.3 88.5
0 −11.5 −8.79 5.9 13.1 26.2 27.1 88.7
0 10 30 100 300 1000 3000 10000
100
75
50
25
0

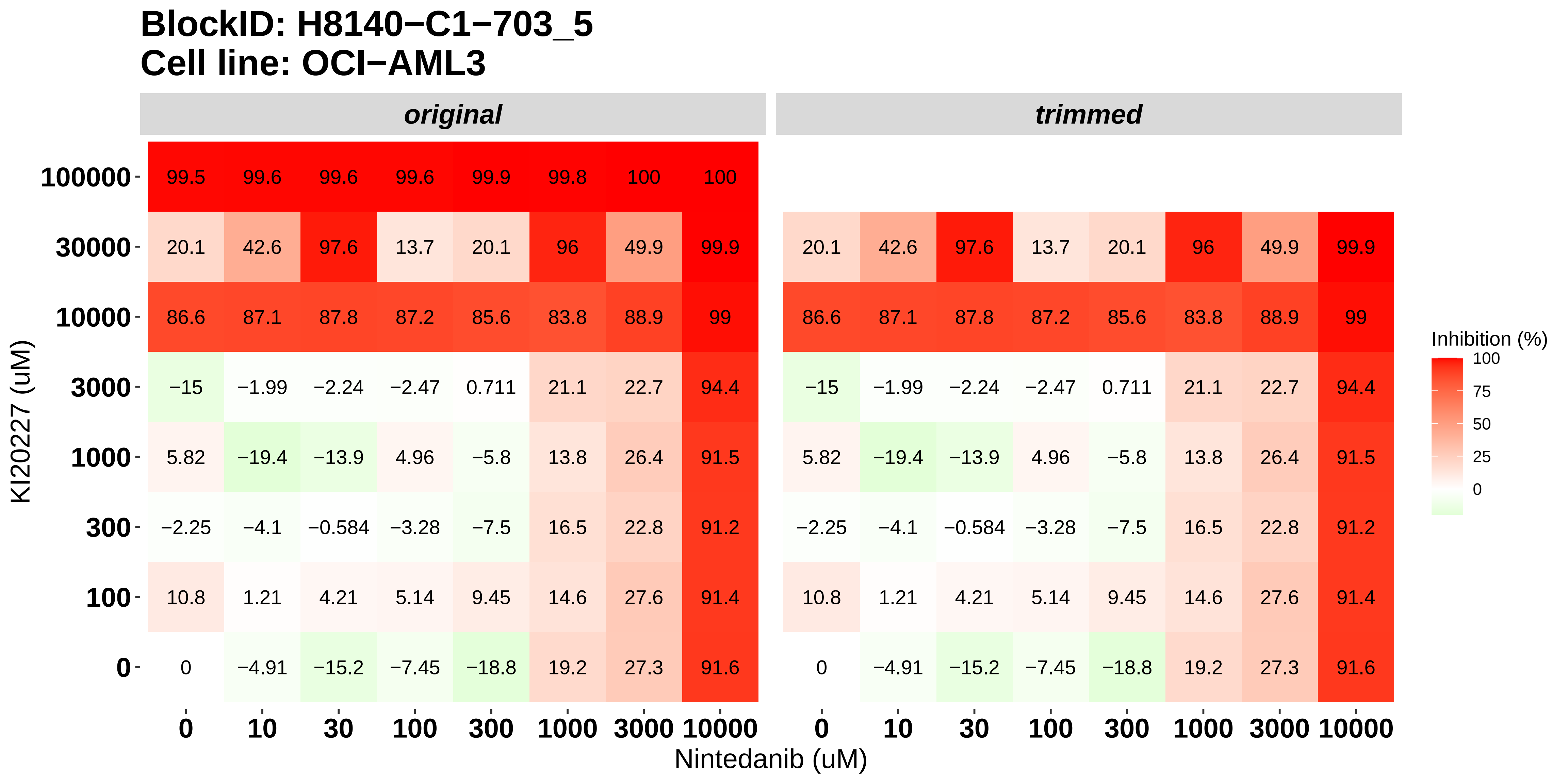

BlockID: H8140−C1−703_5
Cell line: OCI−AML3
original
trimmed
100000
30000
10000
3000
1000
300
100
0
KI20227 (uM)
99.5 99.6 99.6 99.6 99.9 99.8 100 100
20.1 42.6 97.6 13.7 20.1 96 49.9 99.9
86.6 87.1 87.8 87.2 85.6 83.8 88.9 99
−15 −1.99 −2.24 −2.47 0.711 21.1 22.7 94.4
5.82 −19.4 −13.9 4.96 −5.8 13.8 26.4 91.5
−2.25 −4.1 −0.584 −3.28 −7.5 16.5 22.8 91.2
10.8 1.21 4.21 5.14 9.45 14.6 27.6 91.4
0 −4.91 −15.2 −7.45 −18.8 19.2 27.3 91.6
20.1 42.6 97.6 13.7 20.1 96 49.9 99.9
86.6 87.1 87.8 87.2 85.6 83.8 88.9 99
−15 −1.99 −2.24 −2.47 0.711 21.1 22.7 94.4
5.82 −19.4 −13.9 4.96 −5.8 13.8 26.4 91.5
−2.25 −4.1 −0.584 −3.28 −7.5 16.5 22.8 91.2
10.8 1.21 4.21 5.14 9.45 14.6 27.6 91.4
0 −4.91 −15.2 −7.45 −18.8 19.2 27.3 91.6
0 10 30 100 300 1000 3000 10000
0 10 30 100 300 1000 3000 10000
Nintedanib (uM)
Inhibition (%)
100
75
50
25
0

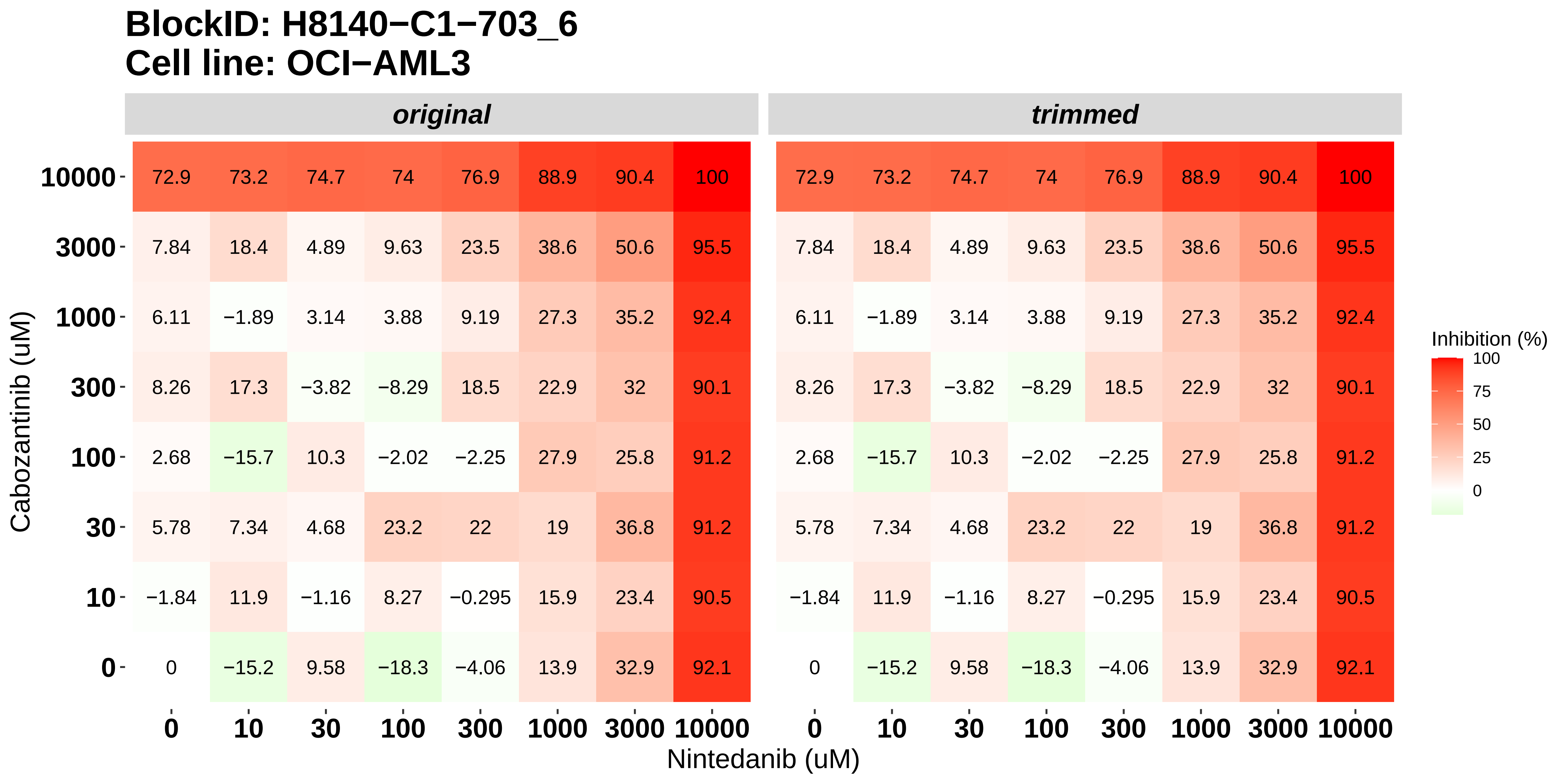

BlockID: H8140−C1−703_6
Cell line: OCI−AML3
original
trimmed
Cabozantinib (uM)
Nintedanib (uM)
Inhibition (%)
100
75
50
25
0
10000
3000
1000
300
100
30
10
0
72.9 73.2 74.7 74 76.9 88.9 90.4 100
7.84 18.4 4.89 9.63 23.5 38.6 50.6 95.5
6.11 −1.89 3.14 3.88 9.19 27.3 35.2 92.4
8.26 17.3 −3.82 −8.29 18.5 22.9 32 90.1
2.68 −15.7 10.3 −2.02 −2.25 27.9 25.8 91.2
5.78 7.34 4.68 23.2 22 19 36.8 91.2
−1.84 11.9 −1.16 8.27 −0.295 15.9 23.4 90.5
0 −15.2 9.58 −18.3 −4.06 13.9 32.9 92.1

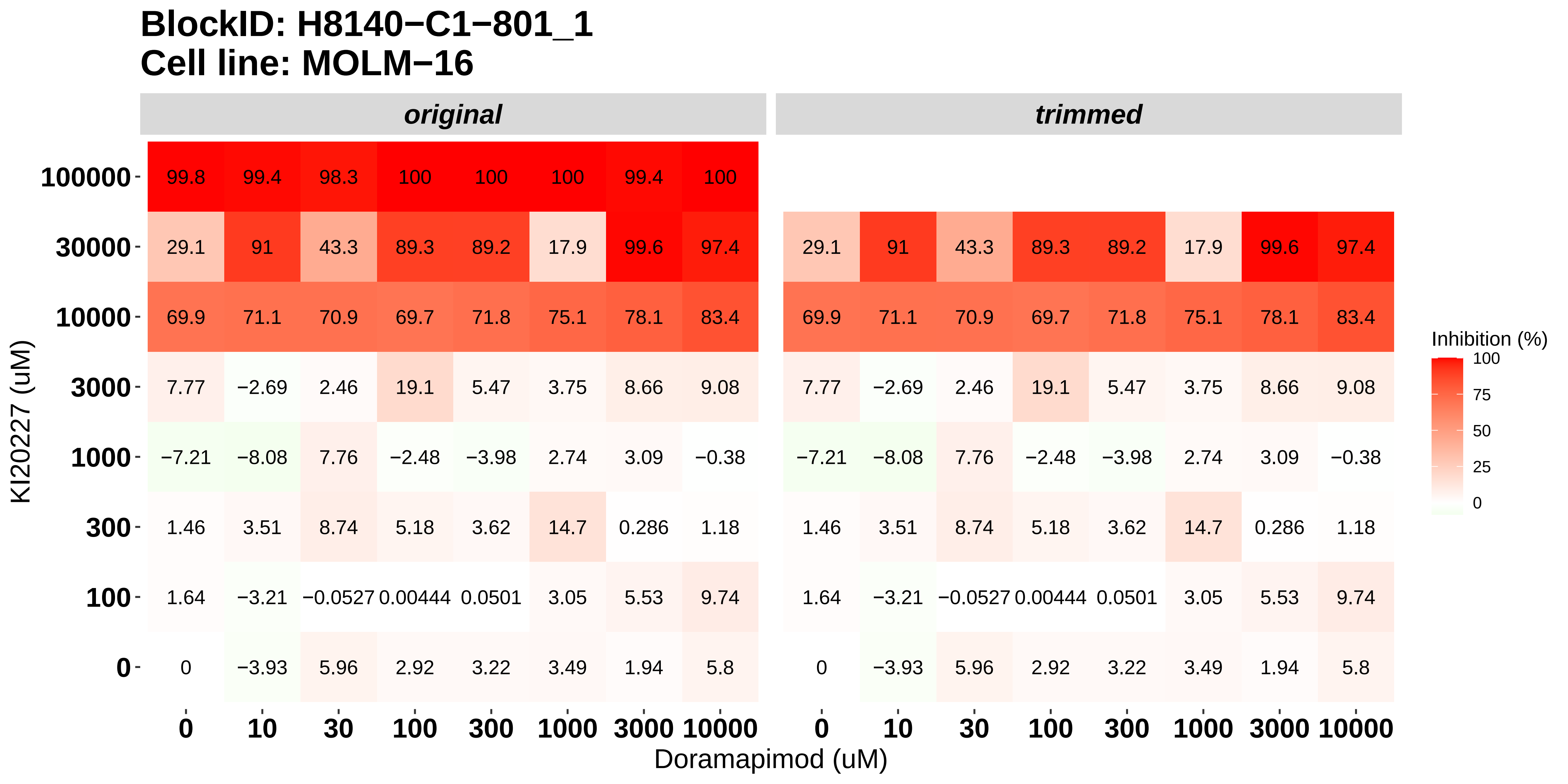

BlockID: H8140−C1−801_1
Cell line: MOLM−16
original
trimmed
KI20227 (uM)
100000
30000
10000
3000
1000
300
100
0
99.8 99.4 98.3 100 100 100 99.4 100
29.1 91 43.3 89.3 89.2 17.9 99.6 97.4
69.9 71.1 70.9 69.7 71.8 75.1 78.1 83.4
7.77 −2.69 2.46 19.1 5.47 3.75 8.66 9.08
−7.21 −8.08 7.76 −2.48 −3.98 2.74 3.09 −0.38
1.46 3.51 8.74 5.18 3.62 14.7 0.286 1.18
1.64 −3.21 −0.0527 0.00444 0.0501 3.05 5.53 9.74
0 −3.93 5.96 2.92 3.22 3.49 1.94 5.8
29.1 91 43.3 89.3 89.2 17.9 99.6 97.4
69.9 71.1 70.9 69.7 71.8 75.1 78.1 83.4
7.77 −2.69 2.46 19.1 5.47 3.75 8.66 9.08
−7.21 −8.08 7.76 −2.48 −3.98 2.74 3.09 −0.38
1.46 3.51 8.74 5.18 3.62 14.7 0.286 1.18
1.64 −3.21 −0.0527 0.00444 0.0501 3.05 5.53 9.74
0 −3.93 5.96 2.92 3.22 3.49 1.94 5.8
0 10 30 100 300 1000 3000 10000
0 10 30 100 300 1000 3000 10000
Doramapimod (uM)
Inhibition (%)
100
75
50
25
0

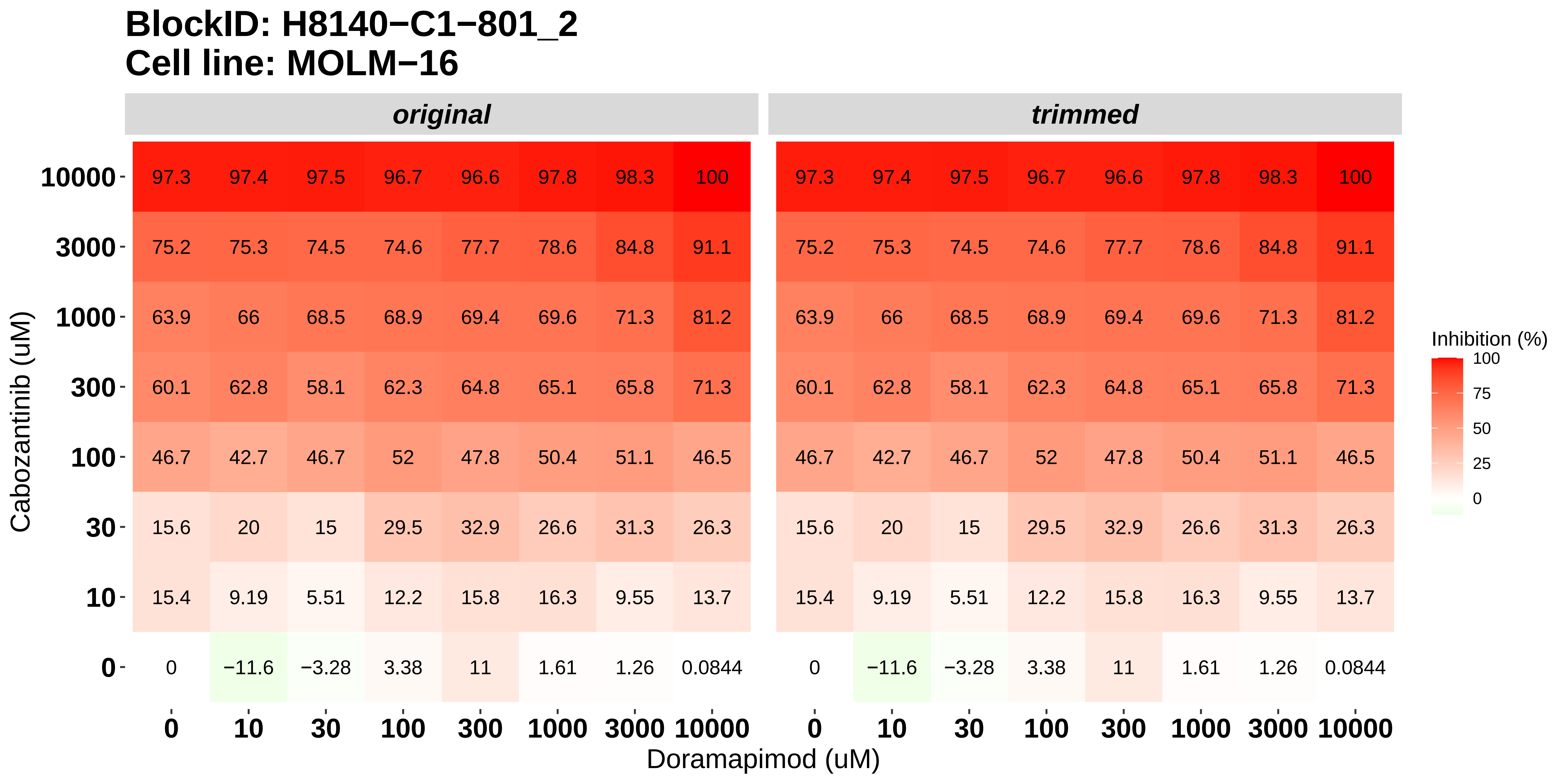
BlockID: H8140−C1−801_2
Cell line: MOLM−16
original
trimmed
Cabozantinib (uM)
10000 3000 1000 300 100 30 10 0
Doramapimod (uM)
0 10 30 100 300 1000 3000 10000
97.3 97.4 97.5 96.7 96.6 97.8 98.3 100
75.2 75.3 74.5 74.6 77.7 78.6 84.8 91.1
63.9 66 68.5 68.9 69.4 69.6 71.3 81.2
60.1 62.8 58.1 62.3 64.8 65.1 65.8 71.3
46.7 42.7 46.7 52 47.8 50.4 51.1 46.5
15.6 20 15 29.5 32.9 26.6 31.3 26.3
15.4 9.19 5.51 12.2 15.8 16.3 9.55 13.7
0 −11.6 −3.28 3.38 11 1.61 1.26 0.0844
Inhibition (%)
100 75 50 25 0

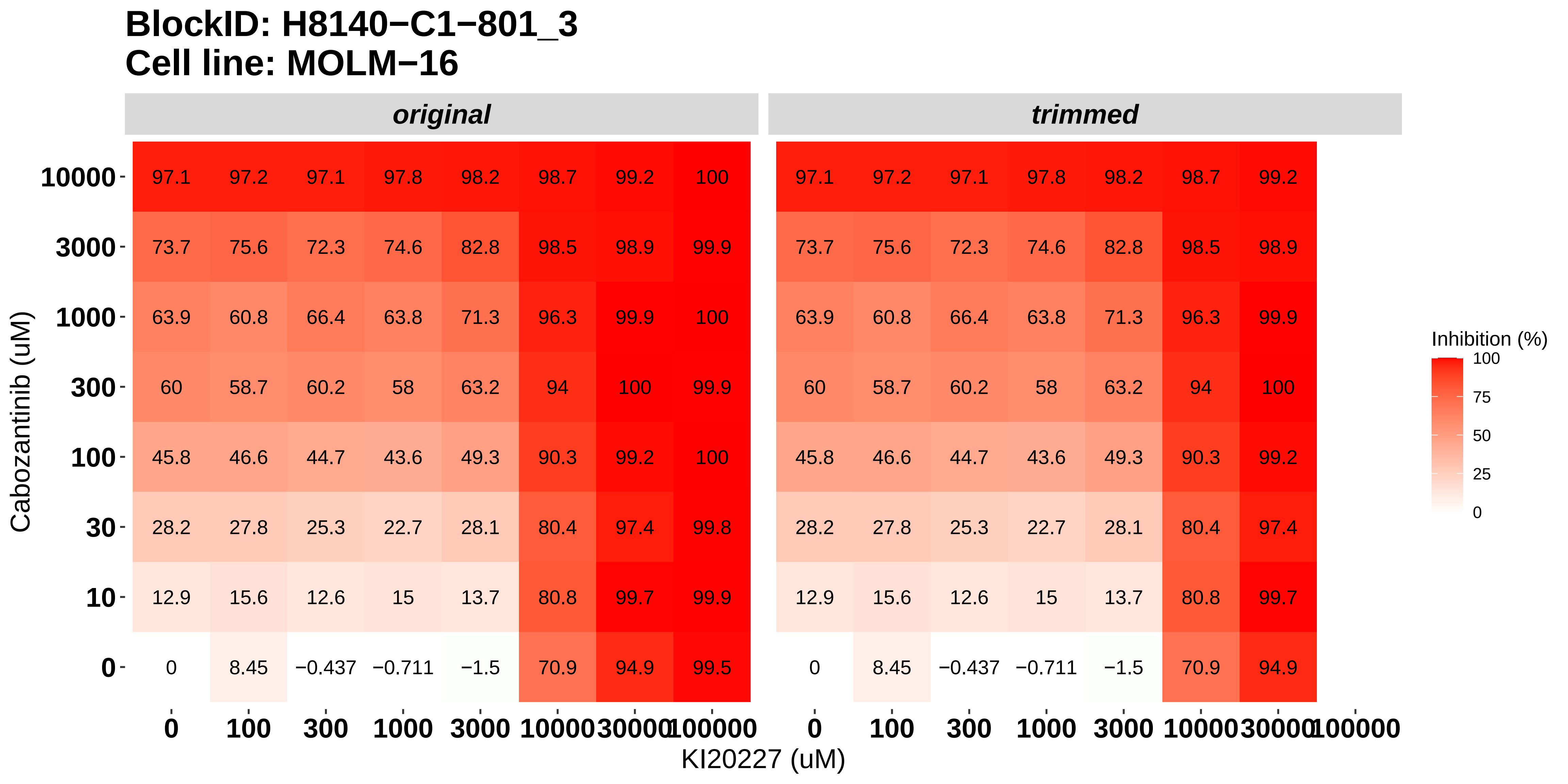
BlockID: H8140−C1−801_3
Cell line: MOLM−16
original
trimmed
Cabozantinib (uM)
KI20227 (uM)
Inhibition (%)
100
75
50
25
0
10000
3000
1000
300
100
30
10
0
0
100
300
1000
3000
10000
30000
100000
97.1 97.2 97.1 97.8 98.2 98.7 99.2 100
73.7 75.6 72.3 74.6 82.8 98.5 98.9 99.9
63.9 60.8 66.4 63.8 71.3 96.3 99.9 100
60 58.7 60.2 58 63.2 94 100 99.9
45.8 46.6 44.7 43.6 49.3 90.3 99.2 100
28.2 27.8 25.3 22.7 28.1 80.4 97.4 99.8
12.9 15.6 12.6 15 13.7 80.8 99.7 99.9
0 8.45 −0.437 −0.711 −1.5 70.9 94.9 99.5
97.1 97.2 97.1 97.8 98.2 98.7 99.2
73.7 75.6 72.3 74.6 82.8 98.5 98.9
63.9 60.8 66.4 63.8 71.3 96.3 99.9
60 58.7 60.2 58 63.2 94 100
45.8 46.6 44.7 43.6 49.3 90.3 99.2
28.2 27.8 25.3 22.7 28.1 80.4 97.4
12.9 15.6 12.6 15 13.7 80.8 99.7
0 8.45 −0.437 −0.711 −1.5 70.9 94.9

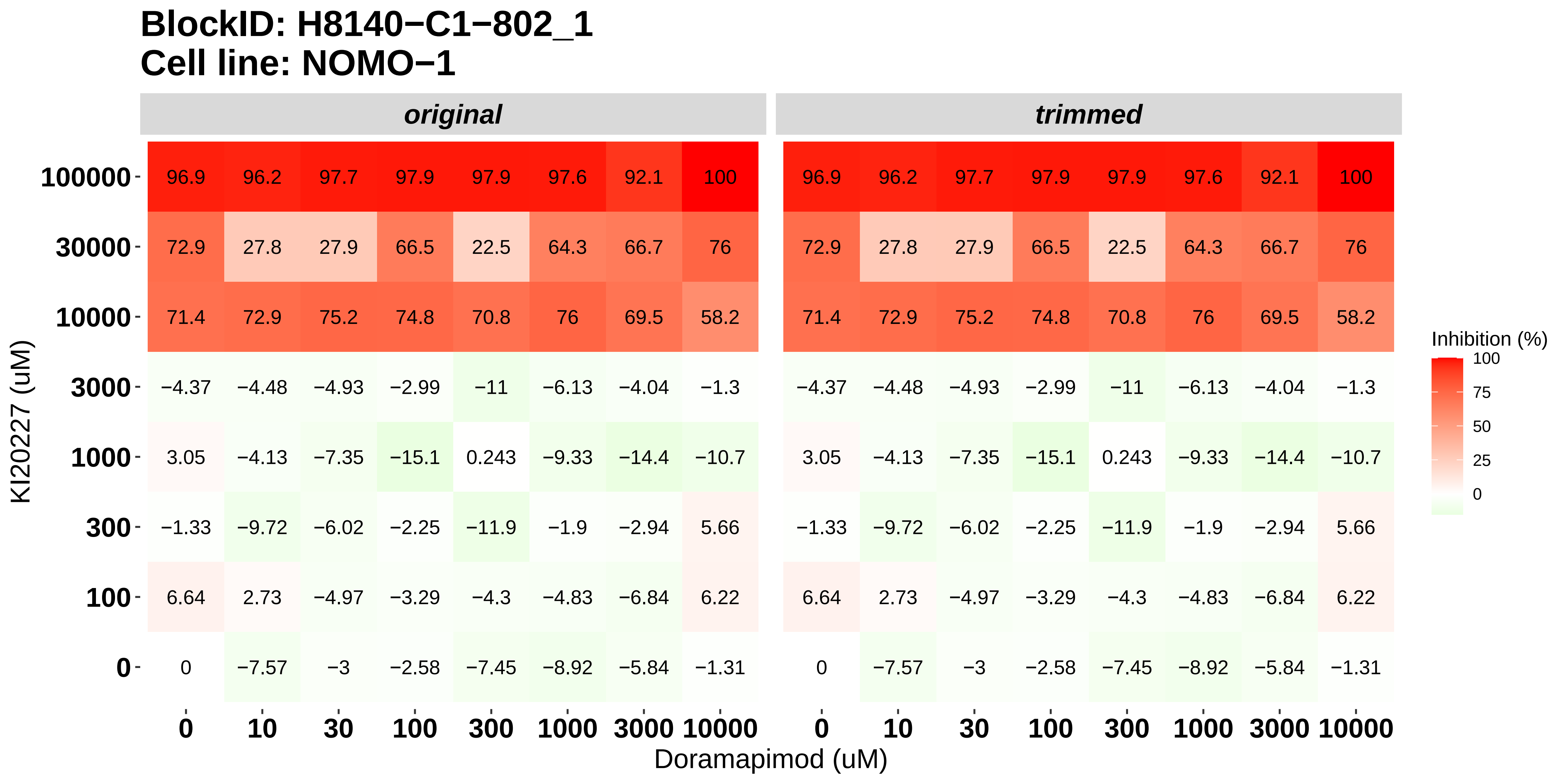
BlockID: H8140−C1−802_1
Cell line: NOMO−1
original
trimmed
KI20227 (uM)
100000
30000
10000
3000
1000
300
100
0
96.9 96.2 97.7 97.9 97.9 97.6 92.1 100
72.9 27.8 27.9 66.5 22.5 64.3 66.7 76
71.4 72.9 75.2 74.8 70.8 76 69.5 58.2
−4.37 −4.48 −4.93 −2.99 −11 −6.13 −4.04 −1.3
3.05 −4.13 −7.35 −15.1 0.243 −9.33 −14.4 −10.7
−1.33 −9.72 −6.02 −2.25 −11.9 −1.9 −2.94 5.66
6.64 2.73 −4.97 −3.29 −4.3 −4.83 −6.84 6.22
0 −7.57 −3 −2.58 −7.45 −8.92 −5.84 −1.31
0 10 30 100 300 1000 3000 10000
Doramapimod (uM)
Inhibition (%)
100
75
50
25
0

BlockID: H8140−C1−802_2
Cell line: NOMO−1

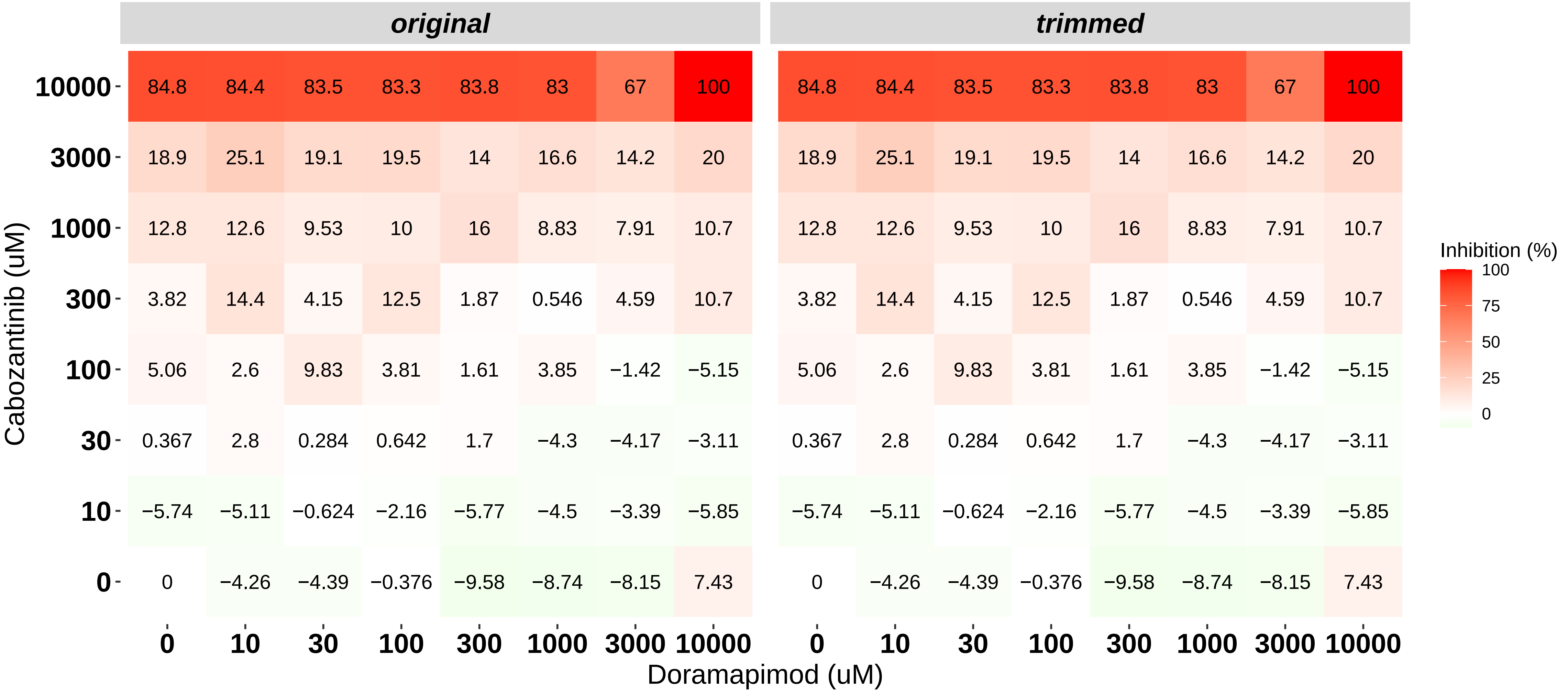

BlockID: H8140−C1−802_3
Cell line: NOMO−1

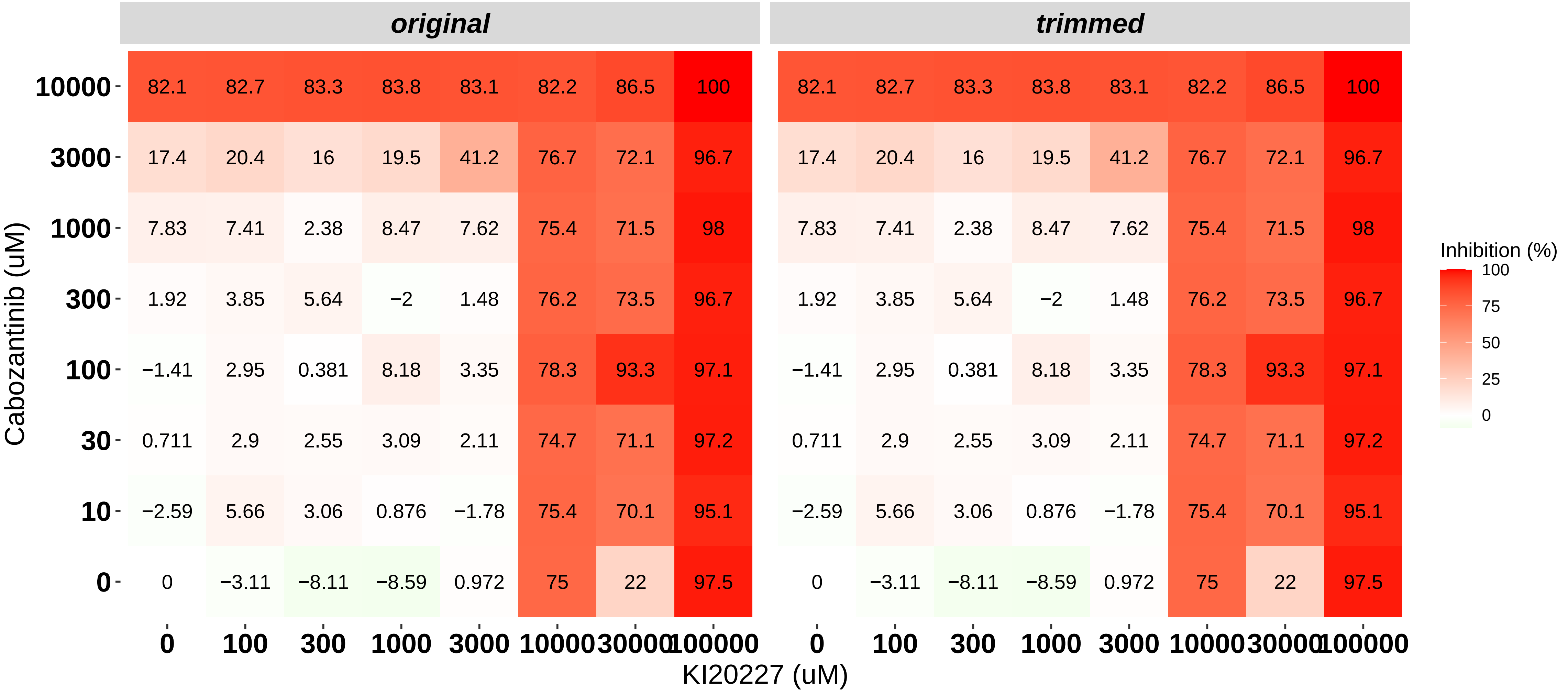

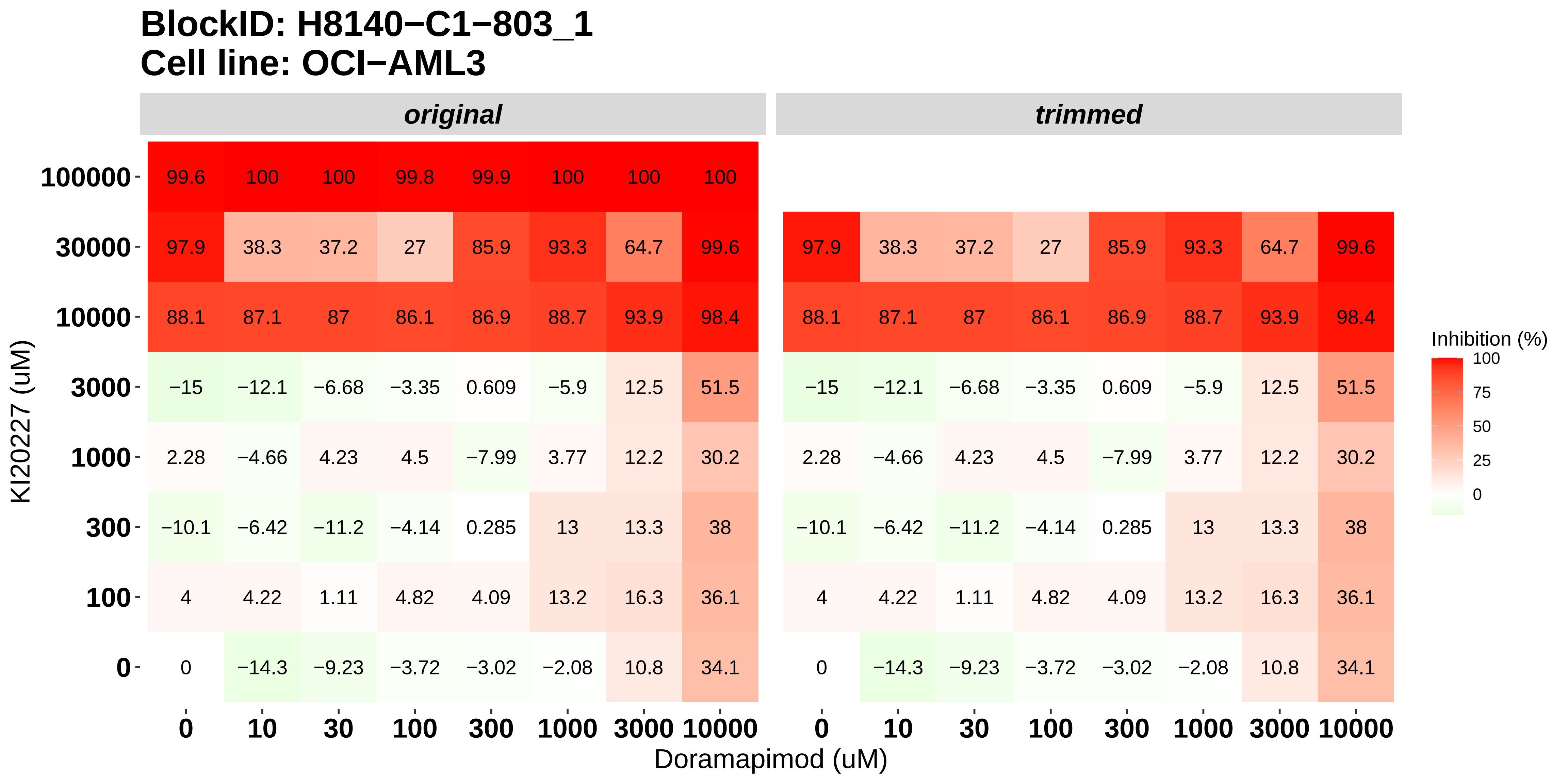
BlockID: H8140−C1−803_1
Cell line: OCI−AML3
original
trimmed
KI20227 (uM)
Doramapimod (uM)
Inhibition (%)
100000
30000
10000
3000
1000
300
100
0
10
30
100
300
1000
3000
10000
99.6 100 100 99.8 99.9 100 100 100
97.9 38.3 37.2 27 85.9 93.3 64.7 99.6
88.1 87.1 87 86.1 86.9 88.7 93.9 98.4
−15 −12.1 −6.68 −3.35 0.609 −5.9 12.5 51.5
2.28 −4.66 4.23 4.5 −7.99 3.77 12.2 30.2
−10.1 −6.42 −11.2 −4.14 0.285 13 13.3 38
4 4.22 1.11 4.82 4.09 13.2 16.3 36.1
0 −14.3 −9.23 −3.72 −3.02 −2.08 10.8 34.1
100
75
50
25
0

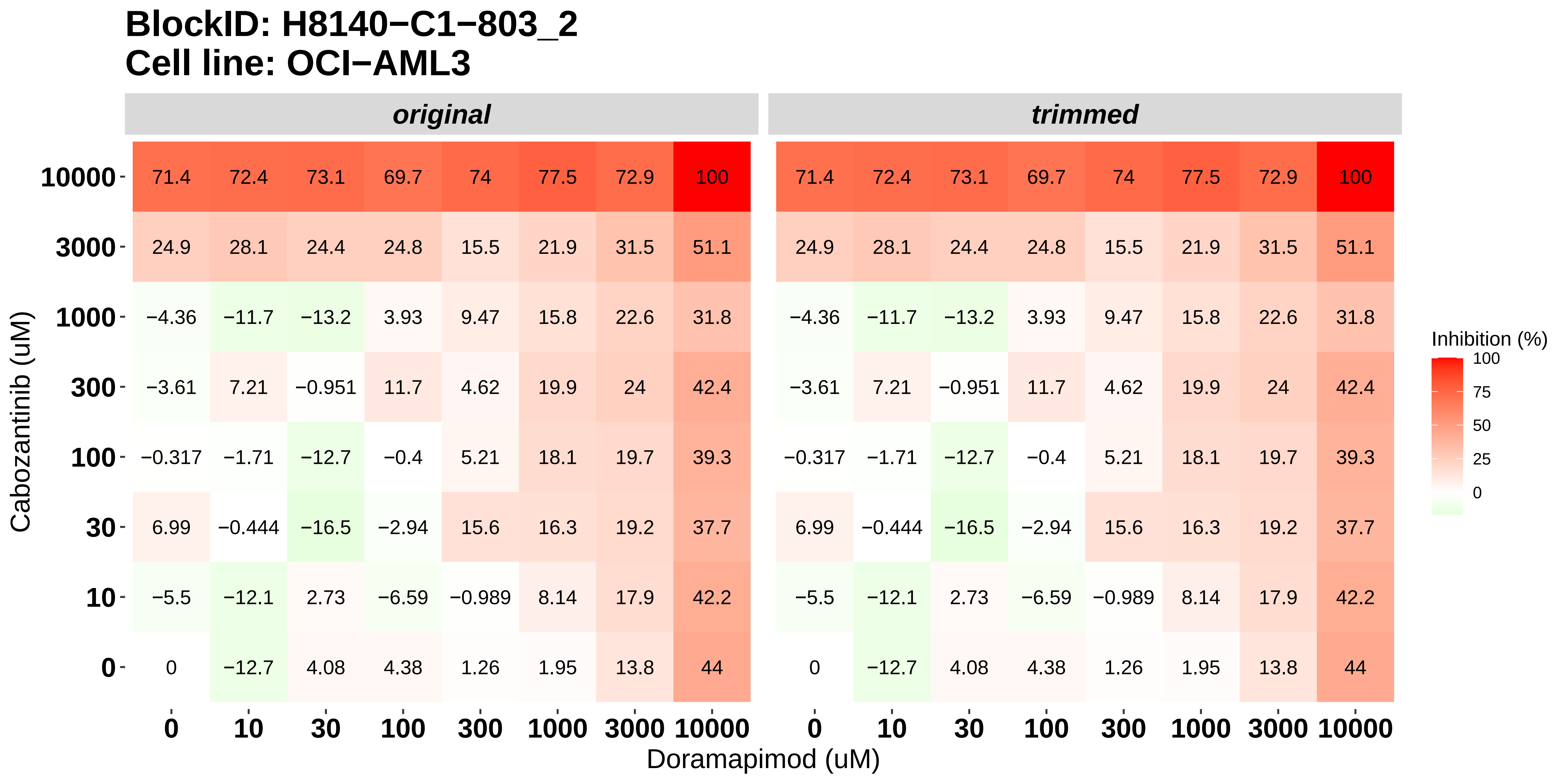
BlockID: H8140−C1−803_2
Cell line: OCI−AML3
original
trimmed
10000 71.4 72.4 73.1 69.7 74 77.5 72.9 100
3000 24.9 28.1 24.4 24.8 15.5 21.9 31.5 51.1
1000 −4.36 −11.7 −13.2 3.93 9.47 15.8 22.6 31.8
300 −3.61 7.21 −0.951 11.7 4.62 19.9 24 42.4
100 −0.317 −1.71 −12.7 −0.4 5.21 18.1 19.7 39.3
30 6.99 −0.444 −16.5 −2.94 15.6 16.3 19.2 37.7
10 −5.5 −12.1 2.73 −6.59 −0.989 8.14 17.9 42.2
0 0 −12.7 4.08 4.38 1.26 1.95 13.8 44
0 10 30 100 300 1000 3000 10000
Cabozantinib (uM)
Doramapimod (uM)
Inhibition (%)
100
75
50
25
0

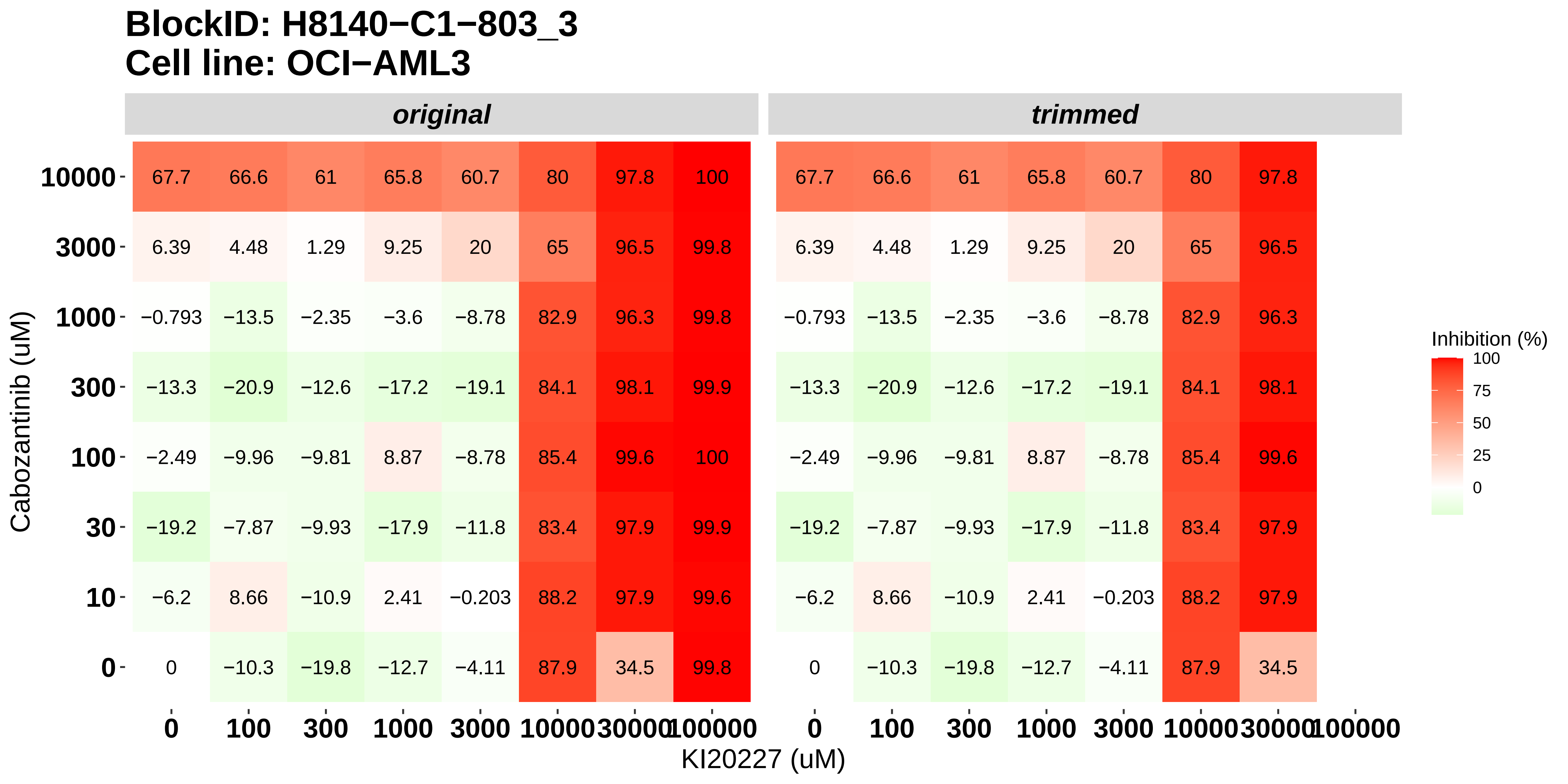
BlockID: H8140−C1−803_3
Cell line: OCI−AML3
original
trimmed
Cabozantinib (uM)
10000
3000
1000
300
100
30
10
0
67.7 66.6 61 65.8 60.7 80 97.8 100
6.39 4.48 1.29 9.25 20 65 96.5 99.8
−0.793 −13.5 −2.35 −3.6 −8.78 82.9 96.3 99.8
−13.3 −20.9 −12.6 −17.2 −19.1 84.1 98.1 99.9
−2.49 −9.96 −9.81 8.87 −8.78 85.4 99.6 100
−19.2 −7.87 −9.93 −17.9 −11.8 83.4 97.9 99.9
−6.2 8.66 −10.9 2.41 −0.203 88.2 97.9 99.6
0 −10.3 −19.8 −12.7 −4.11 87.9 34.5 99.8
67.7 66.6 61 65.8 60.7 80 97.8
6.39 4.48 1.29 9.25 20 65 96.5
−0.793 −13.5 −2.35 −3.6 −8.78 82.9 96.3
−13.3 −20.9 −12.6 −17.2 −19.1 84.1 98.1
−2.49 −9.96 −9.81 8.87 −8.78 85.4 99.6
−19.2 −7.87 −9.93 −17.9 −11.8 83.4 97.9
−6.2 8.66 −10.9 2.41 −0.203 88.2 97.9
0 −10.3 −19.8 −12.7 −4.11 87.9 34.5
0 100 300 1000 3000 10000 30000 100000
0 100 300 1000 3000 10000 30000 100000
KI20227 (uM)
Inhibition (%)
100
75
50
25
0

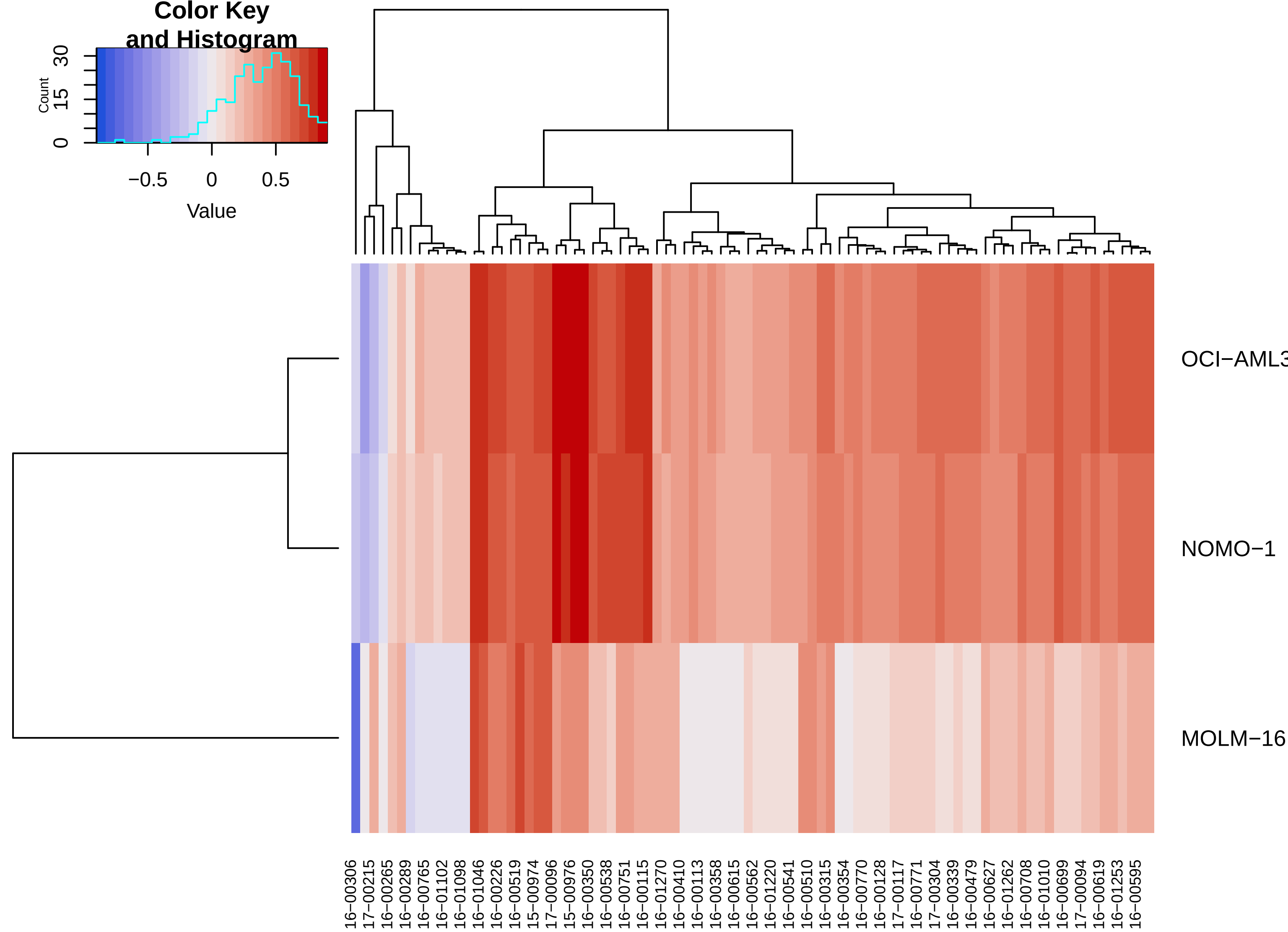
Color Key
and Histogram
Count
0
15
30
−0.5
0
0.5
Value
OCI−AML3
NOMO−1
MOLM−16
16−00306
17−00215
16−00265
16−00289
16−00765
16−01102
16−01098
16−01046
16−00226
16−00519
15−00974
17−00096
15−00976
16−00350
16−00538
16−00751
16−00115
16−01270
16−00410
16−00113
16−00358
16−00615
16−00562
16−01220
16−00541
16−00510
16−00315
16−00354
16−00770
16−00128
17−00117
16−00771
17−00304
16−00339
16−00479
16−00627
16−01262
16−00708
16−01010
16−00699
17−00094
16−00619
16−01253
16−00595
