# Supplementary material for: Bipartite network models to design combination therapies in acute myeloid leukaemia
Source: Nat Commun. 2022 Apr 19;13:2128. doi: 10.1038/s41467-022-29793-5 (PMC9018865; doi:10.1038/s41467-022-29793-5)
Supplement: Supplementary file 3 — Reporting Summary [file 41467_2022_29793_MOESM3_ESM.pdf]

nature portfolio

Corresponding author(s): Mohieddin Jafari, Jing Tang

Last updated by author(s): Feb 10, 2022

Reporting Summary

Nature Portfolio wishes to improve the reproducibility of the work that we publish. This form provides structure for consistency and transparency in reporting. For further information on Nature Portfolio policies, see our Editorial Policies and the Editorial Policy Checklist.

Statistics

For all statistical analyses, confirm that the following items are present in the figure legend, table legend, main text, or Methods section.

n/a	Confirmed	
☐	☒	The exact sample size (n) for each experimental group/condition, given as a discrete number and unit of measurement
☐	☒	A statement on whether measurements were taken from distinct samples or whether the same sample was measured repeatedly
☐	☒	The statistical test(s) used AND whether they are one- or two-sided<br>*Only common tests should be described solely by name; describe more complex techniques in the Methods section.*
☒	☐	A description of all covariates tested
☐	☒	A description of any assumptions or corrections, such as tests of normality and adjustment for multiple comparisons
☐	☒	A full description of the statistical parameters including central tendency (e.g. means) or other basic estimates (e.g. regression coefficient) AND variation (e.g. standard deviation) or associated estimates of uncertainty (e.g. confidence intervals)
☐	☒	For null hypothesis testing, the test statistic (e.g. F, t, r) with confidence intervals, effect sizes, degrees of freedom and P value noted<br>*Give P values as exact values whenever suitable.*
☒	☐	For Bayesian analysis, information on the choice of priors and Markov chain Monte Carlo settings
☒	☐	For hierarchical and complex designs, identification of the appropriate level for tests and full reporting of outcomes
☐	☒	Estimates of effect sizes (e.g. Cohen's d, Pearson's r), indicating how they were calculated

Our web collection on statistics for biologists contains articles on many of the points above.

Software and code

Policy information about availability of computer code

Data collection	No software was used for data collection.
Data analysis	The R software (v. 4.0.0) and the corresponding packages (including "AMR", "car", "CINNA", "cowplot", "fingerprint", "GGally", "ggcorrplot", "ggpubr", "ggrepel", "ggridges", "igraph", "janitor", "minerva", "network", "OmnipathR", "rcdk", "readxl", "rstatix", "tidyverse", "visNetwork", and "webshot") were used in this study. The details are provided in this link (Direct link: https://doi.org/10.5281/zenodo.5789170).

For manuscripts utilizing custom algorithms or software that are central to the research but not yet described in published literature, software must be made available to editors and reviewers. We strongly encourage code deposition in a community repository (e.g. GitHub). See the Nature Portfolio guidelines for submitting code & software for further information.

Data

Policy information about availability of data

All manuscripts must include a data availability statement. This statement should provide the following information, where applicable:

- Accession codes, unique identifiers, or web links for publicly available datasets
- A description of any restrictions on data availability
- For clinical datasets or third party data, please ensure that the statement adheres to our policy

The following databases were used to collect data in the computational part of our study: BeatAML dataset: http://www.vizome.org/aml/, ALMANAC dataset: http//drugcomb.fimm.fi/, GDSC dataset: https://www.cancerrxgene.org/. The authors declare that all other supporting the findings of this study are available within the paper and its supplementary information files (Direct link: https://doi.org/10.5281/zenodo.5789170).

Field-specific reporting

Please select the one below that is the best fit for your research. If you are not sure, read the appropriate sections before making your selection.

☒ Life sciences ☐ Behavioural & social sciences ☐ Ecological, evolutionary & environmental sciences

For a reference copy of the document with all sections, see nature.com/documents/nr-reporting-summary-flat.pdf

Life sciences study design

All studies must disclose on these points even when the disclosure is negative.

Sample size	For statistical testing, the following sample sizes were used: N=17280 (number of separate dose-response matrix entries) for predicted dose-response values and N=270 (number of distinct dose-response matrices) for prediction of synergy scores. Generally, sample sizes are deemed sufficient for conducting systematic comparisons of predictive algorithms' capability to predict the actions of suggested drug combinations. There was no formal statistical power analysis because the study's emphasis was on predictive power rather than statistical power.
Data exclusions	In the computational section of this paper, we use a subset of datasets without missing values. Our experimental findings do not consider any exclusions.
Replication	We utilized duplicate technical values in this project. In addition, 9 replicates were conducted for every single drug screening. All attempts at replication were successful.
Randomization	We did not utilize randomization in our experimental design since it is impractical. In other words, with sufficient controls and a machine-read experimental readout, the risk of bias may not be regarded high enough to justify allowing the risk of human error in such an in vitro study with multi-well plates.
Blinding	We adopted full blinding in our experiment, which means that experimenters were unaware of the coding information for the positive and negative groups of drugs from the time of experimental treatment, data collection, and analysis, until the time point for comparing the outcomes of the two groups.

Reporting for specific materials, systems and methods

We require information from authors about some types of materials, experimental systems and methods used in many studies. Here, indicate whether each material, system or method listed is relevant to your study. If you are not sure if a list item applies to your research, read the appropriate section before selecting a response.

Materials & experimental systems

n/a	Involved in the study	
☒	☐	Antibodies
☐	☒	Eukaryotic cell lines
☒	☐	Palaeontology and archaeology
☒	☐	Animals and other organisms
☒	☐	Human research participants
☒	☐	Clinical data
☒	☐	Dual use research of concern

Methods

n/a	Involved in the study	
☒	☐	ChIP-seq
☒	☐	Flow cytometry
☒	☐	MRI-based neuroimaging

Eukaryotic cell lines

Policy information about cell lines

Cell line source(s)	AML cell lines MOLM-16,NOMO-1, and OCL-AML3 were purchased from https://www.dsmz.de/dsmz (Leibniz Institute, DSMZ-German Collection of Microorganisms and Cell Cultures).
Authentication	STR analysis according to the global standard ANSI/ATCC ASN-0002.1-2021 (2021) resulted in an authentic STR profile of the reference STR database.
Mycoplasma contamination	Mycoplasma contamination was found to be absent in all cell lines examined using a PCR-based Venor™ GeM Mycoplasma Detection Kit (Minerva Biolabs).
Commonly misidentified lines (See ICLAC register)	No commonly misidentified cell lines were used in the study.
